# Access to Spirooxindole-Fused Cyclopentanes via a Stereoselective Organocascade Reaction Using Bifunctional Catalysis

**DOI:** 10.1021/acs.joc.2c02478

**Published:** 2023-01-27

**Authors:** Andrea Vopálenská, Vojtěch Dočekal, Simona Petrželová, Ivana Císařová, Jan Veselý

**Affiliations:** † Department of Organic Chemistry, Faculty of Science, Charles University, Hlavova 2030/8, 128 43 Prague 2, Czech Republic; ‡ Department of Teaching and Didactics of Chemistry, Faculty of Science, Charles University, Hlavova 2030/8, 128 43 Prague 2, Czech Republic; § Department of Inorganic Chemistry, Faculty of Science, Charles University, Hlavova 2030/8, 128 43 Prague 2, Czech Republic

## Abstract

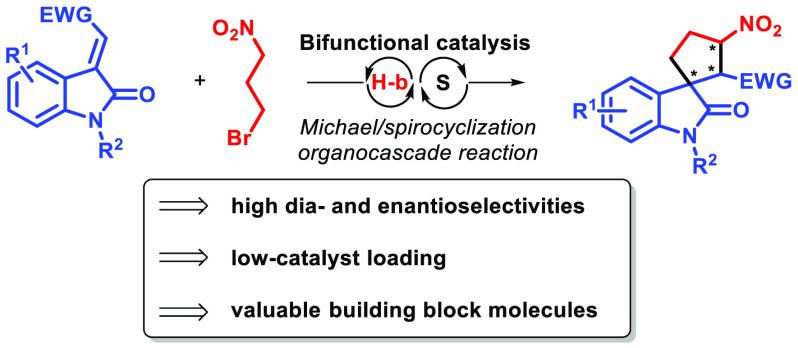

The present study reports an asymmetric organocascade reaction of oxindole-derived alkenes with 3-bromo-1-nitropropane efficiently catalyzed by the bifunctional catalyst. Spirooxindole-fused cyclopentanes were produced in moderate-to-good isolated yields (15–69%) with excellent stereochemical outcomes. The synthetic utility of the protocol was exemplified on a set of additional transformations of the corresponding spirooxindole compounds.

## Introduction

**Figure 1:**
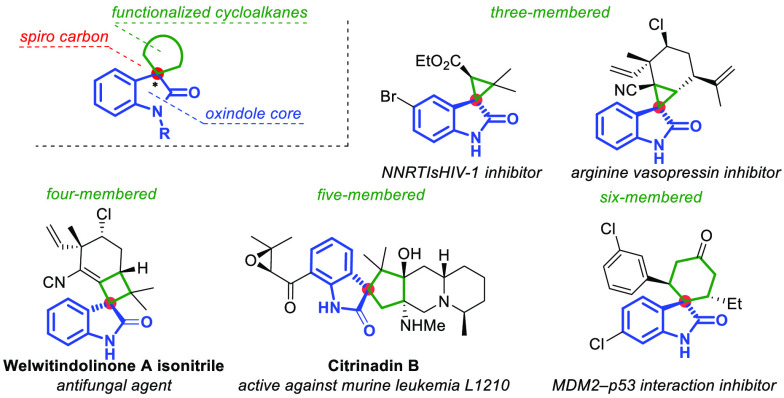
Selected biologically active spirooxindoles.

Nowadays, cascade reactions (or domino reactions)^1^ represent a formidable challenge for modern synthetic chemistry.^2^ Those reactions are generally described as multicomponent one-pot processes involving two or more transformations. That strategy offers many advantages over classical “stop-and-go” sequences, for example, in avoiding protecting groups or isolation of reaction intermediates. Besides operational efficiency (step and pot economy),^3^ organocascade reactions showed significant advantages for constructing complex molecular frameworks with high selectivity levels (stereo-, chemo-). Not surprisingly, organocascade reactions were successfully used for the stereoselective preparation of valuable spirocyclic compounds,^4^ for example, the privileged scaffold—spirocyclic oxindole derivatives (spirooxindoles).^5^ The spirooxindole structural motif appears as part of various natural or synthetic compounds with remarkable biological activity, including medicinally relevant compounds (Figure 1).^6^

**Scheme 1:**
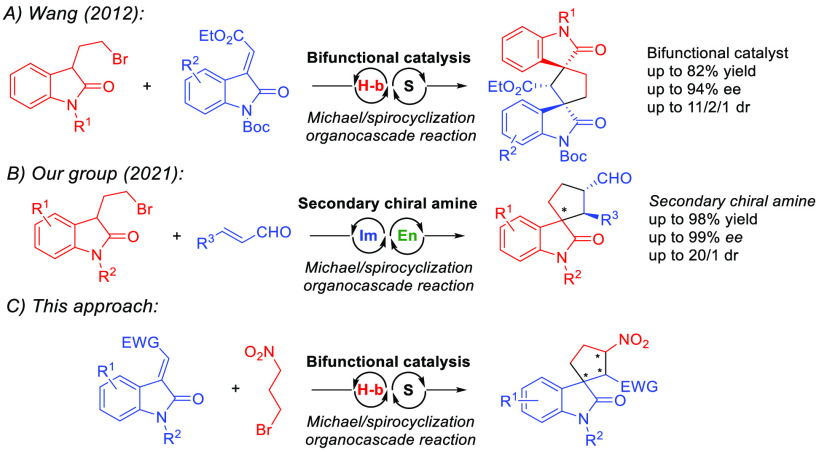
Examples of Organocascade Approaches toward Spirooxindole-Fused Cyclopentanes

Organocascade reactions initiated by the Michael reaction are highly efficient for the construction of spirooxindole-fused derivatives,^7^ using either oxindoles with nucleophilic C3 (Michael donors)^8−10^ or electrophilic methyleneindolinones (Michael acceptors).^11,12^ Interestingly, a combination of both types of starting materials was applied by Wang for the preparation of highly rigid bispirooxindoles via a Michael/spirocyclization reaction promoted by a bifunctional organocatalyst (Scheme 1A).^13^ Recently, our group described the Michael/alkylation organocascade reaction of 3-(2-bromoethyl)oxindoles with α,β-unsaturated aldehydes efficiently catalyzed by a chiral secondary amine, producing valuable spirooxindole-fused cyclopentanes (Scheme 1B).^14^

Considering the above, and in light of our ongoing interest in the enantioselective synthesis of spirocyclic compounds,^15^ we envisioned the construction of novel spirooxindole-fused cyclopentane derivatives having up to three stereocenters via a stereoselective organocascade Michael/spirocyclization reaction promoted by the bifunctional catalyst from 3-bromo-1-nitropropane and methyleneindolinones (Scheme 1C).

## Results and Discussion

To verify our design strategy, we began our study by mixing easily accessible methyleneindolinone **1a** with 3-bromo-1-nitropropane (**2a**), bifunctional organocatalyst, and base (Table 1). To our delight, a reaction conducted with commercially available Takemoto catalyst (**C1**) and K_2_CO_3_ produced spirooxindole derivative **3a** as the main diastereomer. Moreover, compound **3a** was readily separable on silica and isolated in good yield (58%) with high enantioselectivity (99% ee, entry 1). Besides, we observed the formation of **5a** in traces as a product of base-induced HNO_2_ elimination. Conversely, the diastereocontrol of the reactions catalyzed by Rawal’s and Soos’s catalysts was significantly diminished (entries 2 and 3). Apart from **C1**–**C3**, we tested other bifunctional organocatalysts (for details, please see the SI file), but no further improvement in reaction efficiency was observed. Interestingly, the reaction rate was significantly decreased when using Na_2_CO_3_ (entry 4) and NaHCO_3_ (entry 5). Using organic bases, such as DIPEA (entry 6), significantly reduced the diastereocontrol. Then, the effect of solvent on reaction efficiency and the stereochemical outcome was evaluated. Using polar aprotic solvents (ethyl acetate or MTBE) resulted in the highest reaction rates. On the other hand, diastereoselectivities of those reactions were significantly lowered (entries 7 and 8). The model reaction conducted in chloroform (entry 8) produced spirooxindole **3a** in high yield, with excellent stereochemical outcome. Additionally, we conducted the model reaction with a reduced amount of 3-bromo-1-nitropropane (**2a**) and organocatalyst **C1** (1 mol %), producing **3a** with the same efficiency and stereocontrol (entry 11). For complete optimization studies, please see the SI.

**Table 1:**
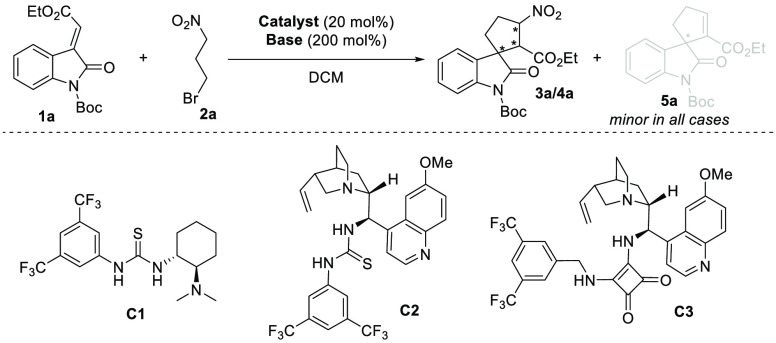
Optimization Studies of Cascade Reaction

entry^a^	cat.	base	time (h)	dr^b^	yield^c^ (%)	ee^d^ (%)
1	**C1**	K_2_CO_3_	24	17/1	58	99
2	**C2**	K_2_CO_3_	24	3/1	39	92
3	**C3**	K_2_CO_3_	24	3/1	58	91
4	**C1**	Na_2_CO_3_	48	20/1	55	99
5	**C1**	NaHCO_3_	168	20/1	32	99
6	**C1**	DIPEA	24	2/1	49	98
7^e^	**C1**	K_2_CO_3_	2	3/1	38	91
8^f^	**C1**	K_2_CO_3_	3	8/1	23	99
9^g^	**C1**	K_2_CO_3_	24	>20/1	59	99
10^h^	**C1**	K_2_CO_3_	18	>20/1	57	99
11^i^	**C1**	K_2_CO_3_	45	>20/1	64	99

a Reactions were conducted with **1a** (0.1 mmol), **2a** (0.2 mmol), corresponding base (0.2 mmol), and catalyst (20 mol %) in DCM (1.0 mL) at room temperature. After the full disappearance of methyleneindolinone **1a** (monitored by TLC), the reaction mixture was concentrated using rotavap. Crude product was purified using column chromatography.

b Determined by ^1^H NMR of the crude reaction mixture (**3a**/**4a**).

c Isolated yield of **3a** after column chromatography.

d Determined by chiral HPLC analysis.

e EtOAc was used.

f MTBE was used.

g CHCl_3_ was used.

h Reaction was conducted with **1a** (0.10 mmol), **3a** (0.15 mmol), and **C1** (20 mol %) in CHCl_3_ (1.0 mL) at room temperature.

i Reaction was conducted with **1a** (0.10 mmol), **3a** (0.15 mmol), and **C1** (1 mol %) in CHCl_3_ (1.0 mL) at room temperature.

**Scheme 2:**
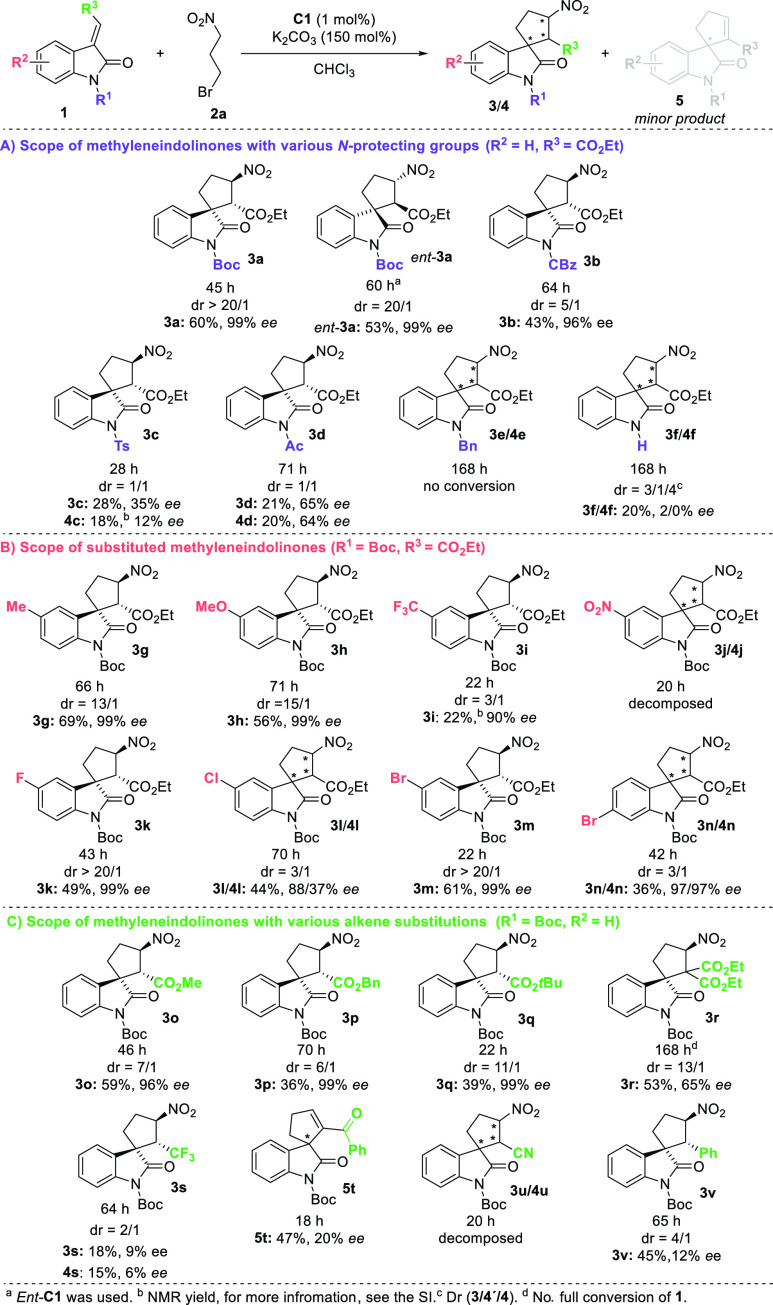
Substrate Scope of Organocascade Reactions

After optimizing the reaction conditions, we began exploring the scope of the organocascade reaction by varying *N*-protecting groups of methyleneindolinones **1** (Scheme 2A). We assessed the effect on reactivity and stereoselectivity of organocascade reactions using various *N*-protected methyleneindolinones. We identified oxycarbonyl-protecting groups as most effective in terms of stereocontrol. Corresponding spirocyclic compounds **3a**,**b** were isolated in good yields (43–60%) with high stereoselectivity.

On the other hand, organocascade reactions of other *N*-protected methyleneindolinones did not give products with acceptable yields and stereochemical outcomes. For example, the organocascade reaction of unprotected methyleneindolinone **1f** produced a mixture of products (**3f**/**4f**) with poor stereocontrol. Luckily, substrate **3f** can be prepared in high yield by TFA-mediated deprotection of the *N*-Boc protecting group of **3a** (for more details, please see late-stage transformations). Subsequently, the scope of the developed organocascade reaction was investigated by varying substituted methyleneindolinones **1** (Scheme 2B). In general, spirooxindoles **3** were obtained in moderate-to-good yields with excellent stereoselectivity, when oxindole derivatives **1** bearing electron-donating (**3g**, **3h**) and weakly electron-withdrawing groups (**3k**–**n**) on the oxindole aromatic ring were used. The reaction of methyleneindolinones bearing a strong electron-withdrawing group, such as the nitro group, led to a complex mixture or to the decomposition of starting material. Additionally, we studied the process using various substituted alkenes of methyleneindolinone derivatives **1**. Good efficiency of the developed method was shown in reactions of alkenes bearing various electron-withdrawing groups, especially in reactions of ester-derived alkenes producing spirocycles **3o**–**r** in moderate-to-good yields (36–59%) and excellent stereochemical outcomes (Scheme 2C). Remarkably, other electron-withdrawing groups did not show similar efficiency. For example, the reaction between ketone-derived alkene **1t** and **2a** produced only elimination product **5t** in moderate yield and low enantioselectivity.

**Figure 2:**
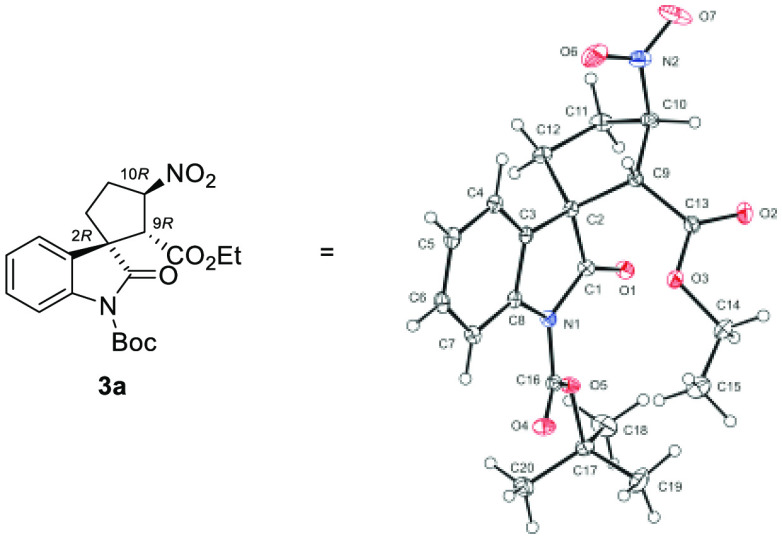
X-ray single-crystal structure of **3a** and the displacement ellipsoids at 30% probability level.

The relative configuration of spirooxindole-fused cyclopentanes **3** was adopted on the basis of 1D NOE NMR spectroscopy of mixtures **3l**/**4l** and **3n**/**4n** (for details, please see the SI). In addition, the absolute configuration of **3a** was ascertained using X-ray diffraction analysis, and the configuration of **3a** was assigned as 2*R*, 9*R*, and 10*R* (Figure 2, for details, see the SI). Absolute configurations of other spirooxindole derivatives **3** were assigned by the analogy of chemical shifts and *J* values of the cyclopentane ring.

**Figure 3:**
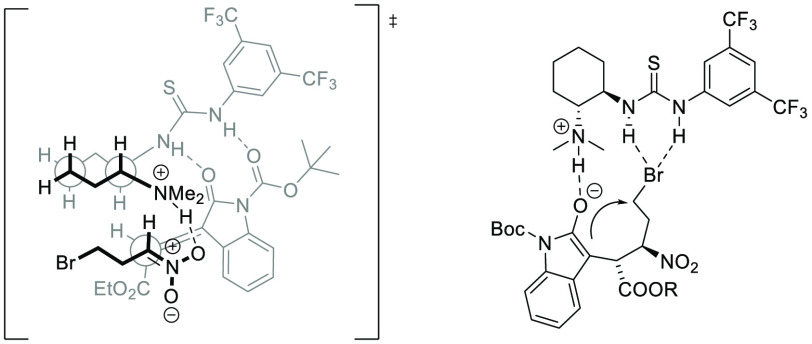
Proposed bifunctional activation and subsequent spirocyclization.

On the basis of the absolute configuration of products and the previous report,^16^ the transition state was proposed to rationalize the stereochemical outcome of the cascade process (Figure 3). The tertiary amine moiety of catalyst **C1** deprotonates an acidic proton of nitroalkane **2a**, generating the complex of nitronate noncovalently bonded to the tertiary amine. Simultaneously, the thiourea part of the catalyst activates methyleneindolinone **1a**, prompting *Si*-face addition of nitronate to the electron-deficient alkene. As a result, the corresponding Michael adduct with 9*R* and 10*R* configuration is formed. Subsequently, the intramolecular α-alkylation proceeds with good diastereocontrol, forming spirocycle **3** with 2*R* configuration at the spiro atom. The observed diastereocontrol can be explained by kinetically favored spirocyclization in the presence of bifunctional organocatalyst **C1**,^11c^ which can participate in the spirocyclization step by H-bonding to a bromide anion. Noteworthy, the sterical hindrance of the *tert-*butyl moiety of the *N-*Boc group may increase the rigidity of the initially formed ternary complex, which seems crucial for high stereocontrol. That hypothesis is supported by lowered diastereocontrol, when methyleneindolinone **3b** with a more planar *N*-CBz protecting group is used.

**Scheme 3:**
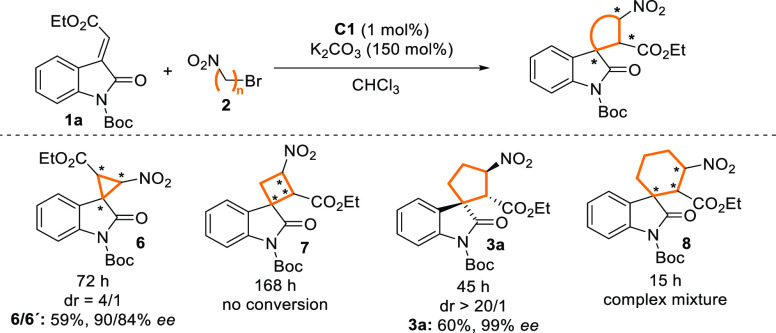
Substrate Scope with Diverse Bromonitroalkanes

To expand the developed organocatalytic process toward the construction of spiro compounds containing 3-, 4-, and 6-membered rings (Scheme 3), 1-bromonitroalkanes **2** with various lengths of alkyl moiety were subjected to the reaction with methyleneindolinone **1a**. With respect to previously reported methods,^17^ we isolated the corresponding spirooxindole-fused cyclopropane **6** in good yield and stereochemical outcomes. Despite known examples of spirooxindole-fused cyclobutanes,^11b^ we did not observe any conversion of starting methyleneindolinone **1a** in reaction with 1-bromo-2-nitroethane (**2c**). Interestingly, reaction of longer 1-bromo-2-nitrobutane produced an unseparable complex mixture of products with major uncyclized products of the Michael reaction.

**Scheme 4:**
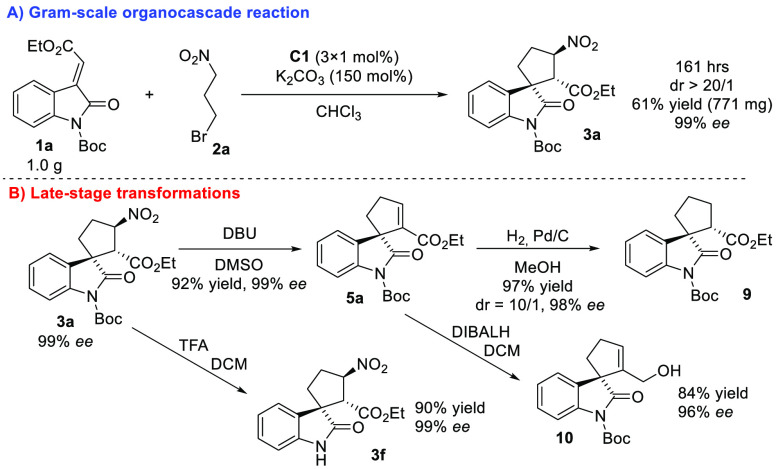
Gram-Scale Organocascade Reaction and Late-Stage Transformations

To demonstrate the synthetic utility of the developed organocascade reaction, we performed a reaction between **1a** and **2a** in gram scale, giving the product **3a** in 61% yield with retained stereochemical outcomes (99% ee and dr > 20/1, Scheme 4A). To reduce reaction time, the reaction was performed with a slightly higher amount of **C1** (3 mol %). This observation can be explained by the limited stability of **C1** in the presence of an excess of 3-bromo-1-nitropropane (**2a**) and base (for more information, please see the SI). As an example of late-stage transformations, spirooxindole **3a** was selectively converted to various derivatives (Scheme 4B). The *N*-Boc-protecting group was removed by treatment of **3a** with an excess of TFA. The reaction provided the corresponding spirooxindole **3f** in excellent yield with retained enantioselectivity. Noteworthy, the sequence of the developed organocascade followed by *N*-deprotection is more appropriate compared to the direct organocascade reaction starting from **1f**. Next, DBU-mediated elimination of HNO_2_ produced alkene **5a** in excellent yield with retained optical purity. Noteworthy, the double bond of alkene **5a** can be selectively reduced under catalytic hydrogenation conditions, producing cyclopentane derivative **9** with high diastereocontrol. The relative configuration of **9** was determined by 1D NOE NMR experiments (for more information, please see the SI). Furthermore, ethyl ester **5a** can be chemoselectively reduced to the corresponding allylic alcohol **9** by treatment with DIBALH. Spirocyclic allylic alcohol **10** may be used as a valuable building block for synthesizing valuable complex molecules.^18^

## Conclusion

In summary, we have developed an enantioselective organocascade Michael/spirocyclization reaction of readily available methyleneindolinone with 1-bromo-3-nitropropane. The reaction is efficiently catalyzed by a chiral bifunctional catalyst, affording chiral spirooxindole-fused cyclopentanes in moderate-to-good yields and excellent stereochemical outcomes. The developed synthetic protocol is suitable for late-stage functionalizations, as shown by a set of additional transformations.

## Experimental Section

Chemicals and solvents were purchased from commercial suppliers and purified using standard techniques. For thin-layer chromatography (TLC), silica gel plates from Merck 60 F_254_ were used, and compounds were visualized by irradiation with UV light and/or by treatment with a solution of phosphomolybdic acid (AMC) or vanillin followed by heating. Column chromatography was performed using silica gel Fluka (40–63 μm) or SiliCycle-SiliaFlash P60 (particle size: 40–63 μm, pore diameter: 60 Å). ^1^H, ^13^C, and ^19^F NMR spectra were recorded with Bruker AVANCE III 400. Chemical shifts for protons are given in δ relative to tetramethylsilane (TMS), and they are referenced to residual protium in the NMR solvent (chloroform-*d*: δ_H_ = 7.26 ppm). Splitting patterns are stated as singlet (s), doublet (d), triplet (t), quartet (q), doublet of doublet (dd), doublet doublet of doublet (ddd), doublet of triplet (dt), doublet triplet of doublet (dtd), doublet of quartet (dq), triplet of doublet (td), quartet of doublet (qd), m (multiplet), and broad singlet (br s). Splitting patterns that could not be easily interpreted were marked as multiplets. Chemical shifts for carbon are referenced to the carbon of NMR solvent (chloroform-*d*: δ_C_ = 77.16 ppm). The coupling constants *J* are given in hertz. IR DRIFT or ATR spectra were recorded with Nicolet AVATAR 370 FT-IR in cm^–1^. Chiral HPLC was carried out using a LC20AD Shimadzu liquid chromatograph with SPD-M20A diode array detector with columns Daicel Chiralpak IA, Daicel Chiralpak IB, Daicel Chiralpak AD, and Daicel Chiralpak ODH. Samples for measurement of chiral HPLC were prepared by dissolving the corresponding sample in an *n-*heptane/*i*-PrOH (8/2, *v*/*v*) mixture. Optical rotations were measured on an AU-Tomatica polarimeter, and Autopol III and specific optical rotation are given in concentrations *c* [g/100 mL]. Samples for the measurement of specific optical rotation were prepared by dissolving the corresponding sample in chloroform in concentrations which are labeled for each compound. Melting points were measured using a Büchi melting point B-545 apparatus. All melting points were measured in an open glass capillary, and all values are uncorrected. High-resolution mass spectra were recorded with an LCQ Fleet spectrometer. The measurement of low-resolution mass spectra was performed on a GCMS-QP2010 Shimadzu spectrometer. Samples for mass spectrometry were prepared by dissolving the corresponding sample in methanol.

### Preparation of Catalyst

Catalyst **C1** was purchased from commercial suppliers. *Ent*-**C1**, **C2**, and **C3** are known and prepared according to previously reported procedures.^19^

### Preparation of Methyleneindolinones

Methyleneindolinones **1** are typically known (**1i** is a new compound), and they were prepared according to previously reported procedures.^20^

#### *tert*-Butyl (*E*)-3-(2-Ethoxy-2-oxoethylidene)-2-oxo-5-(trifluoromethyl)indoline-1-carboxylate (**1i**)

Ethyl 2-(triphenyl-λ^5^-phosphanylidene)acetate (142 mg, 0.41 mmol, 1.1 equiv) was added in one portion to a stirred solution of 5-trifluoromethylisatin^21^ (80 mg, 0.37 mmol, 1.0 equiv) in THF (1 mL). The resulting mixture was stirred at room temperature for 3 h. After the consumption of starting isatin (monitored by TLC), solvent was removed under reduced pressure. Crude product was purified by column chromatography (eluting with hexane/EtOAc = 3/1–1/1). The resulting heterocyclic alkene (quantitative yield) was used in the next step without other purification. Heterocyclic alkene (116 mg, 0.41 mmol, 1.0 equiv) and di-*tert*-butyldicarbonate (98 mg, 0.45 mmol, 1.1 equiv) were added in one portion to a stirred solution of DMAP (3 mg, 0.02 mmol, 0.05 equiv) in THF (2 mL) at room temperature. The resulting mixture was stirred at room temperature for 14 h. After the consumption of starting alkene (monitored by TLC), the reaction was quenched by adding water (5 mL) and diluted with EtOAc (5 mL). The organic phase was separated, and the water phase was extracted with EtOAc (3 × 10 mL). Collected organic phases were washed with brine (1 × 10 mL) and dried over MgSO_4_. After filtration of drying agent, solvents were removed under reduced pressure. The crude product was purified by column chromatography with toluene as an eluent.

#### Yellow Amorphous Solid

Yield = 49% (70 mg, over two steps). ^1^H NMR (400 MHz, chloroform-*d*): δ 9.04–8.97 (m, 1H), 8.04 (dt, *J* = 8.7, 0.7 Hz, 1H), 7.69 (ddd, *J* = 8.6, 2.0, 0.8 Hz, 1H), 6.98 (s, 1H), 4.36 (q, *J* = 7.1 Hz, 2H), 1.65 (s, 9H), 1.38 (t, *J* = 7.1 Hz, 3H) ppm. ^13^C{^1^H} NMR (101 MHz, chloroform-*d*): δ 165.2, 165.1, 148.6, 144.3, 135.2, 129.6 (q, *J* = 3.8 Hz, 1C), 127.0 (q, *J* = 33.0 Hz, 1C), 125.7 (q, *J* = 4.0 Hz, 1C), 125.4, 124.0 (q, *J* = 272.1 Hz), 120.4, 115.2, 85.6, 61.9, 28.1 (3C), 14.2 ppm. ^19^F NMR (376 MHz, chloroform-*d*): δ −62.27 (d, *J* = 0.9 Hz) ppm. IR (KBr): ν = 1765 (C=O, ester, amide), 1738 (C=O, ester, amide), 1711 (C=O, ester, amide), 1201 (C–CF_3_) cm^–1^. HRMS (ESI+) *m*/*z*: calcd. for C_18_H_18_F_3_NNaO_5_ [M + Na]^+^: 408.1029, found: 408,1027.

### Preparation of 1-Bromonitrolalkanes

Alkane **2b** was purchased from commercial suppliers. **2d** is known and was prepared according to a previously reported procedure.^22^

### General Procedure for the Appel Reaction (GP1)

NBS (1.3 equiv) and PPh_3_ (1.3 equiv) were added portionwise to a stirred solution of nitroalcohol (1.00 g, 9.51 mmol, 1.0 equiv) in DCM (0.3 M solution of alcohol) at room temperature. The reaction mixture was stirred at room temperature for 1 h. After the full consumption of starting 3-nitropropan-1-ol (monitored by TLC), solvent was removed under reduced pressure. The crude product was purified by column chromatography (eluting with hexane/EtOAc = 7/1).

#### 1-Bromo-3-nitropropane (**2a**)

The title compound was synthesized according to general procedure GP1, using 3-nitropropan-1-ol^23^ (1.00 g, 9.51 mmol).

#### Yellow Oil

Yield = 50% (780 mg). ^1^H NMR (400 MHz, chloroform-*d*): δ 4.59 (t, *J* = 6.5 Hz, 2H), 3.50 (t, *J* = 6.2 Hz, 2H), 2.54 (p, *J* = 6.4 Hz, 2H) ppm. ^13^C{^1^H} NMR (101 MHz, chloroform-*d*): δ 73.3, 29.9, 28.7 ppm. GCMS (EI, 70 eV): *t*_R_ = 8.1 min. *m*/*z* (%): 89 (1), 72 (1), 57 (2), 42 (4), 41 (100), 39 (65), 27 (8). Our physical and spectroscopic data matched previously reported data.^24^

#### 1-Bromo-2-nitroethane (**2c**)

The title compound was synthesized according to general procedure GP1, starting from 2-nitroethan-1-ol^25^ (1030 mg, 11.3 mmol).

Light yellow liquid. Yield = 92% (1600 mg). ^1^H NMR (400 MHz, chloroform-*d*): δ 4.77 (t, *J* = 6.4 Hz, 2H), 3.81 (t, *J* = 6.3 Hz, 2H) ppm. ^13^C{^1^H} NMR (101 MHz, chloroform-*d*): δ 75.7, 23.8 ppm. GCMS (EI, 70 eV): *t*_R_ = 10.1 min. *m*/*z* (%): 89 (17), 75 (100), 59 (16), 47 (21), 31 (18). Our physical and spectroscopic data matched previously reported data.^24^

### General Procedure for the Michael/Alkylation Cascade Reaction (GP2)

The catalyst **C1** (0.4 mg, 0.001 mmol, 0.01 equiv) was added to a solution of the corresponding methyleneindolinone **1** (0.1 mmol, 1.0 equiv) in anhydrous chloroform (0.5 mL) at room temperature. Then, 1-bromonitroalkane **2** (0.15 mmol, 1.5 equiv) and potassium carbonate (20.7 mg, 0.15 mmol, 1.5 equiv) were added. The reaction was stirred at room temperature for the indicated time (TLC monitoring). With complete conversion of methyleneindolinone **1**, solvent was removed under reduced pressure. Crude product was purified by column chromatography.

*Note*: For racemic reactions, catalyst *rac*-**C1** was used.

#### 1′-(*tert*-Butyl) 2-Ethyl (1*R*,2*R*,3*R*)-3-Nitro-2′-oxospiro[cyclopentane-1,3′-indoline]-1′,2-dicarboxylate (**3a**, *ent*-**3a**)

The title compound was synthesized according to the GP2, using methyleneindolinone **1a** (31.8 mg, 0.1 mmol) and 1-bromo-3-nitropropane (25.0 mg, 0.15 mmol) at a reaction time of 45 h. The products were purified by column chromatography (hexane/EtOAc = 10/1), with a diastereomeric ratio of **3a**/**4a** > 20/1.

*Ent*-**3a** was prepared according to the modified GP2, using catalyst *ent-*C1 instead of **C1** and the same starting materials and purification method as **3a** (reaction time: 60 h). The diastereomeric ratio of *ent***-3a**/*ent***-4a** = 20/1.

White crystalline solid, crystals suitable for X-ray analysis, were grown by the dissolution of **3a** (20 mg) in a boiling *i-*PrOH (0.5 mL), followed by standing at rt overnight. Yield **3a** = 60% (24 mg). Yield *ent*-**3a** = 53% (21 mg). mp (**3a**) = 78–80 °C (*i* -PrOH). 99% *ee* for **3a** and 99% *ee* for *ent-***3a**, and the enantiomeric excess of **3a** was determined by HPLC (IA, *n*-heptane/*i*-PrOH = 80/20, flow rate = 1.0 mL/min, λ = 208 nm) at *t*_R_ = 4.4 min (minor) and 5.0 min (major). The enantiomeric excess of *ent-***3a** was determined by HPLC (IA, *n*-heptane/*i*-PrOH = 80/20, flow rate = 1.0 mL/min, λ = 216 nm) at *t*_R_ = 4.4 min (major) and 5.0 min (minor). [α]_D_^20^ (**3a**) = +9.2 (*c* = 0.4, CHCl_3_). [α]_D_^20^ (*ent-***3a**) = −6.0 (*c* = 1.4, CHCl_3_). ^1^H NMR (400 MHz, chloroform-*d*): δ 7.88–7.81 (m, 1H), 7.42–7.29 (m, 2H), 7.28–7.20 (m, 1H), 5.69 (ddd, *J* = 9.5, 6.8, 3.6 Hz, 1H), 4.20 (d, *J* = 6.8 Hz, 1H), 4.09 (dq, *J* = 10.7, 7.1 Hz, 1H), 3.95 (dq, *J* = 10.8, 7.1 Hz, 1H), 3.08–2.89 (m, 1H), 2.47–2.21 (m, 3H), 1.64 (s, 9H), 1.06 (t, *J* = 7.1 Hz, 3H) ppm. ^13^C{^1^H} NMR (101 MHz, chloroform-*d*): δ 177.5, 168.5, 149.1, 139.8, 129.7, 129.1, 125.1, 122.0, 115.3, 87.6, 84.8, 62.0, 57.9, 56.7, 37.6, 31.2, 28.2 (3C), 13.7 ppm. IR (KBr): ν =1759 (C=O, ester, amide), 1739 (C=O, ester, amide), 1549 (NO_2_), 1350 (NO_2_) cm^–1^. HRMS (ESI+) *m*/*z*: calcd. for C_20_H_24_N_2_NaO_7_ [M + Na]^+^: 427.1476, found: 427.1474.

#### Ethyl (1*R*,2*R*,3*R*)-3-Nitro-2′-oxo-1′-(2-oxo-2-phenyl-1λ^2^-ethyl)spiro[cyclopentane-1,3′-indoline]-2-carboxylate (**3b**)

The title compound was synthesized according to the GP2, using methyleneindolinone **1b** (33.3 mg, 0.1 mmol) and 1-bromo-3-nitropropane (25.0 mg, 0.15 mmol) at a reaction time of 64 h. The product was purified by column chromatography (hexane/EtOAc = 3/1). The diastereomeric ratio of **3b**/**4b** = 5/1.

Yellow oil. Yield = 43% (18 mg). 96% ee. The enantiomeric excess of product **3b** was determined by HPLC (IB, *n*-heptane/*i*-PrOH = 80/20, flow rate = 1.0 mL/min, λ = 208 nm) at *t*_R_ = 11.1 min (minor) and 12.9 min (major). [α]_D_^20^ = +3.6 (*c* = 1.0, CHCl_3_). ^1^H NMR (400 MHz, chloroform-*d*): δ 7.97–7.87 (m, 1H), 7.55–7.47 (m, 2H), 7.46–7.31 (m, 5H), 7.29–7.23 (m, 1H), 5.68 (ddd, *J* = 9.6, 6.9, 3.6 Hz, 1H), 5.51–5.39 (m, 2H), 4.22 (d, *J* = 6.9 Hz, 1H), 4.07–3.88 (m, 2H), 3.07–2.93 (m, 1H), 2.47–2.36 (m, 1H), 2.36–2.22 (m, 2H), 0.97 (t, *J* = 7.1 Hz, 3H) ppm. ^13^C{^1^H} NMR (101 MHz, chloroform-*d*): δ 177.3, 168.4, 150.7, 139.4, 134.9, 129.6, 129.3, 128.84 (2C), 128.76, 128.5 (2C), 125.5, 122.1, 115.5, 87.6, 68.9, 62.2, 58.1, 56.7, 37.6, 31.2, 13.6 ppm. IR (KBr): ν 1765 (C=O, ester, amide), 1741 (C=O, ester, amide), 1724 (C=O, ester, amide), 1556 (NO_2_), 1377 (NO_2_) cm^–1^. HRMS (ESI+) *m*/*z*: calcd. for C_23_H_22_N_2_NaO_7_ [M + Na + H_2_O]^+^: 461.1319, found: 461.1327.

#### Ethyl (1*R*,2*R*,3*R*)-3-Nitro-2′-oxo-1′-tosylspiro[cyclopentane-1,3′-indoline]-2-carboxylate (**3c**/**4c**)

The title compounds were synthesized according to the GP2, using methyleneindolinone **1c** (37.1 mg, 0.1 mmol) and 1-bromo-3-nitropropane (25.0 mg, 0.15 mmol), at a reaction time of 28 h. The product was purified by column chromatography (hexane/EtOAc = 10/1). The diastereomeric ratio of **3c**/**4c** = 1/1. Products were obtained as an inseparable mixture of **4c**/**5c** and pure **3c**.

#### Ethyl (1*R*,2*R*,3*R*)-3-Nitro-2′-oxo-1′-tosylspiro[cyclopentane-1,3′-indoline]-2-carboxylate (**3c**)

Yellow oil. Yield = 28% (13 mg). 35% ee. The enantiomeric excess of product **3c** was determined by HPLC (IB, *n*-heptane/*i*-PrOH = 90/10, flow rate = 1.0 mL/min, λ = 207 nm) at *t*_R_ = 10.6 min (minor) and 11.9 min (major). [α]_D_^20^ = −2.3 (*c* = 0.7, CHCl_3_). ^1^H NMR (400 MHz, chloroform-*d*): δ 8.00–7.91 (m, 3H), 7.41–7.29 (m, 4H), 7.23 (dd, *J* = 7.5, 1.0 Hz, 1H), 5.58 (ddd, *J* = 9.7, 7.0, 3.5 Hz, 1H), 4.13 (d, *J* = 7.0 Hz, 1H), 3.85 (dq, *J* = 10.6, 7.1 Hz, 1H), 3.63 (dq, *J* = 10.6, 7.1 Hz, 1H), 2.95–2.84 (m, 1H), 2.42 (s, 3H), 2.40–2.03 (m, 3H), 0.91 (t, *J* = 7.1 Hz, 3H) ppm. ^13^C{^1^H} NMR (101 MHz, chloroform-*d*): δ 177.3, 168.0, 145.8, 139.4, 135.0, 129.8 (2C), 129.6, 129.4, 128.3 (2C), 125.5, 122.4, 113.9, 87.1, 62.0, 57.6, 56.7, 37.2, 30.9, 21.8, 13.7 ppm. IR (KBr): ν = 1738 (C=O, ester, amide), 1552 (NO_2_), 1371 (NO_2_), 1336 (S=O, sulfonamide) cm^–1^. HRMS (ESI+) *m*/*z*: calcd. for C_22_H_23_N_2_O_7_S [M + H]^+^: 459.1220, found 459.1220.

#### Ethyl-3-nitro-2′-oxo-1′-tosylspiro[cyclopentane-1,3′-indoline]-2-carboxylate (**4c**)

Inseparable mixture of **4c**/**5c** = 5/1. Yellow oil. NMR yield **4c** = 18%. 12/12% ee (**4c**/**5c**). The enantiomeric excess of product **4c** was determined by HPLC (IB, *n*-heptane/*i*-PrOH = 80/20, flow rate = 1.0 mL/min, λ = 207 nm) at *t*_R_ = 14.9 min (major) and 20.4 min (minor). The enantiomeric excess of product **5c** was determined by HPLC (IB, *n*-heptane/*i*-PrOH = 80/20, flow rate = 1.0 mL/min, λ = 207 nm) at *t*_R_ = 8.5 min (minor) and 9.0 min (major). ^1^H NMR (400 MHz, chloroform-*d*, **4c** – H′, **5c** – H): δ 8.03 (dd, *J* = 8.6, 2.0 Hz, 2H′), 8.01 (d, *J* = 6.6 Hz, 2H), 7.95 (dt, *J* = 8.2, 0.7 Hz, 1H′), 7.91 (dt, *J* = 8.2, 0.8 Hz, 1H), 7.38 (d, *J* = 1.3 Hz, 1H), 7.35 (dd, *J* = 8.2, 1.2 Hz, 3H′), 7.33–7.28 (m, 2H), 7.18–7.11 (m, 1H + 1H′, *overlapped*), 7.11–7.08 (m, 1H), 7.02 (dd, *J* = 7.5, 1.5 Hz, 1H), 6.99 (dd, *J* = 7.5, 1.4 Hz, 1H′), 5.58 (td, *J* = 8.8, 5.9 Hz, 1H′), 4.22 (d, *J* = 8.3 Hz, 1H′), 3.84 (dq, *J* = 10.8, 7.1 Hz, 1H), 3.64–3.55 (m, 1H + 1H′, *overlapped*), 3.44–3.32 (m, 1H + 1H′, *overlapped*), 2.78 (dtd, *J* = 8.3, 5.9, 2.6 Hz, 1H), 2.74–2.65 (m, 1H′), 2.65–2.54 (m, 1H′ + 1H, *overlapped*), 2.53–2.45 (m, 1H′), 2.43 (s, 3H′), 2.40 (s, 3H), 2.22–2.13 (m, 1H), 1.95 (ddd, *J* = 13.2, 7.4, 4.7 Hz, 1H′), 0.83 (t, *J* = 7.1 Hz, 3H′), 0.43 (t, *J* = 7.1 Hz, 3H′) ppm. ^13^C{^1^H} NMR (101 MHz, chloroform-*d*, **4c** – C′, **5c** – C): δ 177.5 (1C), 176.3 (1C′), 167.8 (1C′), 162.4 (1C), 148.7 (1C′), 146.1 (1C′), 145.5 (1C), 139.0 (1C), 138.8 (1C′), 137.4 (1C), 135.5 (1C), 135.2 (1C′), 132.4 (1C), 129.9 (2C′ + 2C, *overlapped*), 129.8 (1C′), 129.7 (1C′), 129.2 (1C), 128.9 (1C), 128.2 (2C′ + 2C, *overlapped*), 125.2 (1C′), 125.0 (1C), 123.2 (1C′), 122.9 (1C), 113.9 (1C′), 113.5 (1C), 85.6 (1C′), 61.6 (1C′), 60.5 (1C), 57.0 (1C′), 56.4 (1C), 38.1 (1C), 37.3 (1C′), 32.2 (1C), 30.7 (1C′), 21.9 (1C′), 21.8 (1C), 13.8 (1C), 13.2 (1C′) ppm. IR (KBr): ν = 1761 (C=O, ester, amide), 1736 (C=O, ester, amide), 1550 (NO_2_), 1373 (S=O, sulfonamide), 1317 (NO_2_) cm^–1^. HRMS (**4c**, ESI+) *m*/*z*: calcd. for C_22_H_23_N_2_O_7_S [M + H]^+^: 459.1220, found: 459.1216.

#### Ethyl 1′-Acetyl-3-nitro-2′-oxospiro[cyclopentane-1,3′-indoline]-2-carboxylate (**3d**/**4d**)

The title compounds were synthesized according to the GP2, using methyleneindolinone **1d** (25.9 mg, 0.1 mmol) and 1-bromo-3-nitropropane (25.0 mg, 0.15 mmol) at a reaction time of 71 h. The products were purified by column chromatography (hexane/EtOAc = 7/1), and the diastereomeric ratio of **3d**/**4d** = 1/1.

#### Ethyl (1*R*,2*R*,3*R*)-1′-Acetyl-3-nitro-2′-oxospiro[cyclopentane-1,3′-indoline]-2-carboxylate (**3d**)

Yellow oil. Yield = 21% (7 mg). 65% ee. The enantiomeric excess of product **3d** was determined by HPLC (IB, *n*-heptane/*i*-PrOH = 98/2, flow rate = 1.0 mL/min, λ = 224 nm) at *t*_R_ = 17.2 min (minor) and 38.1 min (major). [α]_D_^20^ = +6.8 (*c* = 0.4, CHCl_3_). ^1^H NMR (400 MHz, chloroform-*d*): δ 8.24 (dt, *J* = 8.1, 0.8 Hz, 1H), 7.43–7.35 (m, 2H), 7.32–7.26 (m, 1H), 5.66 (ddd, *J* = 9.5, 7.1, 3.8 Hz, 1H), 4.23 (d, *J* = 7.1 Hz, 1H), 4.11–3.91 (m, 2H), 3.05–2.83 (m, 1H), 2.64 (s, 3H), 2.51–2.18 (m, 3H), 1.06 (t, *J* = 7.1 Hz, 3H) ppm. ^13^C{^1^H} NMR (101 MHz, chloroform-*d*): δ 180.0, 170.7, 168.5, 140.2, 129.4, 128.7, 125.9, 121.8, 116.9, 87.5, 62.2, 58.3, 56.7, 37.5, 31.2, 26.7, 13.8 ppm. IR (KBr): ν = 1739 (C=O, ester, amide), 1705 (C=O, ester, amide), 1552 (NO_2_), 1273 (NO_2_) cm^–1^. HRMS (ESI+) *m*/*z*: calcd. for C_17_H_18_N_2_NaO_6_ [M + Na]^+^: 369.1057, found: 369.1052.

#### Ethyl 1′-Acetyl-3-nitro-2′-oxospiro[cyclopentane-1,3′-indoline]-2-carboxylate (**4d**)

Yellow oil. Yield = 20% (7 mg). 64% ee. The enantiomeric excess of product **4d** was determined by HPLC (IB, *n*-heptane/*i*-PrOH = 98/2, flow rate = 1.0 mL/min, λ = 216 nm) at *t*_R_ = 23.8 min (major) and 49.2 min (minor). [α]_D_^20^ = +4.4 (*c* = 0.3, CHCl_3_). ^1^H NMR (400 MHz, chloroform-*d*): δ 8.27 (dt, *J* = 8.1, 0.9 Hz, 1H), 7.36 (ddd, *J* = 8.2, 7.6, 1.4 Hz, 1H), 7.19 (td, *J* = 7.6, 1.1 Hz, 1H), 7.07 (ddd, *J* = 7.6, 1.4, 0.6 Hz, 1H), 5.66 (ddd, *J* = 9.5, 8.4, 5.7 Hz, 1H), 4.32 (d, *J* = 8.4 Hz, 1H), 3.73 (qd, *J* = 7.2, 1.7 Hz, 2H), 2.88–2.71 (m, 1H), 2.74 (s, 3H), 2.73–2.53 (m, 2H), 2.06 (ddd, *J* = 13.0, 7.6, 4.3 Hz, 1H), 0.70 (t, *J* = 7.1 Hz, 3H) ppm. ^13^C{^1^H} NMR (101 MHz, chloroform-*d*): δ 178.8, 170.9, 167.9, 139.8, 129.6, 129.3, 125.6, 122.6, 117.0, 85.7, 61.8, 57.8, 56.8, 37.2, 30.8, 26.8, 13.5 ppm. IR (KBr): ν = 1759 (C=O, ester, amide), 1738 (C=O, ester, amide), 1699 (C=O, ester, amide), 1554 (NO_2_), 1309 (NO_2_) cm^–1^. HRMS (ESI+) *m*/*z*: calcd. for C_17_H_19_N_2_O_6_ [M + H]^+^: 347.1243, found: 347.1240.

#### Ethyl 3-Nitro-2′-oxospiro[cyclopentane-1,3′-indoline]-2-carboxylate (**3f**/**4f́/4f**)

The title compounds were synthesized according to the GP2, using methyleneindolinone **1f** (21.7 mg, 0.1 mmol) and 1-bromo-3-nitropropane (25.0 mg, 0.15 mmol) at a reaction time of 168 h. The products were purified by column chromatography (hexane/EtOAc = 3/1). The diastereomeric ratio of **3f**/**4f′/4f** = 3/1/4. Products were obtained as an inseparable mixture of diastereomers **3f/4f** and diastereomer **4f′**as an inseparable mixture with byproducts.

Inseparable mixture of diastereomers (**3f/4f**) = 1/1. Yellow oil. Combined yield (**3f/4f**) = 20% (8 mg). 2/0% ee (**3f/4f**). The enantiomeric excess of product **3f** was determined by HPLC (IC, *n*-heptane/*i*-PrOH = 90/10, flow rate = 1.0 mL/min, λ = 208 nm) at *t*_R_ = 9.6 min (minor) and 12.6 min (major). The enantiomeric excess of product **4f** was determined by HPLC (IC, *n*-heptane/*i*-PrOH = 90/10, flow rate = 1.0 mL/min, λ = 208 nm) at *t*_R_ = 13.6 min and 28.1 min. ^1^H NMR (400 MHz, chloroform-*d*, **4f** – H′, **3f** – H): δ 8.35 (br s, 1H′), 7.99 (br s, 1H), 7.35 (dd, *J* = 7.5, 1.2 Hz, 1H), 7.29–7.21 (m, 1H′ + 1H, *overlapped*), 7.11 (td, *J* = 7.6, 1.0 Hz, 1H), 7.06–6.98 (m, 2H′), 6.94–6.89 (m, 1H′ + 1H, *overlapped*), 5.76–5.63 (m, 1H′ + 1H, *overlapped*), 4.27 (d, *J* = 8.1 Hz, 1H′), 4.19 (d, *J* = 7.2 Hz, 1H), 4.10–3.95 (m, 2H), 3.83 (dq, *J* = 10.8, 7.1 Hz, 1H′), 3.74 (dq, *J* = 10.7, 7.1 Hz, 1H′), 3.06–2.94 (m, 1H), 2.81–2.62 (m, 2H′), 2.53 (dt, *J* = 13.1, 9.0 Hz, 1H′), 2.47–2.16 (m, 3H), 2.00 (ddd, *J* = 13.1, 7.3, 4.8 Hz, 1H′), 1.08 (t, *J* = 7.1 Hz, 3H), 0.72 (t, *J* = 7.1 Hz, 3H′) ppm. ^13^C{^1^H} NMR (101 MHz, chloroform-*d*, **4f** – C′, **3f** – C): δ 180.5 (1C), 179.4 (1C′), 168.7 (1C), 168.5 (1C′), 140.8 (1C), 140.6 (1C′), 131.2 (1C), 130.6 (1C′), 129.2 (1C′), 128.9 (1C), 123.5 (1C′), 123.2 (1C), 123.0 (1C′), 122.6 (1C), 110.2 (1C′), 110.0 (1C), 87.4 (1C), 86.1 (1C′), 61.9 (1C), 61.6 (1C′), 56.8 (1C), 56.5 (1C′), 36.5 (1C′ + 1C, *overlapped*), 30.9 (1C), 30.8 (1C′), 13.8 (1C), 13.5 (1C′) ppm. One *q*C′ and one *q*C were not found. IR (KBr): ν = 3192 (N–H), 1734 (C=O, ester, amide), 1707 (C=O, ester, amide), 1552 (NO_2_), 1342 (NO_2_) cm^–1^. HRMS (ESI+) *m*/*z*: calcd. for C_15_H_16_N_2_NaO_5_ [M + Na]^+^: 327.0951, found: 327.0948.

#### 1′-(*tert*-Butyl) 2-Ethyl (1*R*,2*R*,3*R*)-5′-Methyl-3-nitro-2′-oxospiro[cyclopentane-1,3′-indoline]-1′,2-dicarboxylate (**3g**)

The title compound was synthesized according to the GP2, using methyleneindolinone **1g** (33.1 mg, 0.1 mmol) and 1-bromo-3-nitropropane (25.0 mg, 0.15 mmol) at a reaction time of 66 h. The product was purified by column chromatography (hexane/EtOAc = 10/1). The diastereomeric ratio of **3g**/**4g** = 13/1.

Yellow oil. Yield = 69% (29 mg). 99% ee. The enantiomeric excess of product **3g** was determined by HPLC (IA, *n*-heptane/*i*-PrOH = 90/10, flow rate = 1.0 mL/min, λ = 204 nm) at *t*_R_ = 4.9 min (minor) and 6.0 min (major). [α]_D_^20^ = +19.9 (*c* = 0.7, CHCl_3_). ^1^H NMR (400 MHz, chloroform-*d*): δ 7.71 (d, *J* = 8.3 Hz, 1H), 7.19–7.09 (m, 2H), 5.68 (ddd, *J* = 9.4, 6.8, 3.6 Hz, 1H), 4.18 (d, *J* = 6.8 Hz, 1H), 4.10 (dq, *J* = 10.7, 7.1 Hz, 1H), 3.94 (dq, *J* = 10.7, 7.1 Hz, 1H), 3.09–2.89 (m, 1H), 2.45–2.19 (m, 6H), 1.63 (s, 9H), 1.06 (t, *J* = 7.1 Hz, 3H) ppm. ^13^C{^1^H} NMR (101 MHz, chloroform-*d*): δ 177.7, 168.5, 149.2, 137.4, 134.9, 129.61, 129.58, 122.6, 115.1, 87.6, 84.6, 62.0, 57.9, 56.7, 37.7, 31.2, 28.2 (3C), 21.3, 13.7 ppm. IR (KBr): ν 1786 (C=O, ester, amide), 1757 (C=O, ester, amide), 1732 (C=O, ester, amide), 1552 (NO_2_), 1369 (NO_2_) cm^–1^. HRMS (ESI+) *m*/*z*: calcd. for C_21_H_26_N_2_NaO_7_ [M + Na]^+^: 441.1632, found: 441.1626.

#### 1′-(*tert*-Butyl) 2-Ethyl (1*R*,2*R*,3*R*)-5′-Methoxy-3-nitro-2′-oxospiro[cyclopentane-1,3′-indoline]-1′,2-dicarboxylate (**3h**)

The title compound was synthesized according to the GP2, using methyleneindolinone **1h** (33.1 mg, 0.1 mmol) and 1-bromo-3-nitropropane (25.0 mg, 0.15 mmol) at a reaction timeof 71 h. The product was purified by column chromatography (hexane/EtOAc = 10/1). The diastereomeric ratio of **3h**/**4h** = 15/1.

Yellow oil. Yield = 56% (25 mg). 99% ee. The enantiomeric excess of product **3h** was determined by HPLC (IB, *n*-heptane/*i*-PrOH = 80/20, flow rate = 1.0 mL/min, λ = 207 nm) at *t*_R_ = 6.5 min (minor) and 8.6 min (major). [α]_D_^20^ = +20.4 (*c* = 1.1, CHCl_3_). ^1^H NMR (400 MHz, chloroform-*d*): δ 7.76 (d, *J* = 8.9 Hz, 1H), 6.92 (d, *J* = 2.6 Hz, 1H), 6.86 (dd, *J* = 8.8, 2.6 Hz, 1H), 5.69 (ddd, *J* = 10.1, 6.7, 3.7 Hz, 1H), 4.17 (d, *J* = 6.7 Hz, 1H), 4.10 (dq, *J* = 10.6, 7.1 Hz, 1H), 3.95 (dq, *J* = 10.5, 7.1 Hz, 1H), 3.84 (s, 3H), 2.99 (dq, *J* = 14.4, 9.4 Hz, 1H), 2.39 (ddt, *J* = 14.6, 7.5, 4.0 Hz, 1H), 2.33–2.21 (m, 2H), 1.62 (s, 9H), 1.07 (t, *J* = 7.1 Hz, 3H) ppm. ^13^C{^1^H} NMR (101 MHz, chloroform-*d*): δ 177.5, 168.4, 157.5, 149.2, 133.1, 130.9, 116.3, 113.7, 108.3, 87.6, 84.6, 62.0, 57.9, 57.0, 55.9, 37.7, 31.3, 28.2 (3C), 13.7 ppm. IR (KBr): ν 1784 (C=O, ester, amide), 1755 (C=O, ester, amide), 1728 (C=O, ester, amide), 1552 (NO_2_), 1369 (NO_2_) cm^–1^. HRMS (ESI+) *m*/*z*: calcd. for C_21_H_26_N_2_NaO_8_ [M + Na]^+^: 457.1581, found: 457.1584.

#### 1′-(*tert*-Butyl) 2-Ethyl 3-Nitro-2′-oxo-5′-(trifluoromethyl)spiro[cyclopentane-1,3′-indoline]-1′,2-dicarboxylate (**3i**/**4i**)

The title compounds were synthesized according to the GP2, using methyleneindolinone **1i** (38.5 mg, 0.1 mmol) and 1-bromo-3-nitropropane (25.0 mg, 0.15 mmol) at a reaction time of 22 h. The products were purified by column chromatography (hexane/EtOAc = 10/1) at a diastereomeric ratio of **3i**/**4i** = 3/1. Products were obtained as an inseparable mixture of diastereomers **3i**/**4i** with **5i**.

Inseparable mixture (**3i**/**4i**/**5i**) = 14/1/5. Yellow oil. NMR yield **3i** = 22%. 90% ee (**3i**). The enantiomeric excess of product **3i** was determined by HPLC (IC, *n*-heptane/*i*-PrOH = 98/2, flow rate = 1.0 mL/min, λ = 222 nm) at *t*_R_ = 17.6 min (minor) and 36.6 min (major). ^1^H NMR (400 MHz, chloroform-*d*, **3i** – H″, **4i** – H′, **5i** – H): δ 8.06 (d, *J* = 8.6 Hz, 1H′), 8.02–7.96 (m, 1H″ + 1H, *overlapped*), 7.65–7.62 (m, 1H + 1H′, *overlapped*), 7.62–7.59 (m, 1H″), 7.57 (ddd, *J* = 8.6, 2.0, 0.9 Hz, 1H), 7.30–7.28 (m, 1H′ + 1H, *overlapped*), 7.25 (q, *J* = 2.6 Hz, 1H), 5.73–5.66 (m, 1H′), 5.63 (dd, *J* = 8.7, 6.4 Hz, 1H′), 4.58 (t, *J* = 6.5 Hz, 1H′), 4.34 (d, *J* = 8.4 Hz, 1H′), 4.24 (d, *J* = 6.8 Hz, 1H″), 4.10 (dq, *J* = 10.8, 7.2 Hz, 1H″), 4.04–3.91 (m, 1H″ + 2H, *overlapped*), 3.77 (q, *J* = 7.1 Hz, 1H′), 3.11–2.94 (m, 1H″), 2.91–2.86 (m, 2H), 2.83–2.75 (m, 1H′), 2.76–2.66 (m, 1H + 1H′, *overlapped*), 2.48–2.37 (m, 1H″ + 1H′, *overlapped*), 2.37–2.23 (m, 2H′), 2.12–2.00 (m, 1H′), 1.67 (s, 9H′), 1.65 (s, 9H), 1.64 (s, 9H″), 1.08 (t, *J* = 7.1 Hz, 3H″), 1.04 (d, *J* = 7.1 Hz, 3H), 0.76 (t, *J* = 7.1 Hz, 3H′) ppm. ^13^C{^1^H} NMR (101 MHz, chloroform-*d*, **3i** – C″, **4i** – C′, **5i** – C): δ 177.1 (1C), 176.7 (1C″), 168.1 (1C′′), 167.7 (1C), 162.4 (1C), 148.9, (1C′′) 148.7 (1C), 142.7 (1C′′), 137.4 (1C′′), 133.0 (1C), 130.2 (1C′′), 127.3 (q, *J* = 33.1 Hz, 1C″), 126.6 (q, *J* = 3.8 Hz, 1C″), 126.0 (q, *J* = 3.8 Hz, 1C), 123.9 (q, *J* = 272.1 Hz, 1C″), 119.3 (q, *J* = 3.6 Hz, 1C), 119.0 (q, *J* = 3.8 Hz, 1C″), 115.4 (1C′′), 115.1 (1C), 87.2 (1C′′ + 1C, *overlapped*), 85.4 (1C″), 62.2 (1C′′), 60.8 (1C), 57.8 (1C′′), 56.4 (1C), 38.0 (1C), 37.3 (1C′′), 32.2 (1C), 31.0 (1C′′), 28.1 (3C′′ + 3C, *overlapped*), 13.7 (1C), 13.6 (1C″) ppm. Four *qC* were not found. ^19^F NMR (376 MHz, chloroform-*d*, **3i** – F″, **4i** – F′, **5i** – F): δ −61.83 (d, *J* = 0.8 Hz, 3F), −61.90 (d, *J* = 0.8 Hz, 3F″), −61.98 (s, 3F′) ppm. IR (KBr): ν = 1792 (C=O, ester, amide), 1766 (C=O, ester, amide), 1734 (C=O, ester, amide), 1556 (NO_2_), 1371 (NO_2_), 1120 (C–CF_3_) cm^–1^. HRMS (**3i**, ESI+) *m*/*z*: calcd. for C_21_H_23_F_3_N_2_NaO_7_ [M + Na]^+^: 495.1350, found: 495.1357. *Note:*^13^C{^1^H} NMR was determined for the mixture **3i/5i**.

#### 1′-(*tert*-Butyl) 2-Ethyl (1*R*,2*R*,3*R*)-5′-Fluoro-3-nitro-2′-oxospiro[cyclopentane-1,3′-indoline]-1′,2-dicarboxylate (**3k**)

The title compound was synthesized according to the GP2, using methyleneindolinone **1k** (33.5 mg, 0.1 mmol) and 1-bromo-3-nitropropane (25.0 mg, 0.15 mmol) at a reaction time of 43 h. The product was purified by column chromatography (hexane/EtOAc = 10/1), and the diastereomeric ratio of **3k**/**4k** > 20/1.

Yellow oil. Yield = 49% (21 mg). 99% ee. The enantiomeric excess of product **3k** was determined by HPLC (IB, *n*-heptane/*i*-PrOH = 80/20, flow rate = 1.0 mL/min, λ = 208 nm) at *t*_R_ = 5.8 min (minor) and 6.4 min (major). [α]_D_^20^ = +5.8 (*c* = 1.0, CHCl_3_). ^1^H NMR (400 MHz, chloroform-*d*): δ 7.84 (dd, *J* = 9.0, 4.5 Hz, 1H), 7.11 (dd, *J* = 7.6, 2.7 Hz, 1H), 7.04 (td, *J* = 8.9, 2.7 Hz, 1H), 5.68 (ddd, *J* = 9.5, 6.6, 3.5 Hz, 1H), 4.16 (d, *J* = 6.7 Hz, 1H), 4.10 (dq, *J* = 10.8, 7.2 Hz, 1H), 3.96 (dq, *J* = 10.7, 7.1 Hz, 1H), 3.07–2.91 (m, 1H), 2.47–2.35 (m, 1H), 2.32–2.22 (m, 2H), 1.63 (s, 9H), 1.07 (t, *J* = 7.1 Hz, 3H) ppm. ^13^C{^1^H} NMR (101 MHz, chloroform-*d*): δ 177.0, 168.2, 160.4 (d, *J* = 244.6 Hz, 1C), 149.1, 135.8 (d, *J* = 2.7 Hz, 1C), 131.5 (d, *J* = 7.9 Hz, 1C), 116.8 (d, *J* = 7.9 Hz, 1C), 115.7 (d, *J* = 22.7 Hz, 1C), 109.7 (d, *J* = 24.6 Hz, 1C), 87.5, 85.0, 62.2, 57.9, 56.9 (d, *J* = 1.9 Hz, 1C), 37.6, 31.3, 28.2 (3C), 13.7 ppm. ^31^F NMR (376 MHz, CDCl_3_): δ −116.68 (ddd, *J* = 9.0, 7.6, 4.6 Hz) ppm. IR (KBr): ν = 1788 (C=O, ester, amide), 1759 (C=O, ester, amide), 1732 (C=O, ester, amide), 1552 (NO_2_), 1369 (NO_2_), 1246 (C–F) cm^–1^. HRMS (ESI+) *m*/*z*: calcd. for C_20_H_23_FN_2_NaO_7_ [M + Na]^+^: 445.1382, found: 445.1380.

#### 1′-(*tert*-Butyl) 2-Ethyl 5′-Chloro-3-nitro-2′-oxospiro[cyclopentane-1,3′-indoline]-1′,2-dicarboxylate (**3l/4l**)

The title compounds were synthesized according to the GP2, using methyleneindolinone **1l** (35.2 mg, 0.1 mmol) and 1-bromo-3-nitropropane (25.0 mg, 0.15 mmol) at a reaction time of 70 h. The products were purified by column chromatography (hexane/EtOAc = 10/1). The diastereomeric ratio of **3l**/**4l** = 3/1.

Inseparable mixture of diastereomers **3l**/**4l** = 3/1. Yellow oil. Combined yield **3l**/**4l** = 44% (19 mg). 88/37% ee (**3l**/**4l**). The enantiomeric excess of product **3l** was determined by HPLC (IG, *n*-heptane/*i*-PrOH = 90/10, flow rate = 1.0 mL/min, λ = 209 nm) at *t*_R_ = 8.1 min (minor) and 11.7 min (major). The enantiomeric excess of product **4l** was determined by HPLC (IG, *n*-heptane/*i*-PrOH = 90/10, flow rate = 1.0 mL/min, λ = 209 nm) at *t*_R_ = 10.0 min (minor) and 10.8 min (major). ^1^H NMR (400 MHz, chloroform-*d*, **3l** – H′, **4l** – H): δ 7.88 (d, *J* = 8.7 Hz, 1H), 7.81 (d, *J* = 8.7 Hz, 1H′), 7.36 (d, *J* = 2.0 Hz, 1H′), 7.33–7.30 (m, 1H′ + 1H, *overlapped*), 7.01 (d, *J* = 2.2 Hz, 1H), 5.67 (ddd, *J* = 9.4, 6.7, 3.5 Hz, 1H′), 5.64–5.57 (m, 1H), 4.32 (d, *J* = 8.4 Hz, 1H), 4.18 (d, *J* = 6.7 Hz, 1H′), 4.10 (dq, *J* = 10.7, 7.1 Hz, 1H′), 3.97 (dq, *J* = 10.8, 7.1 Hz, 1H′), 3.80 (qd, *J* = 7.1, 4.6 Hz, 2H), 3.49–3.39 (m, 1H), 3.05–2.93 (m, 1H′), 2.88–2.62 (m, 1H), 2.57 (dt, *J* = 13.2, 8.9 Hz, 1H), 2.47–2.35 (m, 1H′), 2.35–2.23 (m, 2H′), 2.05–1.97 (m, 1H), 1.65 (s, 9H), 1.62 (s, 9H′), 1.07 (t, *J* = 7.1 Hz, 3H′), 0.80 (t, *J* = 7.1 Hz, 3H) ppm. ^13^C{^1^H} NMR (101 MHz, chloroform-*d*, **3l** – C′, **4l** – C): δ 176.8 (1C′), 175.7 (1C), 168.2 (1C′), 167.9 (1C), 148.9 (1C′ + 1C, *overlapped*), 138.4 (1C′), 138.2 (1C), 131.4 (1C′), 130.8 (1C), 130.6 (1C′), 130.3 (1C), 129.4 (1C), 129.2 (1C′), 123.0 (1C), 122.5 (1C′), 116.8 (1C′ + 1C, *overlapped*), 87.4 (1C′ + 1C, *overlapped*), 85.5 (1C′), 85.4 (1C), 62.2 (1C′), 61.9 (1C), 57.9 (1C′), 57.6 (1C), 56.6 (1C′), 56.5 (1C), 37.5 (1C′), 37.1 (1C), 31.2 (1C′), 30.7 (1C), 28.2 (3C′ + 3C, *overlapped*) 13.7 (1C′), 13.5 (1C) ppm. IR (KBr): ν = 1790 (C=O, ester, amide), 1761 (C=O, ester, amide), 1732 (C=O, ester, amide), 1556 (NO_2_), 1371 (NO_2_), 752 (C–Cl) cm^–1^. HRMS (ESI+) *m*/*z*: calcd. for C_20_H_23_ClN_2_NaO_7_ [M + Na]^+^: 461.1086, found: 461.1088.

#### 1′-(*tert*-Butyl) 2-Ethyl (1*R*,2*R*,3*R*)-5′-Bromo-3-nitro-2′-oxospiro[cyclopentane-1,3′-indoline]-1′,2-dicarboxylate (**3m**)

The title compound was synthesized according to the GP2, using methyleneindolinone **1m** (39.6 mg, 0.1 mmol) and 1-bromo-3-nitropropane (25.0 mg, 0.15 mmol) at a reaction time of 22 h. The product was purified by column chromatography (hexane/EtOAc = 10/1). The diastereomeric ratio of **3m**/**4m** > 20/1.

Yellow oil. Yield = 61% (29 mg). 99% ee. The enantiomeric excess of product **3m** was determined by HPLC (IA, *n*-heptane/*i*-PrOH = 90/10, flow rate = 1.0 mL/min, λ = 205 nm) at *t*_R_ = 5.1 min (minor) and 6.3 min (major). [α]_D_^20^ = +24.1 (*c* = 1.0, CHCl_3_). ^1^H NMR (400 MHz, chloroform-*d*): δ 7.76 (d, *J* = 8.5 Hz, 1H), 7.52–7.43 (m, 2H), 5.72–5.62 (m, 1H), 4.18 (d, *J* = 6.8 Hz, 1H), 4.10 (dq, *J* = 10.8, 7.1 Hz, 1H), 3.97 (dq, *J* = 10.7, 7.1 Hz, 1H), 3.07–2.90 (m, 1H), 2.47–2.20 (m, 3H), 1.62 (s, 9H), 1.08 (t, *J* = 7.2 Hz, 3H) ppm. ^13^C{^1^H} NMR (101 MHz, chloroform-*d*): δ 176.7, 168.2, 148.9, 138.9, 132.1, 131.7, 125.3, 118.0, 117.0, 87.4, 85.2, 62.2, 57.9, 56.6, 37.5, 31.2, 28.2 (3C), 13.7 ppm. IR (KBr): ν 1790 (C=O, ester, amide), 1761 (C=O, ester, amide), 1730 (C=O, ester, amide), 1552 (NO_2_), 1369 (NO_2_), 538 (C–Br) cm^–1^. HRMS (ESI+) *m*/*z*: calcd. for C_20_H_23_BrN_2_NaO_7_ [M + Na]^+^: 505.0580, found: 505.0577.

#### 1′-(*tert*-Butyl) 2-Ethyl 6′-Bromo-3-nitro-2′-oxospiro[cyclopentane-1,3′-indoline]-1′,2-dicarboxylate (**3n**/**4n**)

The title compounds were synthesized according to the GP2, using methyleneindolinone **1n** (39.6 mg, 0.1 mmol) and 1-bromo-3-nitropropane (25.0 mg, 0.15 mmol) at a reaction time of 42 h. The products were purified by column chromatography (hexane/EtOAc = 10/1). The diastereomeric ratio of **3n**/**4n** = 3/1.

Inseparable mixture of diastereomers **3n**/**4n** = 4/1. Yellow oil. Combined yield **3n**/**4n** = 36% (17 mg). 97/97% ee (**3n**/**4n**). The enantiomeric excess of product **3n** was determined by HPLC (IA, *n*-heptane/*i*-PrOH = 80/20, flow rate = 1.0 mL/min, λ = 221 nm) at *t*_R_ = 4.4 min (minor) and 5.6 min (major). The enantiomeric excess of product **4n** was determined by HPLC (IA, *n*-heptane/*i*-PrOH = 80/20, flow rate = 1.0 mL/min, λ = 221 nm) at *t*_R_ = 5.2 min (major) and 6.2 min (minor). ^1^H NMR (400 MHz, chloroform-*d*, **3n** – H′, **4n** – H): δ 8.15 (d, *J* = 1.8 Hz, 1H), 8.09 (d, *J* = 1.8 Hz, 1H′), 7.39 (dd, *J* = 8.0, 1.8 Hz, 1H′), 7.31–7.28 (m, 1H), 7.25 (d, *J* = 8.1 Hz, 1H′), 6.91 (d, *J* = 8.1 Hz, 1H), 5.67 (ddd, *J* = 9.4, 6.7, 3.5 Hz, 1H′), 5.59 (td, *J* = 8.8, 6.1 Hz, 1H), 4.30 (d, *J* = 8.4 Hz, 1H), 4.17 (d, *J* = 6.6 Hz, 1H′), 4.15–4.07 (m, 1H′), 3.97 (dq, *J* = 10.8, 7.1 Hz, 1H′), 3.79 (q, *J* = 7.1 Hz, 2H), 3.07–2.86 (m, 1H′), 2.79–2.63 (m, 2H), 2.56 (dt, *J* = 13.1, 8.9 Hz, 1H), 2.45–2.35 (m, 1H′), 2.31–2.22 (m, 2H′), 1.99 (ddd, *J* = 12.7, 7.4, 4.8 Hz, 1H), 1.66 (s, 9H), 1.63 (s, 9H′), 1.08 (t, *J* = 7.2 Hz, 3H′), 0.82 (t, *J* = 7.1 Hz, 3H) ppm. ^13^C{^1^H} NMR (101 MHz, chloroform-*d*, **3n** – C′, **4n** – C): δ 176.9 (1C′), 175.8 (1C), 168.3 (1C′), 168.0 (1C), 148.9 (1C′ + 1C, *overlapped*), 140.9 (1C′), 140.7 (1C), 128.6 (1C), 128.1 (1C′), 128.0 (1C), 127.8 (1C′), 123.9 (1C), 123.3 (1C′), 123.2 (1C), 122.8 (1C′), 119.0 (1C), 118.9 (1C′), 87.5 (1C′ + 1C, *overlapped*), 85.5 (1C), 85.4 (1C′), 62.2 (1C′), 61.9 (1C), 57.8 (1C′), 57.5 (1C), 56.5 (1C′), 56.3 (1C), 37.5 (1C′), 37.1 (1C), 31.3 (1C′), 30.7 (1C), 28.2 (3C′ + 3C, *overlapped*), 13.8 (1C′), 13.5 (1C) ppm. IR (KBr): ν = 1792 (C=O, ester, amide), 1763 (C=O, ester, amide), 1732 (C=O, ester, amide), 1552 (NO_2_), 1369 (NO_2_), 528 (C–Br) cm^–1^. HRMS (ESI+) *m*/*z*: calcd. for C_20_H_23_BrN_2_NaO_7_ [M + Na]^+^: 505.0580; found: 505.0580.

#### 1′-(*tert*-Butyl) 2-Methyl (1*R*,2*R*,3*R*)-3-Nitro-2′-oxospiro[cyclopentane-1,3′-indoline]-1′,2-dicarboxylate (**3o**)

The title compound was synthesized according to the GP2, using methyleneindolinone **1o** (30.3 mg, 0.1 mmol) and 1-bromo-3-nitropropane (25.0 mg, 0.15 mmol) at a reaction time of 46 h. The product was purified by column chromatography (hexane/EtOAc = 10/1). The diastereomeric ratio of **3o**/**4o** = 7/1.

Yellow oil. Yield = 59% (23 mg). 96% ee. The enantiomeric excess of product **3o** was determined by HPLC (IA, *n*-heptane/*i*-PrOH = 80/20, flow rate = 1.0 mL/min, λ = 210 nm) at *t*_R_ = 4.6 min (minor) and 5.2 min (major). [α]_D_^20^ = +8.8 (*c* = 1.0, CHCl_3_). ^1^H NMR (400 MHz, chloroform-*d*): δ 7.85 (dt, *J* = 8.0, 0.8 Hz, 1H), 7.41–7.31 (m, 2H), 7.24 (td, *J* = 7.5, 1.1 Hz, 1H), 5.69 (ddd, *J* = 9.5, 6.9, 3.6 Hz, 1H), 4.24 (d, *J* = 6.9 Hz, 1H), 3.58 (s, 3H), 3.07–2.93 (m, 1H), 2.41 (ddt, *J* = 14.3, 7.4, 3.7 Hz, 1H), 2.35–2.21 (m, 2H), 1.64 (s, 9H) ppm. ^13^C{^1^H} NMR (101 MHz, chloroform-*d*): δ 177.6, 169.0, 149.1, 139.9, 129.4, 129.2, 125.2, 122.0, 115.5, 87.6, 84.8, 58.0, 56.7, 52.9, 37.5, 31.3, 28.2 (3C) ppm. IR (KBr): ν 1763 (C=O, ester, amide), 1738 (C=O, ester, amide), 1724 (C=O, ester, amide), 1556 (NO_2_), 1352 (NO_2_) cm^–1^. HRMS (ESI+) *m*/*z*: calcd. for C_19_H_22_N_2_NaO_7_ [M + Na]^+^: 413.1319; found: 413.1321.

#### 2-Benzyl 1′-(*tert*-Butyl) (1*R*,2*R*,3*R*)-3-Nitro-2′-oxospiro[cyclopentane-1,3′-indoline]-1′,2-dicarboxylate (**3p**)

The title compound was synthesized according to the GP2, using methyleneindolinone **1p** (37.9 mg, 0.1 mmol) and 1-bromo-3-nitropropane (25.0 mg, 0.15 mmol) at a reaction time of 70 h. The product was purified by column chromatography (hexane/EtOAc = 10/1). The diastereomeric ratio of **3p**/**4p** = 6/1.

Yellow oil. Yield = 36% (17 mg). 99% ee. The enantiomeric excess of product **3p** was determined by HPLC (IA, *n*-heptane/*i*-PrOH = 80/20, flow rate = 1.0 mL/min, λ = 217 nm) at *t*_R_ = 4.7 min (minor) and 6.5 min (major). [α]_D_^20^ = +7.1 (*c* = 1.0, CHCl_3_). ^1^H NMR (400 MHz, chloroform-*d*): δ 7.76 (ddd, *J* = 8.1, 1.1, 0.6 Hz, 1H), 7.42–7.27 (m, 2H), 7.29–7.19 (m, 4H), 7.04–6.94 (m, 2H), 5.74 (ddd, *J* = 9.6, 6.9, 3.6 Hz, 1H), 5.06–4.91 (m, 2H), 4.28 (d, *J* = 7.0 Hz, 1H), 3.10–2.92 (m, 1H), 2.47–2.20 (m, 3H), 1.58 (s, 9H) ppm. ^13^C{^1^H} NMR (101 MHz, chloroform-*d*): δ 177.4, 168.3, 148.9, 139.8, 134.5, 129.3, 129.1, 128.6 (2C), 128.4, 127.7 (2C), 125.1, 122.0, 115.5, 87.5, 84.7, 67.7, 57.6, 56.8, 37.7, 31.0, 28.2 (3C) ppm. IR (KBr): ν 1780 (C=O, ester, amide), 1730 (C=O, ester, amide), 1709 (C=O, ester, amide), 1543 (NO_2_), 1369 (NO_2_) cm^–1^. HRMS (ESI+) *m*/*z*: calcd. for C_25_H_26_N_2_NaO_7_ [M + Na]^+^: 489.1632, found: 489.1630.

#### Di-*tert*-butyl (1*R*,2*R*,3*R*)-3-Nitro-2′-oxospiro[cyclopentane-1,3′-indoline]-1′,2-dicarboxylate (**3q**)

The title compound was synthesized according to the GP2, using methyleneindolinone **1q** (34.5 mg, 0.1 mmol) and 1-bromo-3-nitropropane (25.0 mg, 0.15 mmol) at a reaction time of 22 h. The product was purified by column chromatography (hexane/EtOAc - 10/1). The diastereomeric ratio of **3q**/**4q** = 11/1.

Yellow oil. Yield = 39% (16 mg). 99% ee. The enantiomeric excess of product **3q** was determined by HPLC (IA, *n*-heptane/*i*-PrOH = 80/20, flow rate = 1.0 mL/min, λ = 211 nm) at *t*_R_ = 4.0 min (minor) and 4.5 min (major). [α]_D_^20^ = +12.3 (*c* = 0.6, CHCl_3_). ^1^H NMR (400 MHz, chloroform-*d*): δ 7.85 (dt, *J* = 8.2, 0.7 Hz, 1H), 7.38 (dt, *J* = 7.3, 0.9 Hz, 1H), 7.34 (td, *J* = 7.9, 1.5 Hz, 1H), 7.23 (dd, *J* = 7.5, 1.1 Hz, 1H), 5.68 (ddd, *J* = 9.5, 6.6, 3.5 Hz, 1H), 4.10 (d, *J* = 6.6 Hz, 1H), 3.03–2.90 (m, 1H), 2.44–2.34 (m, 1H), 2.33–2.20 (m, 2H), 1.63 (s, 9H), 1.21 (s, 9H) ppm. ^13^C{^1^H} NMR (101 MHz, chloroform-*d*): δ 177.3, 167.5, 149.2, 139.8, 130.2, 128.9, 125.1, 122.0, 115.2, 87.7, 84.7, 83.2, 58.6, 56.9, 37.9, 31.1, 28.2 (3C), 27.6 (3C) ppm. IR (KBr): ν 1782 (C=O, ester, amide), 1739 (C=O, ester, amide), 1726 (C=O, ester, amide), 1543 (NO_2_), 1371 (NO_2_) cm^–1^. HRMS (ESI+) *m*/*z*: calcd. for C_22_H_28_N_2_NaO_7_ [M + Na]^+^: 455.1789, found: 455.1786.

#### 1′-(*tert*-Butyl) 2,2-Diethyl 3-Nitro-2′-oxospiro[cyclopentane-1,3′-indoline]-1′,2,2-tricarboxylate (**3r**)

The title compound was synthesized according to the GP2, using methyleneindolinone **1r** (38.9 mg, 0.1 mmol) and 1-bromo-3-nitropropane (25.0 mg, 0.15 mmol) at a reaction time of 168 h (no full conversion of **1r** was observed). The product was purified by column chromatography (hexane/EtOAc = 10/1). The diastereomeric ratio of **3r**/**4r** = 13/1.

Yellow oil. Yield = 53% (25 mg). 65% ee. The enantiomeric excess of product **3r** was determined by HPLC (IB, *n*-heptane/*i*-PrOH = 80/20, flow rate = 1.0 mL/min, λ = 208 nm) at *t*_R_ = 4.8 min (minor) and 5.4 min (major). [α]_D_^20^ = −26.0 (*c* = 0.4, CHCl_3_). ^1^H NMR (400 MHz, chloroform-*d*): δ 7.84 (d, *J* = 8.2 Hz, 1H), 7.35 (t, *J* = 7.8 Hz, 1H), 7.19 (d, *J* = 7.6 Hz, 1H), 7.14 (t, *J* = 7.6 Hz, 1H), 6.15 (dd, *J* = 11.2, 7.8 Hz, 1H), 4.37 (dq, *J* = 10.8, 7.1 Hz, 1H), 4.27 (dq, *J* = 10.8, 7.1 Hz, 1H), 4.14 (dq, *J* = 11.2, 7.2 Hz, 1H), 4.01 (dq, *J* = 10.8, 7.1 Hz, 1H), 3.01–2.91 (m, 1H), 2.90–2.80 (m, 1H), 2.69 (td, *J* = 12.7, 12.2, 7.3 Hz, 1H), 2.17 (ddd, *J* = 12.8, 9.2, 3.1 Hz, 1H), 1.62 (s, 9H), 1.26 (t, *J* = 7.1 Hz, 3H), 1.05 (t, *J* = 7.1 Hz, 3H) ppm. ^13^C{^1^H} NMR (101 MHz, chloroform-*d*): δ 177.5, 167.4, 166.7, 148.9, 140.3, 129.5, 127.0, 124.9, 123.4, 115.1, 88.3, 85.1, 68.2, 62.9, 62.4, 59.9, 34.4, 28.2 (3C), 27.7, 13.8, 13.4 ppm. IR (KBr): ν 1786 (C=O, ester, amide), 1728 (C=O, ester, amide), 1552 (NO_2_), 1346 (NO_2_) cm^–1^. HRMS (ESI+) *m*/*z*: calcd. for C_23_H_28_N_2_NaO_9_ [M + Na]^+^: 499.1687, found: 499.1683.

#### *tert*-Butyl 3-Nitro-2′-oxo-2-(trifluoromethyl)spiro[cyclopentane-1,3′-indoline]-1′-carboxylate (**3s/4s**)

The title compounds were synthesized according to the GP2, using methyleneindolinone **1s** (31.3 mg, 0.1 mmol) and 1-bromo-3-nitropropane (25.0 mg, 0.15 mmol) at a reaction time of 64 h. The products were purified by column chromatography (hexane/EtOAc = 10/1). The diastereomeric ratio of **3s/4s** = 2/1.

#### *tert*-Butyl (1*R*,2*R*,3*R*)-3-Nitro-2′-oxo-2-(trifluoromethyl)spiro[cyclopentane-1,3′-indoline]-1′-carboxylate (**3s**)

Yellow oil. Yield = 18% (7 mg). 9% ee. The enantiomeric excess of product **3s** was determined by HPLC (IA, *n*-heptane/*i*-PrOH = 80/20, flow rate = 1.0 mL/min, λ = 208 nm) at *t*_R_ = 5.9 min (major) and 7.6 min (minor). [α]_D_^20^ = −7.9 (*c* = 0.4, CHCl_3_). ^1^H NMR (400 MHz, chloroform-*d*): δ 7.95 (d, *J* = 8.2 Hz, 1H), 7.39 (t, *J* = 7.7 Hz, 1H), 7.23–7.13 (m, 2H), 5.42 (td, *J* = 9.2, 4.4 Hz, 1H), 4.35–4.22 (m, 1H), 2.93–2.80 (m, 1H), 2.68 (ddd, *J* = 14.1, 7.8, 3.6 Hz, 1H), 2.61 (dt, *J* = 12.6, 9.8 Hz, 1H), 2.02 (ddd, *J* = 12.0, 7.5, 2.7 Hz, 1H), 1.66 (s, 9H) ppm. ^13^C{^1^H} NMR (101 MHz, chloroform-*d*): δ 175.1, 148.9, 139.2, 129.7, 126.9, 124.7, 124.6 (q, *J* = 279.3 Hz), 124.1, 115.8, 85.4, 84.9, 56.3 (q, *J* = 28.6 Hz), 55.8, 38.4, 31.6, 28.2 (3C) ppm. ^19^F NMR (376 MHz, chloroform-*d*): δ −65.46 (d, *J* = 8.8 Hz) ppm. IR (KBr): ν 1763 (C=O, ester, amide), 1732 (C=O, ester, amide), 1554 (NO_2_), 1371 (NO_2_), 1275 (C–CF_3_) cm^–1^. HRMS (ESI+) *m*/*z*: calcd. for C_18_H_19_F_3_N_2_NaO_5_ [M + Na]^+^: 423.1138, found: 423.1126.

#### *tert*-Butyl 3-Nitro-2′-oxo-2-(trifluoromethyl)spiro[cyclopentane-1,3′-indoline]-1′-carboxylate (**4s**)

Yellow oil. Yield = 15% (6 mg). 6% ee. The enantiomeric excess of product **4s** was determined by HPLC (IB, *n*-heptane/*i*-PrOH = 80/20, flow rate = 1.0 mL/min, λ = 261 nm) at *t*_R_ = 4.8 min (minor) and 6.1 min (major). [α]_D_^20^ ∼ 0 (*c* = 0.3, CHCl_3_). ^1^H NMR (400 MHz, chloroform-*d*): δ 7.86 (d, *J* = 8.4 Hz, 1H), 7.38 (t, *J* = 7.2 Hz, 2H), 7.27 (d, *J* = 14.9 Hz, 1H), 5.66–5.57 (m, 1H), 4.03–3.98 (m, 1H), 3.26–3.14 (m, 1H), 2.47–2.35 (m, 2H), 2.28 (dd, *J* = 11.4, 8.3 Hz, 1H), 1.64 (s, 9H) ppm. ^13^C{^1^H} NMR (101 MHz, chloroform-*d*): δ 174.9, 148.9, 139.6, 129.8, 126.8, 125.4, 124.5 (q, *J* = 280.3 Hz), 122.1, 115.6, 85.3, 84.6, 56.9 (q, *J* = 28.6 Hz), 55.2, 37.3, 30.6, 28.2 (3C) ppm. ^19^F NMR (376 MHz, chloroform-*d*): δ −65.34 (d, *J* = 8.4 Hz) ppm. IR (KBr): ν 1790 (C=O, ester, amide), 1759 (C=O, ester, amide), 1732 (C=O, ester, amide), 1552 (NO_2_), 1369 (NO_2_), 1250 (C–CF_3_) cm^–1^. HRMS (ESI+) *m*/*z*: calcd. for C_18_H_19_F_3_N_2_NaO_5_ [M + Na]^+^: 423.1138, found: 423.1141.

#### *tert*-Butyl 2-benzoyl-2′-oxospiro[cyclopentane-1,3′-indolin]-2-ene-1′-carboxylate (**5t**)

The title compound was synthesized according to the GP2, using methyleneindolinone **1t** (34.9 mg, 0.1 mmol) and 1-bromo-3-nitropropane (25.0 mg, 0.15 mmol) at a reaction time of 18 h. The products were purified by column chromatography (hexane/EtOAc = 10/1). Yellow oil. Yield = 47% (18 mg). 20% ee. The enantiomeric excess of product **5t** was determined by HPLC (IA, *n*-heptane/*i*-PrOH = 80/20, flow rate = 1.0 mL/min, λ = 216 nm) at *t*_R_ = 4.9 min (minor) and 6.6 min (major). [α]_D_^20^ ∼ 0 (*c* = 0.6, CHCl_3_). ^1^H NMR (400 MHz, chloroform-*d*): δ 7.89 (dt, *J* = 8.1, 0.8 Hz, 1H), 7.71–7.65 (m, 2H), 7.54–7.48 (m, 1H), 7.43–7.35 (m, 2H), 7.27 (ddd, *J* = 8.2, 6.7, 2.3 Hz, 1H), 7.13–7.04 (m, 2H), 6.94 (t, *J* = 2.6 Hz, 1H), 3.11–2.83 (m, 2H), 2.69 (ddd, *J* = 13.3, 8.9, 5.1 Hz, 1H), 2.28 (ddd, *J* = 13.3, 8.6, 6.1 Hz, 1H), 1.67 (s, 9H) ppm. ^13^C{^1^H} NMR (101 MHz, chloroform-*d*): δ 191.0, 177.7, 150.0, 149.7, 145.8, 140.1, 137.9, 132.5, 132.1, 129.1 (2C), 128.5, 128.4 (2C), 124.6, 121.9, 115.4, 84.2, 61.3, 37.7, 33.3, 28.3 (3C) ppm. IR (KBr): ν 1778 (C=O, ester, amide) cm^–1^. HRMS (ESI+) *m*/*z*: calcd. for C_24_H_23_NNaO_4_ [M + Na]^+^: 412.1519, found: 412.1520.

#### *tert*-Butyl 3-Nitro-2′-oxo-2-phenylspiro[cyclopentane-1,3′-indoline]-1′-carboxylate (**3v**)

The title compound was synthesized according to the GP2, using methyleneindolinone **1v** (32.1 mg, 0.1 mmol) and 1-bromo-3-nitropropane (25.0 mg, 0.15 mmol) at a reaction time of 65 h. The product was purified by column chromatography (hexane/EtOAc = 7/1). The diastereomeric ratio of **3v**/**4v** = 4/1.

Yellow oil. Yield = 45% (41 mg). 12% ee. The enantiomeric excess of product **3v** was determined by HPLC (IB, *n*-heptane/*i*-PrOH = 80/20, flow rate = 1.0 mL/min, λ = 199 nm) at *t*_R_ = 5.2 min (major) and 5.9 min (minor). [α]_D_^20^ = +4.7 (*c* = 0.6, CHCl_3_). ^1^H NMR (400 MHz, chloroform-*d*): δ 7.61–7.55 (m, 1H), 7.51–7.46 (m, 1H), 7.30–7.24 (m, 2H), 7.19–7.07 (m, 3H), 6.92 (dt, *J* = 8.6, 2.1 Hz, 2H), 5.99–5.91 (m, 1H), 4.09 (d, *J* = 10.5 Hz, 1H), 3.06 (dtd, *J* = 13.2, 8.9, 7.7 Hz, 1H), 2.66–2.55 (m, 1H), 2.50 (ddd, *J* = 13.6, 10.6, 7.6 Hz, 1H), 2.40 (ddd, *J* = 13.5, 8.8, 4.3 Hz, 1H), 1.52 (s, 9H) ppm. ^13^C{^1^H} NMR (101 MHz, chloroform-*d*): δ 177.4, 148.6, 139.8, 132.5, 129.1, 128.6, 128.5 (2C), 128.4, 127.7 (2C), 125.0, 122.4, 115.1, 87.7, 84.4, 61.4, 60.1, 34.1, 29.9, 28.1 (3C) ppm. IR (KBr): ν 1786 (C=O, amide), 1755 (C=O, amide), 1730 (C=O, amide), 1549 (NO_2_), 1350 (NO_2_) cm^–1^. HRMS (ESI+) *m*/*z*: calcd. for C_23_H_24_N_2_NaO_5_ [M + Na]^+^: 431.1577, found: 431.1573.

#### 1′-(*tert*-Butyl) 2-Ethyl 3-nitro-2′-oxospiro[cyclopropane-1,3′-indoline]-1′,2-dicarboxylate (**6/6′**)

The title compounds were synthesized according to the GP2, using methyleneindolinone **1a** (31.7 mg, 0.1 mmol) and commercially available bromonitromethane (21.0 mg, 0.15 mmol) at a reaction time of 72 h. The products were purified by column chromatography (hexane/EtOAc = 8/1). The diastereomeric ratio of **6**/**6′** = 4/1.

Inseparable mixture of diastereomers **6**/**6′** = 4/1. Light yellow oil. Combined yield **6**/**6′** = 59% (22 mg). 90/84% ee (**6**/**6′**). The enantiomeric excess of product **6** was determined by HPLC (IB, *n*-heptane/*i*-PrOH = 80/20, flow rate = 1.0 mL/min, λ = 227 nm) at *t*_R_ = 11.9 min (major) and 16.8 min (minor). The enantiomeric excess of product **6′** was determined by HPLC (IB, *n*-heptane/*i*-PrOH = 80/20, flow rate = 1.0 mL/min, λ = 227 nm) at *t*_R_ = 12.7 min (major) and 24.0 min (minor). ^1^H NMR (400 MHz, chloroform-*d*, diastereomer **6** – H′, diastereomer **6′** – H): δ 7.98 (dt, *J* = 8.2, 0.8 Hz, 1H), 7.94 (dt, *J* = 8.3, 0.8 Hz, 1H′), 7.44–7.38 (m, 1H′ + 1H, *overlapped*), 7.29 (ddd, *J* = 7.7, 1.4, 0.6 Hz, 1H), 7.22–7.17 (m, 2H′), 7.16 (dd, *J* = 2.5, 1.0 Hz, 1H), 5.30 (d, *J* = 6.2 Hz, 1H′), 5.27 (d, *J* = 6.3 Hz, 1H), 4.33–4.22 (m, 2H′ + 1H, *overlapped*), 4.22–4.11 (m, 1H), 3.83 (d, *J* = 6.2 Hz, 1H′), 3.80 (d, *J* = 6.3 Hz, 1H), 1.63 (s, 9H′), 1.63 (s, 9H), 1.30 (t, *J* = 7.1 Hz, 3H′), 1.24 (t, *J* = 7.1 Hz, 3H) ppm. ^13^C{^1^H} NMR (chloroform-*d*, diastereomer **6** – C′, diastereomer **6′** – C) δ 168.3 (1C′), 167.4 (1C), 164.4 (1C), 163.0 (1C′), 148.7 (1C), 148.5 (1C′), 141.1 (1C′), 140.8 (1C), 130.3 (1C′), 130.1 (1C), 125.1 (1C′), 124.9 (1C), 122.4 (1C), 122.0 (1C′), 121.1 (1C), 120.2 (1C′), 115.7 (1C′), 115.6 (1C), 85.6 (1C′ + 1C, *overlapped*), 70.1 (1C′), 68.8 (1C), 62.8 (1C′ + 1C, *overlapped*), 40.1 (1C′), 39.4 (1C), 38.2 (1C), 35.9 (1C′), 28.2 (3C′ + 3C, *overlapped*), 14.1 (1C′ + 1C, *overlapped*) ppm. IR (ATR): ν 1793 (C=O, ester, amide), 1765 (C=O, ester, amide), 1736 (C=O, ester, amide), 1554 (NO_2_), 1350 (NO_2_) cm^–1^. HRMS (ESI+) *m*/*z*: calcd. for C_18_H_20_N_2_NaO_7_ [M + Na]^+^, 399.1163; found, 399.1161.

### Gram-Scale Organocascade Reaction and Late-Stage Transformations

#### 1′-(*tert*-Butyl) 2-Ethyl (1*R*,2*R*,3*R*)-3-Nitro-2′-oxospiro[cyclopentane-1,3′-indoline]-1′,2-dicarboxylate (**3a**)

The catalyst **C1** (12.4 mg, 0.001 mmol, 0.01 equiv) was added to a solution of the methyleneindolinone **1a** (1000.0 mg, 3.14 mmol, 1.0 equiv) in anhydrous chloroform (16 mL) at room temperature. Then, 1-bromo-3-nitropropane (786 mg, 4.71 mmol, 1.5 equiv) and potassium carbonate (650 mg, 4.71 mmol, 1.5 equiv) were added. The reaction was stirred at room temperature for 48 h. Then catalyst **C1** (12.4 mg, 0.001 mmol, 0.01 equiv) was added. The addition of **C1** (12.4 mg, 0.001 mmol, 0.01 equiv) was repeated after the next 48 h. With complete conversion of the methyleneindolinone **1a** (TLC monitored, reaction time: 161 h) solvent was removed under reduced pressure. Crude product was purified by column chromatography (hexane/EtOAc = 10/1). The diastereomeric ratio of **3a**/**4a** > 20/1. Yield of **3a** = 61% (771 mg). All analytical data matched the data of identical compounds prepared on a smaller scale

#### Ethyl (1*R*,2*R*,3*R*)-3-Nitro-2′-oxospiro[cyclopentane-1,3′-indoline]-2-carboxylate (**3f**)

TFA (38 μL, 0.5 mmol, 5.0 equiv) was dropwise added (during 1 min) to a stirred solution of spirocycle **3a** (40 mg, 0.1 mmol, 1.0 equiv., 99% ee) in anhydrous DCM (2.0 mL) at rt. At this temperature, the reaction mixture was stirred for 2 h. After the full disappearance of starting material (monitored by TLC), the reaction was quenched by careful addition of a saturated solution of NaHCO_3_ (5 mL). The resulting mixture was diluted with DCM (5 mL). The organic phase was separated, and the water phase was extracted with DCM (3 × 10 mL). Collected organic phases were washed with brine (1 × 10 mL) and dried over MgSO_4_. After filtration of the drying agent, solvents were removed under reduced pressure. The crude product was purified by column chromatography with a mixture of hexane/EtOAc as an eluent (2/1).

Colorless oil. Yield = 90% (27 mg). 99% ee. The enantiomeric excess of product **3f** was determined by HPLC (IC, *n*-heptane/*i*-PrOH = 80/20, flow rate = 1.0 mL/min, λ = 209 nm) at *t*_R_ = 9.5 min (major) and 12.2 min (minor). [α]_D_^20^ = +28.6 (*c* = 1.1, CHCl_3_). ^1^H NMR (600 MHz, chloroform-*d*): δ 8.28 (br s, 1H), 7.35 (d, *J* = 7.4 Hz, 1H), 7.30–7.23 (m, 1H), 7.11 (t, *J* = 7.5 Hz, 1H), 6.90 (d, *J* = 7.7 Hz, 1H), 5.73 (ddd, *J* = 9.4, 7.2, 4.1 Hz, 1H), 4.18 (d, *J* = 7.2 Hz, 1H), 4.10–3.96 (m, 2H), 3.00 (dtd, *J* = 14.1, 10.1, 7.8 Hz, 1H), 2.46–2.37 (m, 1H), 2.32 (ddd, *J* = 13.4, 10.5, 8.8 Hz, 1H), 2.23 (ddd, *J* = 13.5, 7.8, 3.0 Hz, 1H), 1.07 (t, *J* = 7.1 Hz, 3H). ppm. ^13^C{^1^H} NMR (151 MHz, chloroform-*d*): δ 180.9, 168.7, 140.9, 131.2, 128.9, 123.2, 122.5, 110.1, 87.4, 61.9, 56.8, 56.5, 36.4, 30.9, 13.8 ppm. IR (ATR): ν 3159 (N–H, secondary amide), 1726 (C=O, ester, amide), 1701 (C=O, ester, amide), 1728 (C=O, ester, amide), 1545 (NO_2_), 1360 (NO_2_) cm^–1^. HRMS (ESI+) *m*/*z*: calcd. for C_15_H_16_NO_3_ [M + Na]^+^: 327.0951, found: 327.0956.

#### 1′-(*tert*-Butyl) 2-Ethyl (*S*)-2′-Oxospiro[cyclopentane-1,3′-indolin]-2-ene-1′,2-dicarboxylate (**5a**)

DBU (30 μL, 0.2 mmol, 2.0 equiv) was added in one portion to a stirred solution of spirocycle **3a** (40 mg, 0.1 mmol, 1.0 equiv, 99% ee) in DMSO (2.0 mL) at rt. At this temperature, the reaction mixture was stirred for 15 h. After the full disappearance of starting material (monitored by TLC), the reaction mixture was diluted with brine (10 mL). The resulting solution was extracted with EtOAc (3 × 10 mL). Collected organic phases were washed with brine (2 × 10 mL) and dried over MgSO_4_. After filtration of the drying agent, solvents were removed under reduced pressure. The crude product was purified by column chromatography with a mixture of hexane/EtOAc as an eluent (7/1).

White semisolid. Yield = 92% (32 mg). 99% ee. The enantiomeric excess of product **5a** was determined by HPLC (IB, *n*-heptane/*i*-PrOH = 98/2, flow rate = 1.0 mL/min, λ = 243 nm) at *t*_R_ = 10.5 min (major) and 14.7 min (minor). [α]_D_^20^ = −38.7 (*c* = 0.8, CHCl_3_). ^1^H NMR (600 MHz, chloroform-*d*): δ 7.84 (d, *J* = 8.2 Hz, 1H), 7.27 (td, *J* = 7.9, 1.6 Hz, 1H), 7.19 (t, *J* = 2.6 Hz, 1H), 7.10 (td, *J* = 7.5, 1.0 Hz, 1H), 7.06 (dd, *J* = 7.5, 1.5 Hz, 1H), 3.93 (qd, *J* = 7.2, 1.9 Hz, 2H), 2.92–2.76 (m, 2H), 2.67 (ddd, *J* = 13.4, 9.2, 6.4 Hz, 1H), 2.24 (ddd, *J* = 13.2, 8.3, 4.8 Hz, 1H), 1.64 (s, 9H), 1.01 (t, *J* = 7.2 Hz, 3H) ppm. ^13^C{^1^H} NMR (151 MHz, chloroform-*d*): δ 177.9, 162.7, 149.5, 148.3, 139.5, 138.0, 132.4, 128.5, 124.6, 122.4, 115.0, 84.2, 60.6, 60.3, 38.2, 32.2, 28.2 (3C), 13.7 ppm. IR (ATR): ν 1793 (C=O, ester, amide), 1766 (C=O, ester, amide), 1707 (C=O, ester, amide) cm^–1^. HRMS (ESI+) *m*/*z*: calcd. for C_20_H_23_NNaO_5_ [M + Na]^+^: 380.1468, found: 380.1469.

#### 1′-(*tert*-Butyl) 2-Ethyl (1*R*,2*S*)-2′-Oxospiro[cyclopentane-1,3′-indoline]-1′,2-dicarboxylate (**9**)

A solution of spirocycle **5a** (18 mg, 0.05 mmol, 1.0 equiv, 99% ee) in MeOH (1.0 mL) was degassed (flask was evacuated and refilled with Ar three times), and Pd/C (10%, 10.6 mg, 0.2 equiv) was added. The reaction flask was evacuated again and refilled with H_2_ three times at rt. Under a hydrogen atmosphere (ballon), the reaction mixture was stirred for 15 h. After the full disappearance of starting material (monitored by TLC), the reaction mixture was filtered through a short pad of Celite (washed with a minimal amount of MeOH). The resulting filtrate was concentrated under reduced pressure. The crude product was purified by column chromatography with a mixture of hexane/EtOAc as an eluent (8/1). The diastereomeric ratio **9**/**9′** = 9/1.

Colorless oil. Yield = 97% (17 mg). 98% ee. The enantiomeric excess of product **9** was determined by HPLC (IC, *n*-heptane/*i*-PrOH = 80/20, flow rate = 1.0 mL/min, λ = 208 nm) at *t*_R_ = 7.6 min (minor) and 13.9 min (major). [α]_D_^20^ ∼ 0 (*c* = 1.0, CHCl_3_). ^1^H NMR (400 MHz, chloroform-*d*, only major diastereomer): δ 7.84 (dt, *J* = 8.2, 0.9 Hz, 1H), 7.31–7.25 (m, 1H), 7.21–7.08 (m, 2H), 4.03 (dq, *J* = 10.8, 7.1 Hz, 1H), 3.91 (dq, *J* = 10.8, 7.1 Hz, 1H), 3.23 (dd, *J* = 10.7, 8.6 Hz, 1H), 2.57–2.42 (m, 1H), 2.37–2.17 (m, 3H), 2.01–1.88 (m, 2H), 1.63 (s, 9H), 1.06 (t, *J* = 7.1 Hz, 3H). ppm. ^13^C{^1^H} NMR (101 MHz, chloroform-*d*, only major diastereomer): δ 178.5, 171.7, 149.6, 139.8, 132.9, 128.2, 124.6, 121.6, 115.0, 84.1, 60.9, 56.2, 55.8, 40.1, 28.9, 28.3 (3C), 24.2, 13.8 ppm. IR (ATR): ν 1790 (C=O, ester, amide), 1761 (C=O, ester, amide), 1724 (C=O, ester, amide) cm^–1^. HRMS (ESI+) *m*/*z*: calcd. for C_20_H_25_NNaO_5_ [M + Na]^+^: 382.1625, found: 382.1621.

#### *tert*-Butyl (*S*)-2-(Hydroxymethyl)-2′-oxospiro[cyclopentane-1,3′-indolin]-2-ene-1′-carboxylate (**10**)

A solution of DIBALH (25 wt.% in toluene, 150 μL, 0.22 mmol, 2.2 equiv) was dropwise added (during 3 min) to a stirred solution of spirocycle **5a** (36 mg, 0.1 mmol, 1.0 equiv, 99% ee) in anhydrous DCM (2.0 mL) at 0 °C (water/ice cooling bath). At this temperature, the reaction mixture was stirred for 1 h. After the full disappearance of starting material (monitored by TLC), the reaction was quenched by careful addition of MeOH (1 mL) followed with Rochelle’s salt solution (5 mL). Resulting gelly solution was stirred until it became a clear biphasic solution (aprox. 2 h). Then the organic phase was separated and the water phase extracted with DCM (3 × 10 mL). Collected organic phases were washed with brine (1 × 10 mL) and dried over MgSO_4_. After filtration of the drying agent, solvents were removed under reduced pressure. The crude product was purified by column chromatography with a mixture of hexane/EtOAc as an eluent (3/1 to 2/1).

Colorless oil. Yield = 84% (27 mg). 96% ee. The enantiomeric excess of product **10** was determined by HPLC (IC, *n*-heptane/*i*-PrOH = 80/20, flow rate = 1.0 mL/min, λ = 240 nm) at *t*_R_ = 8.0 min (minor) and 10.9 min (major). [α]_D_^20^ = −117.7 (*c* = 0.3, CHCl_3_). ^1^H NMR (400 MHz, chloroform-*d*): δ 7.98–7.61 (m, 1H), 7.26–7.14 (m, 2H), 6.99 (td, *J* = 7.5, 1.1 Hz, 1H), 5.75 (dd, *J* = 3.0, 1.5 Hz, 1H), 5.66 (s, 1H), 4.31 (d, *J* = 11.1 Hz, 1H), 3.89 (ddd, *J* = 7.4, 3.7, 1.9 Hz, 1H), 3.22–3.06 (m, 1H), 2.76 (ddd, *J* = 16.3, 8.7, 3.2 Hz, 1H), 2.41–2.23 (m, 2H), 1.60 (s, 9H) ppm. ^13^C{^1^H} NMR (101 MHz, chloroform-*d*): δ 149.5, 148.4, 128.6, 128.5, 124.7, 123.3, 122.5, 122.1, 114.9, 95.7, 64.9, 38.0, 37.5, 32.3, 28.6 (3C), 28.3 ppm. IR (ATR): ν 3408 (O–H, alcohol) 1705 (C=O, aldehyde, amide) cm^–1^. HRMS (ESI+) *m*/*z*: calcd. for C_18_H_23_NNaO_4_ [M + Na]^+^: 340.1519, found: 340.1513.

## Data Availability

The data underlying this study are available in the published article and its online Supporting Information.

## Supporting Information Available

Reaction condition optimization, crystallographic data, copies of ^1^H NMR, ^13^C NMR, and ^19^F NMR, and copies of chiral HPLC (PDF)
FAIR data, including the primary NMR FID files, for compounds **3**–**10** (ZIP)
FAIR data, including the primary NOE NMR FID files (ZIP)
