# Supplementary material for: Access to Spirooxindole-Fused Cyclopentanes via a Stereoselective Organocascade Reaction Using Bifunctional Catalysis
Source: J Org Chem. 2023 Jan 27;88(12):7724–35. doi: 10.1021/acs.joc.2c02478 (PMC10278142; doi:10.1021/acs.joc.2c02478)
Supplement: Supplementary file 1 — jo2c02478_si_001.pdf [file jo2c02478_si_001.pdf]

Access to Spirooxindole-fused Cyclopentanes via Stereoselective Organocascade Reaction using Bifunctional Catalysis

Andrea Vopálenská,[a] Vojtěch Dočekal,[a] Simona Petrželová,[b] Ivana Císařová,[c] Jan Veselý[*a]*

[a] Department of Organic Chemistry, Faculty of Science, Charles University, Hlavova 2030/8, 128 43 Prague 2, Czech Republic, E-mail: jan.vesely@natur.cuni.cz, http://orgchem.cz/vesely/

[b)] Department of Teaching and Didactics of Chemistry, Faculty of Science, Charles University, Hlavova 2030/8, 128 43 Prague 2, Czech Republic

[c)] Department of Inorganic Chemistry, Faculty of Science, Charles University, Hlavova 2030/8, 128 43 Prague 2, Czech Republic

Supporting information

Organocascade reaction **S2**

Full optimization of reaction conditions *S2*

Control experiments *S5*

Example of determination of diastereomeric ratio *S7*

NMR yield determination *S9*

Relative configuration determination **S11**

Crystallographic data **S19**

NMR spectra **S20**

Chiral HPLC **S55**

References **S83**

Organocascade reaction

Full optimization of reaction conditions

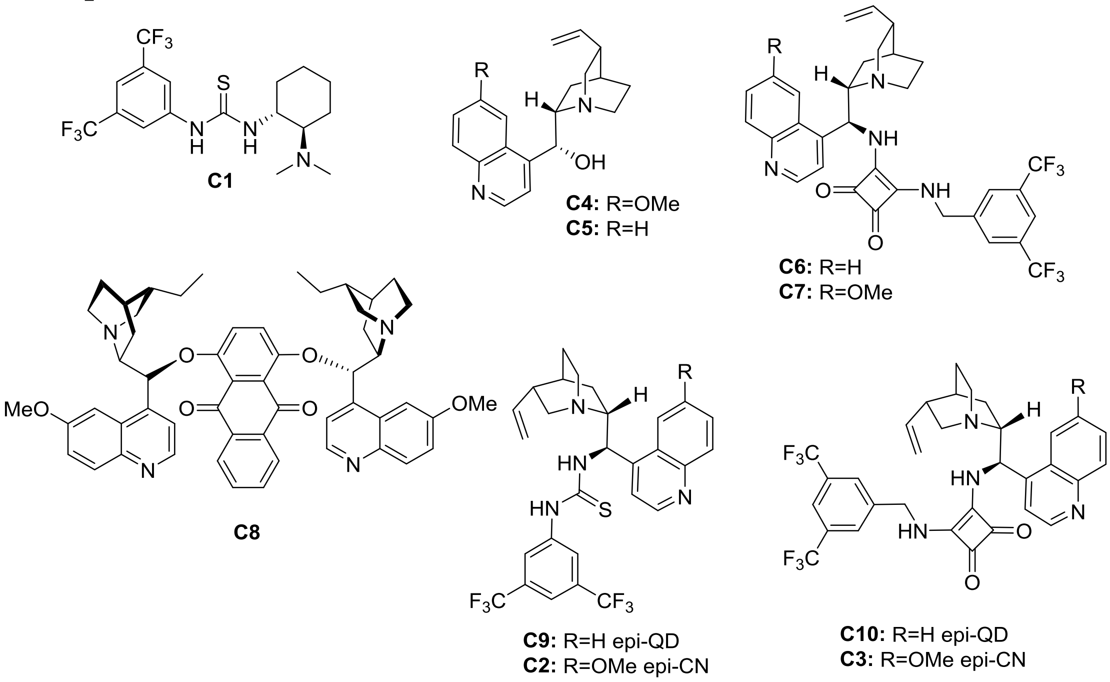

Figure S1: Screened organocatalysts.

Table S1. Catalyst screening.

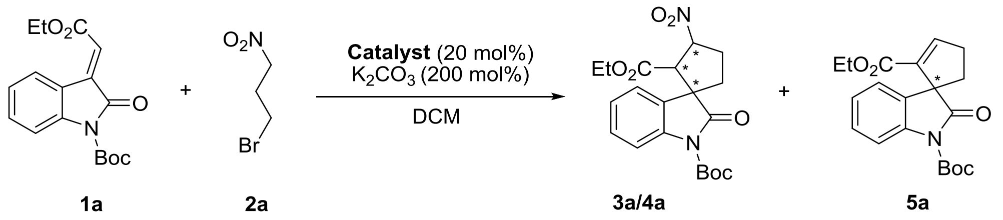

entry[a]	Catalyst	time (h)	dr (3a/4a)[b]	yield (%, 3a)[c]	*ee* (%, 3a)[d]
1	**C1**	24	17/1	58	99
2	**C4**	24	3/1	54	78
3	**C5**	24	3/1	56	79
4	**C6**	24	15/1	24	-98
5	**C7**	24	11/1	44	-97
6[e]	**C8**	48	n.d.	n.d.	n.d.
7	**C2**	24	7/1	24	83
8	**C9**	24	3/1	58	91
9	**C3**	24	3/1	32	90
10	**C10**	24	3/1	39	92

[a] Reactions were conducted with **1a** (0.1 mmol), **2a (**0.2 mmol), K_2CO_3 (0.2 mmol), and selected catalyst **(**20 mol%) in DCM (1.0 ml) at room temperature. [b] Determined by ^{1}H NMR of crude reaction mixture. [c] Isolated after column chromatography. [d] Determined by chiral HPLC. [e] Only traces of **5a** were formed.

Table S2. Base screening.

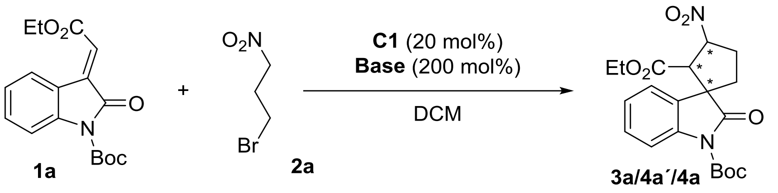

entry[a]	base	time (h)	dr (3a/4a´/4a)[b]	yield (%, 3a)[c]	*ee* (%, 3a)[d]
1	$NaHCO_3$	168	20/1/1	32	99
2	K_2CO_3	24	17/0/1	58	99
3	Na_2CO_3	48	20/0/1	55	99
4	Cs_2CO_3	24	2/0/1	23	74
5	NaOAc	24	9/1/1	35	99
6	DIPEA	24	2/0/1	49	98
7	2,6-lutidine	24	7/3/1	50	99
8	none	168[e]	n.d.	n.d.	n.d.

[a] Reactions were conducted with **1a** (0.1 mmol), **2a** (0.2 mmol), selected base (0.2 mmol), and **C1** (20 mol%) in DCM (1.0 ml) at room temperature. [b] Determined by ^{1}H NMR of crude reaction mixture. [c] Isolated after column chromatography. [d] Determined by chiral HPLC. [e] No conversion of starting **1a** was observed.

Table S3. Solvent screening.

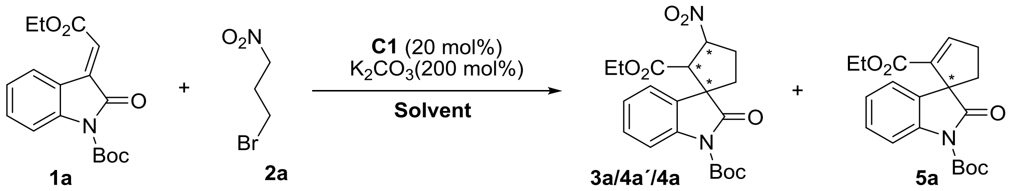

entry[a]	solvent	time (h)	dr (3a/4a´/4a)[b]	yield (%, 3a)[c]	*ee* (%, 3a)[d]
1	Toluene	24	2/0/1	25	86
2	Et_2O	24	6/1/1	27	88
3	MTBE	3	8/1/1	23	99
4	THF	2	2/0/1	29	92
5	$CHCl_3$	24	>20/2/1	59	99
6	DCM	24	17/0/1	58	99
7	1,2-DCE	27	17/0/1	44	99
8	EtOAc	2	3/0/1	38	91
9	MeCN	1	1/0/1	21	90
10	DMF	2	1/0/2	10	5
11[e]	MeOH	2	n.d.	n.d.	n.d.

[a] Reactions were conducted with selected amount of **1a**, **2a**, K_2CO_3 (0.2 mmol), and **C1 (**20 mol%) in $CHCl_3$ (selected volume) at room temperature. [b] Determined by ^{1}H NMR of crude reaction mixture. [c] Isolated after column chromatography. [d] Determined by chiral HPLC.

Table S4. Reagent ratio and concentration screening.

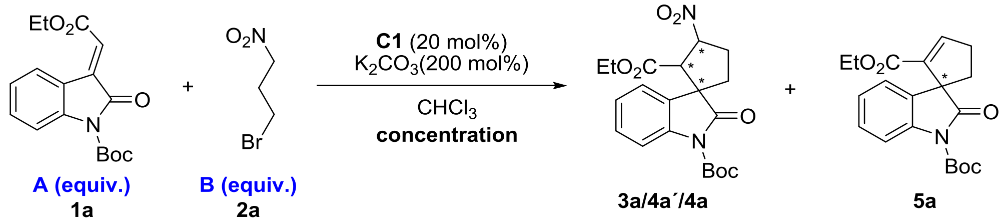

entry[a]	A	B	conc. (mol/l)	time (h)	dr (3a/4a′/4a)[b]	yield (%, 3a)[c]	*ee* (%, 3a)[d]
1	1.0	2.0	0.2	24	>20/2/1	59	99
2	1.0	2.0	0.1	17	>20/0/1	58	99
3	1.0	2.0	0.4	19	>20/0/1	58	99
4	1.0	1.5	0.2	18	>20/1/1	57	99
5	1.0	1.0	0.2	45	>20/1/1	43	99
6	1.5	1.0	0.2	41	>20/2/1	55	99

[a] Reactions were conducted with selected amount of **1a**, **2a**, K_2CO_3 (0.2 mmol), and **C1** (20 mol%) in selected $CHCl_3$ (selected volumel) at room temperature. [b] Determined by ^{1}H NMR of crude reaction mixture. [c] Isolated after column chromatography. [d] Determined by chiral HPLC.

Table S5. Catalyst and base ratio screening.

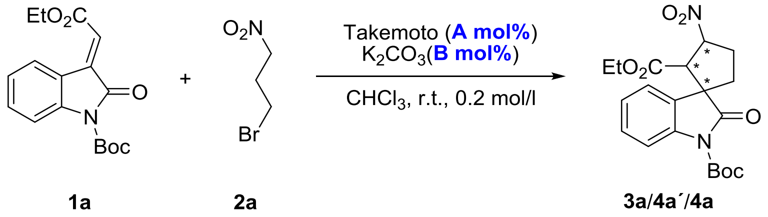

entry[a]	A	B	time (h)	dr (3a/4a′/4a)[b]	yield (%, 3a)[c]	*ee* (%, 3a)[d]
1	20	200	24	>20/2/1	59	99
2	15	200	41	>20/1/1	63	99
3	10	200	26	>20/0/1	55	99
4	5	200	20	>20/1/1	64	99
5	1	200	40	>20/0/1	61	99
6	1	150	45	>20/0/1	64	99
7[e]	1	150	45	>20/0/1	60	99

[a] Reactions were conducted with selected amount of **1a** (0.1 mmol) **2a** (0.2 mmol), K_2CO_3 (selected amount), and **C1** (selected amount) in $CHCl_3$ (1.0 ml) at room temperature. [b] Determined by ^{1}H NMR of crude reaction mixture. [c] Isolated after column chromatography. [d] Determined by chiral HPLC. [e] **2a** (0.15 mmol) used.

Control experiments

In order to explain stability of catalyst, which combined nucleophilic tertiary amine and thiourea units, we desinged control experiments:

Solution of **C1** (8.3 mg, 0.02 mmol, 1 equiv.), and **2a** (16.8 mg, 0.1 mmol, 5.0 equiv.) in $CDCl_3$ (0.7 ml) was stirred for indicated time at room temperature. ^{1}H NMR spectra were taken at different time points.

During that experiment (Figure S2), we did not observe any conversion of **C1** (*) or bromonitropropane **2a** (•).

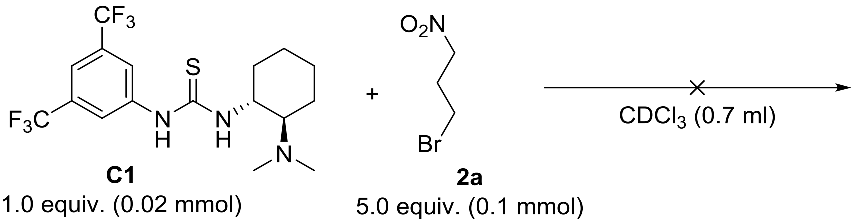

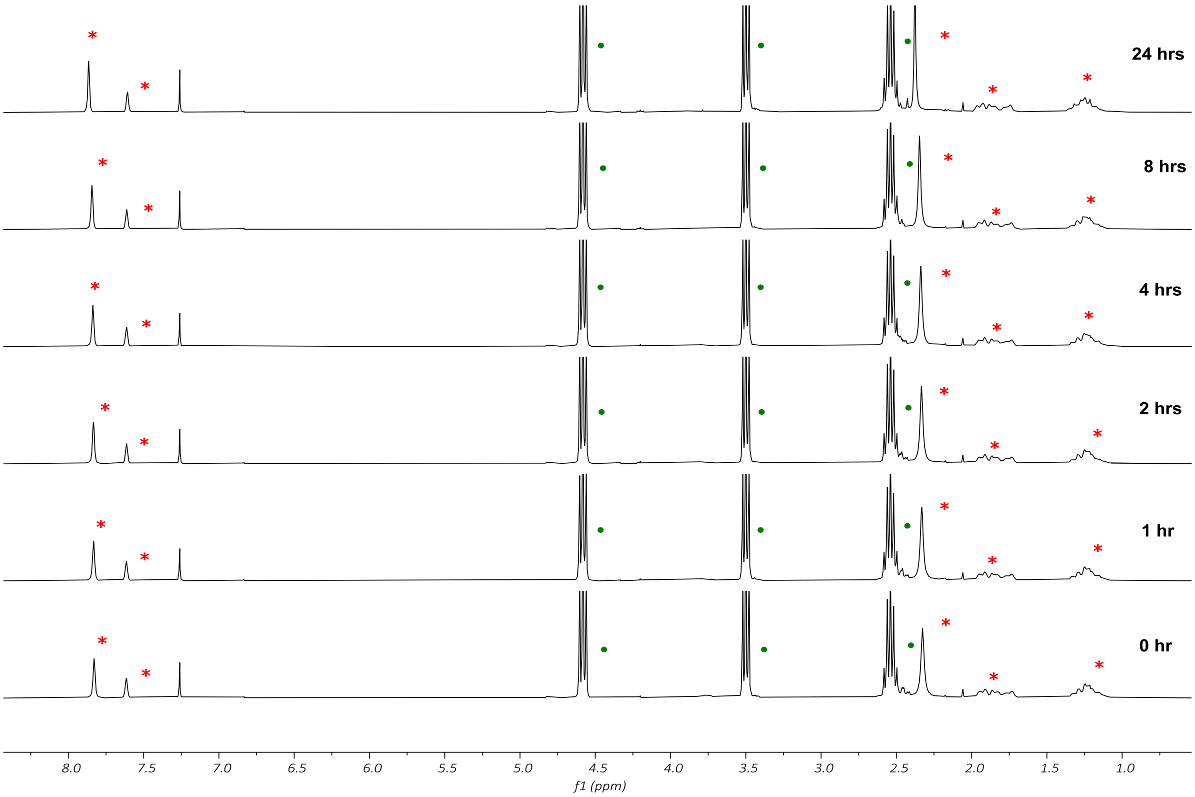

Figure S2. Control experiment w/o base.

Next, we performed reaction in presence of base:

Suspension of **C1** (8.3 mg, 0.02 mmol, 1 equiv.), **2a** (16.8 mg, 0.1 mmol, 5.0 equiv.), and K_2CO_3 (13.8 mg, 0.1 mmol, 1 equiv.) in $CDCl_3$ (0.7 ml) was stirred for indicated time at room temperature. ^{1}H NMR spectra were taken at different time points.

During that experiment (Figure S3), we observed significant decomposition of **C1** (*). After 24 hours, only traces of **C1** (*) were present in reaction mixture. Instead of **C1** formation of undefined by-product(s) (**X**) was observed.

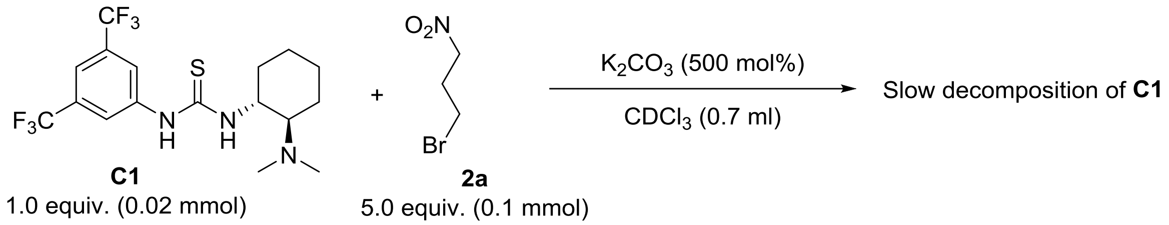

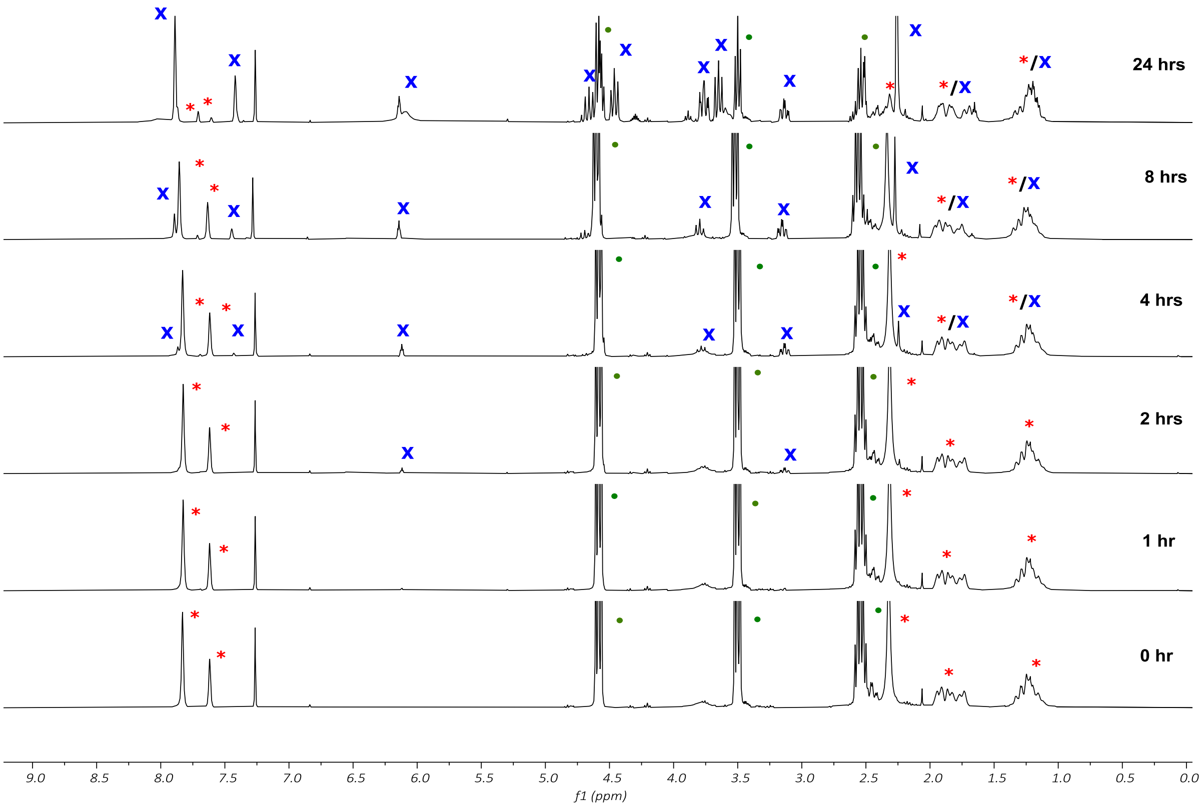

Figure S3. Control experiment with K_2CO_3.

Example of determination of diastereomeric ratio

The diastereomeric ratio between spirocycles **3a**/**4a´**/**4a** was determined from the crude reaction mixture (with the full conversion of methyleneindolinone **1**, the solvent was evaporated, and the crude product was directly analyzed by ^{1}H NMR).

CH_3 triplet of diastereomers **3** was located close to 1.1 ppm

CH_3 triplet of diastereomers **4** was located close to 0.7 ppm

CH_3 triplet of diastereomers **4´** was located close to 0.9 ppm

Additionally, CH_3 triplet of elimination products **5** was located close to 1.0 ppm

The absolute configuration of all prepared spirocycles **3** was determined by chemical correlation with **3a** with defined stereochemistry from X-ray.

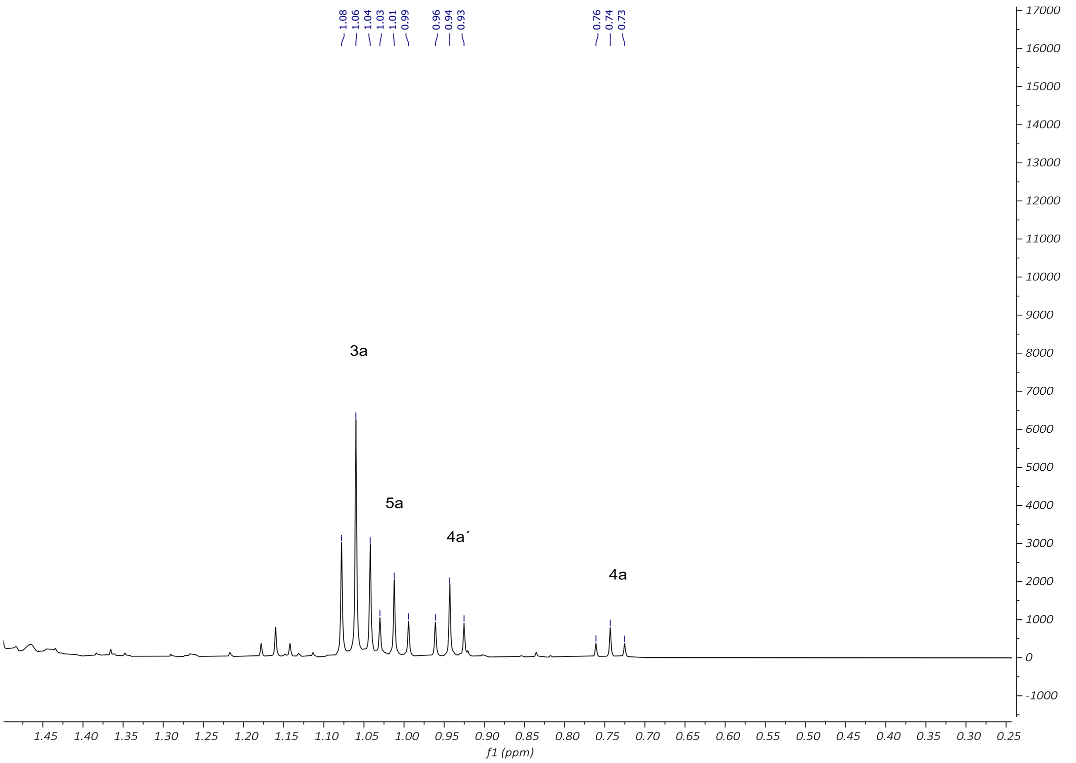

Figure S4. Highlighted ^{1}H NMR spectra of crude reaction mixture (Table S2, entry 7).

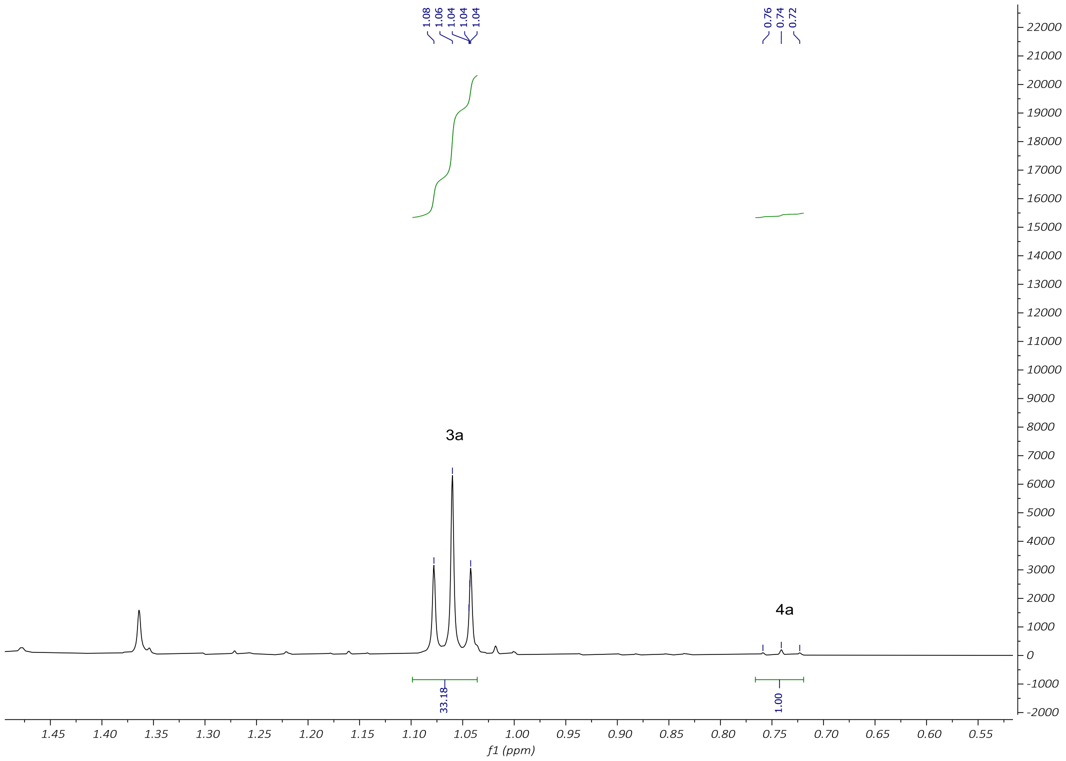

Figure S5. Highlighted ^{1}H NMR spectra of crude reaction mixture (Table S5, entry 7).

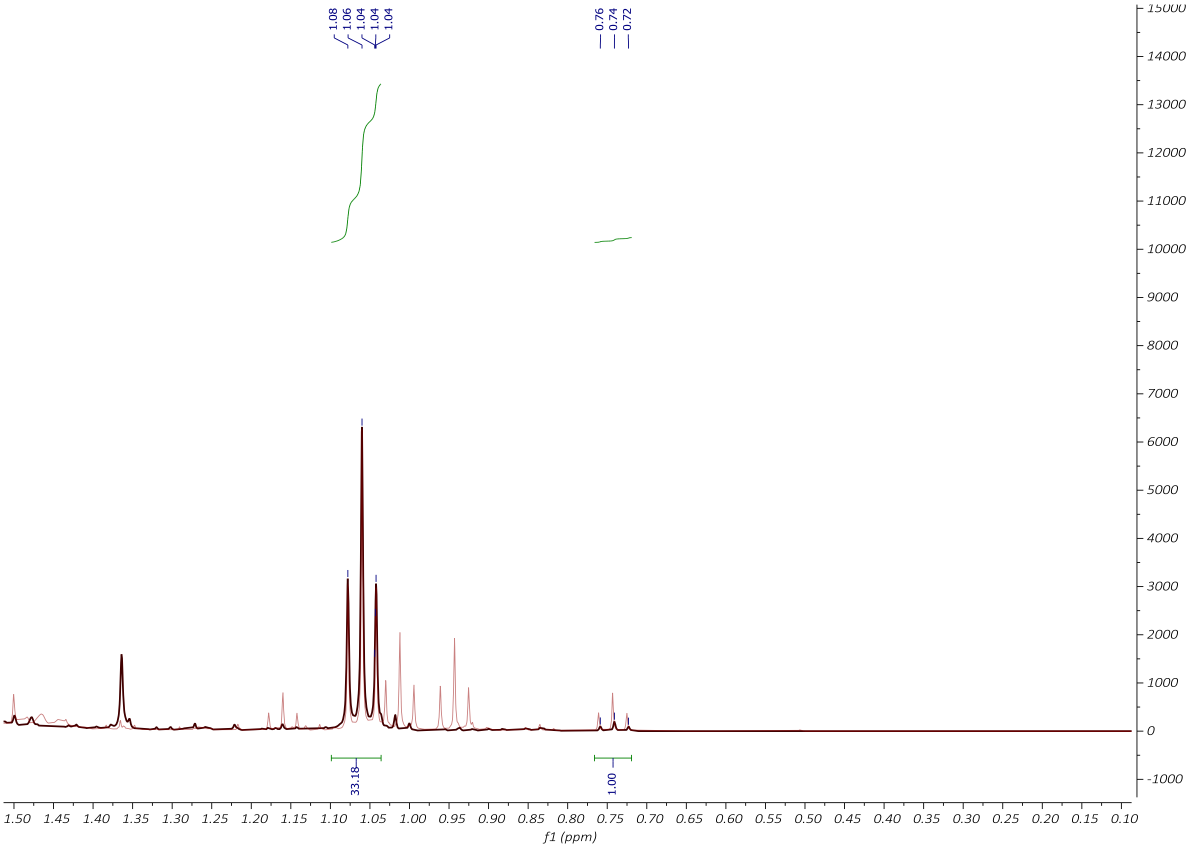

Figure S6. Superimposed ^{1}H NMR spectras of crude reaction mixtures (Table S5, entry 7 and Table S2, entry 7).

NMR yield determination

NMR yields were determined for compounds **4c** and **5i**. Those compounds were obtained as defined mixtures with elimination products **5**.

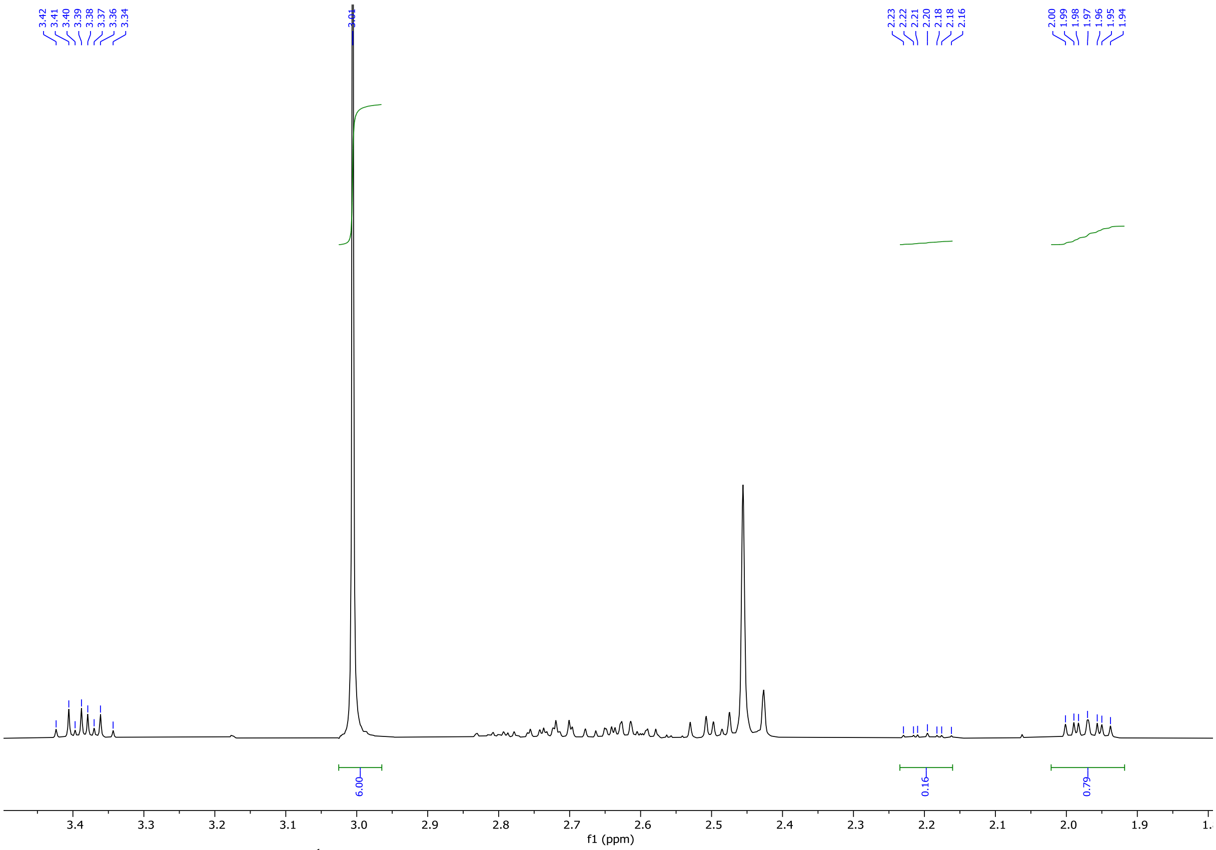

Figure S7. Highlighted ^{1}H NMR spectra used for NMR yield determination.

Table S6. NMR yield of **4c**/**5c**

	**4c**	**5c**	dimethylsulfone (IS)
Signal	1.97 (ddd, *J* = 13.2, 7.4, 4.8 Hz, 1H)	2.20 (ddd, *J* = 13.5, 8.1, 5.6 Hz, 1H)	2.98 (s, 6H)
Integral value	0.79	0.16	6.00
Calculated	0.0184 mmol, (18%)	0.0037 mmol, (4%)	0.0233 mmol (2.2 mg)

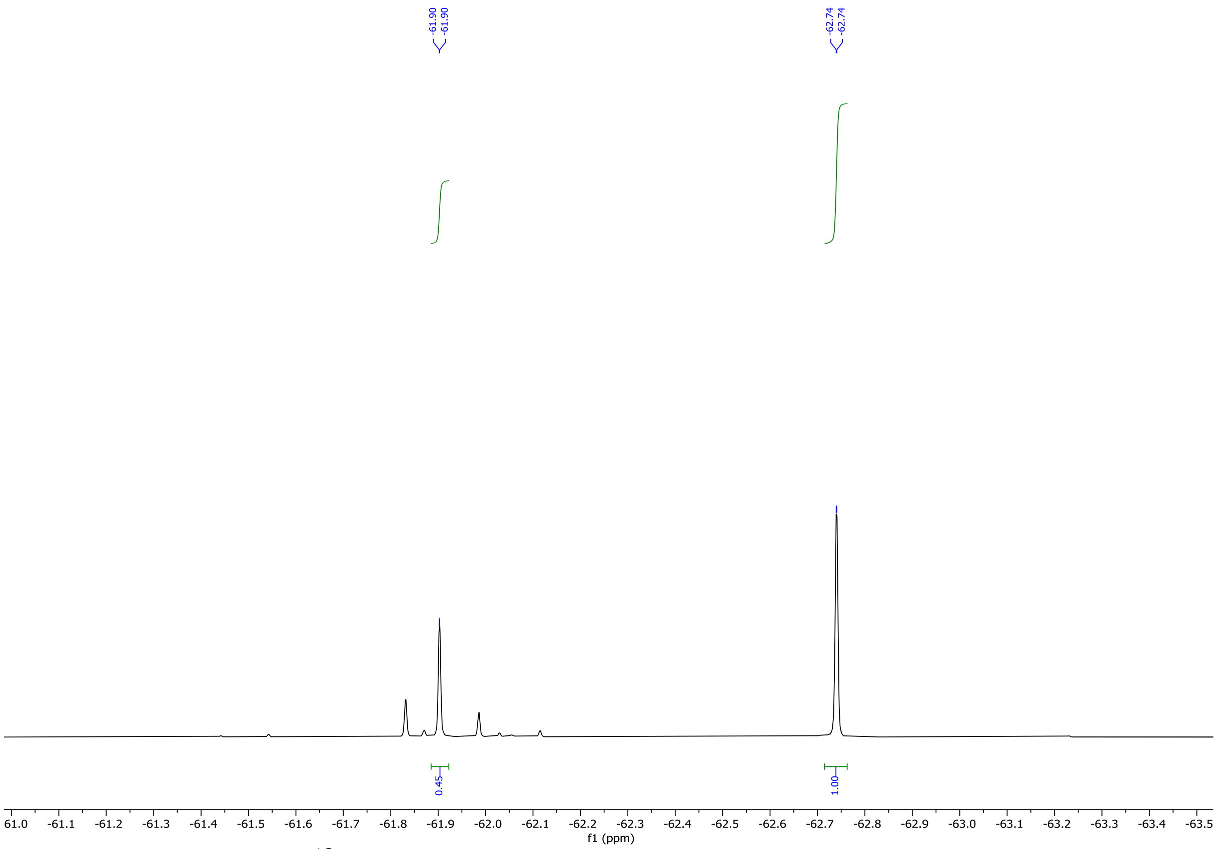

Figure S8. Highlighted ^{19}F NMR spectra used for NMR yield determination.

Table S7. NMR yield of **3i**.

	**3i**	*α*,*α*,*α*-trifluorotoluene (IS)
Signal	-61.9 C**<u>F</u>**$_3$	-62.7 C**<u>F</u>**$_3$
Integral value	0.45	1.00
Calculated	0.022 mmol (22 %)	0.0493 mmol (7.2 mg)

Relative configuration determination

The relative configurations of diastereomers **3l**/**4l** were determined by 1D NOE spectra of its isolated mixture (dr = 3/1). For major diastereomer (**3l**), there are significant cross-peaks between H4-H9, H9-H10, H9-H12β, and H10-H11α, H10-H11β. Based on that, we determined the relative configuration of **3l** as shown in Figure S9. This observation is supported by determined absolute configuration of **3a**, which is determined by the X-ray analysis (see below). For minor diastereomer (**4l**), there are cross-peaks between H4-H10, H4-H11α, H4-H12α, H9-H10, H9-H12β, and H10-H11α. Based on that, we determined the relative configuration of **4l** as shown in Figure S9.

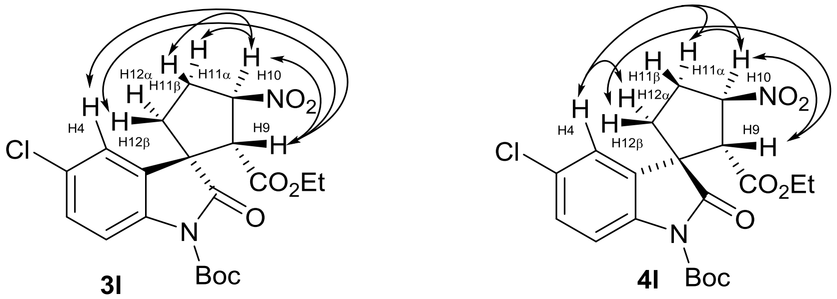

Figure S9. Highlighted crosspeaks for **3l** and **4l** in 1D NOESY

The relative configurations of diastereomers **3n**/**4n** were determined by 1D NOE spectra of its isolated mixture (dr = 4/1). For major diastereomer (**3n**), there are significant cross-peaks between H4-H9, H4-H12α, H9-H10, H9-H12β, H10-H11α, H10-H11β, H10-H12α. Based on that, we determined the relative configuration of **3n** as shown in Figure S10. For minor diastereomer (**4n**), there are cross-peaks between H4-H10, and H4-H11α, H9-H10, H10-H11α. Based on that, we determined the relative configuration of **4n** as shown in Figure S10.

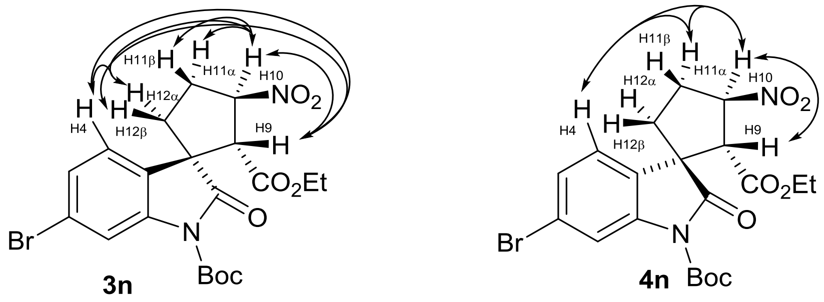

Figure S10. Highlighted crosspeaks for **3n** and **4n** in 1D NOESY

The relative configuration of major diastereomer **9** was determined by 1D NOE spectra. There is a significant NOE crosspeak among hydrogen atoms H9-H4. Based on the known configuration of spiro-carbon (analogy to **3a**), we determined the relative configuration of H9 **9** as shown in Figure S11.

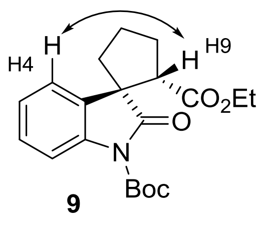

Figure S11. Highlighted crosspeaks for **9** 1D NOESY.

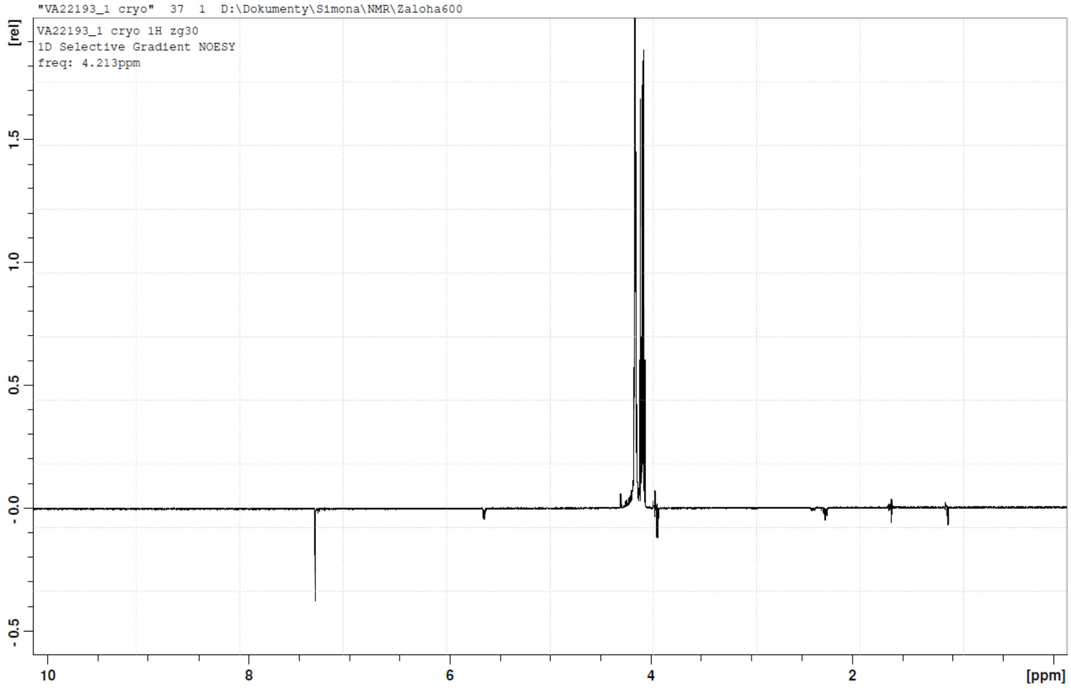

Figure S12. 1D Selective Gradient NOESY spectrum (*δ* = 4.21 ppm) of **3l**/**4l**.

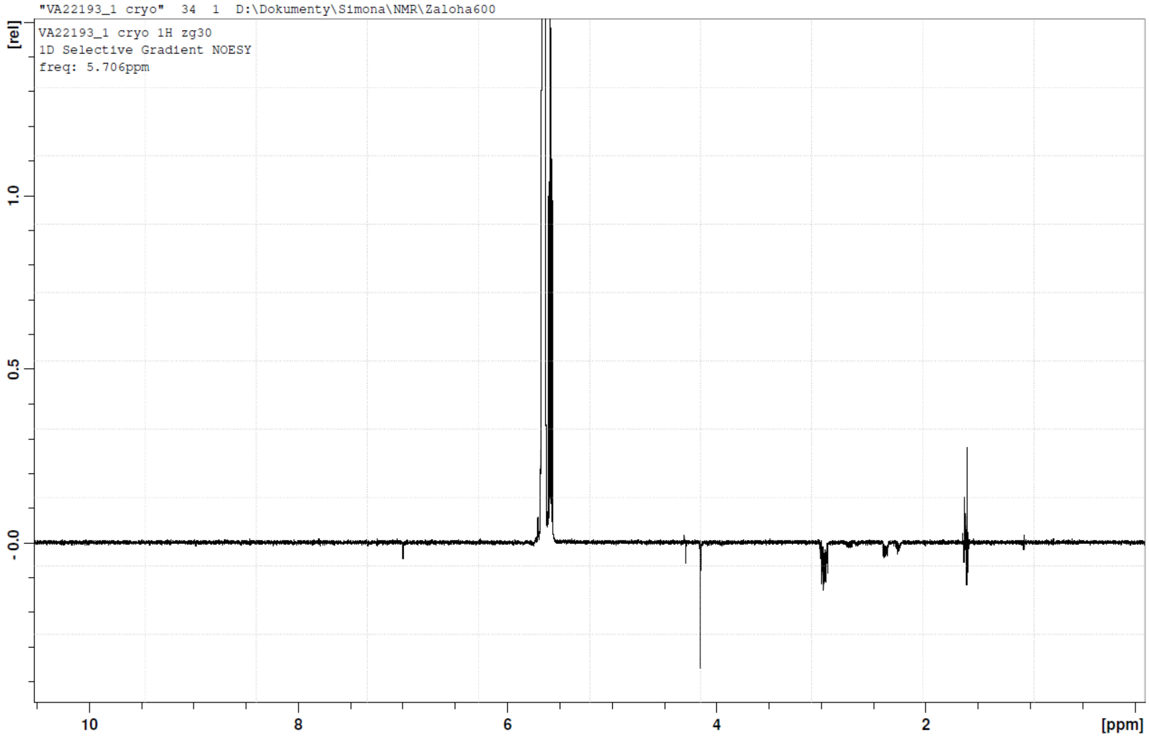

Figure S13. 1D Selective Gradient NOESY spectrum (*δ* = 5.71 ppm) of **3l**/**4l**.

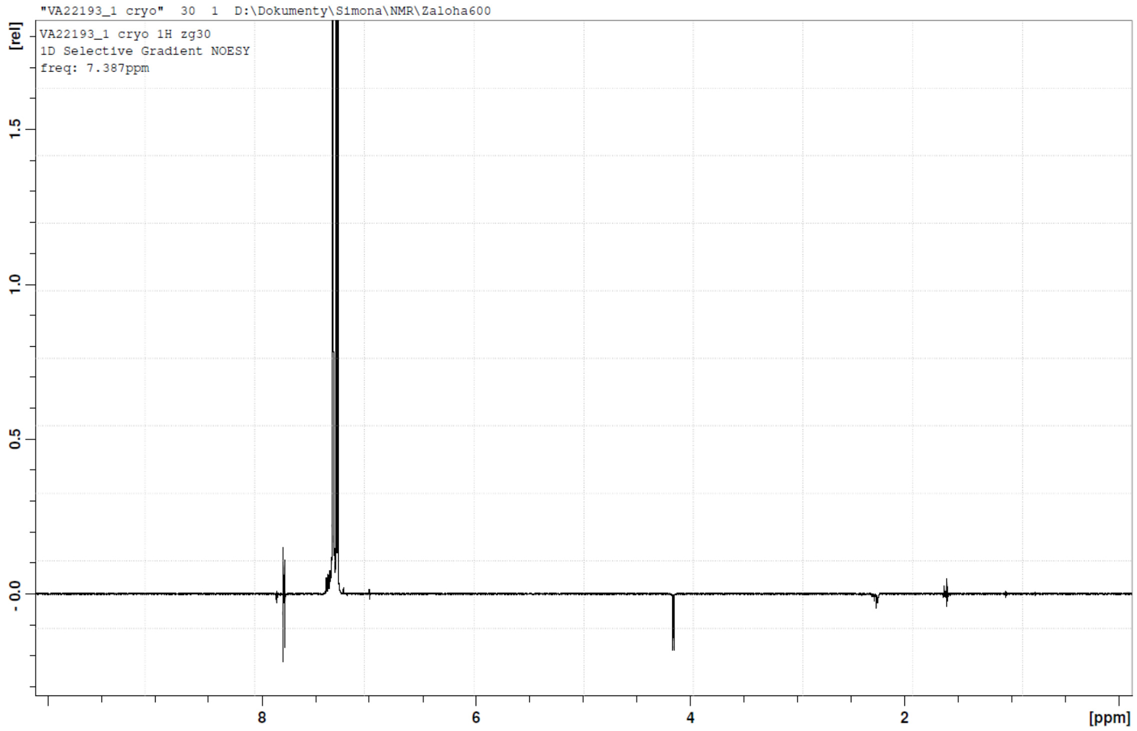

Figure S14. 1D Selective Gradient NOESY spectrum (δ = 7.39 ppm) of **3l**/**4l**.

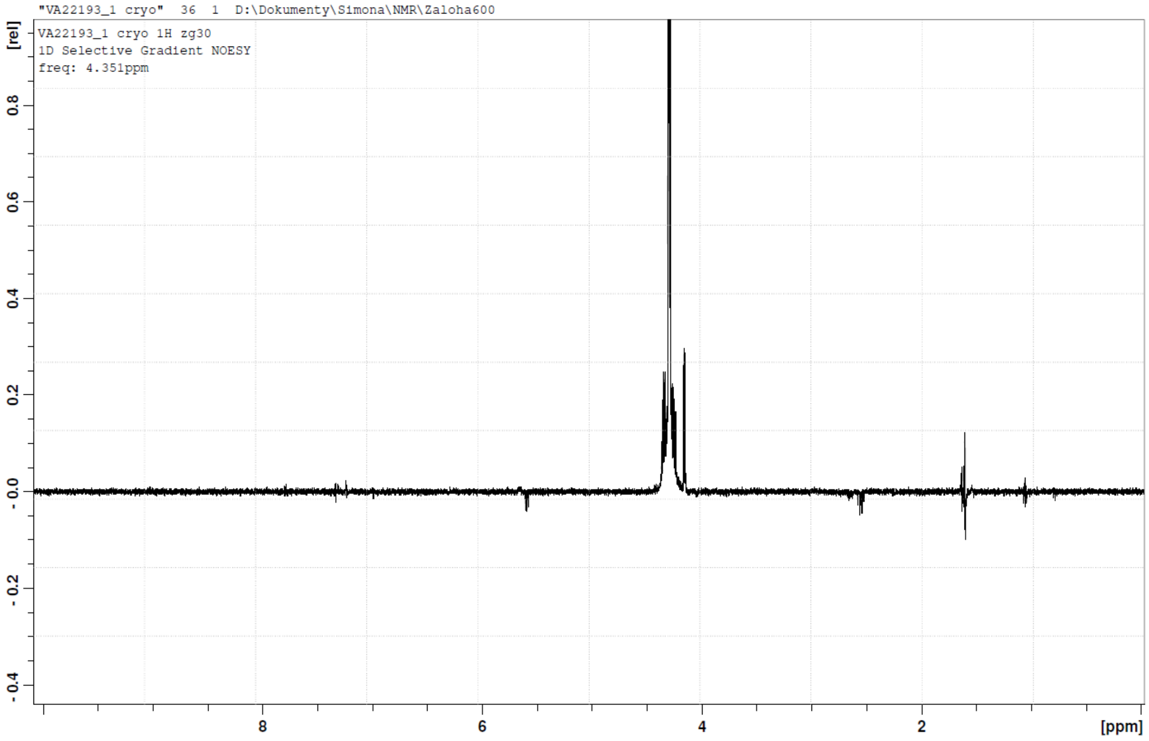

Figure S15. 1D Selective Gradient NOESY spectrum (δ = 4.35 ppm) of **3l**/**4l**.

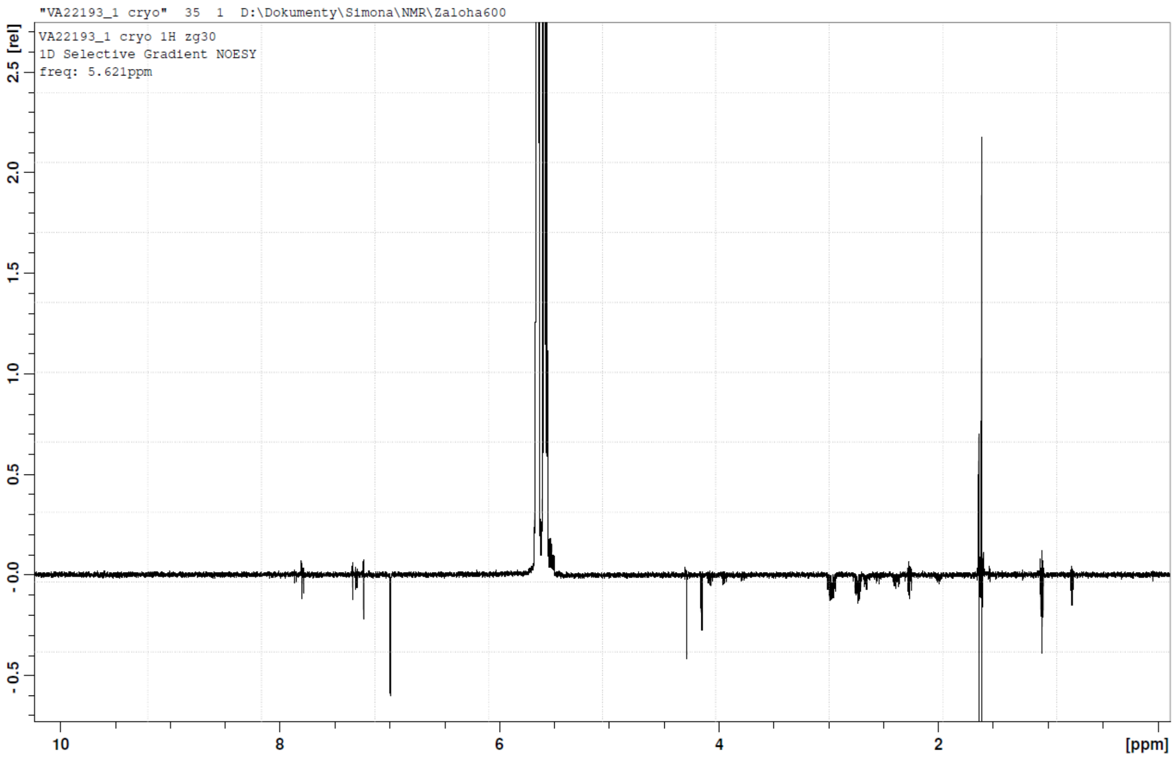

Figure S16. 1D Selective Gradient NOESY spectrum (δ = 5.62 ppm) of **3l**/**4l**.

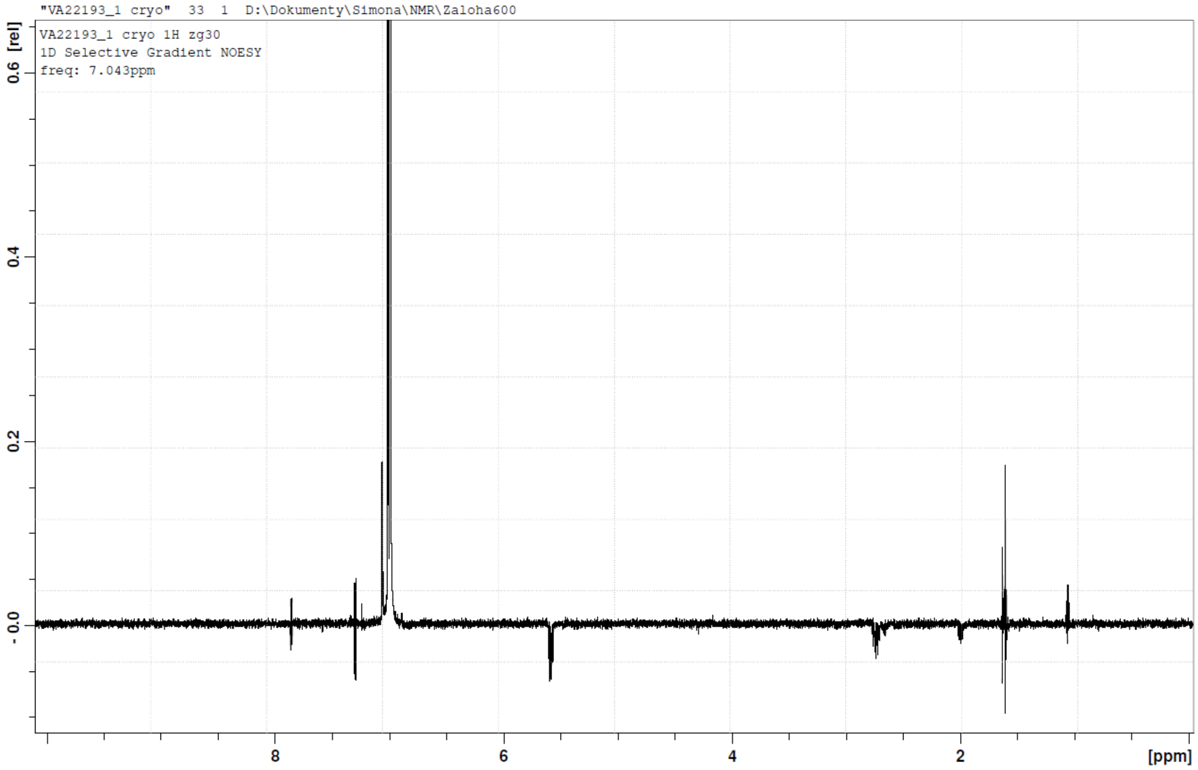

Figure S17. 1D Selective Gradient NOESY spectrum (δ = 7.04 ppm) of **3l**/**4l**.

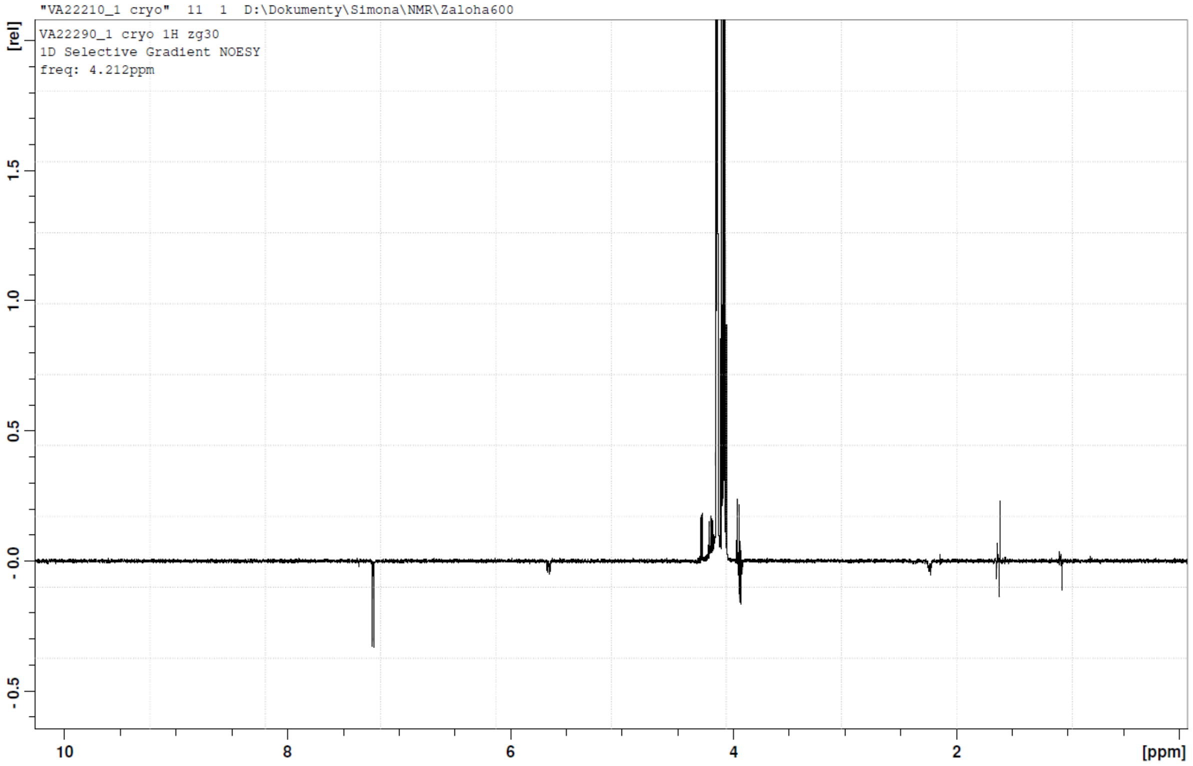

Figure S18. 1D Selective Gradient NOESY spectrum (δ = 4.21 ppm) of **3n**/**4n**.

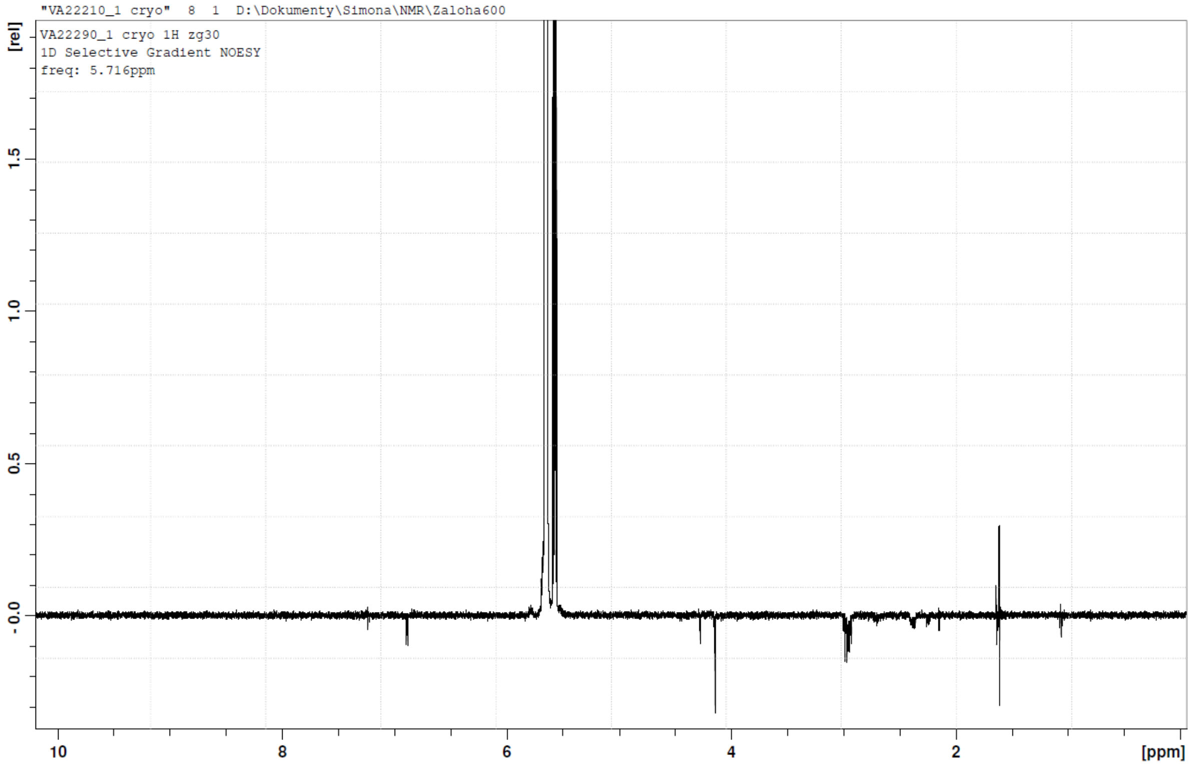

Figure S19. 1D Selective Gradient NOESY spectrum (δ = 5.72 ppm) of **3n**/**4n**.

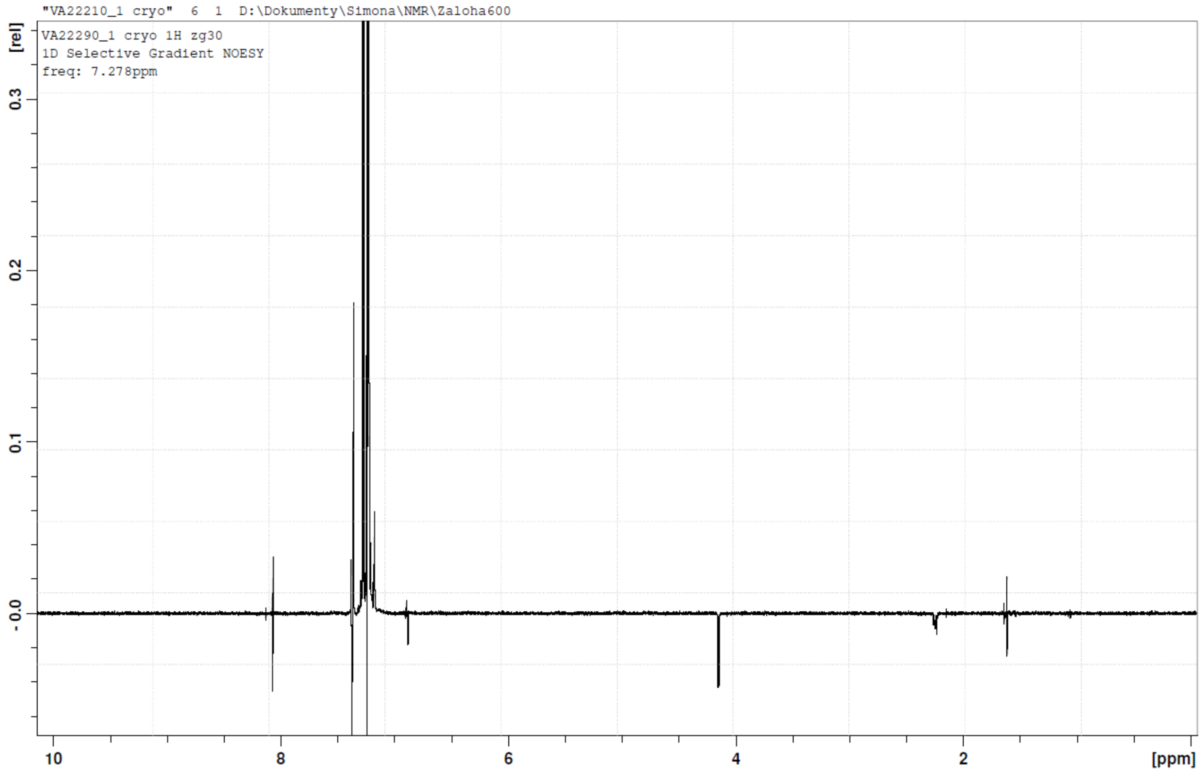

Figure S20. 1D Selective Gradient NOESY spectrum (δ = 7.28 ppm) of **3n**/**4n**.

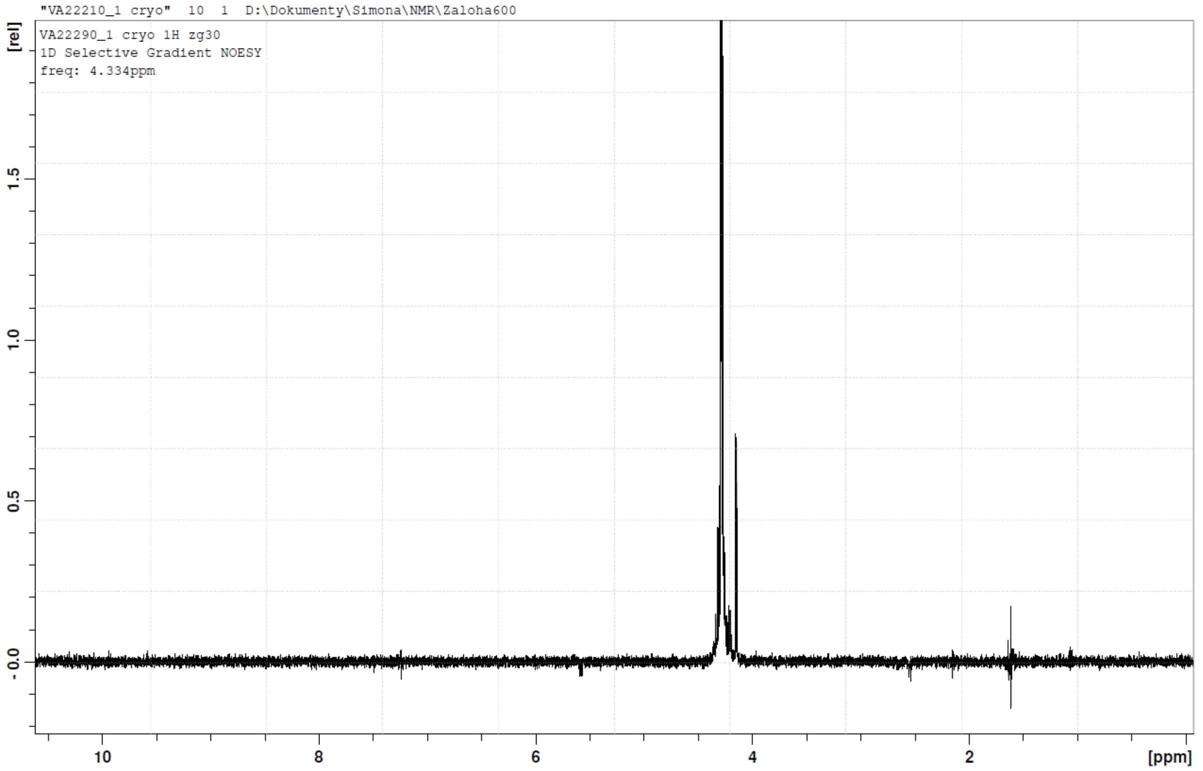

Figure S21. 1D Selective Gradient NOESY spectrum (δ = 4.33 ppm) of **3n**/**4n**.

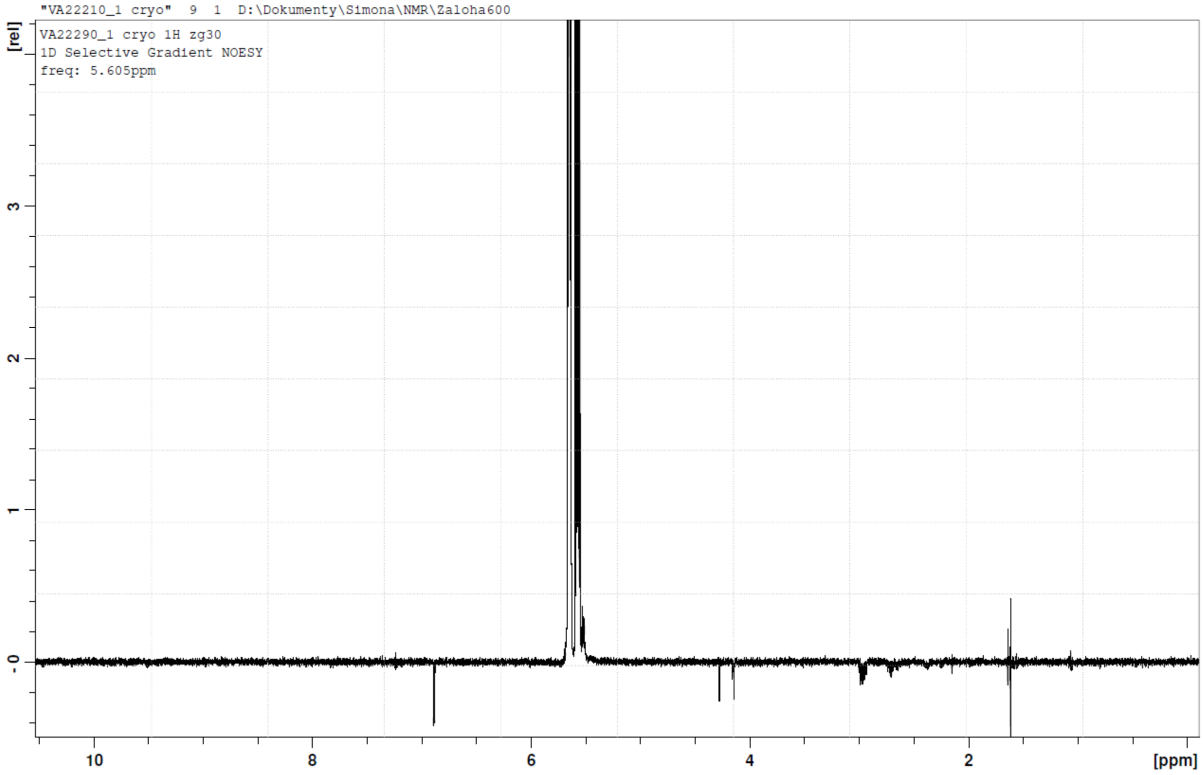

Figure S22. 1D Selective Gradient NOESY spectrum (δ = 5.61 ppm) of **3n**/**4n**.

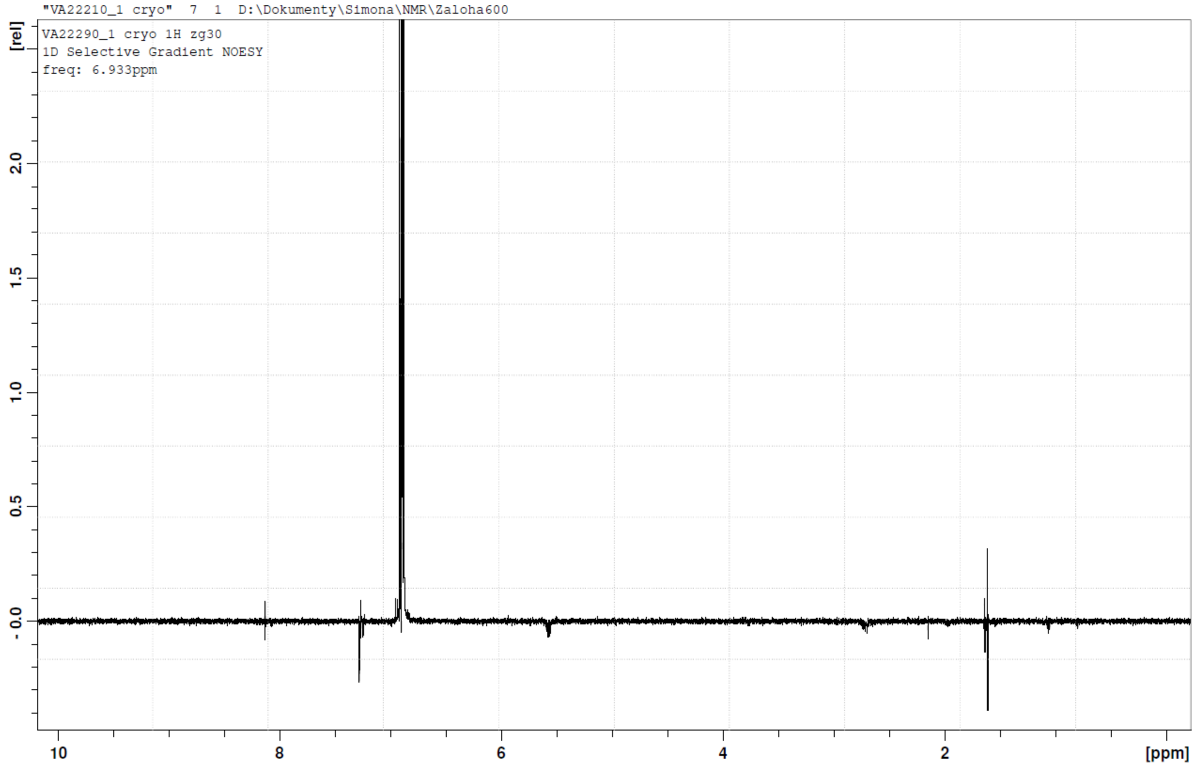

Figure S23. 1D Selective Gradient NOESY spectrum (δ = 6.93 ppm) of **3n**/**4n**.

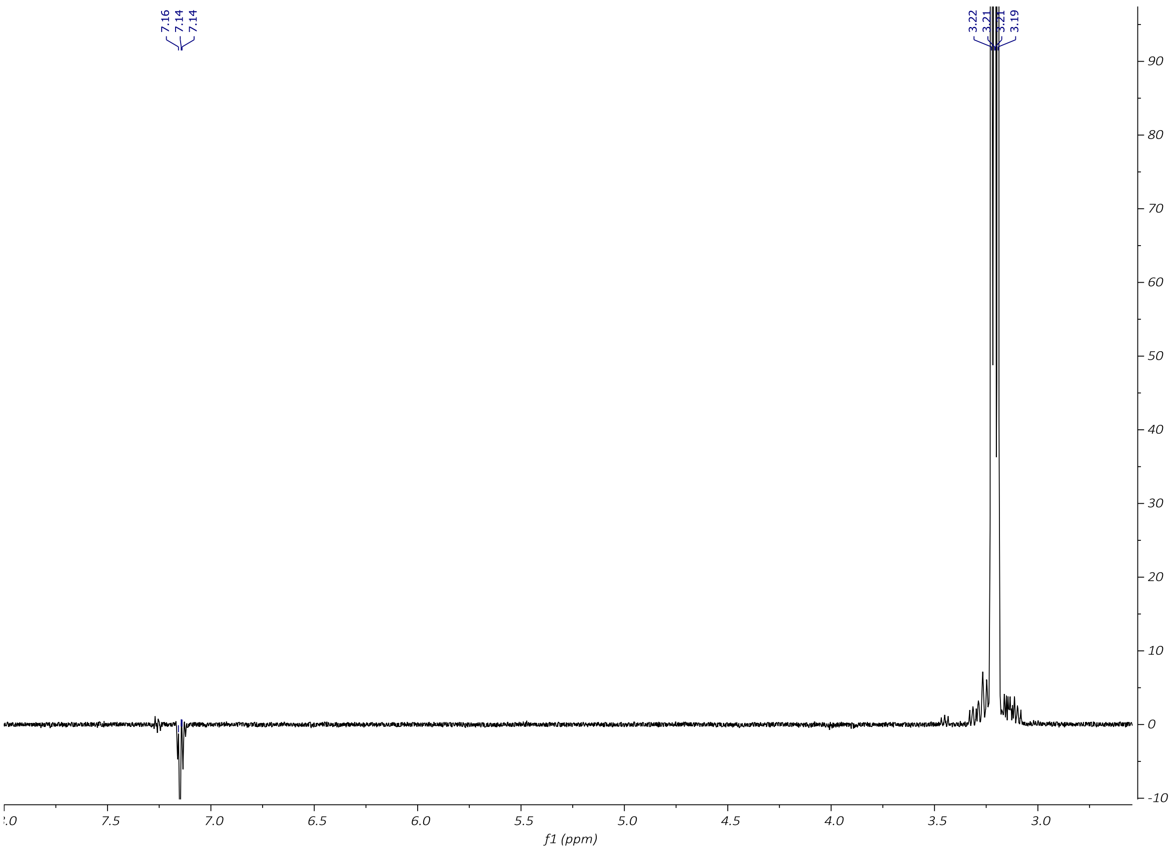

Figure S24. 1D Selective Gradient NOESY spectrum (δ = 3.21 ppm) of **9/9´.**

Crystallographic data

The diffraction experiments for and **3a** was performed on Bruker D8 VENTURE Kappa Duo PHOTON III by IμS micro-focus sealed tube with CuKα (λ= 1.54178) for **3a** radiation at a temperature 120(2)K. The structures were solved by direct methods (XT[1]) and refined by full matrix least squares based on F^2 (SHELXL2018[2]). The hydrogen atoms on carbon were fixed into idealized positions (riding model) and assigned temperature factors either $H_{iso}(H) = 1.2$ U_{eq}(pivot atom) or $H_{iso}(H) = 1.5$ U_{eq} (pivot atom) for methyl moiety. The absolute structure determination[40] was based on anomalous dispersion of heavy atoms.

Crystal data for **3a**: $C_{20}H_{24}N_2O_7$, M_r = 404. 41, Orthorhombic, $P2_1\ 2_1\ 2_1$ (No 19), a = 9.6207 (4) Å, b = 9.6373 (4) Å, c = 21.6515 (9) Å, V = 2007.47 (14) Å^3, Z = 4, D_x = 1.338 Mg m^{-3}, colourles prism of dimensions 0.13 × 0.12 × 0.09 mm, multi-scan absorption correction (μ = 0.85 mm^{-1}) T_{min} = 0.87, T_{max} = 0.93; a total of 32126 measured reflections (θ_{max}= 77.5°), from which 4207 were unique (R_{int} = 0.030) and 4137 observed according to the $I > 2\sigma(I)$ criterion. The refinement converged ($\Delta/\sigma_{max} \leq 0.001$) to R = 0.037 for observed reflections and $wR(F^2)$ = 0.097, GOF = 1.05 for 266 parameters and all 4207 reflections. The final difference map displayed no peaks of chemical significance ($\Delta\rho_{max}$ = 0.77, $\Delta\rho_{min}$ −0.26 e.Å^{-3}). Absolute structure parameter: 0.01 (4).

X-ray crystallographic data have been deposited with the Cambridge Crystallographic Data Centre (CCDC) under deposition number 2212693 and can be obtained free of charge from the Centre via its website (www.ccdc.cam.ac.uk/getstructures).

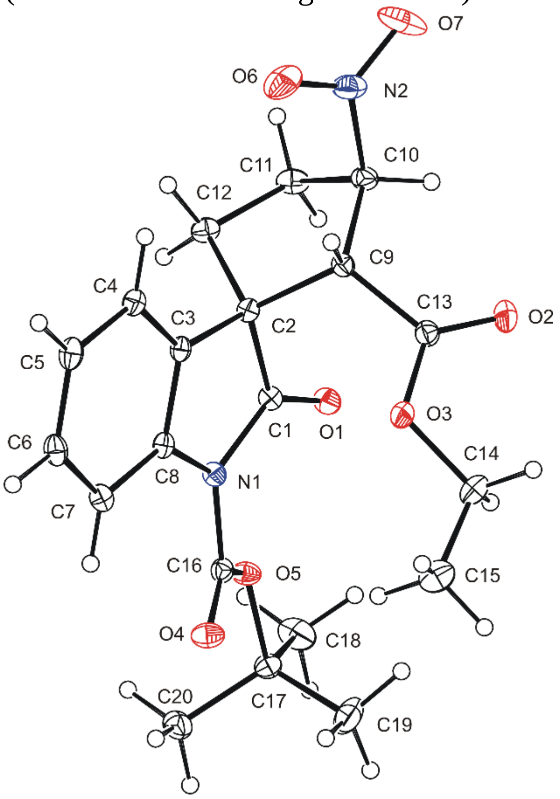

Figure. S25. View on the molecule of **3a** with atom numbering. The displacement ellipsoids are drawn on 30% probability level. The streochemical descriptors are: *R*,*R*,*R* on C2, C9, C10, respectively.

NMR spectra

tert-Butyl (*E*)-3-(2-ethoxy-2-oxoethylidene)-2-oxo-5-(trifluoromethyl)indoline-1-carboxylate (1i)

^{1}H NMR of **1i** (400 MHz, $CDCl_3$)

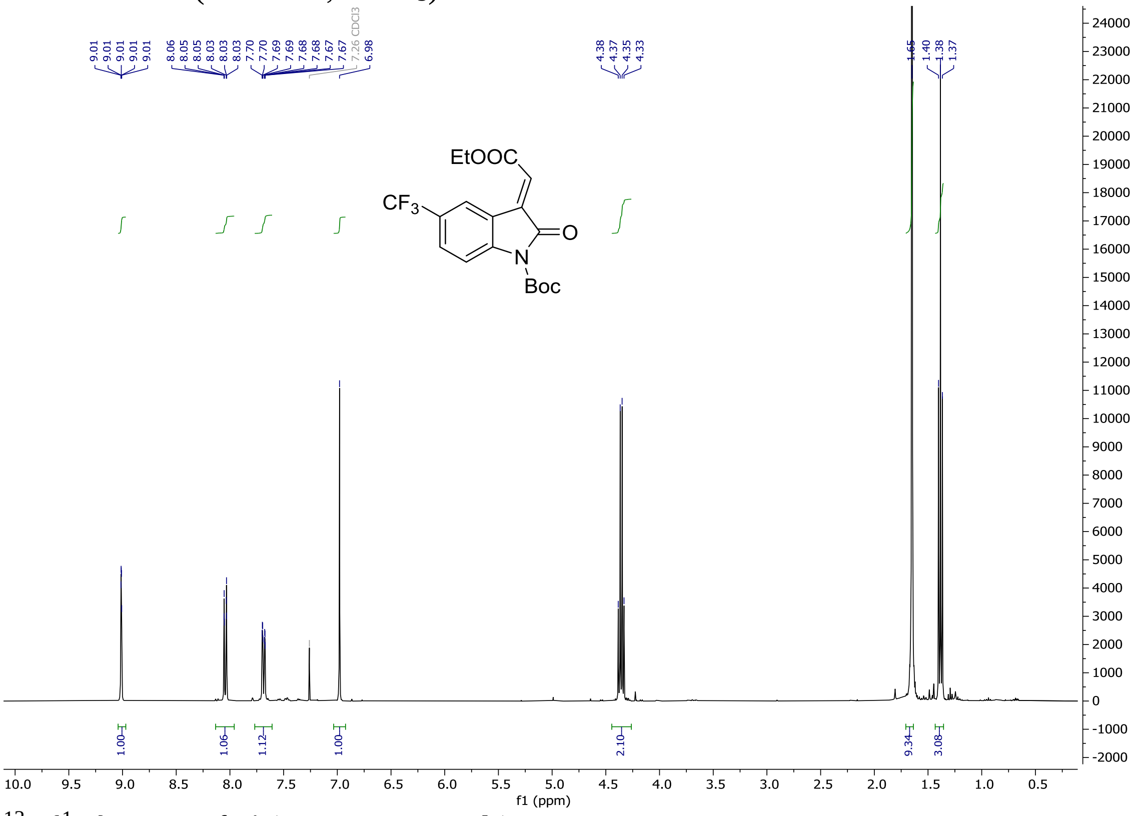

^{13}C{^{1}H} NMR of **1i** (101 MHz, $CDCl_3$)

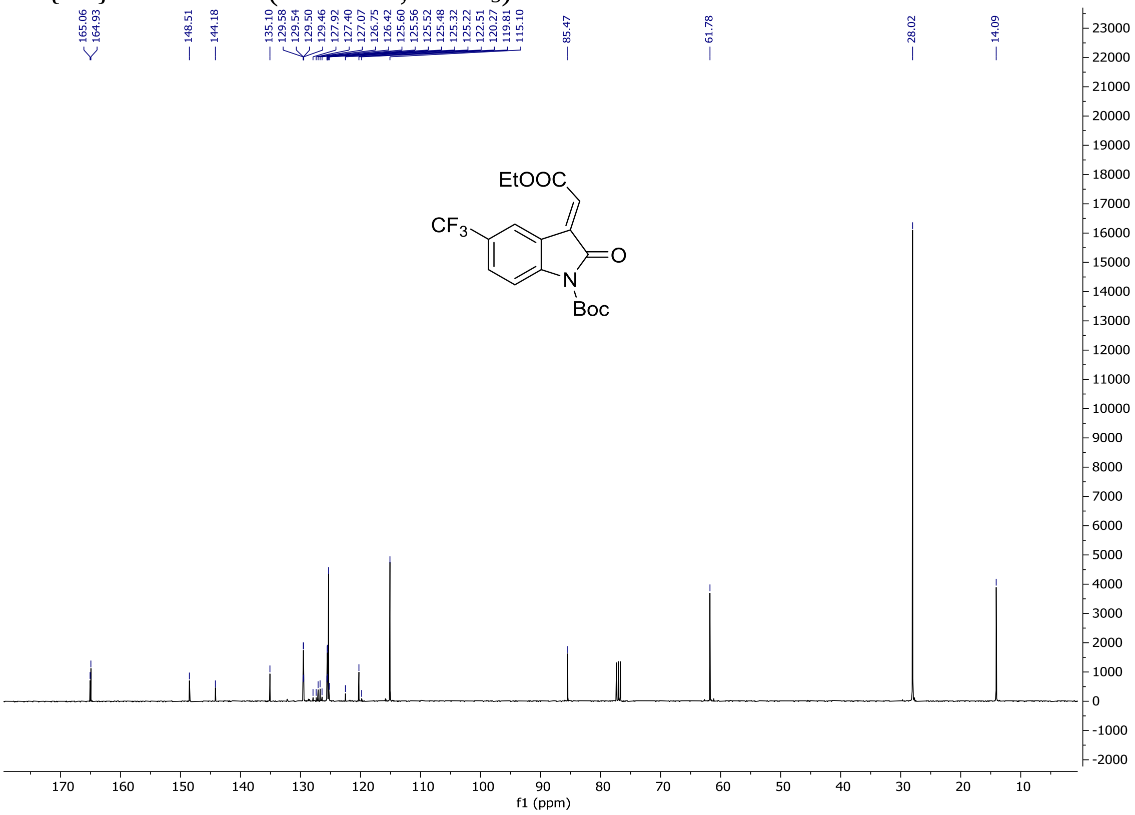

^{19}F NMR of **1i** (376 MHz MHz, $CDCl_3$)

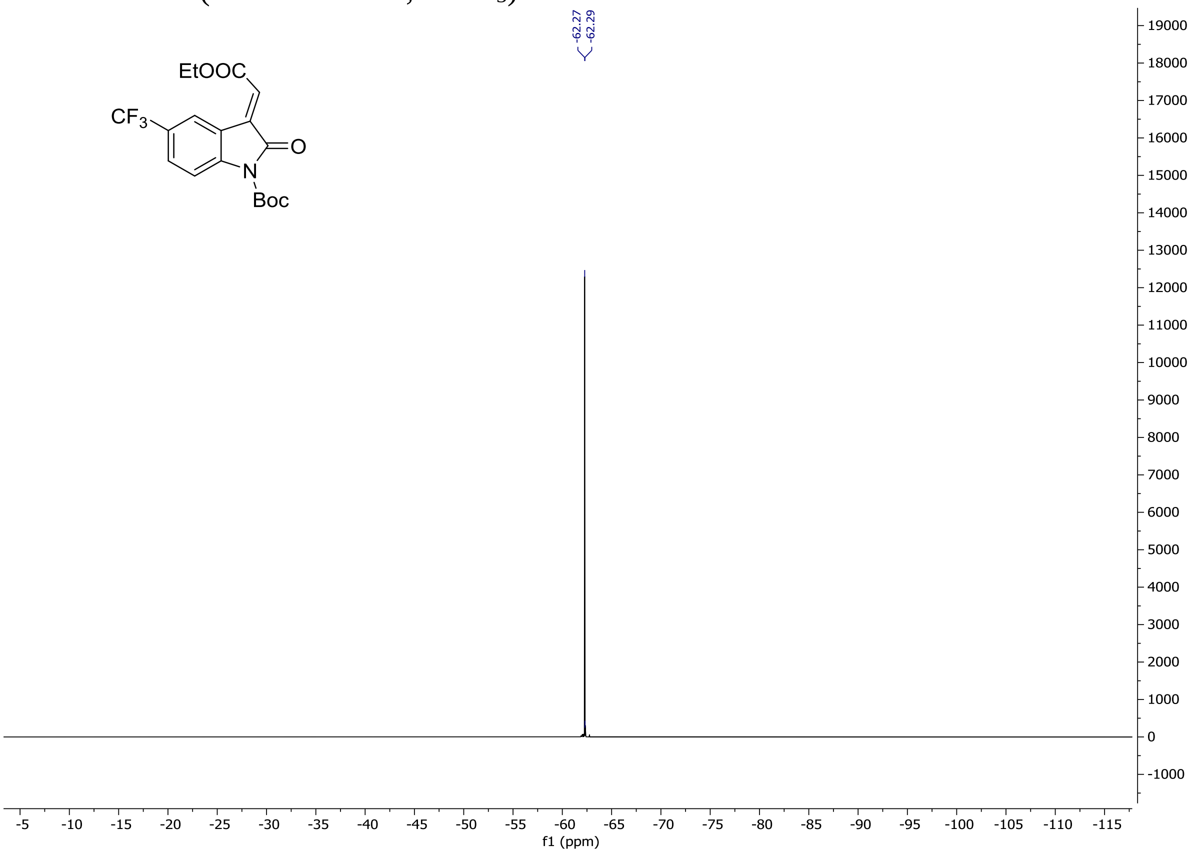

1-Bromo-3-nitropropane (2a)

^{1}H NMR of **2a** (400 MHz, $CDCl_3$)

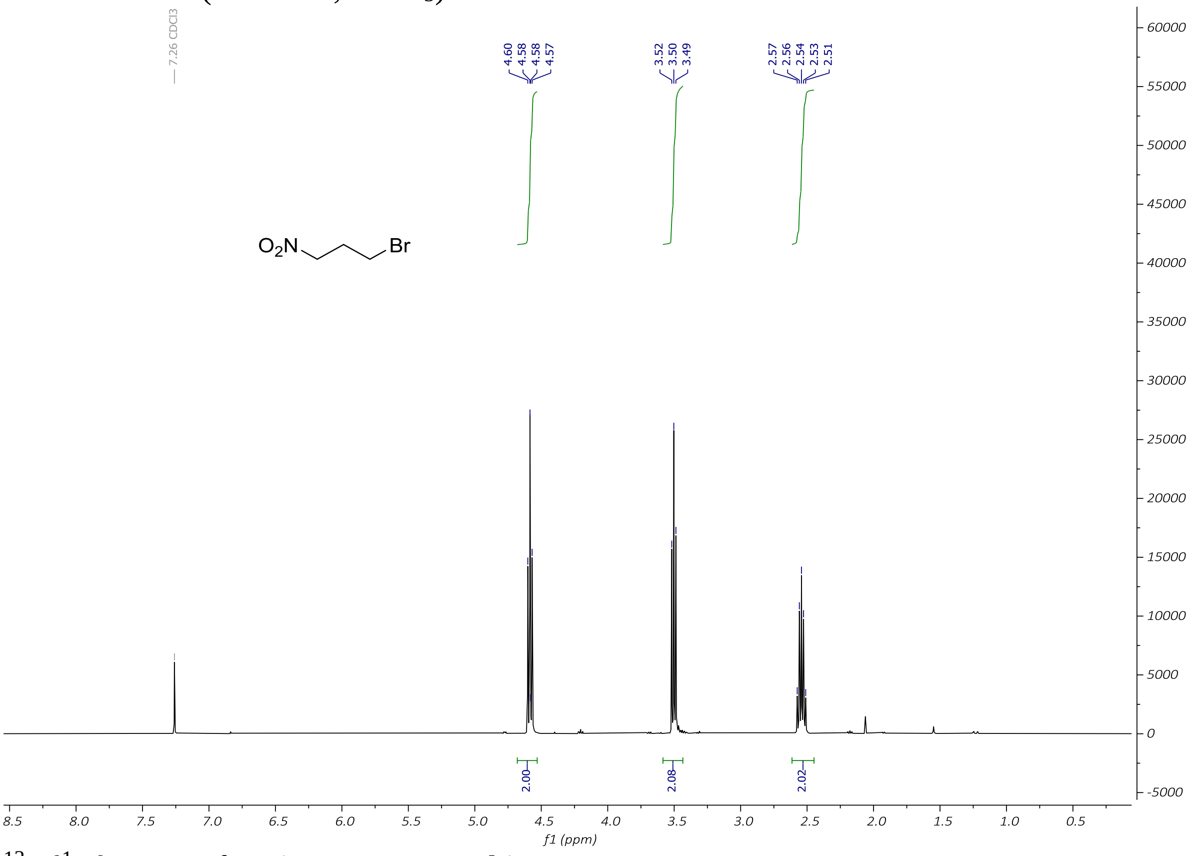

^{13}C{^{1}H} NMR of **2a** (101 MHz, $CDCl_3$)

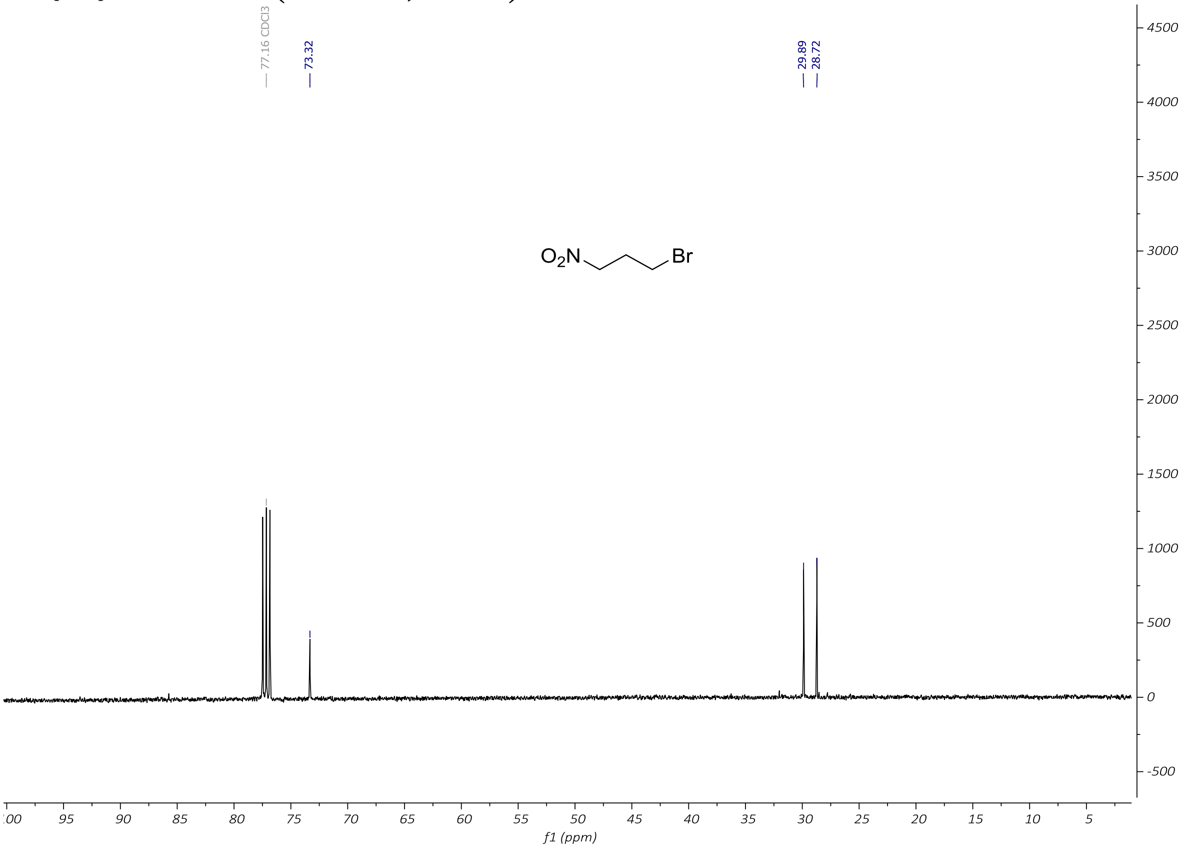

1-Bromo-2-nitroethane (2c)

^{1}H NMR of **2c** (400 MHz, $CDCl_3$)

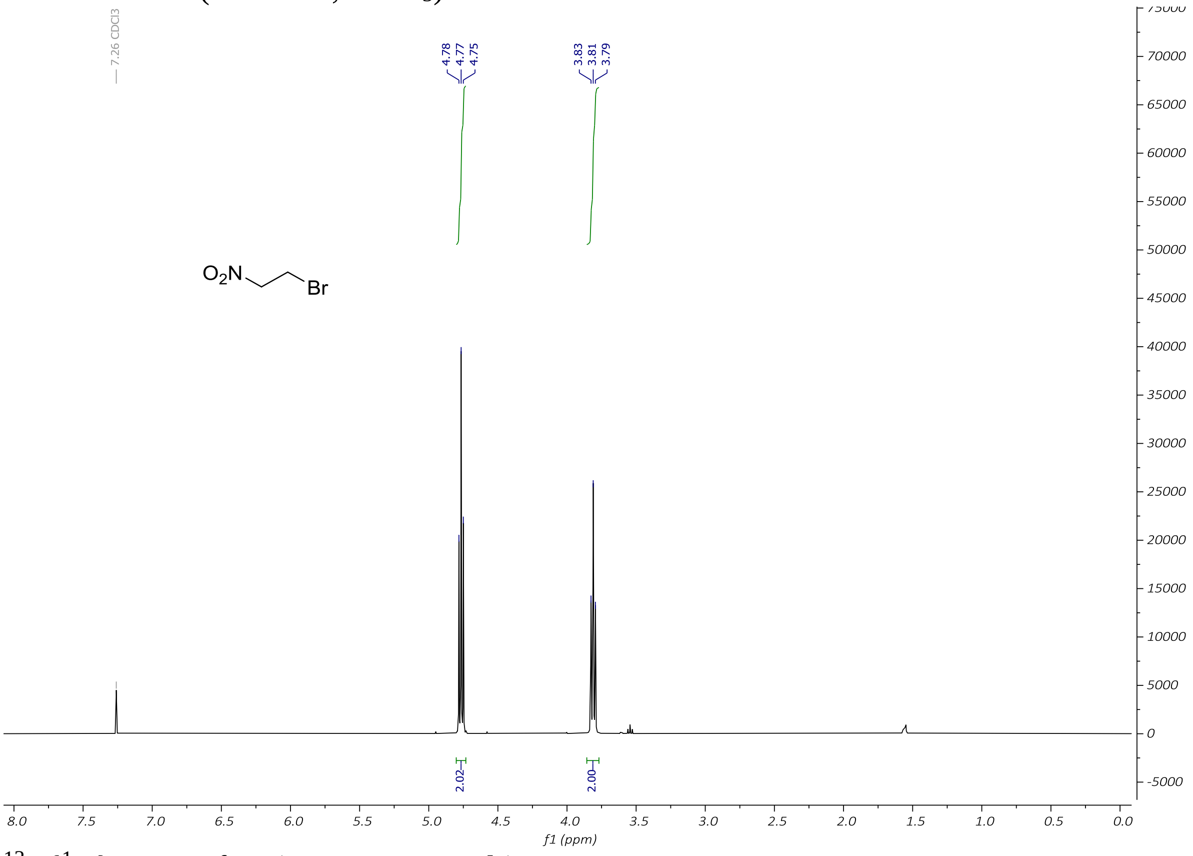

^{13}C{^{1}H} NMR of **2c** (101 MHz, $CDCl_3$)

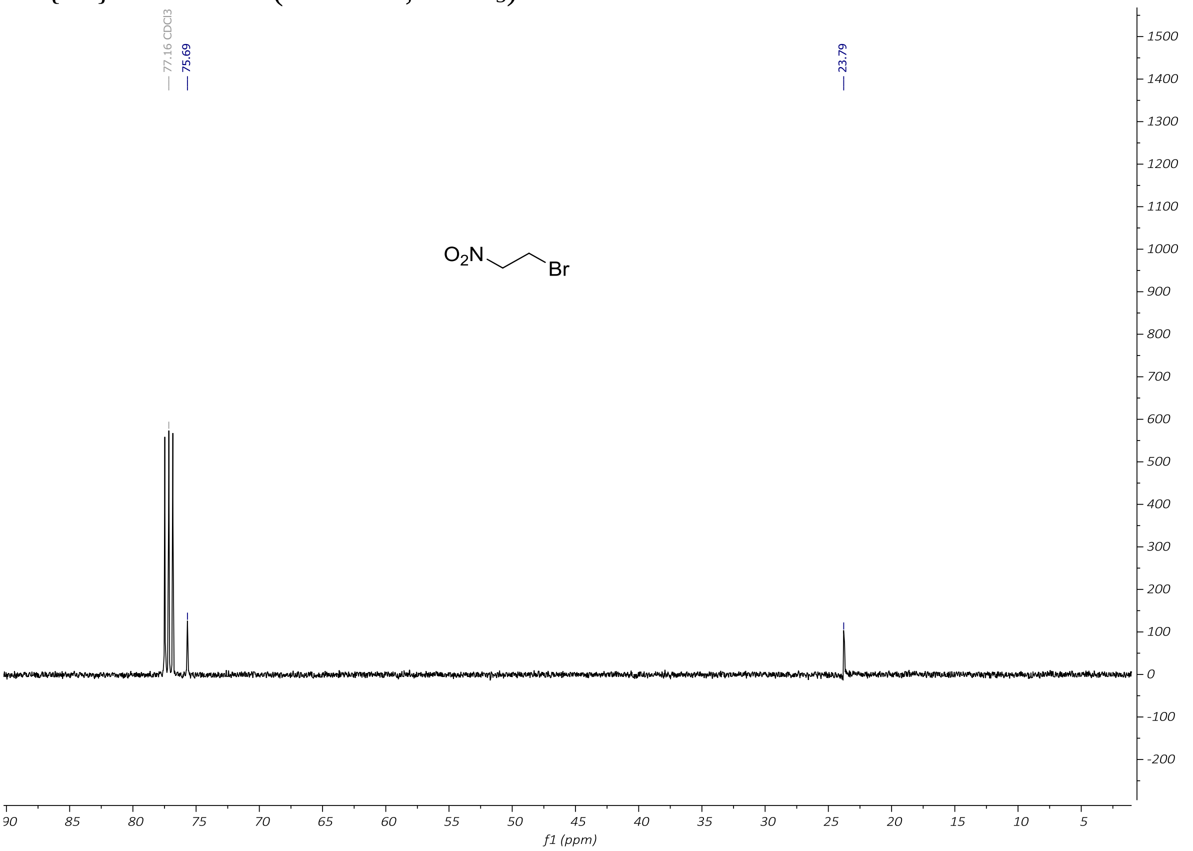

1'-(*tert*-Butyl) 2-ethyl (1*R*,2*R*,3*R*)-3-nitro-2'-oxospiro[cyclopentane-1,3'-indoline]-1',2-dicarboxylate (3a)

^{1}H NMR of **3a** (400 MHz, $CDCl_3$)

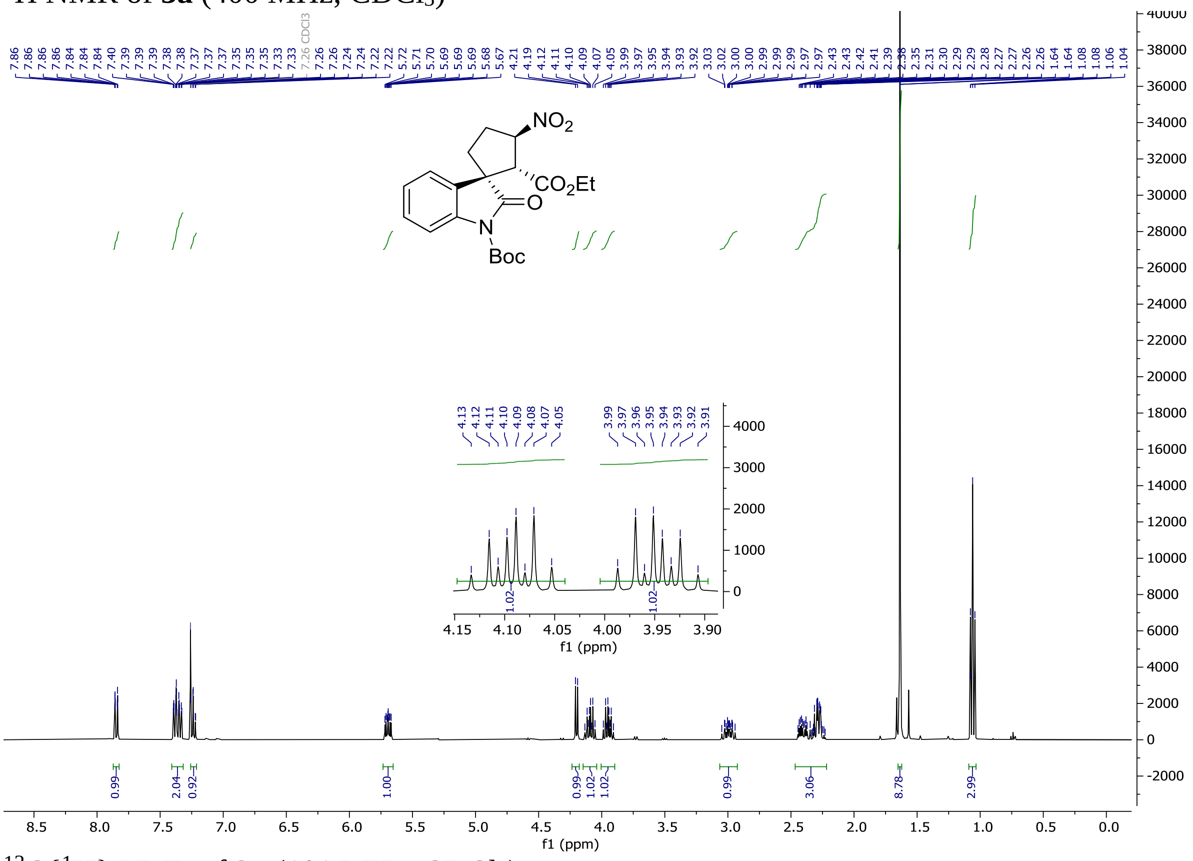

^{13}C{^{1}H} NMR of **3a** (101 MHz, $CDCl_3$)

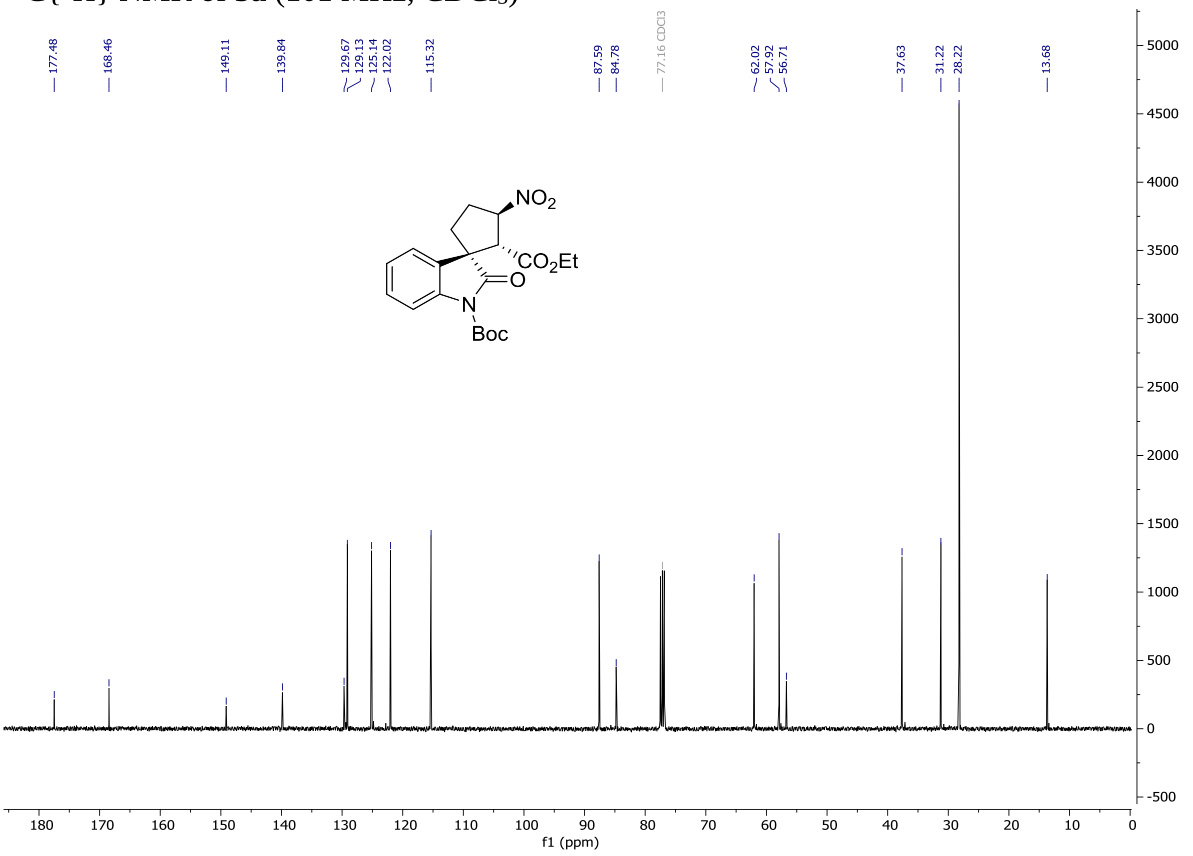

Ethyl (1*R*,2*R*,3*R*)-3-nitro-2'-oxo-1'-(2-oxo-2-phenyl-1λ^2-ethyl)spiro[cyclopentane-1,3'-indoline]-2-carboxylate (3b)

^{1}H NMR of **3b** (400 MHz, $CDCl_3$)

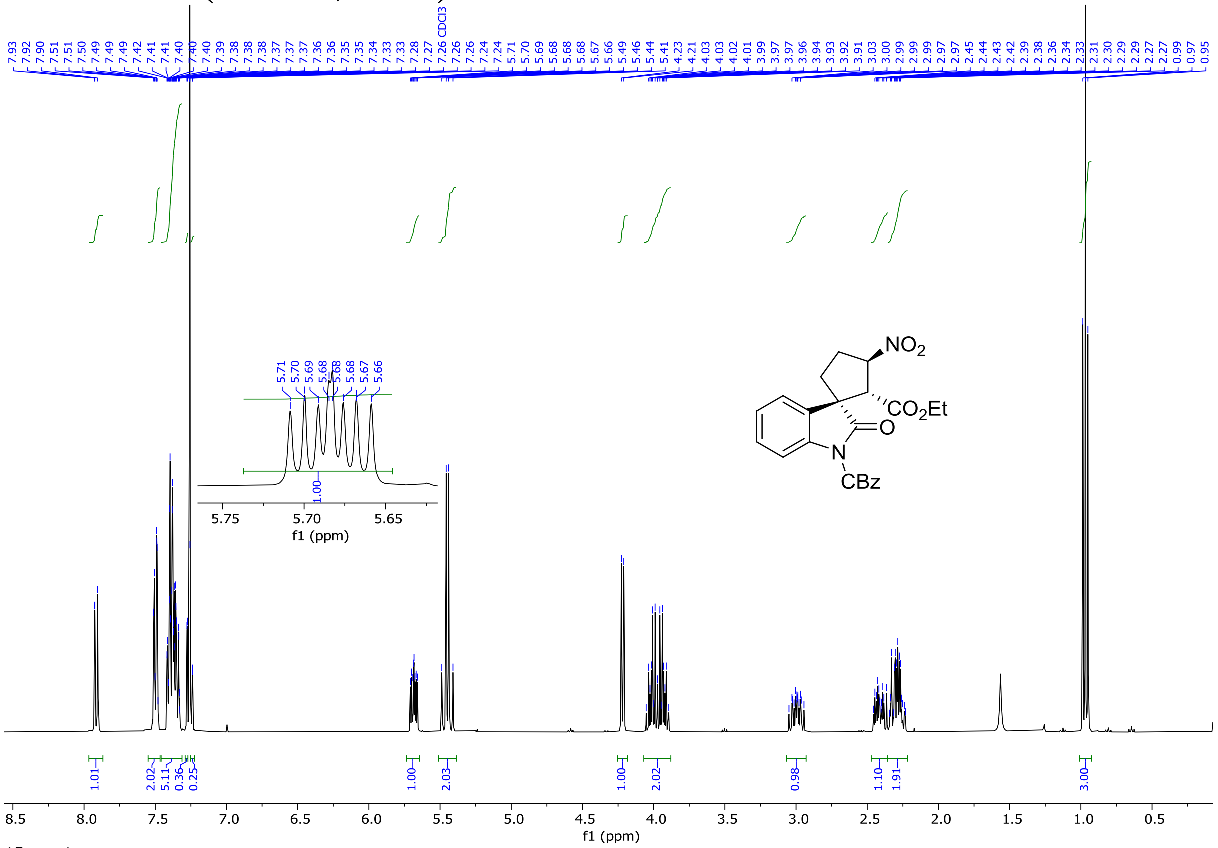

^{13}C{^{1}H} NMR of **3b** (101 MHz, $CDCl_3$)

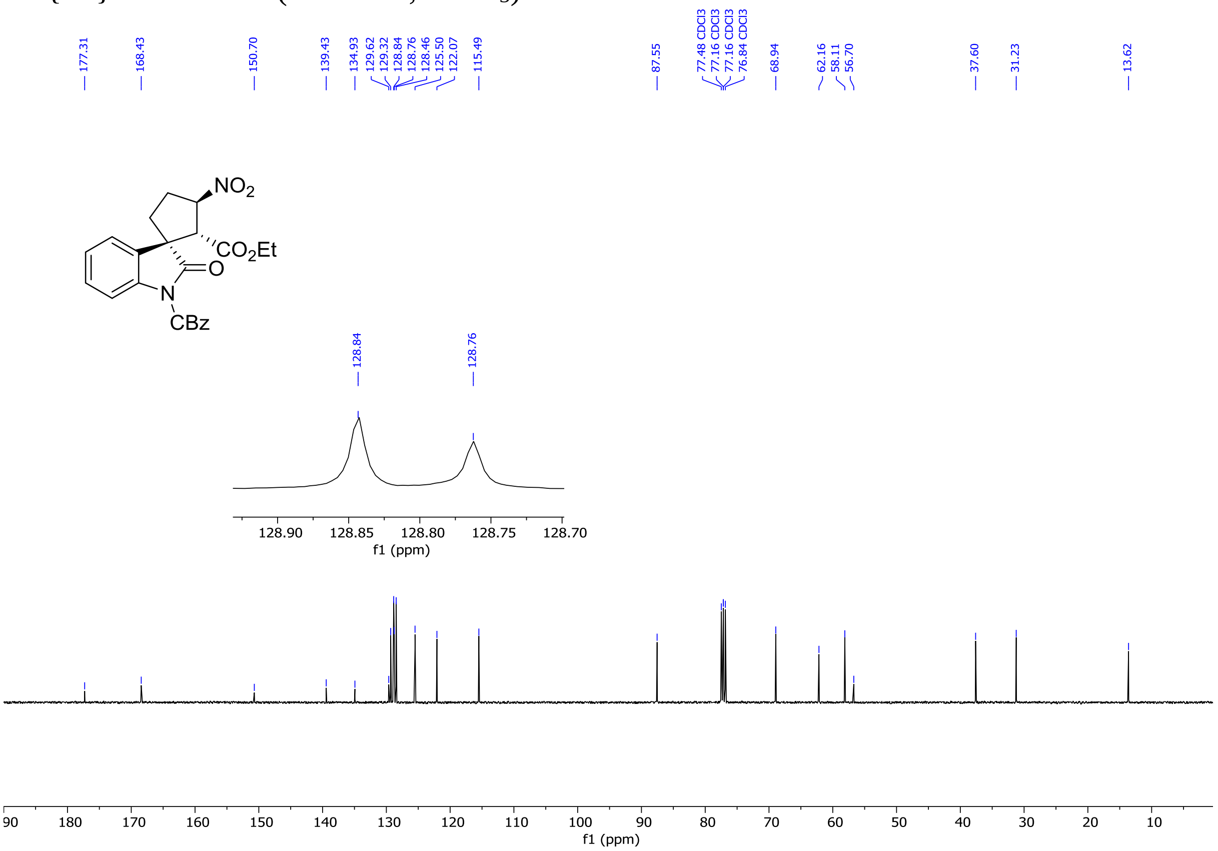

Ethyl (1*R*,2*R*,3*R*)-3-nitro-2'-oxo-1'-tosylspiro[cyclopentane-1,3'-indoline]-2-carboxylate (3c)

^{1}H NMR of **3c** (400 MHz, $CDCl_3$)

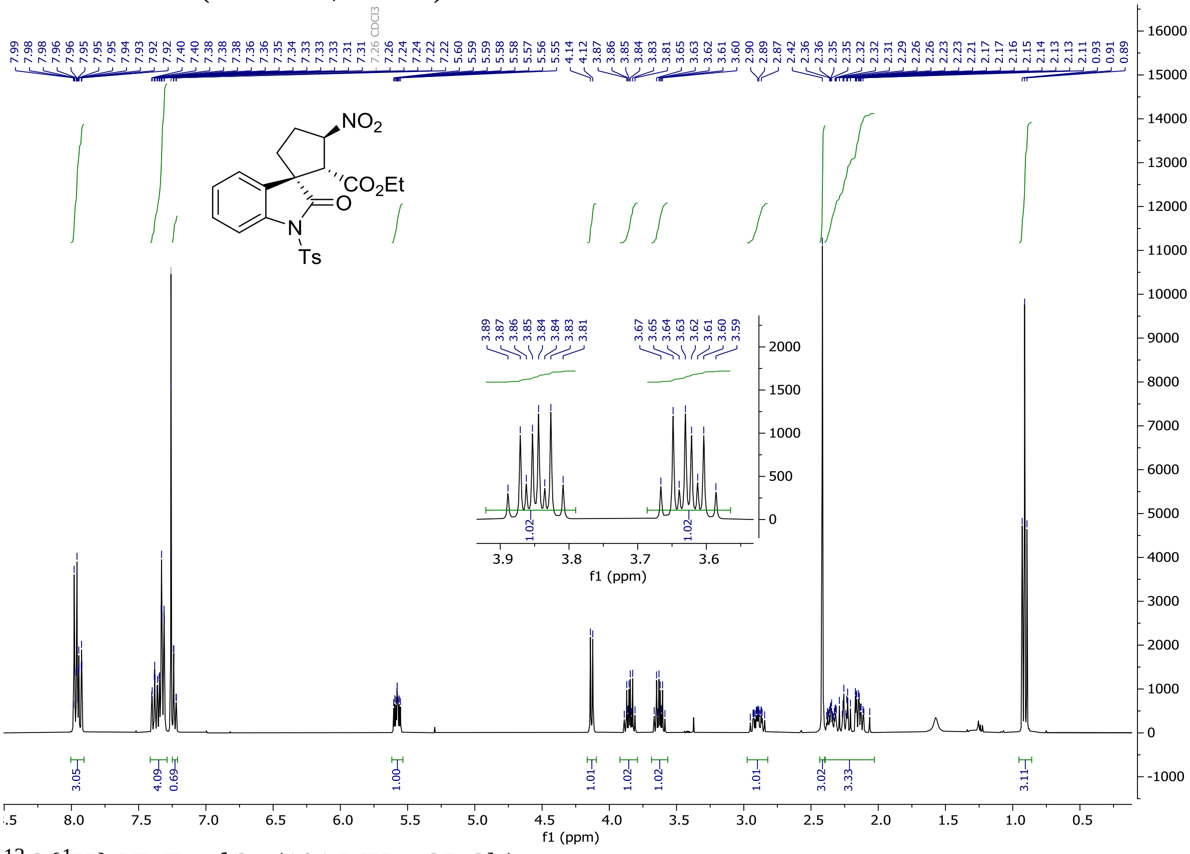

^{13}C{^{1}H} NMR of **3c** (101 MHz, $CDCl_3$)

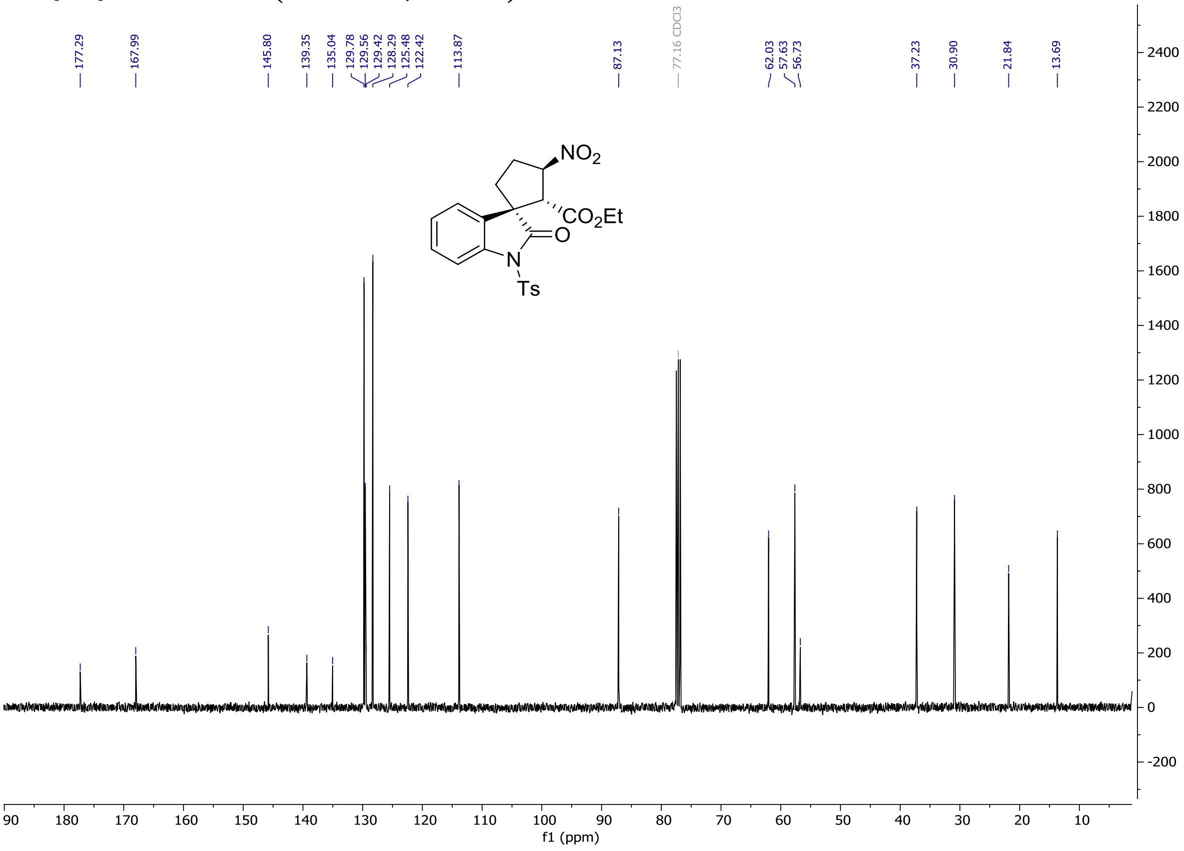

Ethyl 3-nitro-2'-oxo-1'-tosylspiro[cyclopentane-1,3'-indoline]-2-carboxylate (4c/5c)

^{1}H NMR of **4c**/**5c** (400 MHz, $CDCl_3$)

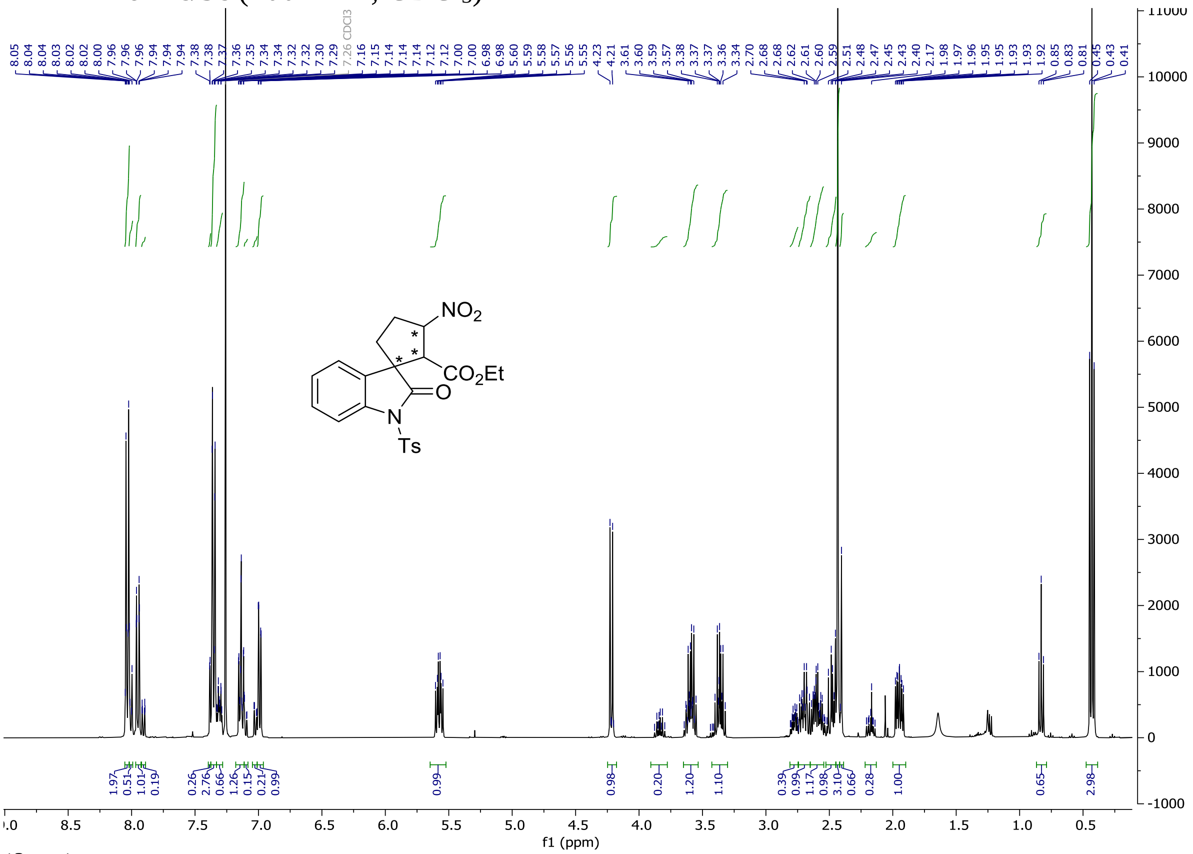

^{13}C{^{1}H} NMR of **4c**/**5c** (101 MHz, $CDCl_3$)

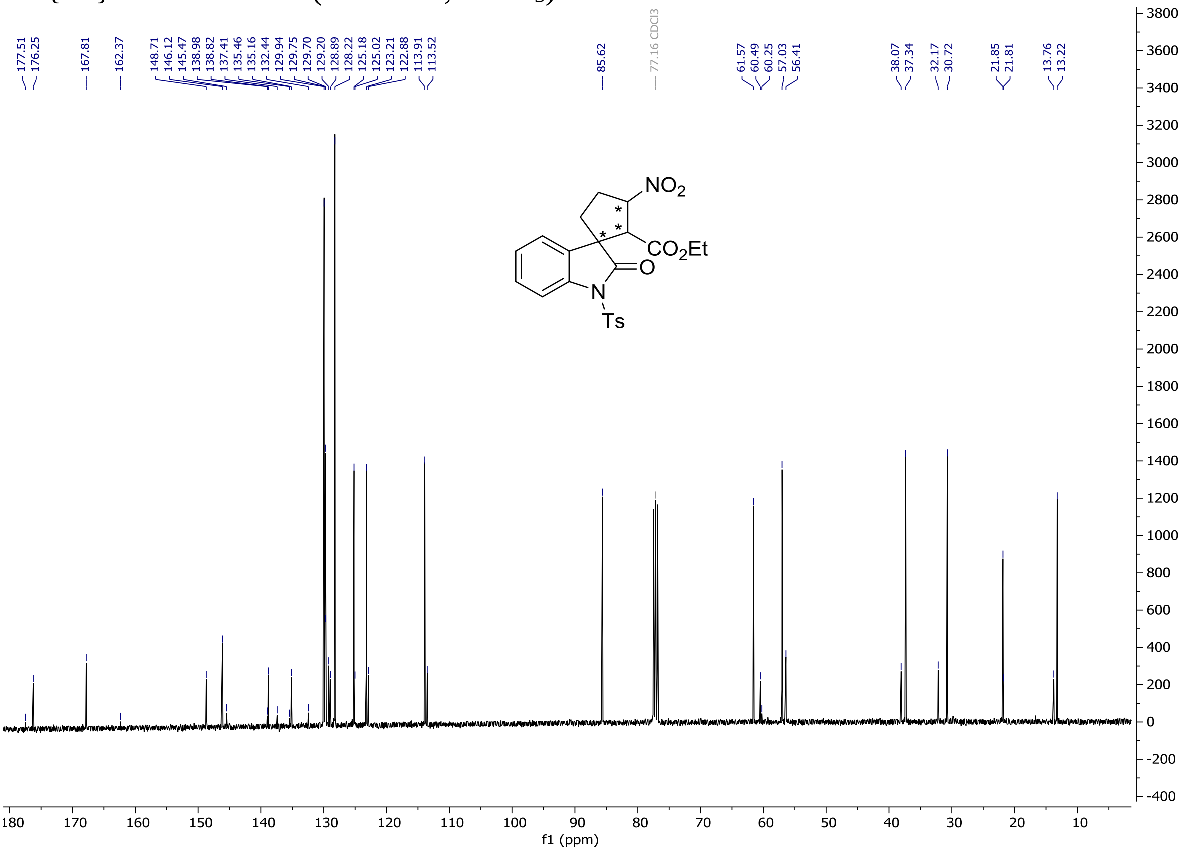

Ethyl (1*R*,2*R*,3*R*)-1'-acetyl-3-nitro-2'-oxospiro[cyclopentane-1,3'-indoline]-2-carboxylate (3d)

^{1}H NMR of **3d** (400 MHz, $CDCl_3$)

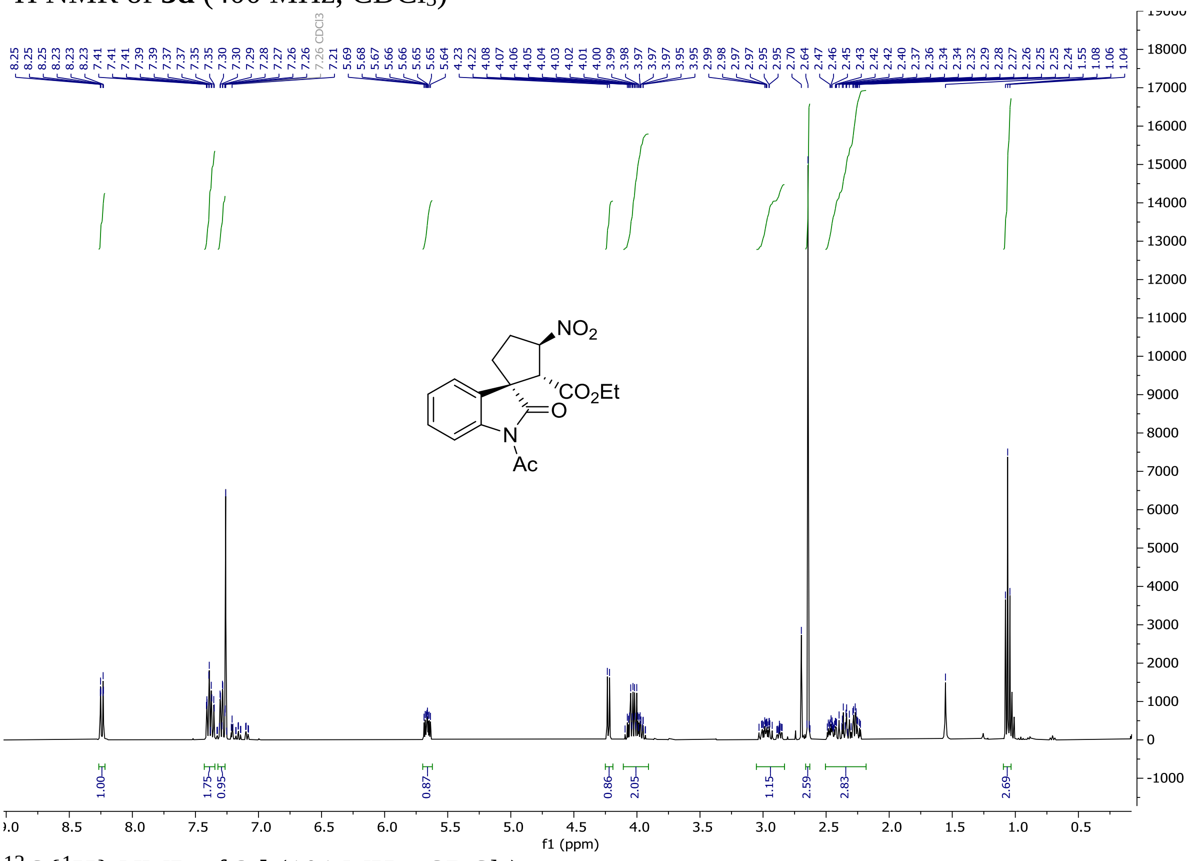

$^{13}C\{^{1}H\}$ NMR of **3d** (101 MHz, $CDCl_3$)

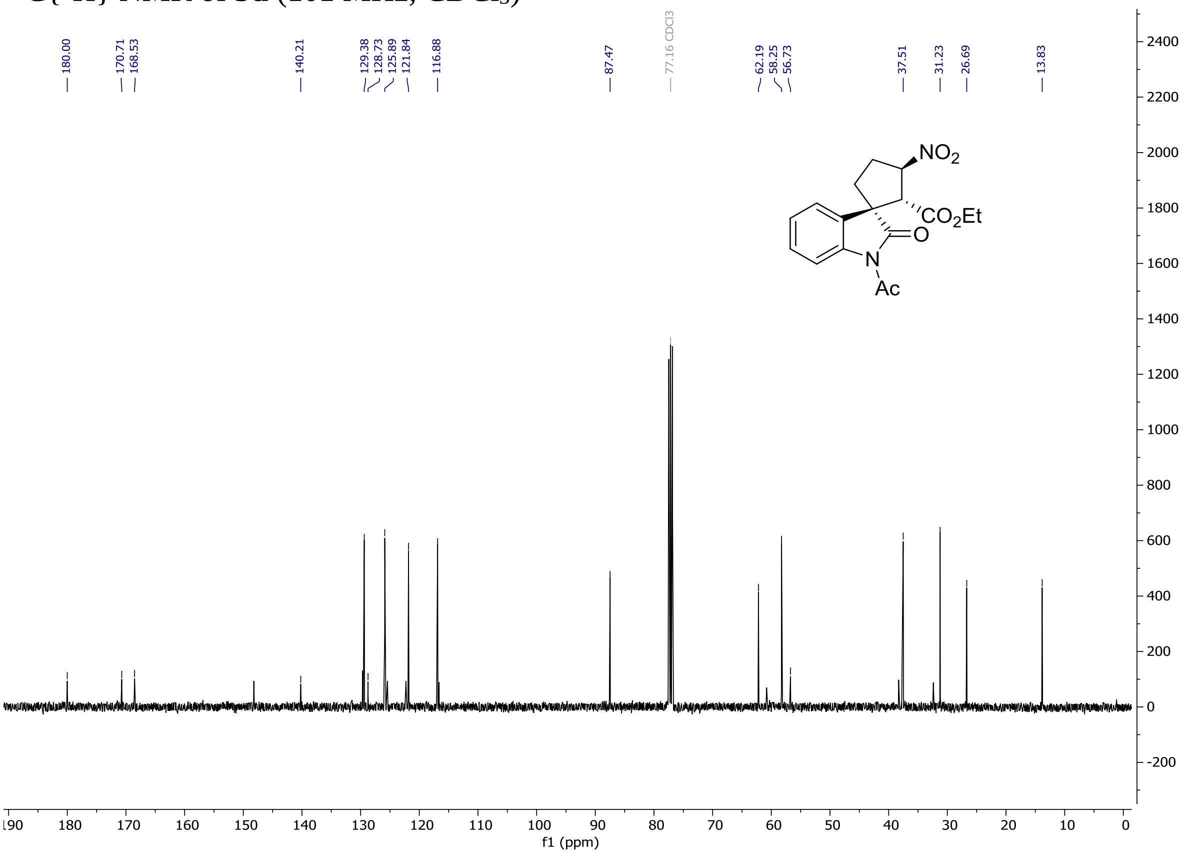

Ethyl -1'-acetyl-3-nitro-2'-oxospiro[cyclopentane-1,3'-indoline]-2-carboxylate (4d)

^{1}H NMR of **4d** (400 MHz, $CDCl_3$)

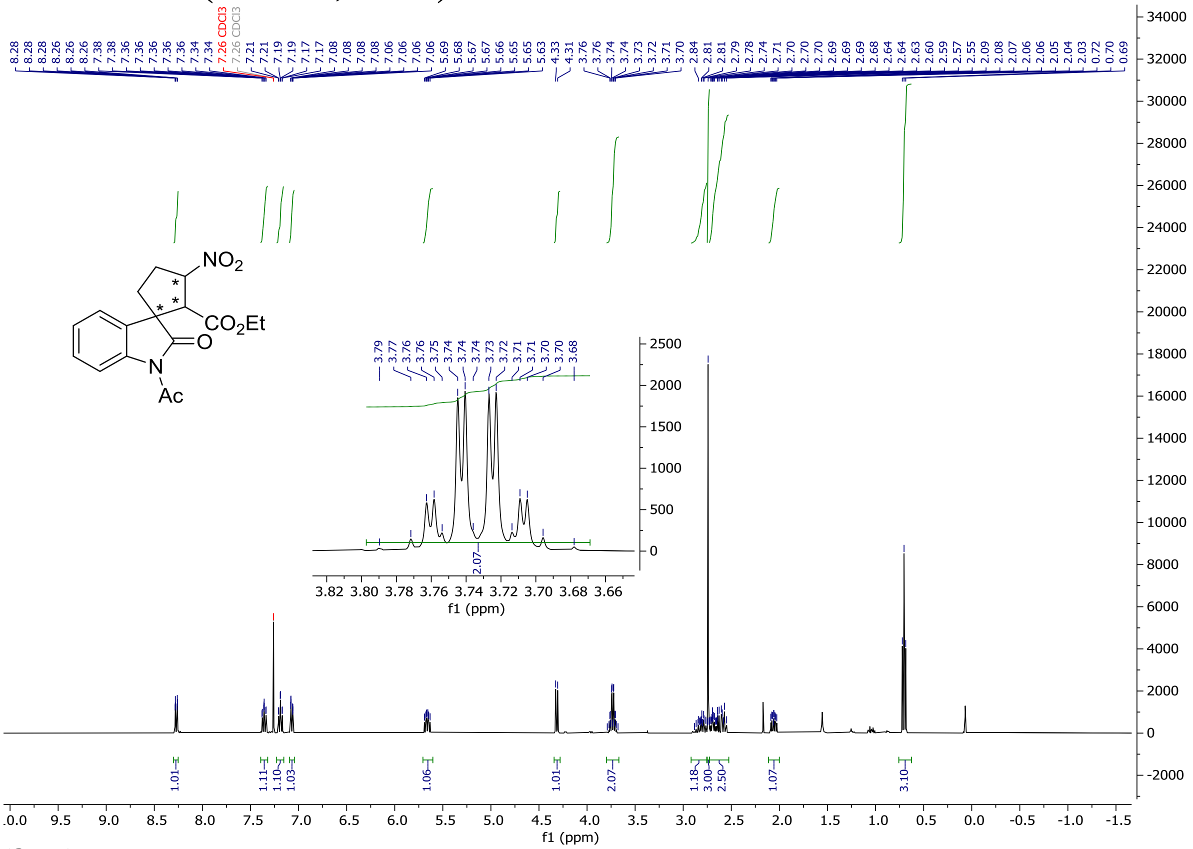

$^{13}C\{^{1}H\}$ NMR of **4d** (101 MHz, $CDCl_3$)

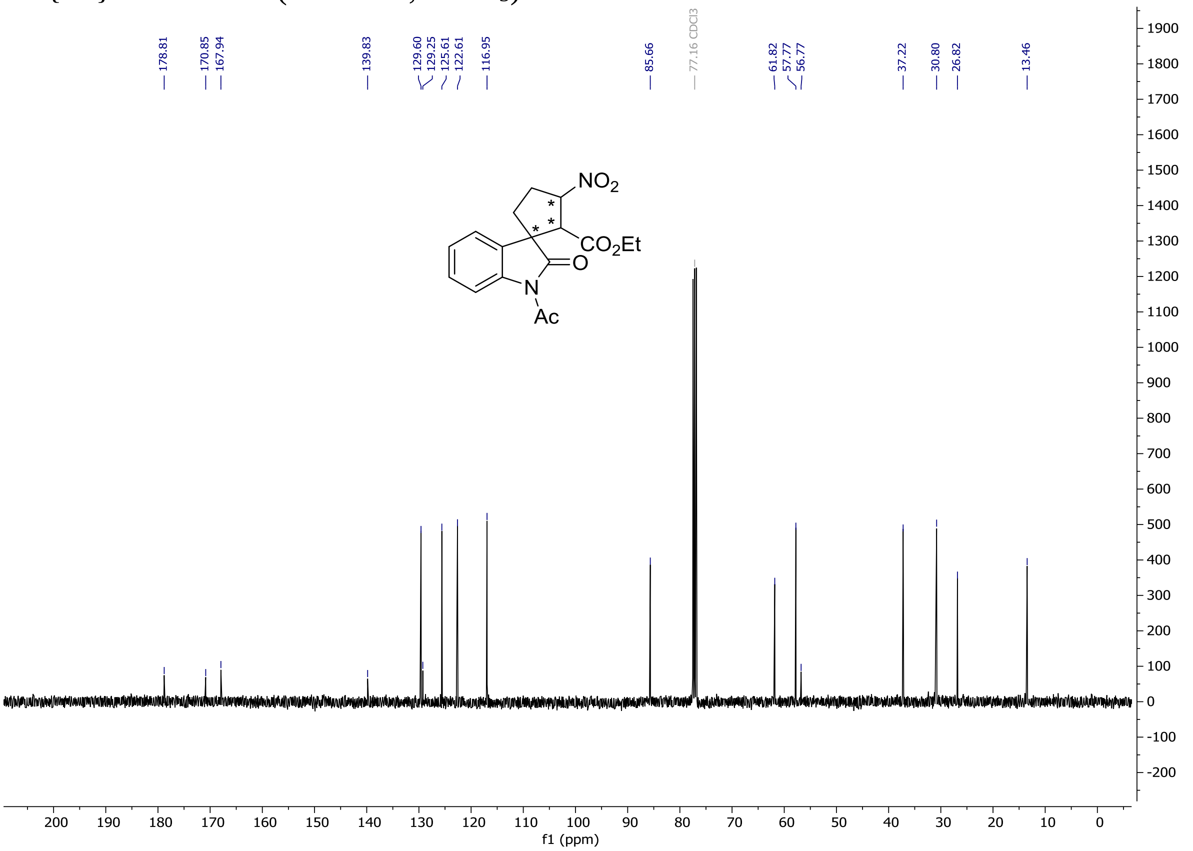

Ethyl 3-nitro-2'-oxospiro[cyclopentane-1,3'-indoline]-2-carboxylate (3f/4f)

^{1}H NMR of **3f/4f** (400 MHz, $CDCl_3$)

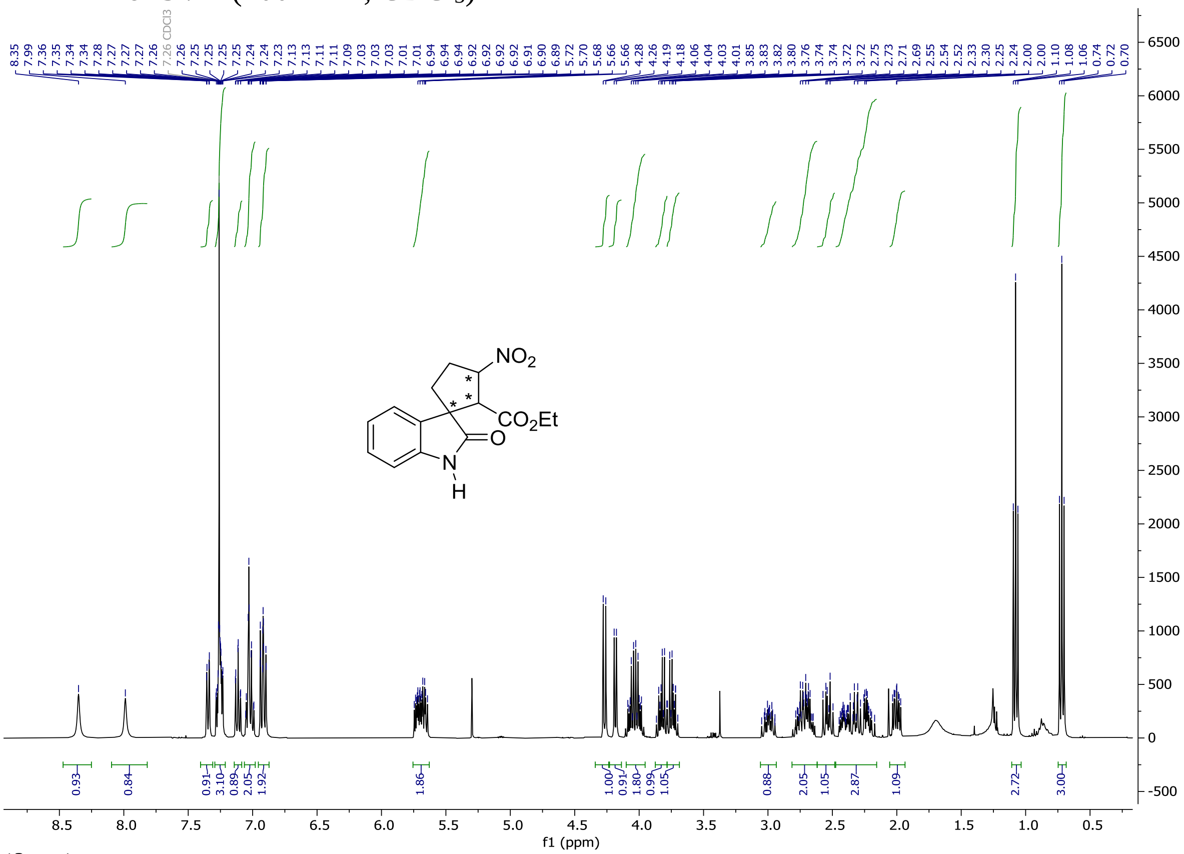

$^{13}C\{^{1}H\}$ NMR of **3f/4f** (101 MHz, $CDCl_3$)

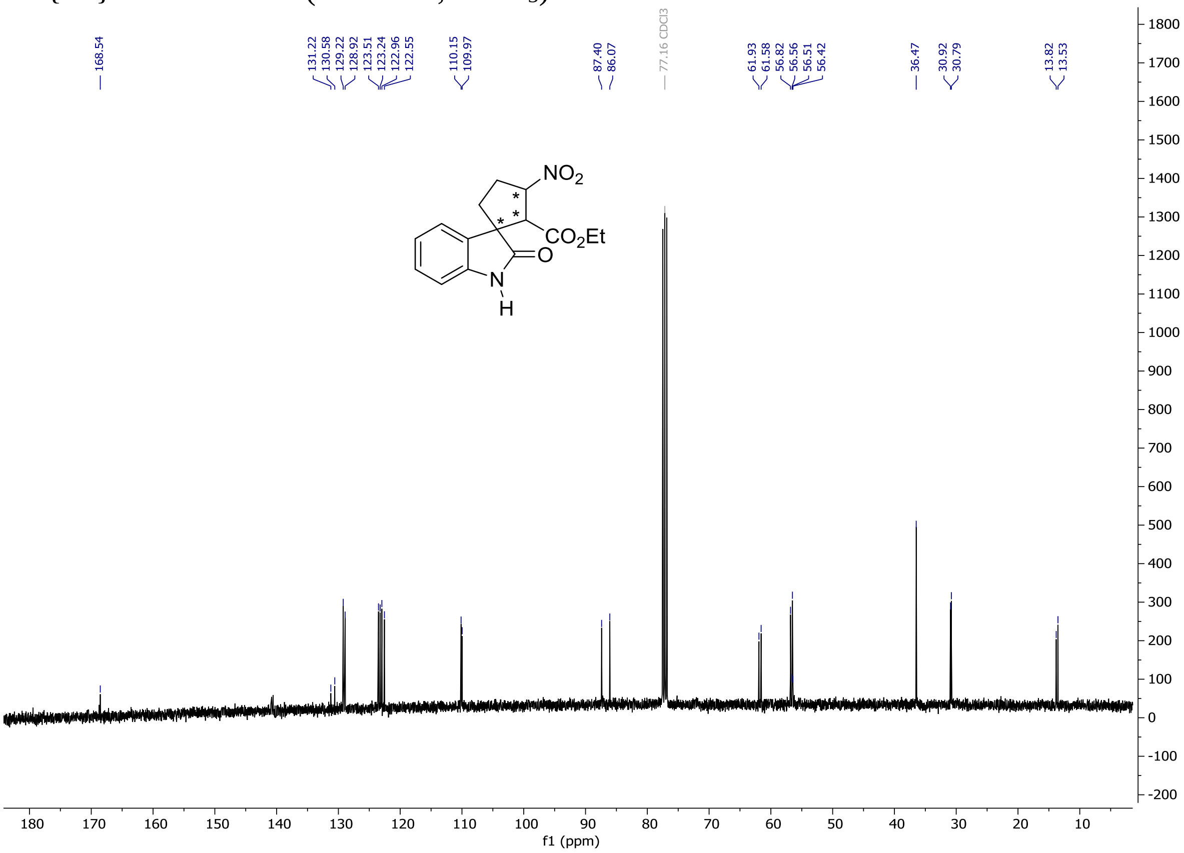

1'-(*tert*-Butyl) 2-ethyl (1*R*,2*R*,3*R*)-5'-methyl-3-nitro-2'-oxospiro[cyclopentane-1,3'-indoline]-1',2-dicarboxylate (3g)

^{1}H NMR of **3g** (400 MHz, $CDCl_3$)

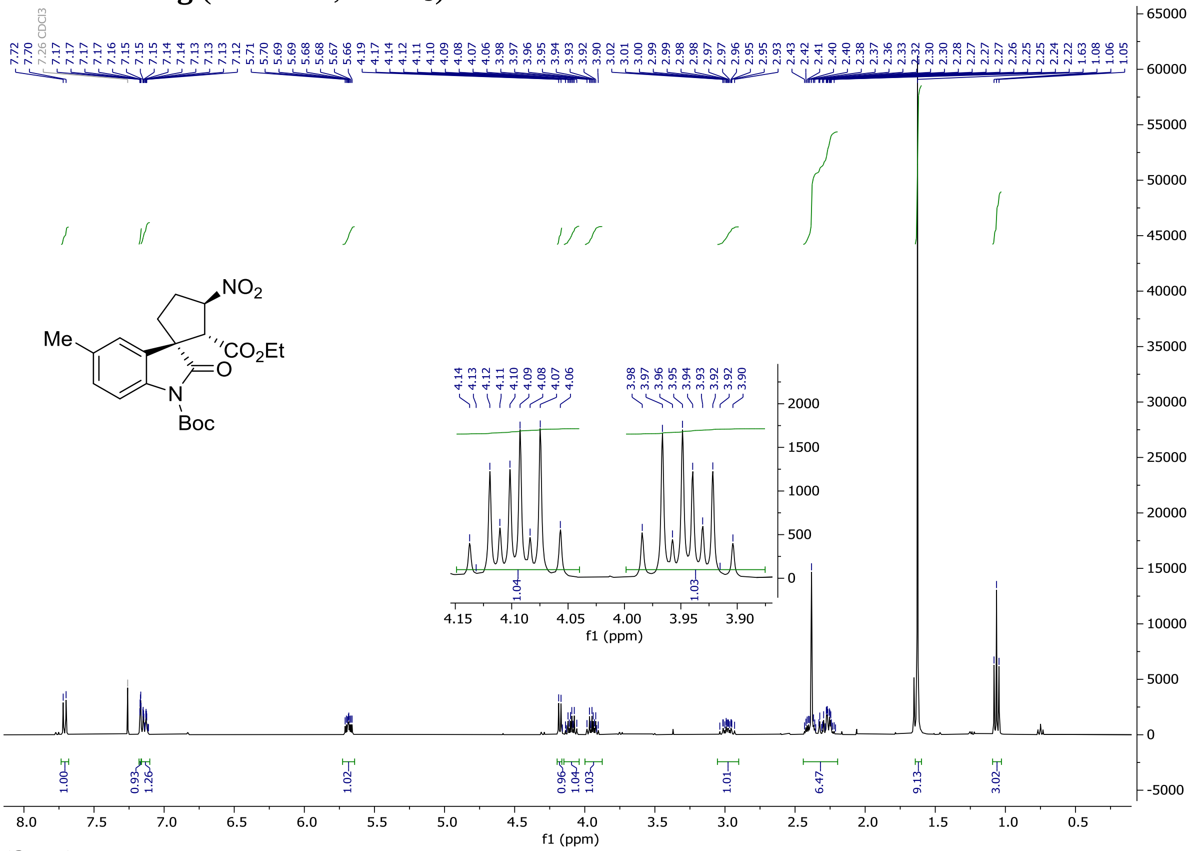

^{13}C{^{1}H} NMR of **3g** (101 MHz, $CDCl_3$)

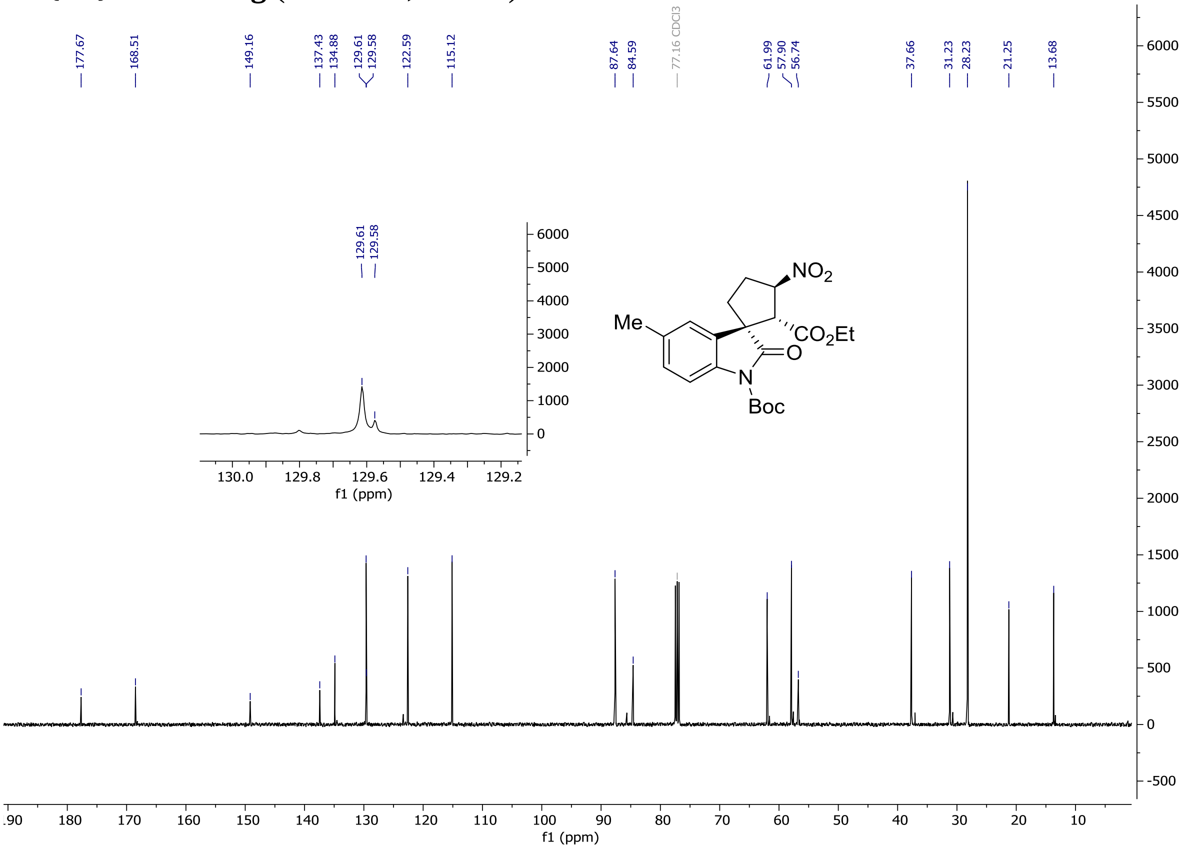

1'-(*tert*-Butyl) 2-ethyl (1*R*,2*R*,3*R*)-5'-methoxy-3-nitro-2'-oxospiro[cyclopentane-1,3'-indoline]-1',2-dicarboxylate (3h)

^{1}H NMR of **3h** (400 MHz, $CDCl_3$)

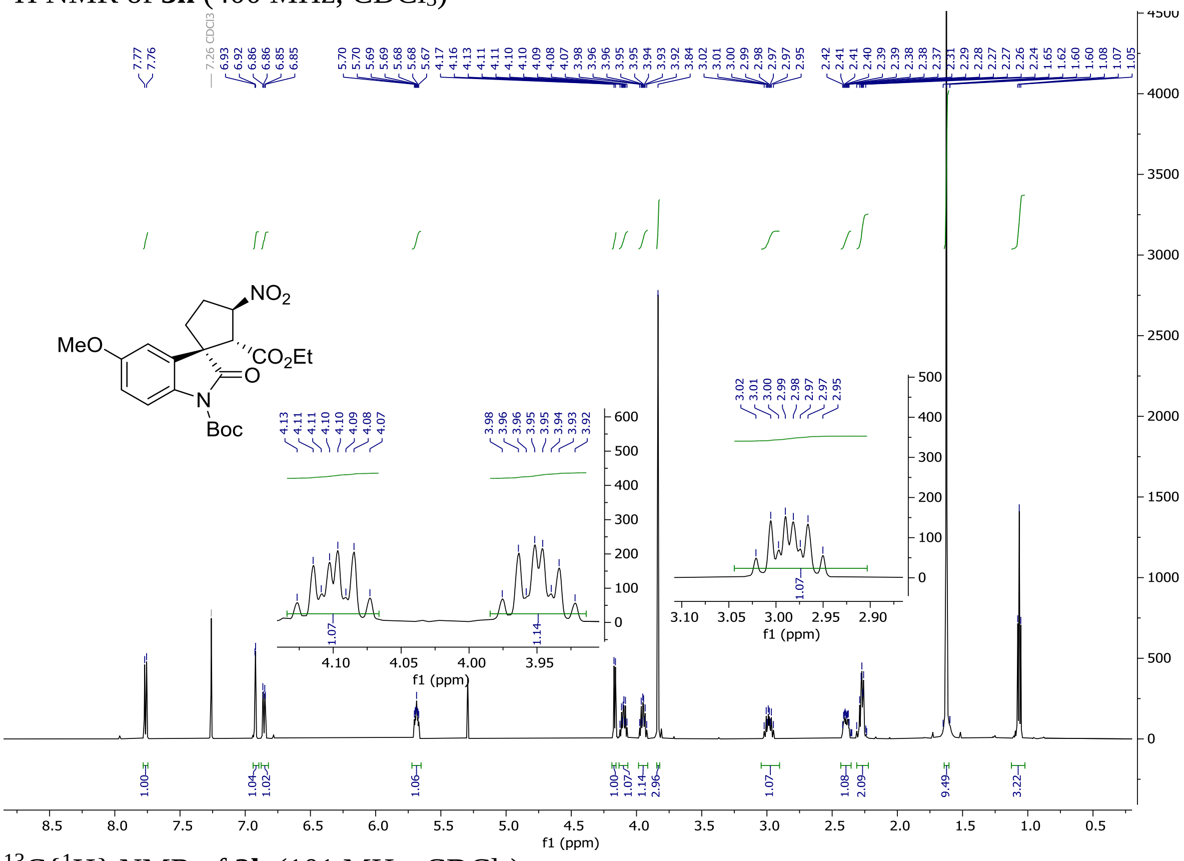

$^{13}C\{^{1}H\}$ NMR of **3h** (101 MHz, $CDCl_3$)

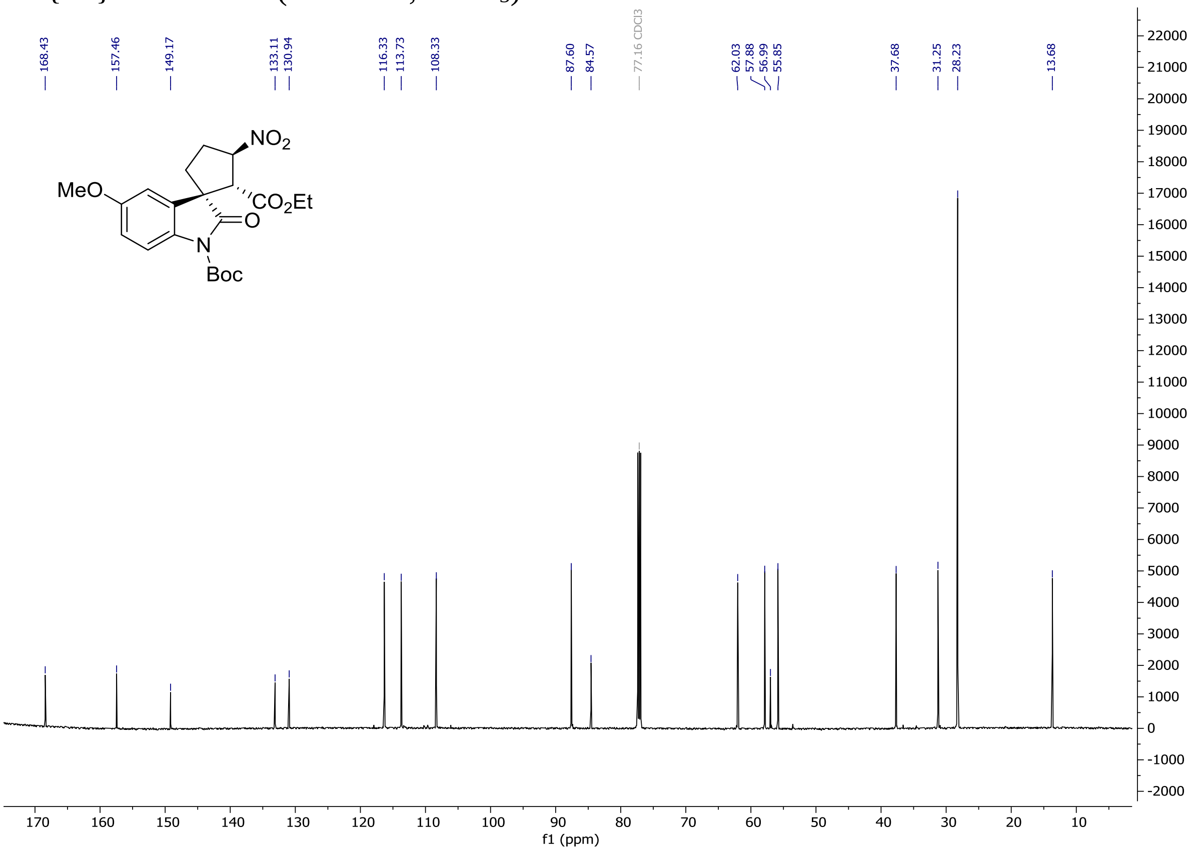

1'-(*tert*-Butyl) 2-ethyl 3-nitro-2'-oxo-5'-(trifluoromethyl)spiro[cyclopentane-1,3'-indoline]-1',2-dicarboxylate (3i/4i/5i)

^{1}H NMR of **3i/4i/5i** (400 MHz, $CDCl_3$)

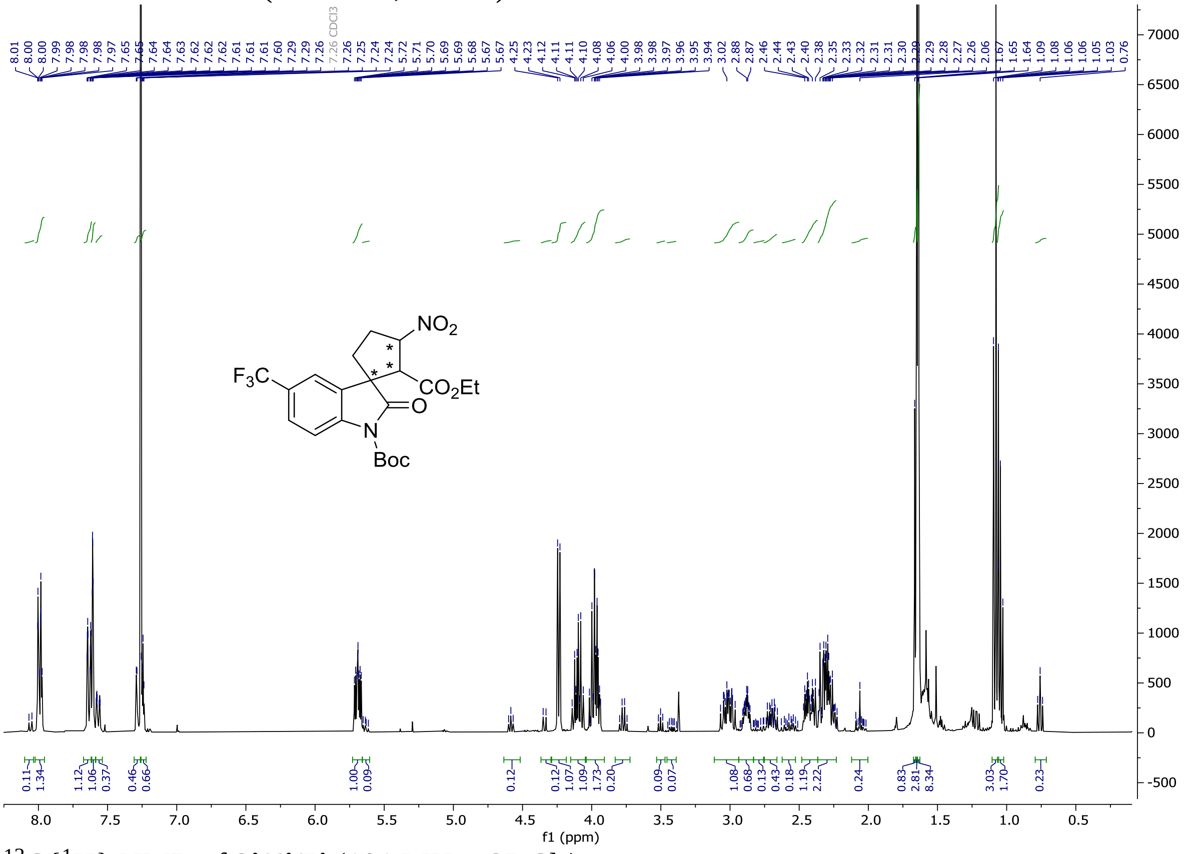

^{13}C{^{1}H} NMR of **3i/4i/5i** (101 MHz, $CDCl_3$)

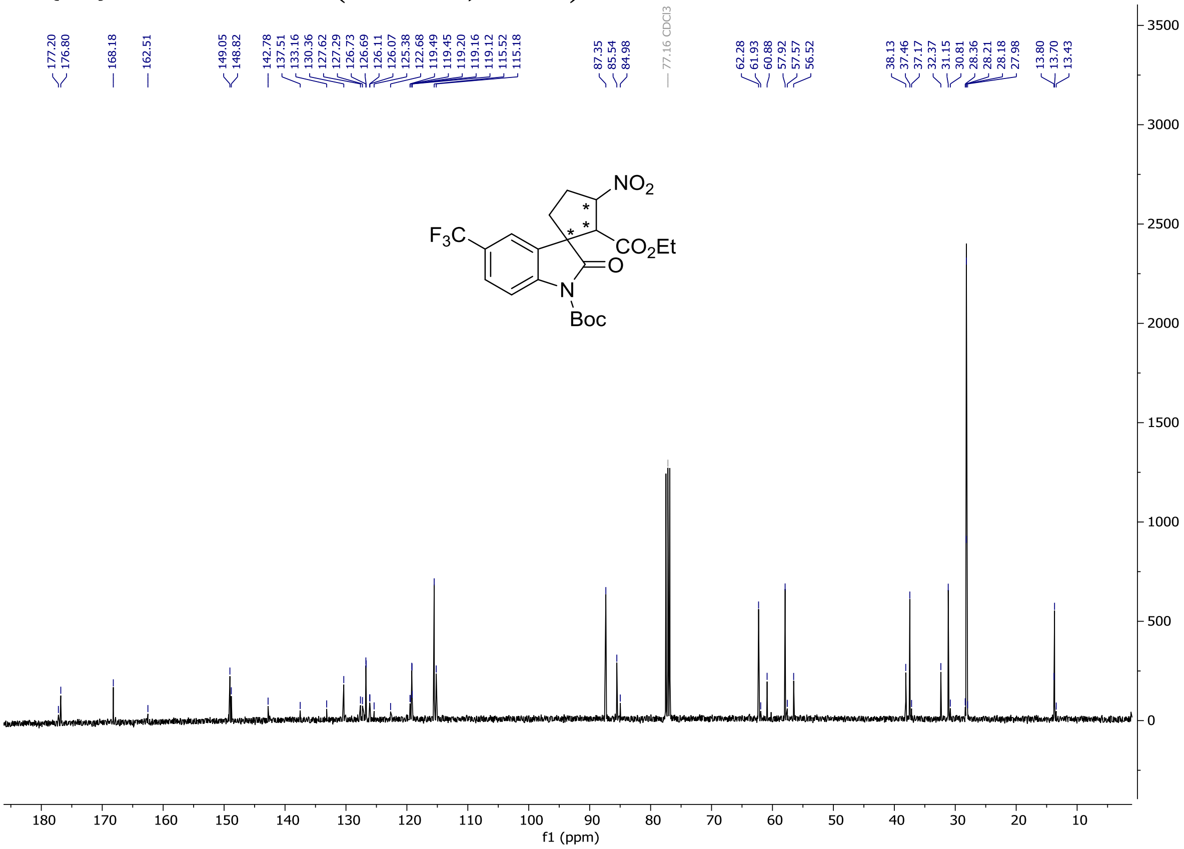

^{19}F NMR of **3i**/**4i**/**5i** (376 MHz MHz, $CDCl_3$)

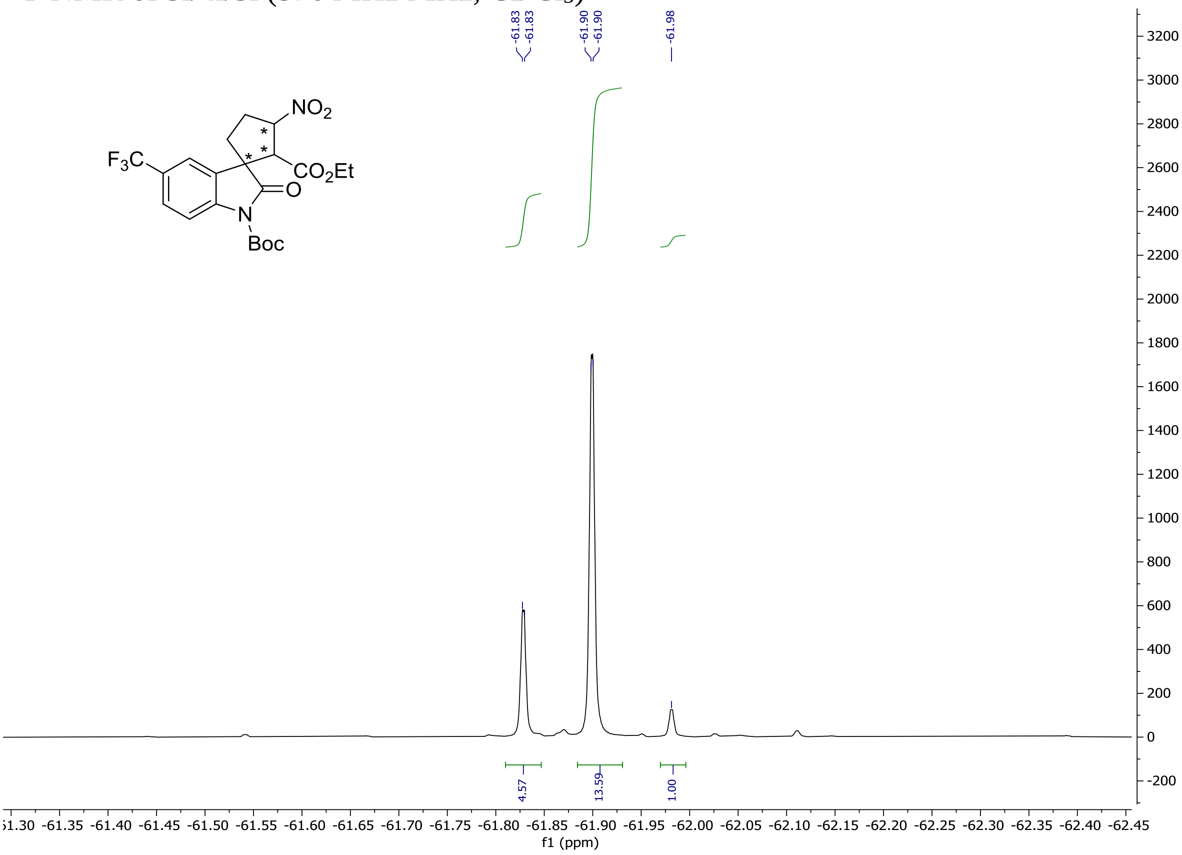

1'-(*tert*-Butyl) 2-ethyl (1*R*,2*R*,3*R*)-5'-fluoro-3-nitro-2'-oxospiro[cyclopentane-1,3'-indoline]-1',2-dicarboxylate (3k)

^{1}H NMR of **3k** (400 MHz, $CDCl_3$)

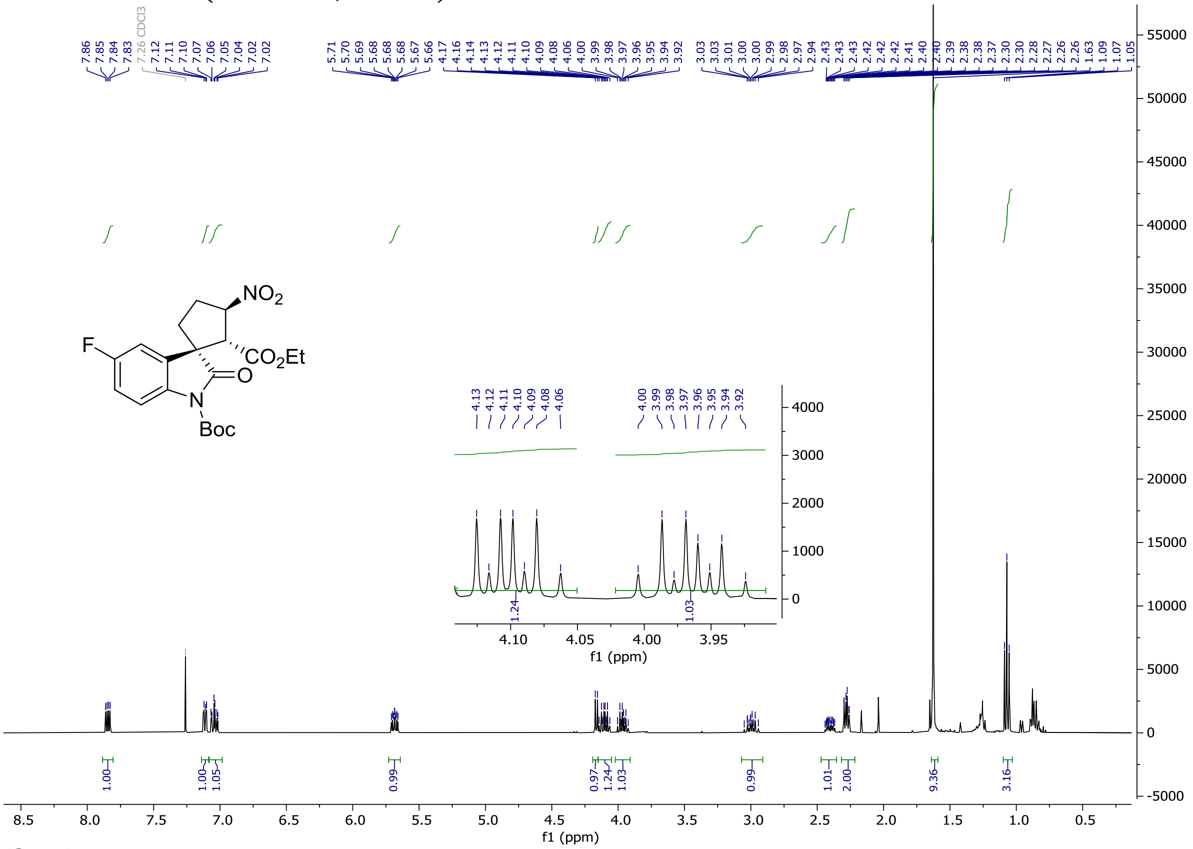

^{13}C{^{1}H} NMR of **3k** (101 MHz, $CDCl_3$)

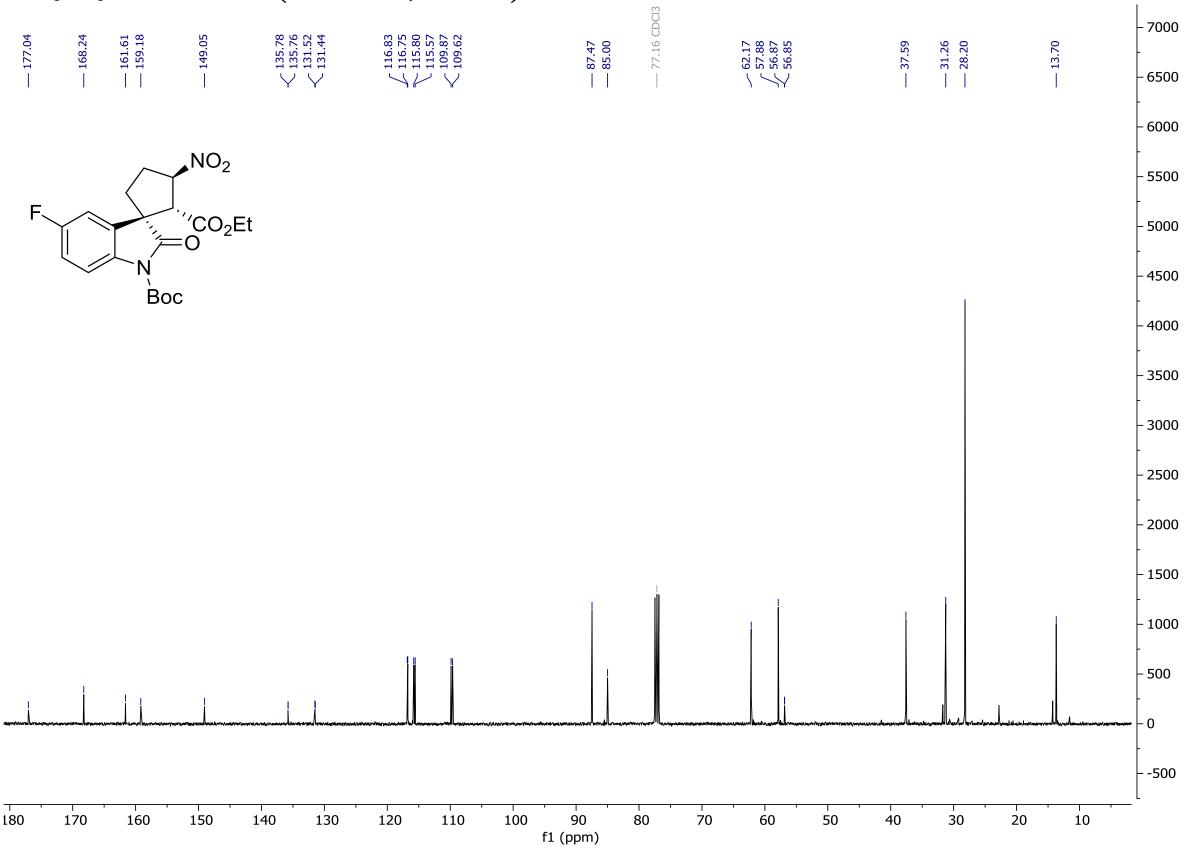

^{19}F NMR of **3k** (376 MHz MHz, $CDCl_3$)

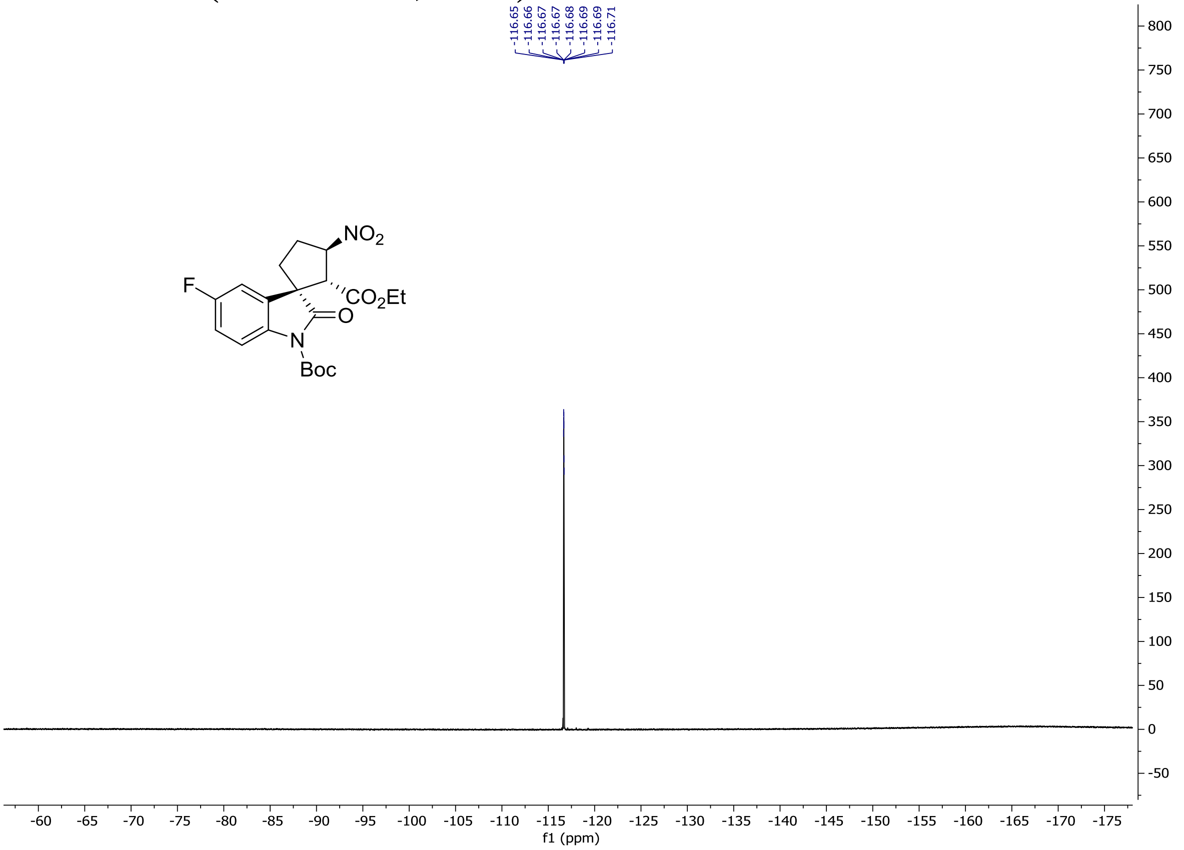

1'-(*tert*-Butyl) 2-ethyl (1*R*,2*R*,3*R*)-5'-chloro-3-nitro-2'-oxospiro[cyclopentane-1,3'-indoline]-1',2-dicarboxylate (3l/5l)

^{1}H NMR of **3l/5l** (400 MHz, $CDCl_3$)

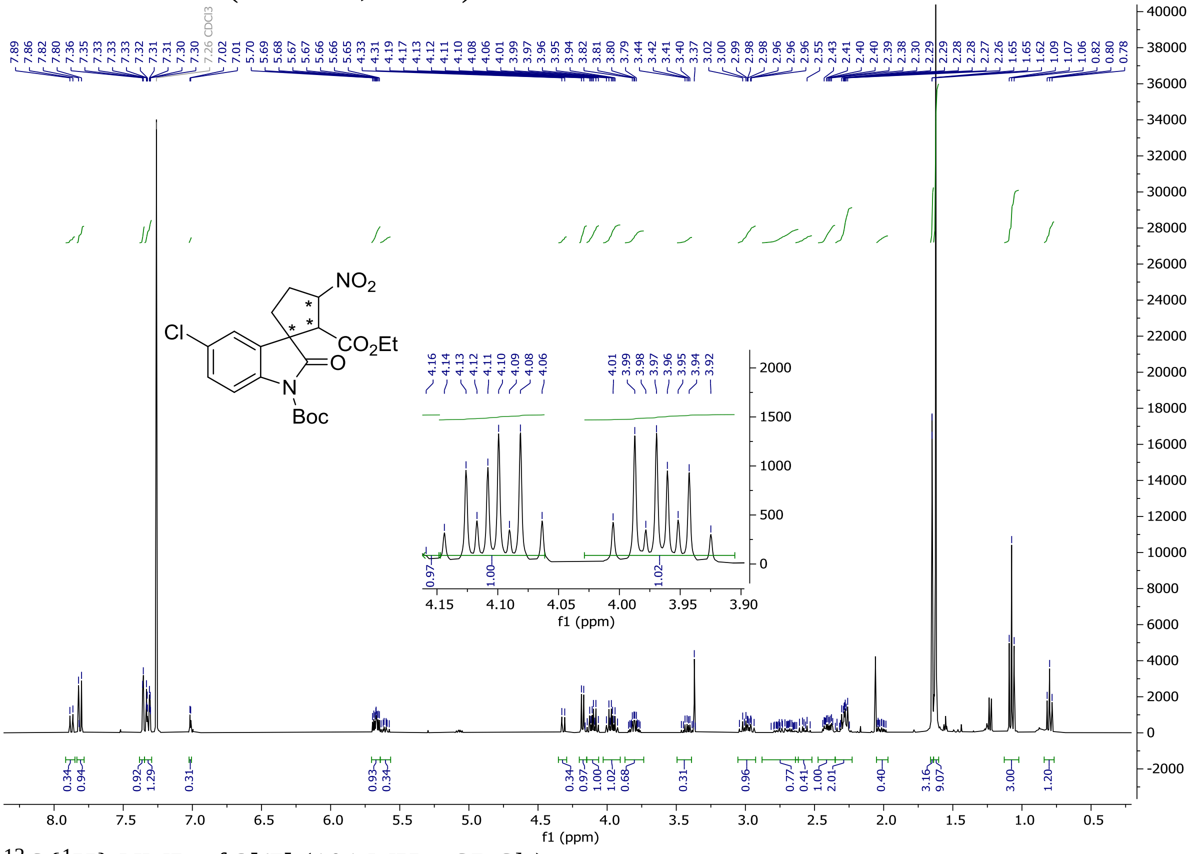

^{13}C{^{1}H} NMR of **3l/5l** (101 MHz, $CDCl_3$)

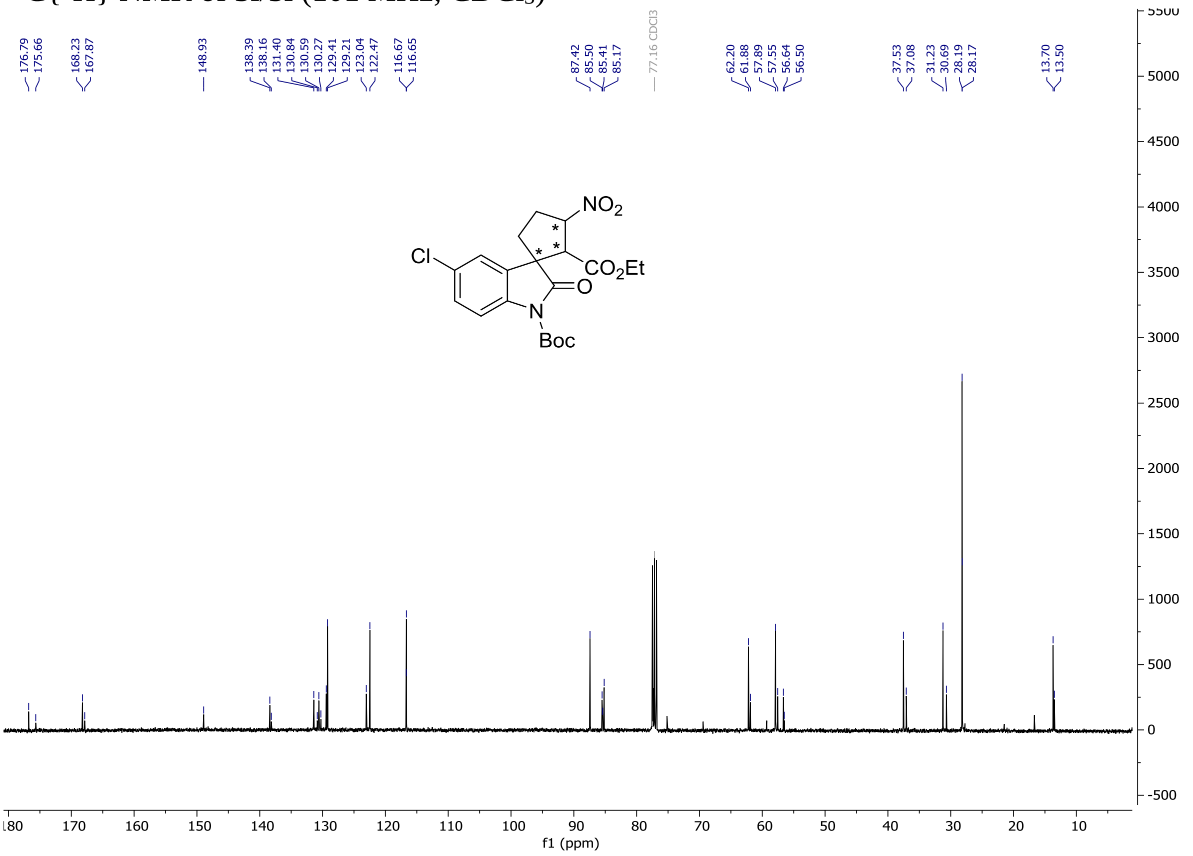

1'-(*tert*-Butyl) 2-ethyl (1*R*,2*R*,3*R*)-5'-bromo-3-nitro-2'-oxospiro[cyclopentane-1,3'-indoline]-1',2-dicarboxylate

^{1}H NMR of **3m** (400 MHz, $CDCl_3$)

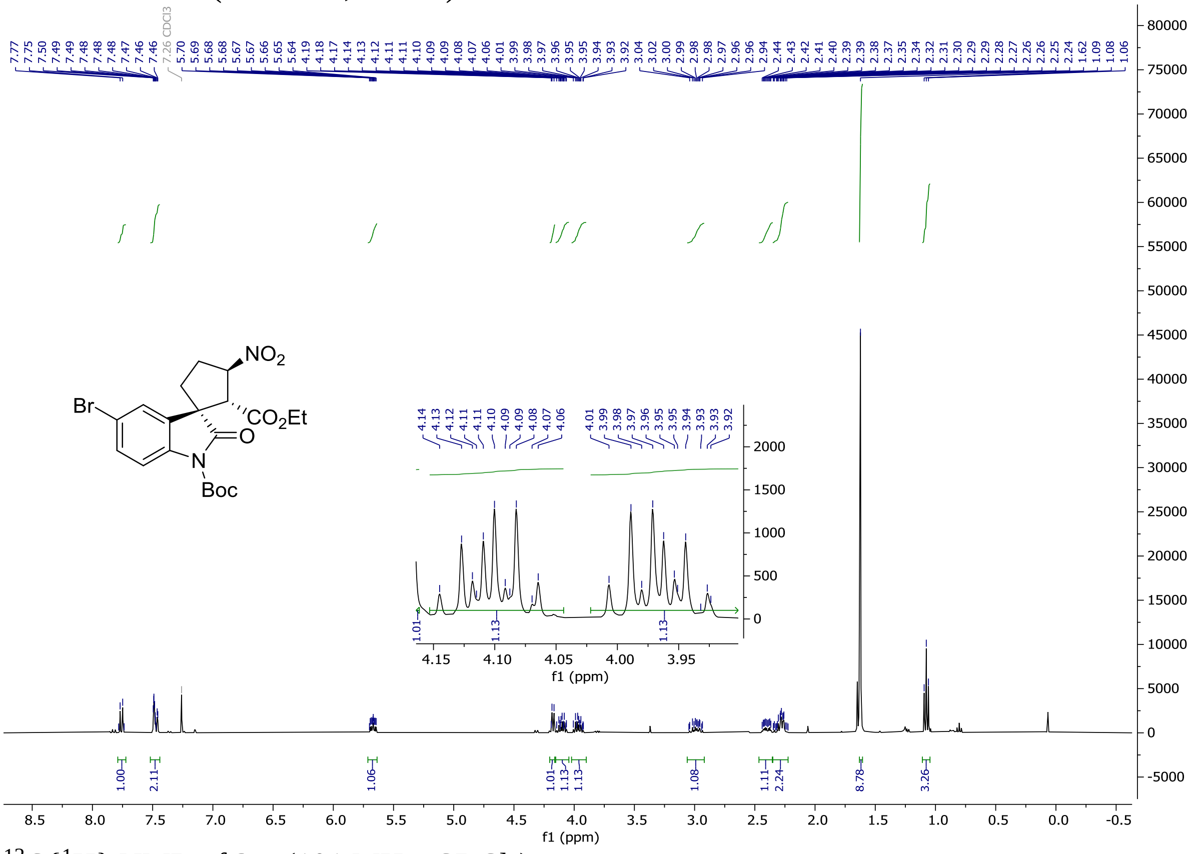

^{13}C{^{1}H} NMR of **3m** (101 MHz, $CDCl_3$)

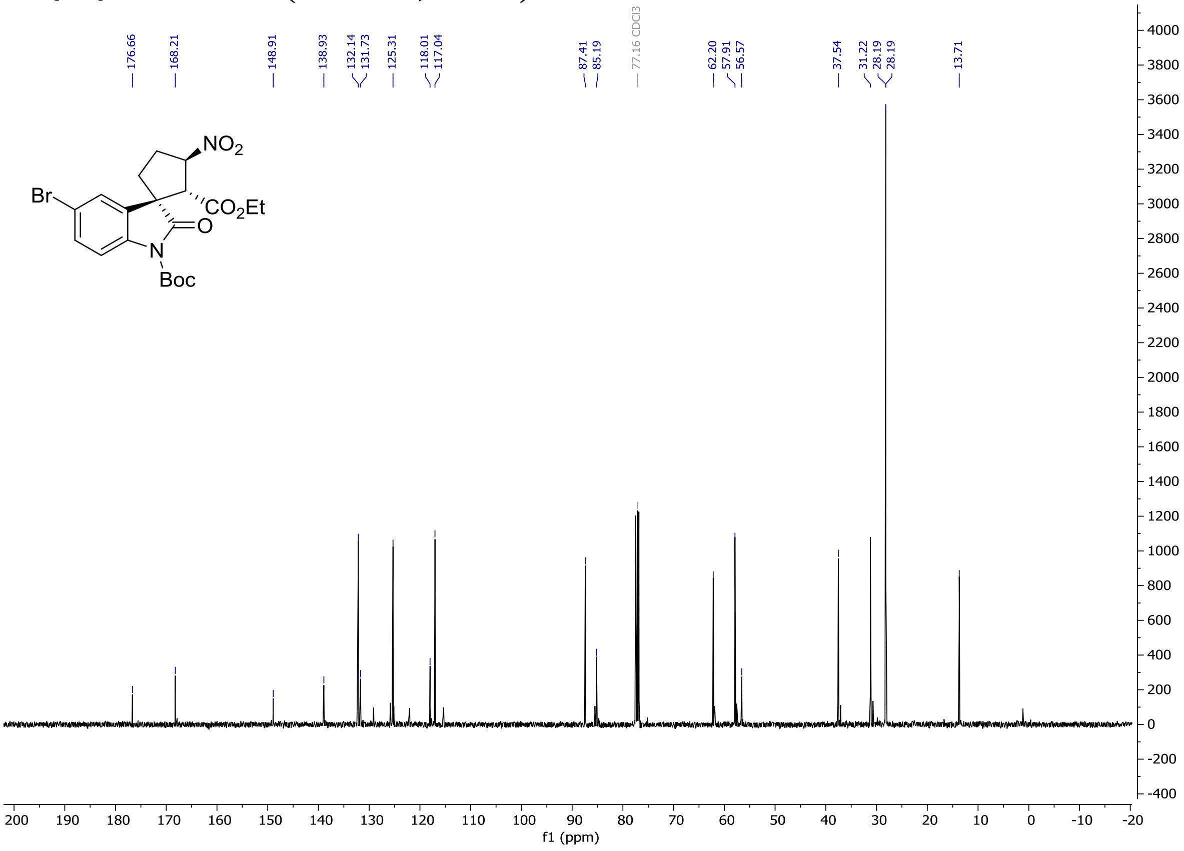

1'-(*tert*-Butyl) 2-ethyl (1*R*,2*R*,3*R*)-6'-bromo-3-nitro-2'-oxospiro[cyclopentane-1,3'-indoline]-1',2-dicarboxylate (3n/4n)

^{1}H NMR of **3n**/**4n** (400 MHz, $CDCl_3$)

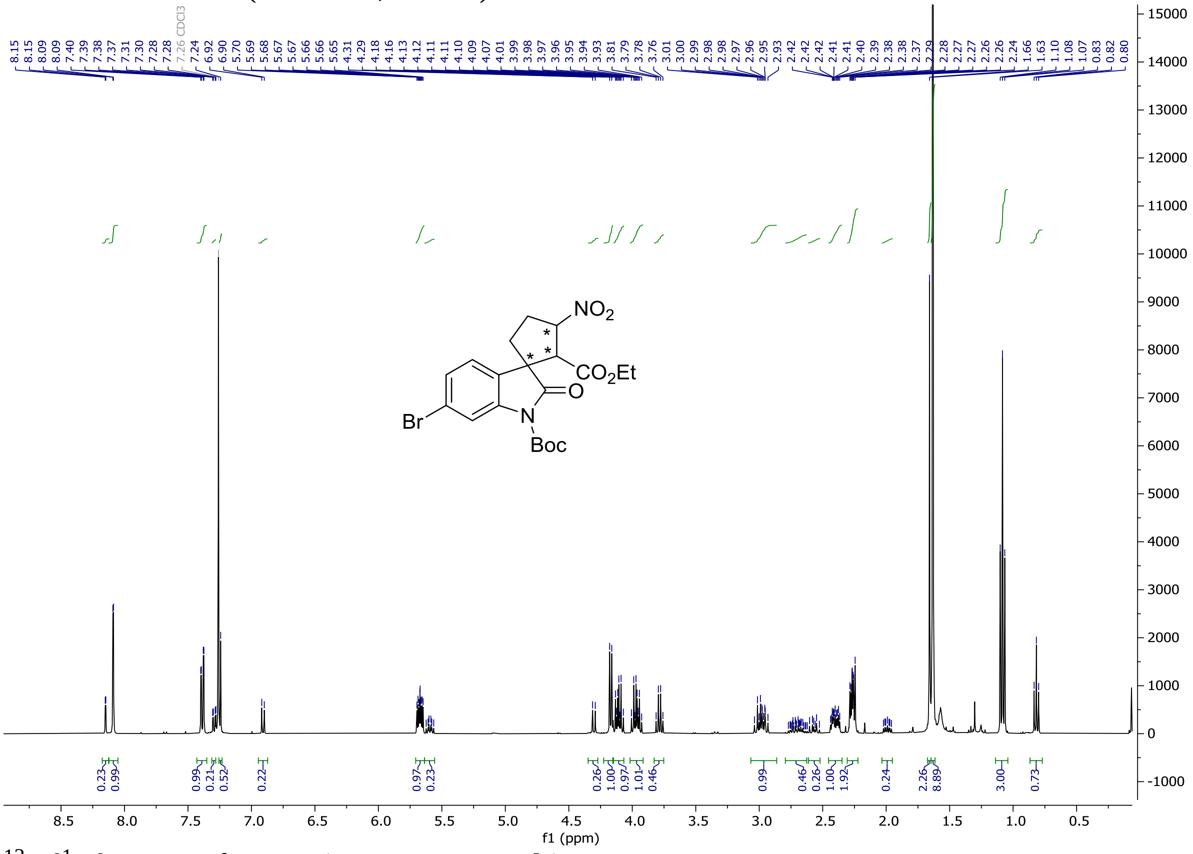

^{13}C{^{1}H} NMR of **3n**/**4n** (101 MHz, $CDCl_3$)

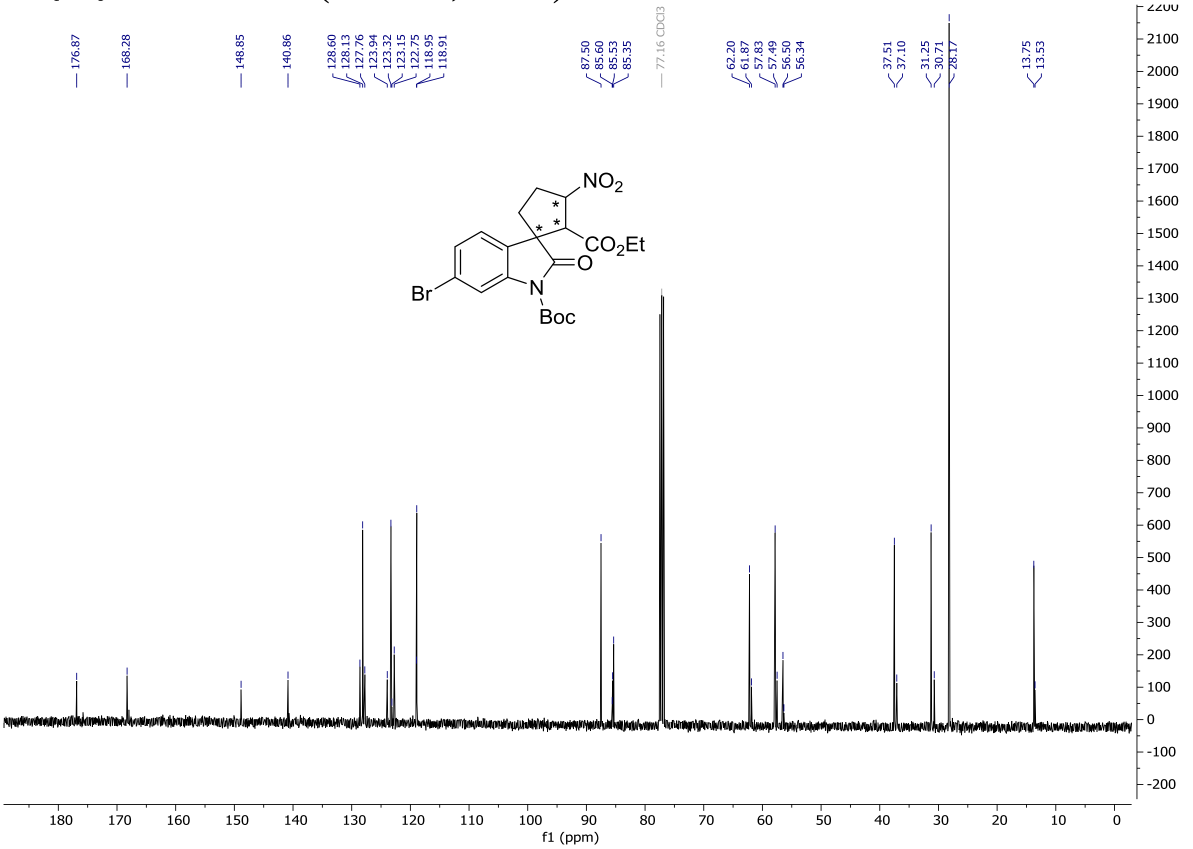

1'-(*tert*-Butyl) 2-methyl (1*R*,2*R*,3*R*)-3-nitro-2'-oxospiro[cyclopentane-1,3'-indoline]-1',2-dicarboxylate (3o)

^{1}H NMR of **3o** (400 MHz, $CDCl_3$)

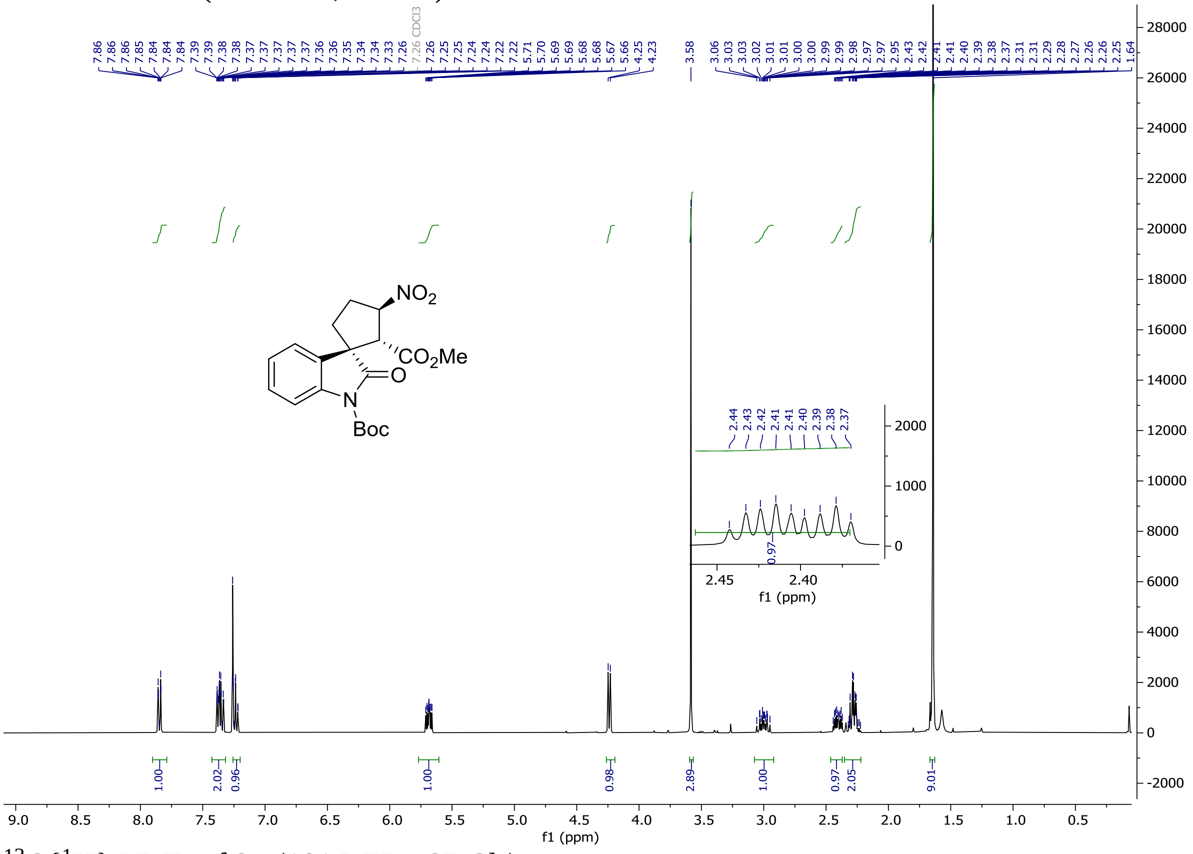

^{13}C{^{1}H} NMR of **3o** (101 MHz, $CDCl_3$)

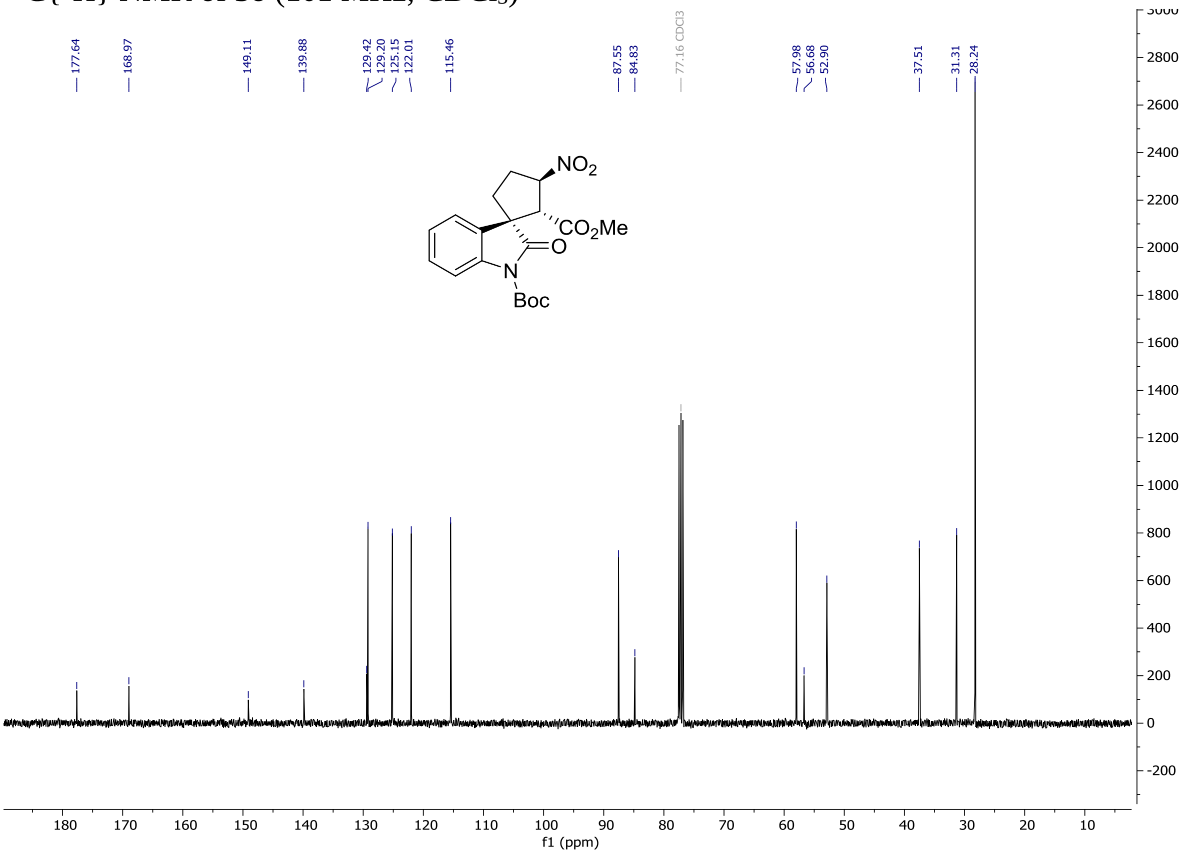

2-Benzyl 1'-(*tert*-butyl) (1*R*,2*R*,3*R*)-3-nitro-2'-oxospiro[cyclopentane-1,3'-indoline]-1',2-dicarboxylate (3p)

^{1}H NMR of **3p** (400 MHz, $CDCl_3$)

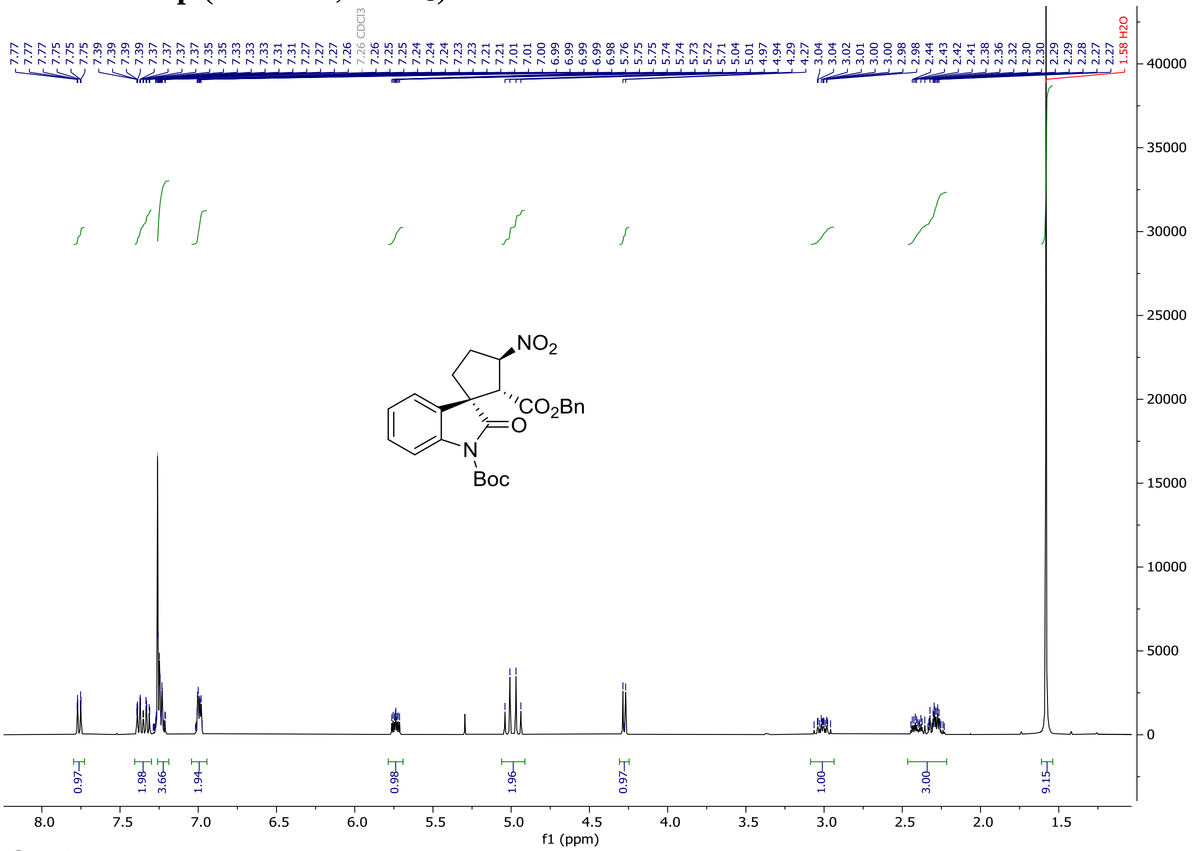

^{13}C{^{1}H} NMR of **3p** (101 MHz, $CDCl_3$)

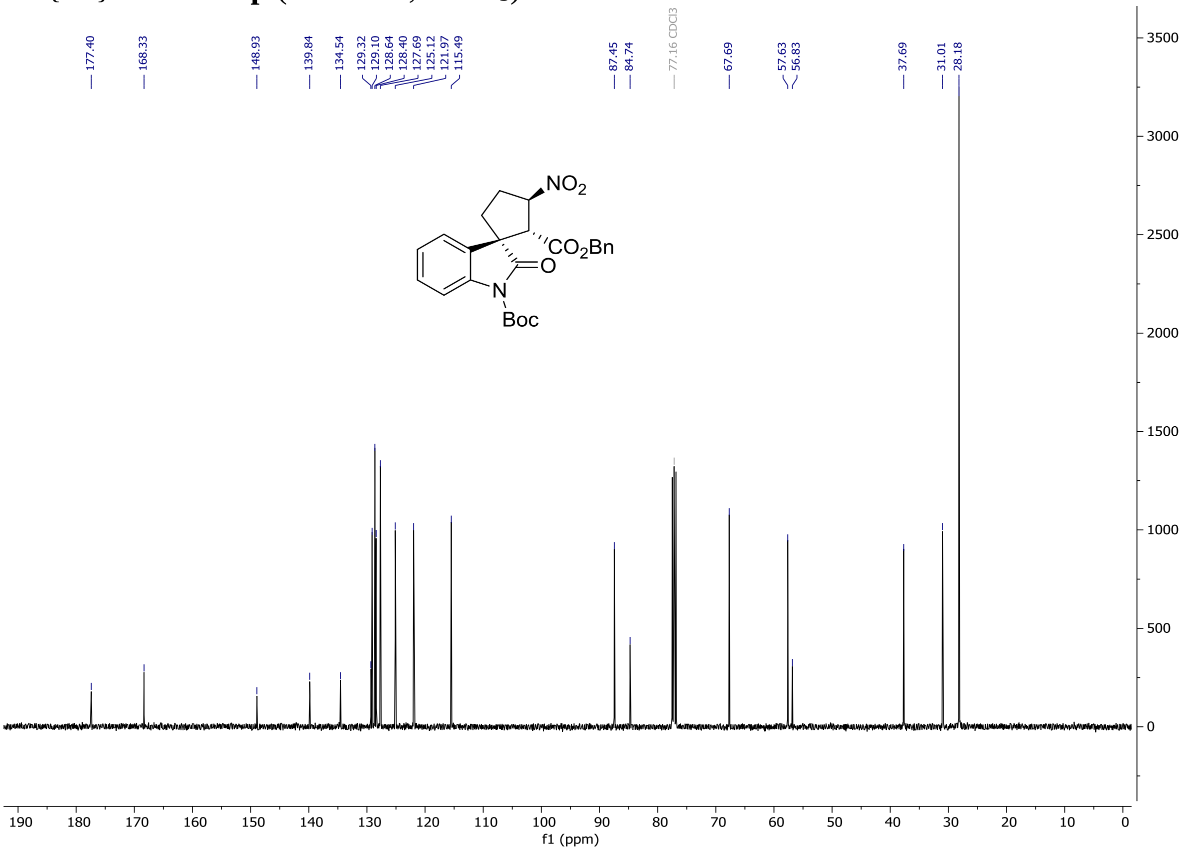

di-*tert*-Butyl (1*R*,2*R*,3*R*)-3-nitro-2'-oxospiro[cyclopentane-1,3'-indoline]-1',2-dicarboxylate (3q)

^{1}H NMR of **3q** (400 MHz, $CDCl_3$)

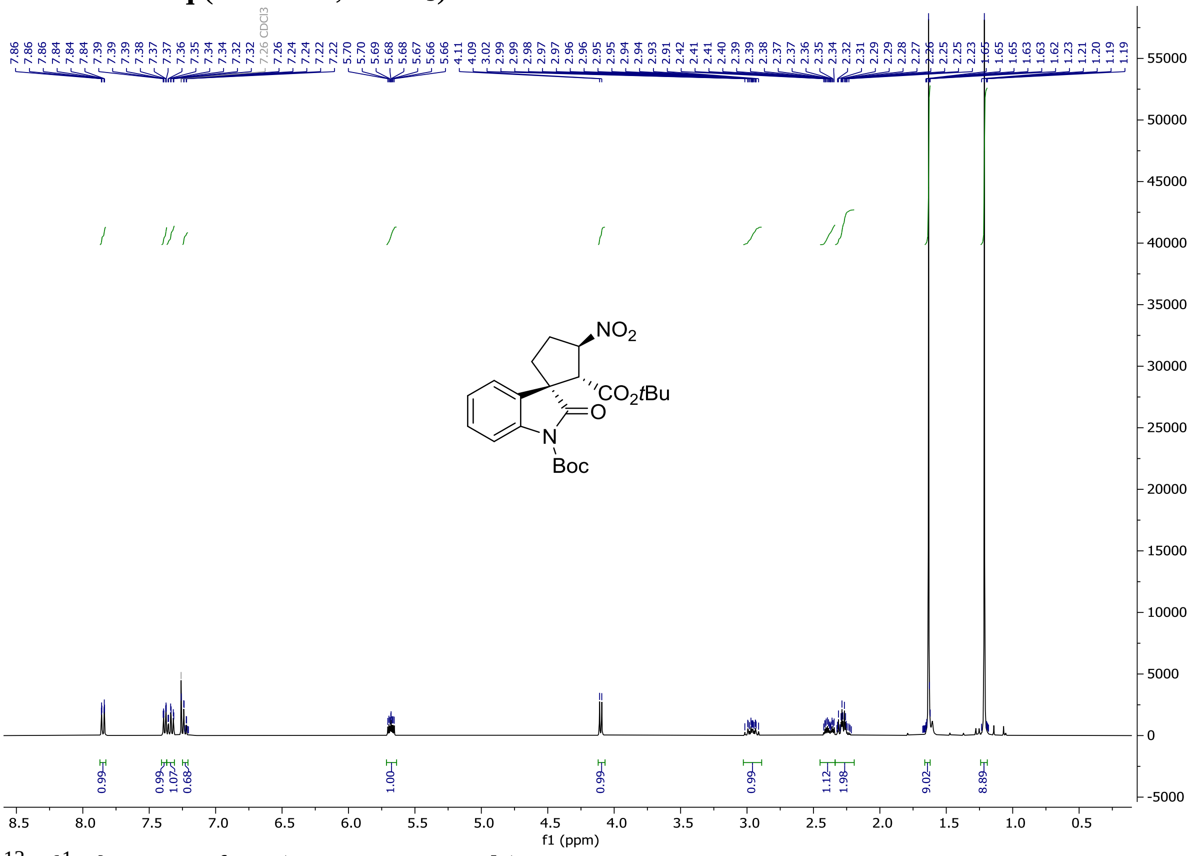

$^{13}C\{^1H\}$ NMR of **3q** (101 MHz, $CDCl_3$)

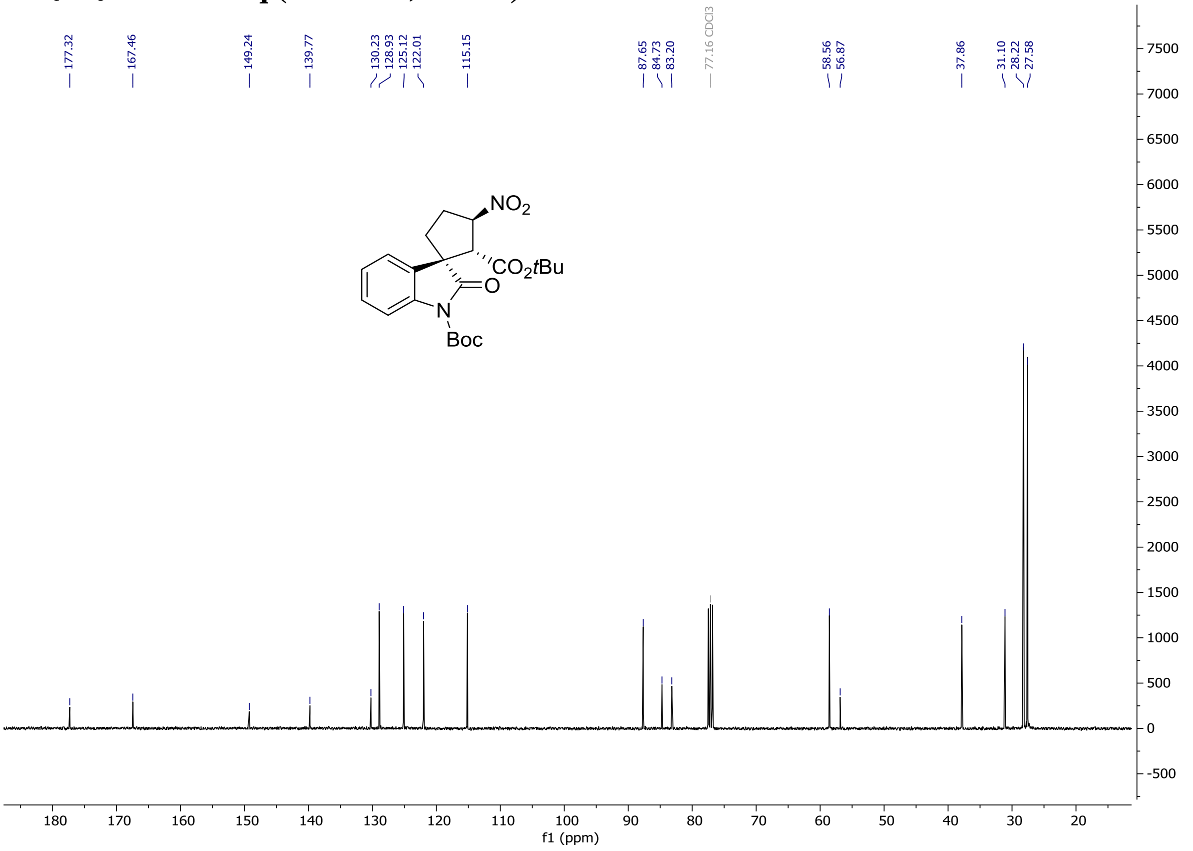

1'-(*tert*-Butyl) 2,2-diethyl 3-nitro-2'-oxospiro[cyclopentane-1,3'-indoline]-1',2,2-tricarboxylate (3r)

^{1}H NMR of **3r** (400 MHz, $CDCl_3$)

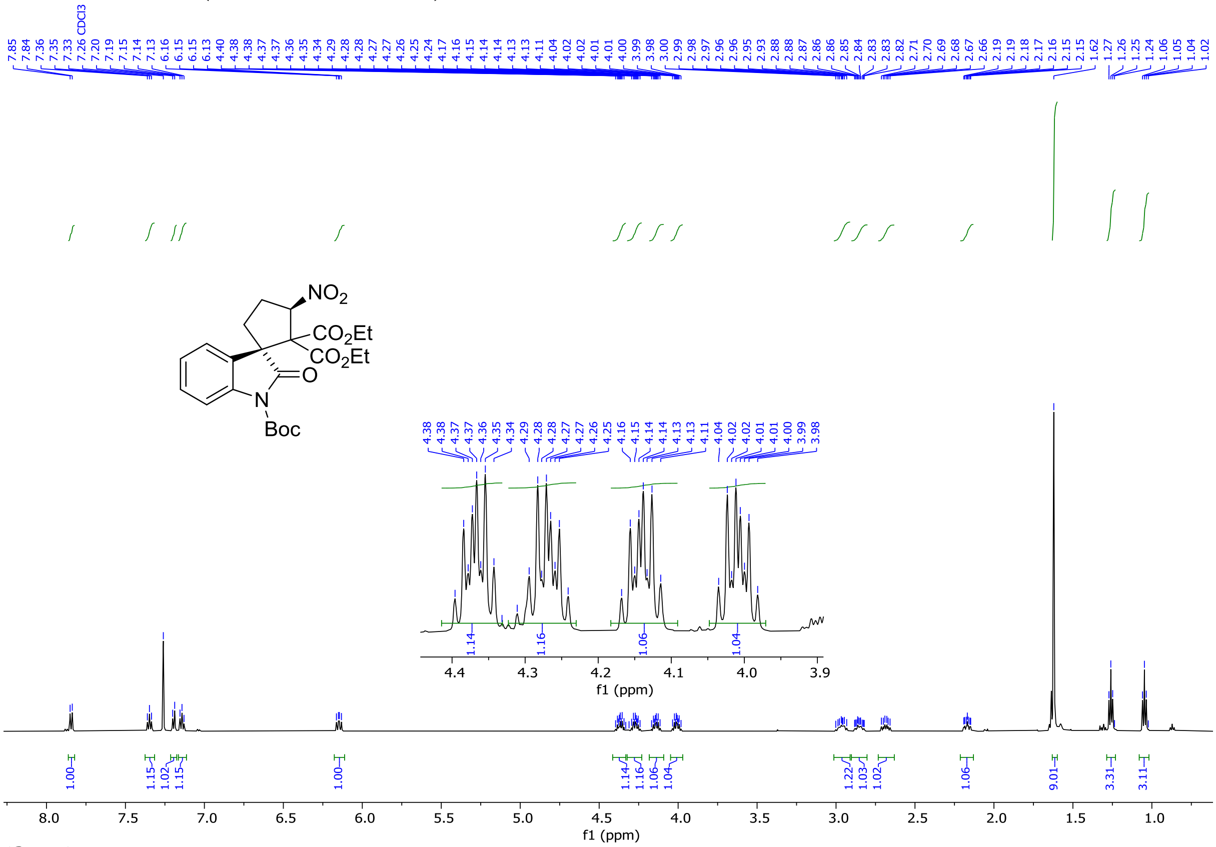

^{13}C{^{1}H} NMR of **3r** (101 MHz, $CDCl_3$)

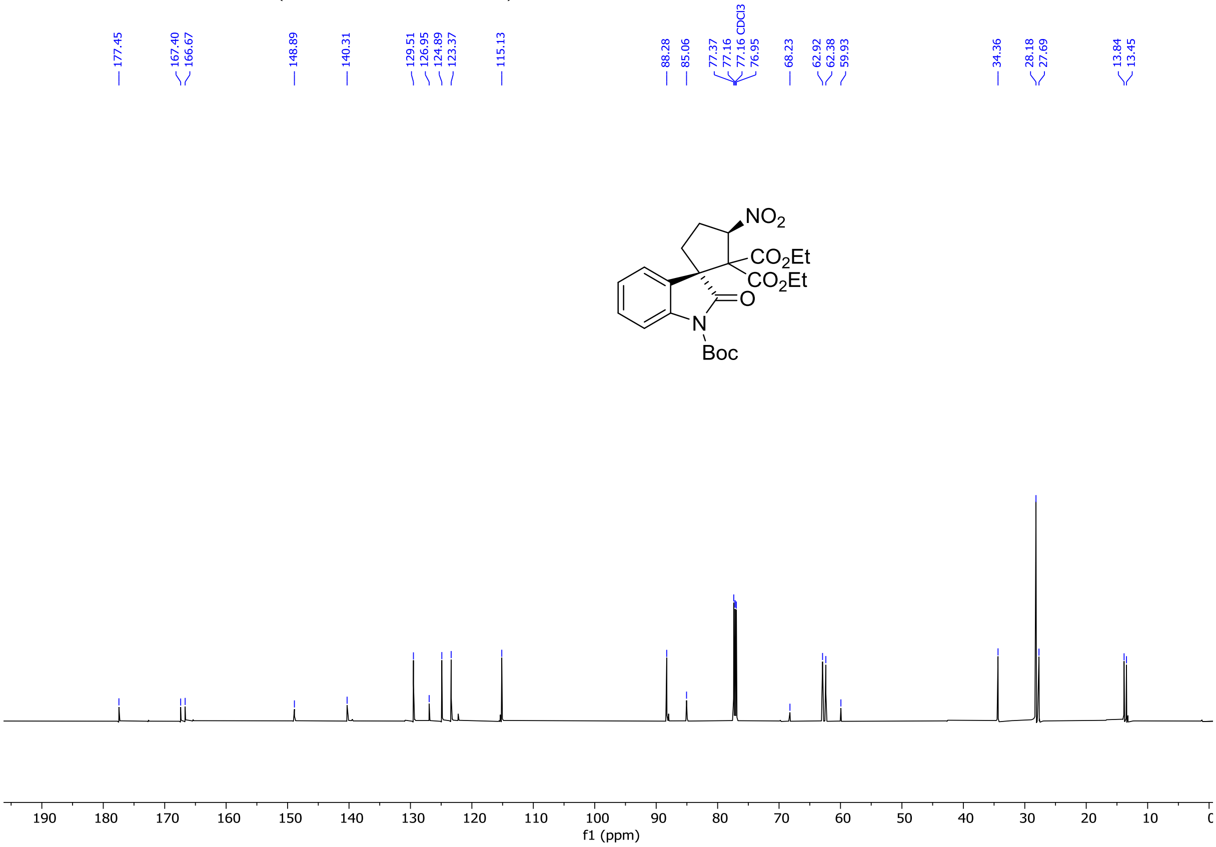

tert-Butyl 3-nitro-2'-oxo-2-(trifluoromethyl)spiro[cyclopentane-1,3'-indoline]-1'-carboxylate (3s)

^{1}H NMR of **3s** (400 MHz, $CDCl_3$)

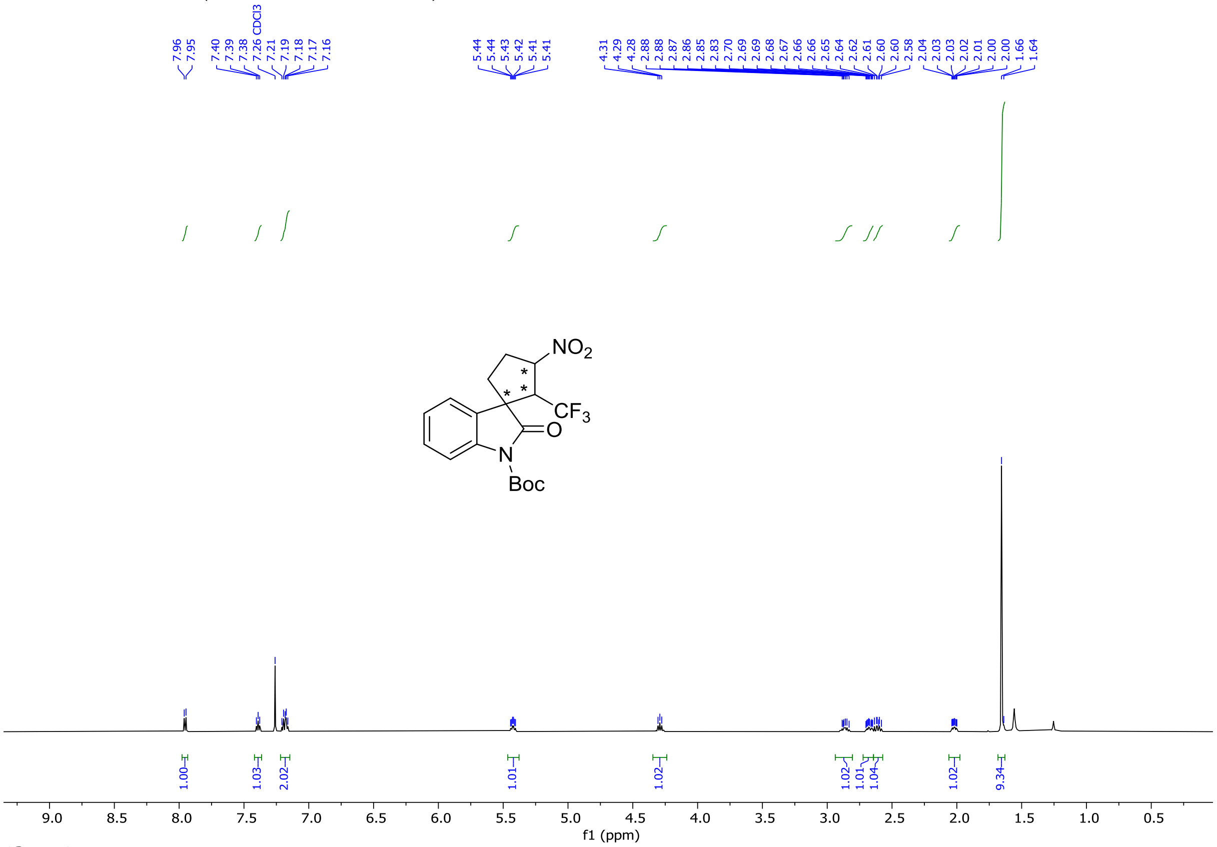

^{13}C{^{1}H} NMR of **3s** (101 MHz, $CDCl_3$)

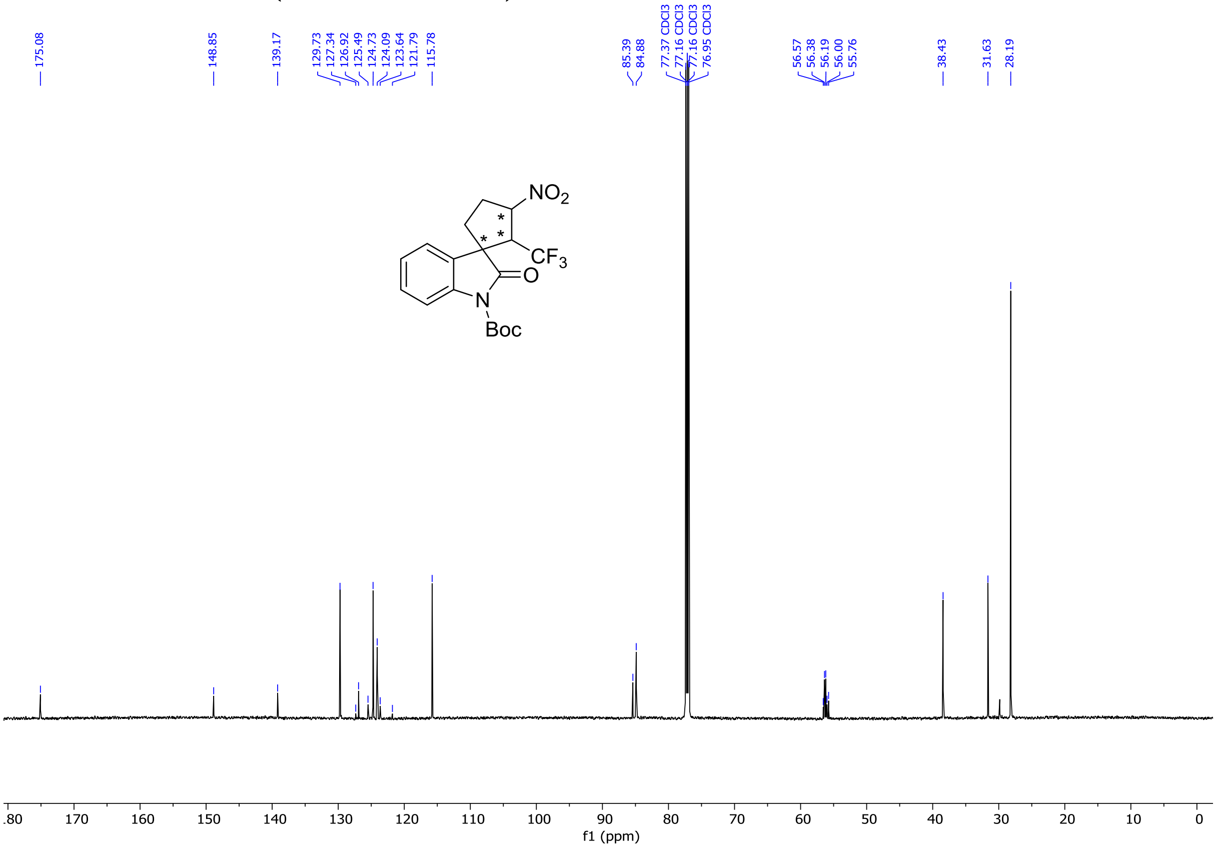

^{19}F NMR of **3s** (376 MHz MHz, $CDCl_3$)

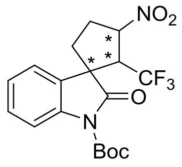

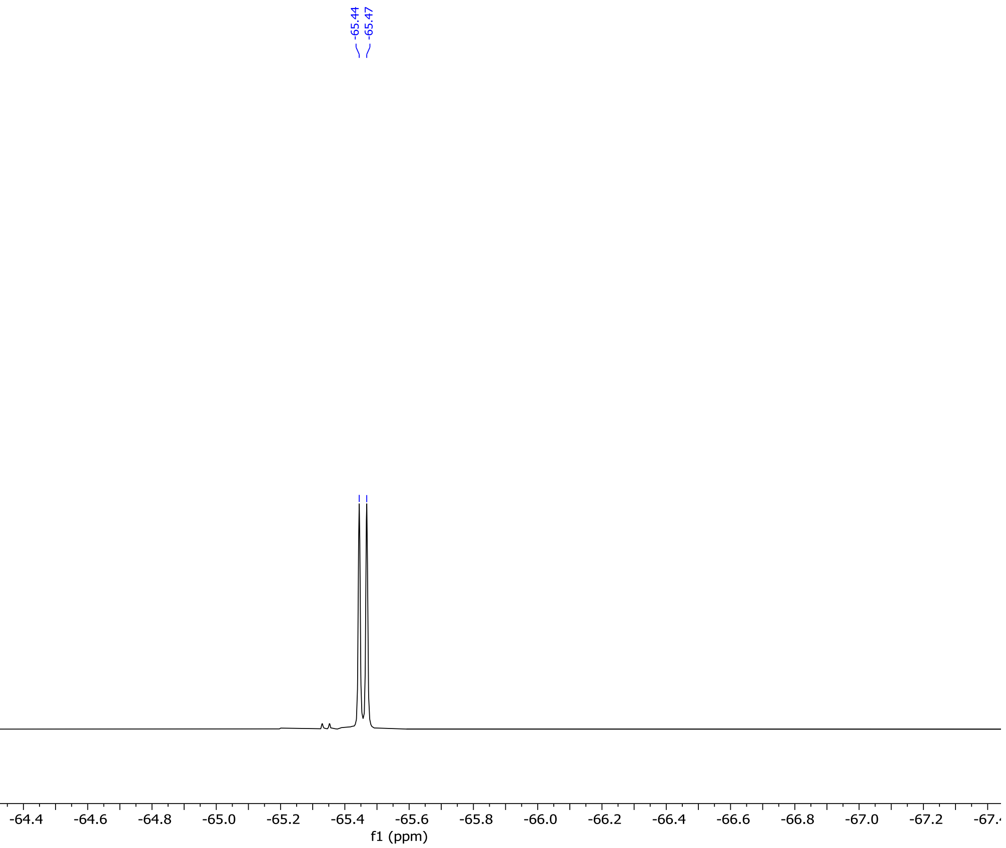

tert-Butyl 3-nitro-2'-oxo-2-(trifluoromethyl)spiro[cyclopentane-1,3'-indoline]-1'-carboxylate (4s)

^{1}H NMR of **4s** (400 MHz, $CDCl_3$)

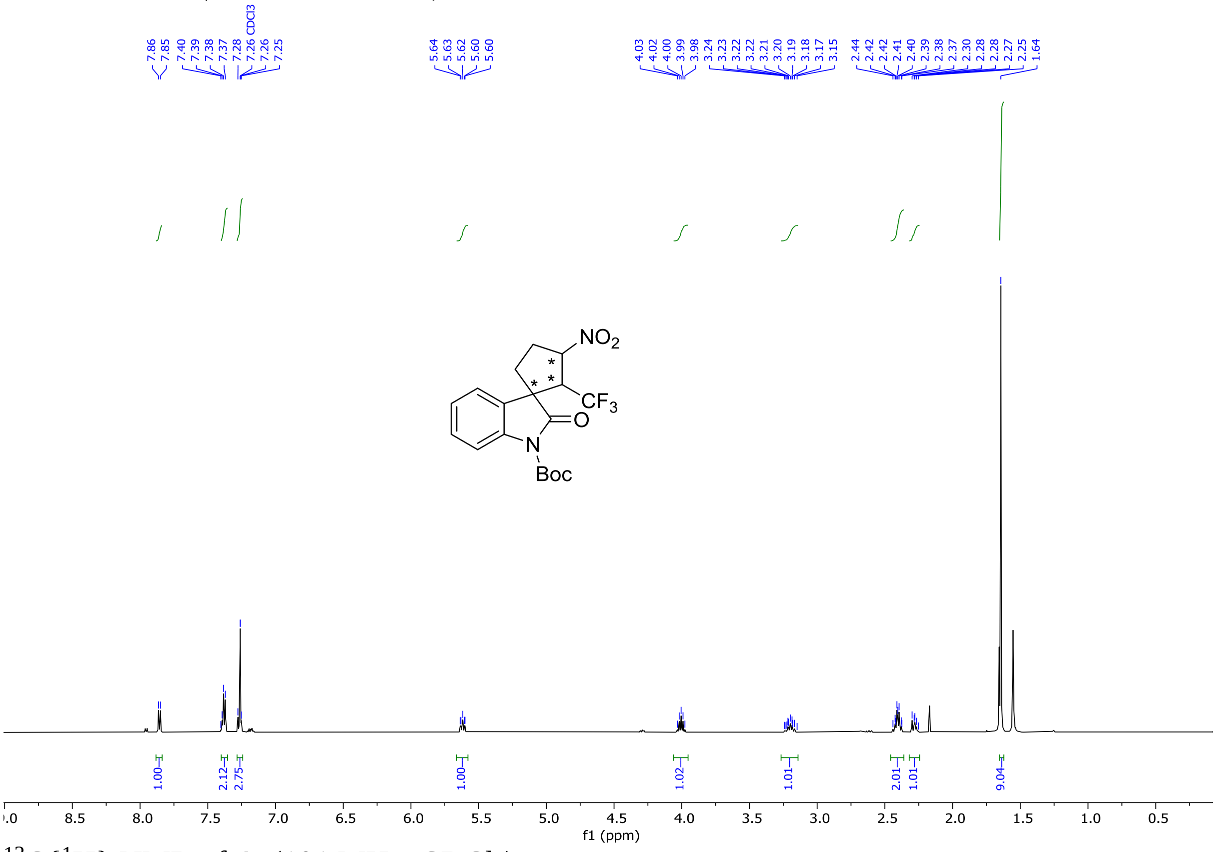

^{13}C{^{1}H} NMR of **4s** (101 MHz, $CDCl_3$)

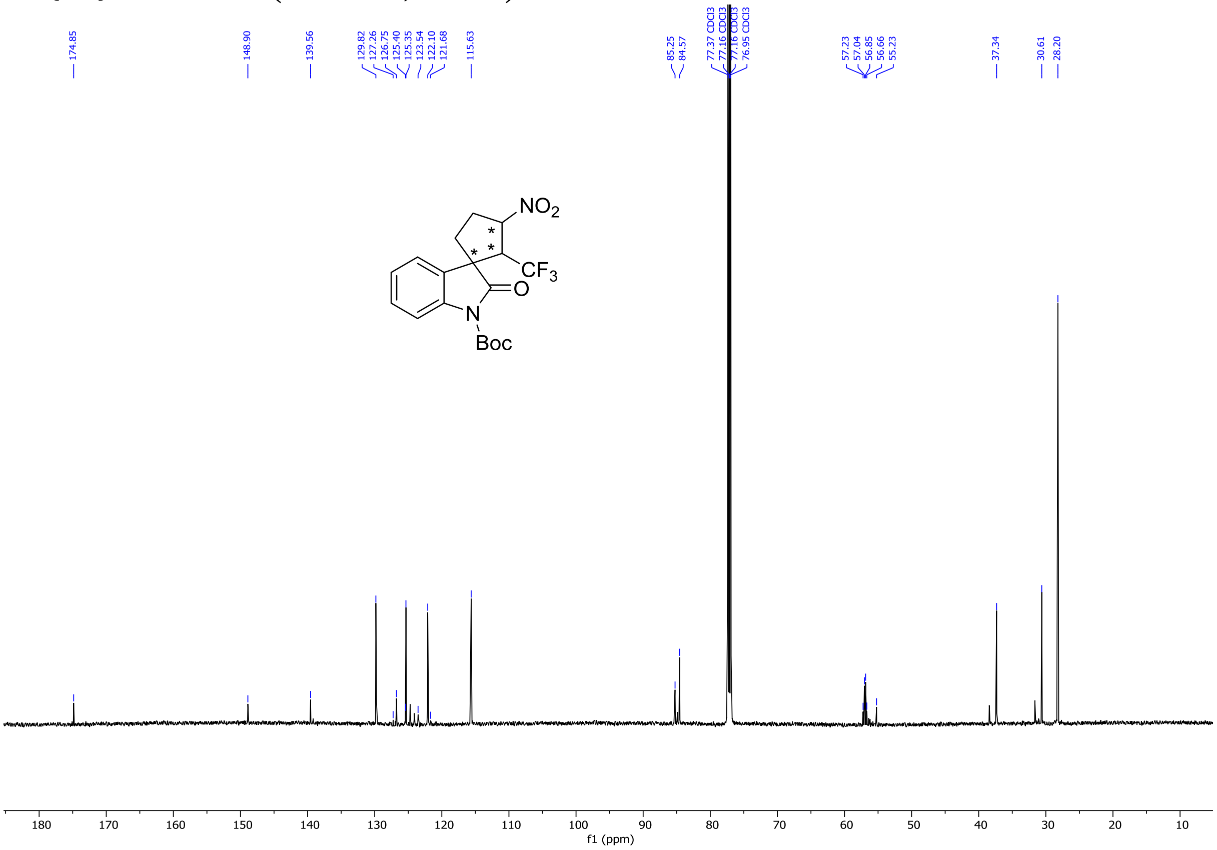

^{19}F NMR of **4s** (376 MHz MHz, $CDCl_3$)

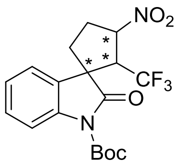

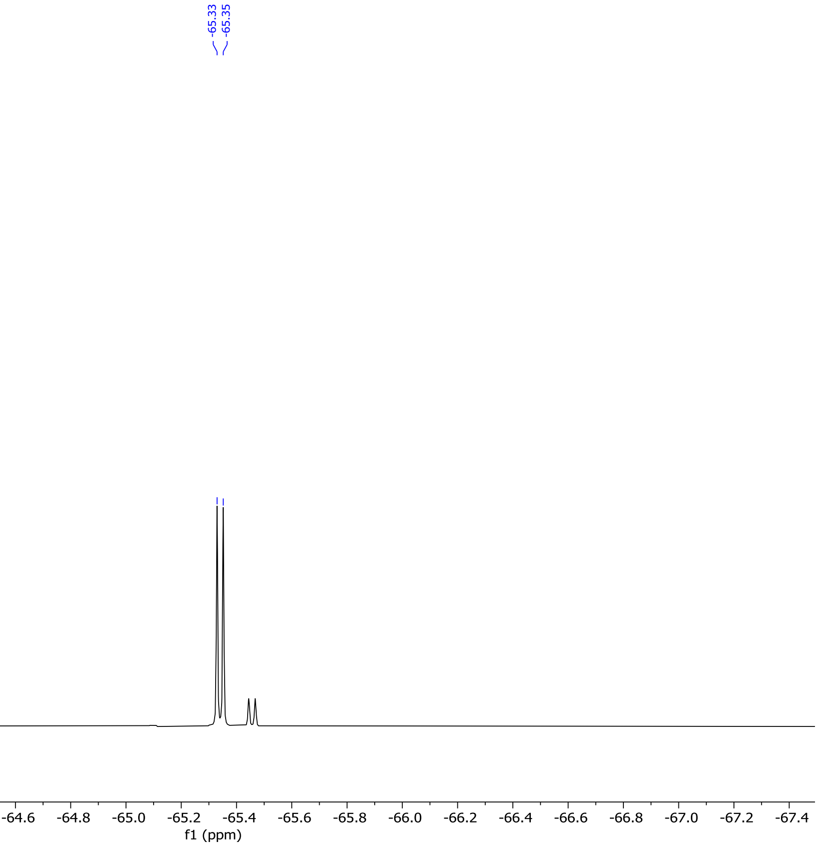

***tert*-Butyl 2-benzoyl-2'-oxospiro[cyclopentane-1,3'-indolin]-2-ene-1'-carboxylate (5t)**

^{1}H NMR of **5t** (400 MHz, $CDCl_3$)

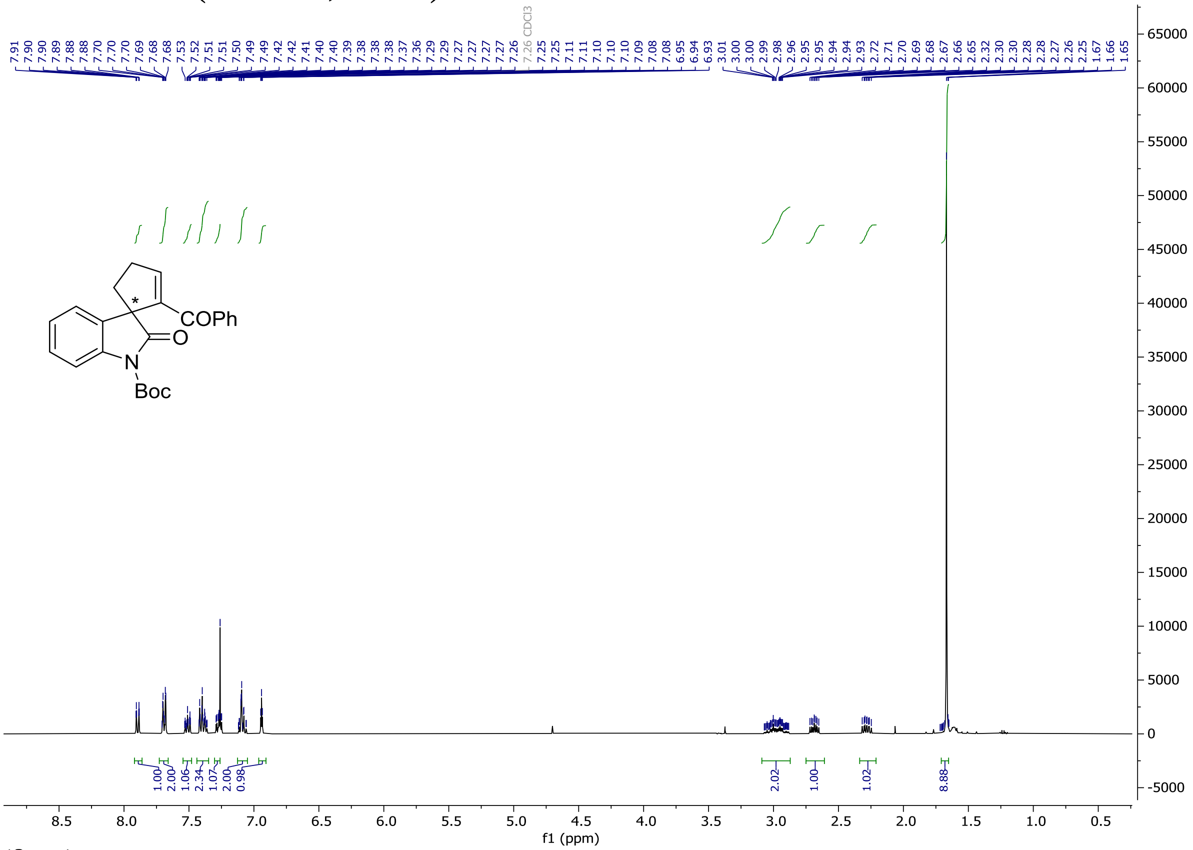

^{13}C{^{1}H} NMR of **5t** (101 MHz, $CDCl_3$)

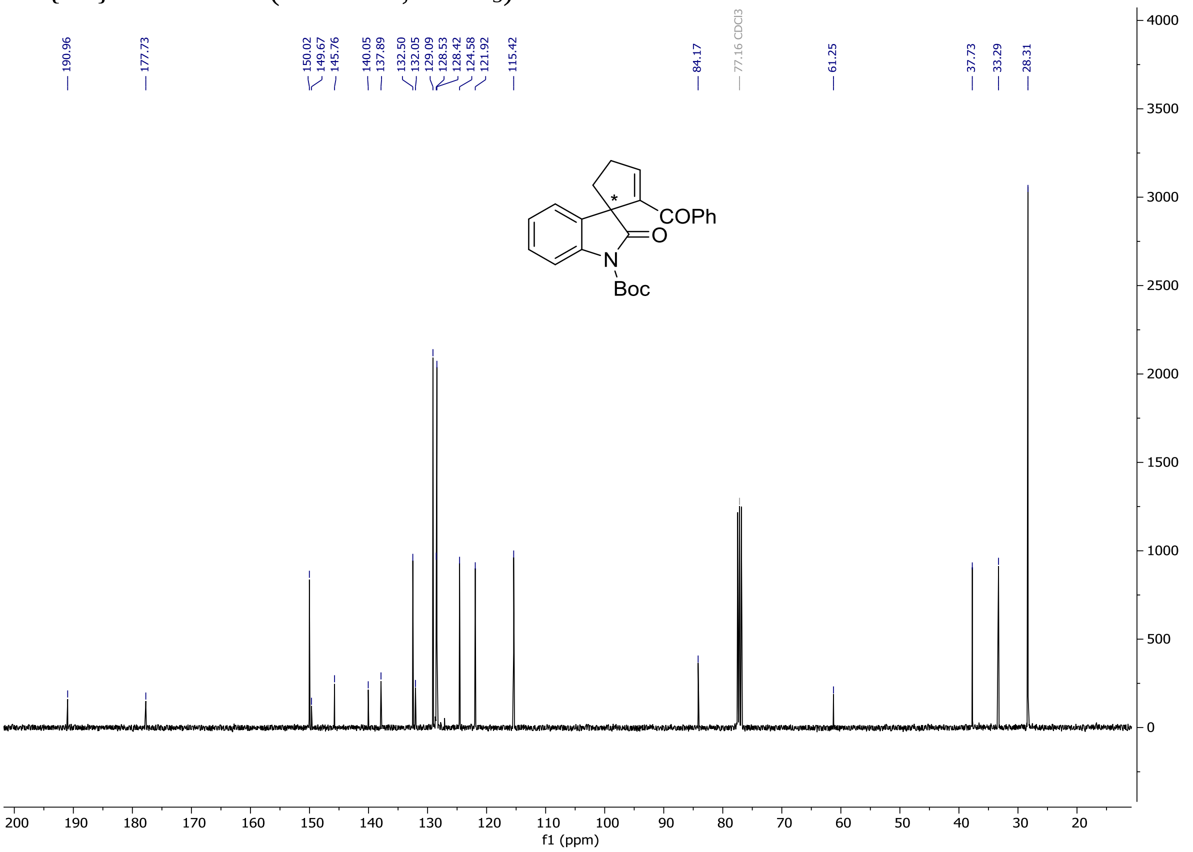

tert-Butyl (1*R*,2*R*,3*R*)-3-nitro-2'-oxo-2-phenylspiro[cyclopentane-1,3'-indoline]-1'-carboxylate (3v)

^{1}H NMR of **3v** (400 MHz, $CDCl_3$)

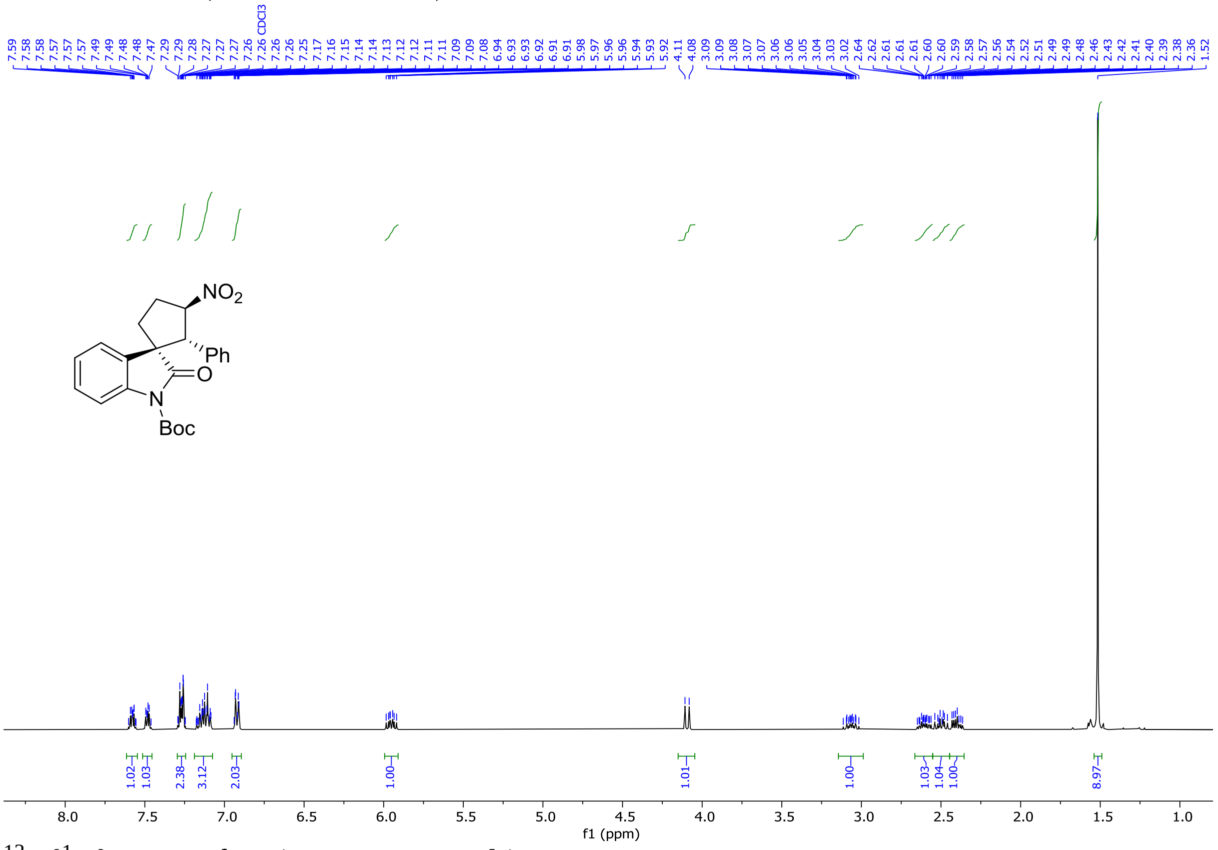

^{13}C{^{1}H} NMR of **3v** (101 MHz, $CDCl_3$)

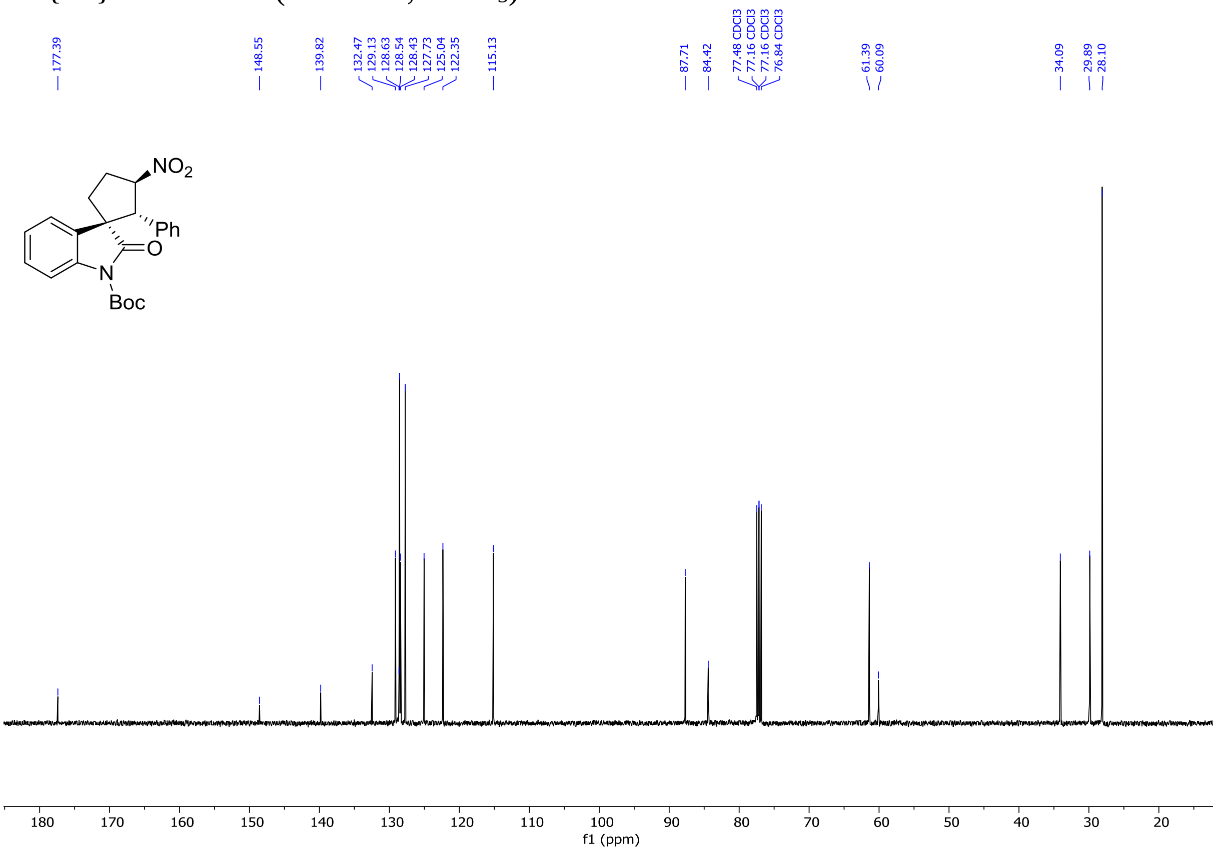

1'-(*tert*-Butyl) 2-ethyl 3-nitro-2'-oxospiro[cyclopropane-1,3'-indoline]-1',2-dicarboxylate (6/6′)

^{1}H NMR of **6/6′** (400 MHz, $CDCl_3$)

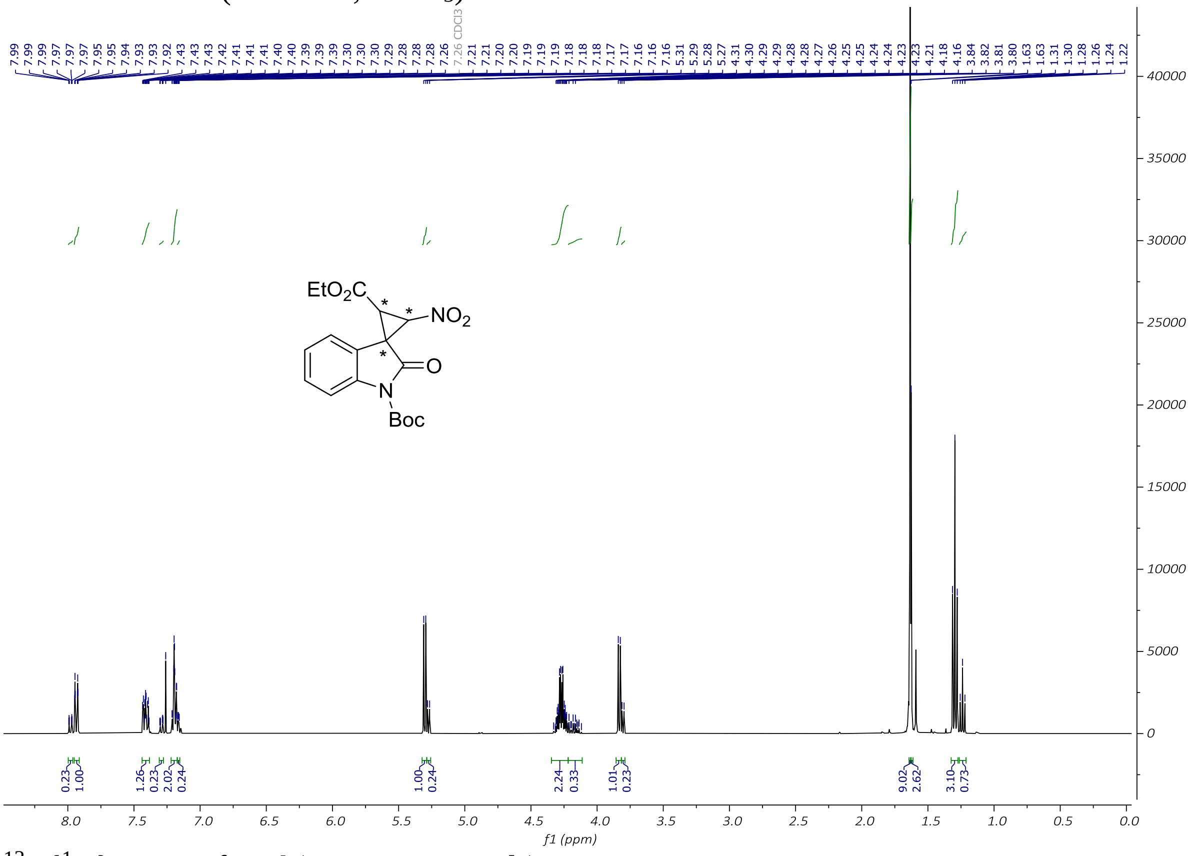

^{13}C{^{1}H} NMR of **6/6′** (101 MHz, $CDCl_3$)

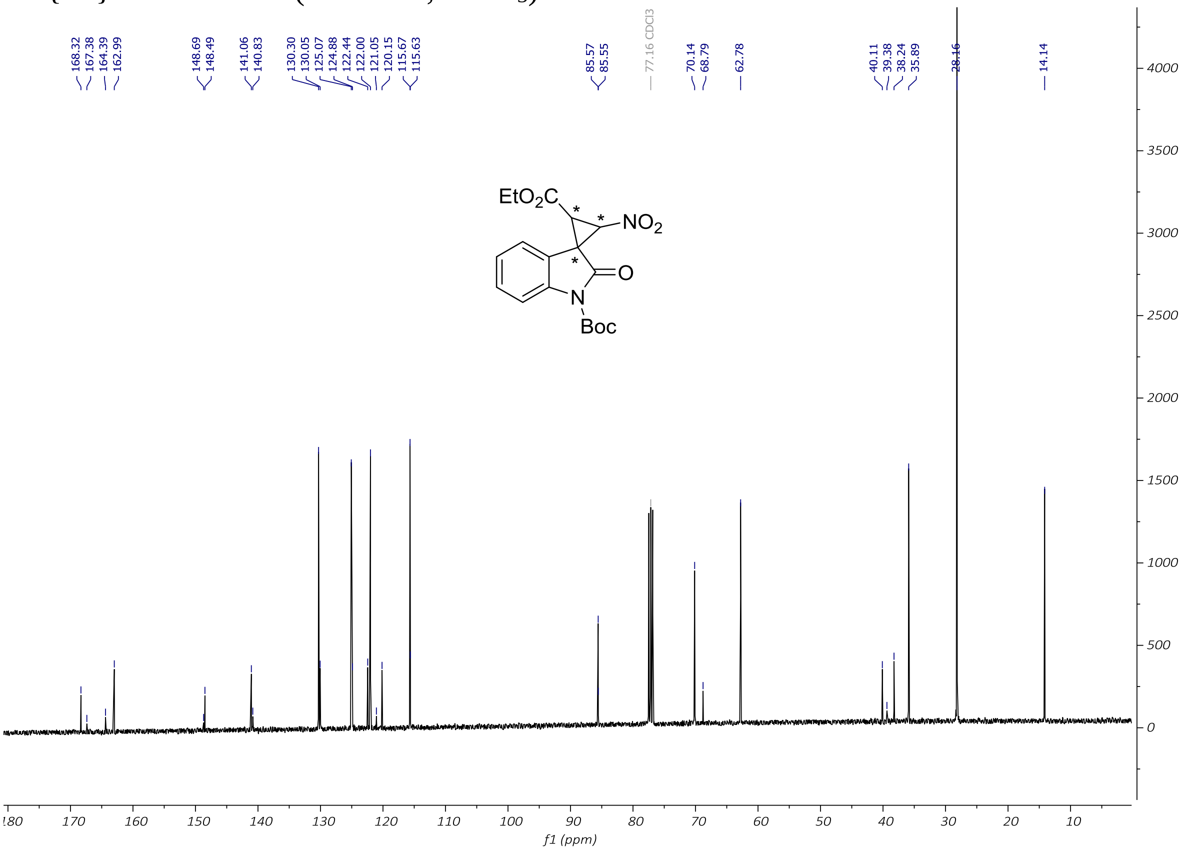

Ethyl (1*R*,2*R*,3*R*)-3-nitro-2'-oxospiro[cyclopentane-1,3'-indoline]-2-carboxylate (3f)

^{1}H NMR of **3f** (600 MHz, $CDCl_3$)

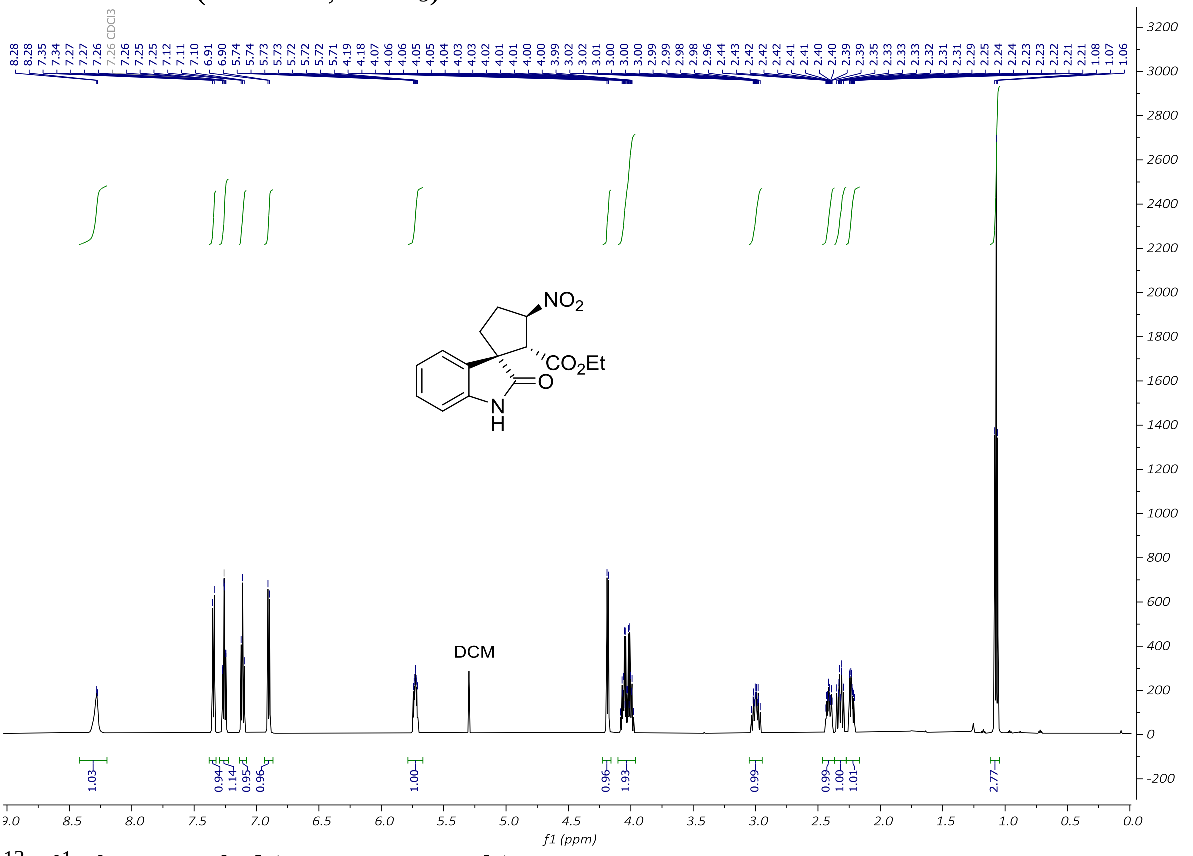

$^{13}C\{^1H\}$ NMR of **3f** (151 MHz, $CDCl_3$)

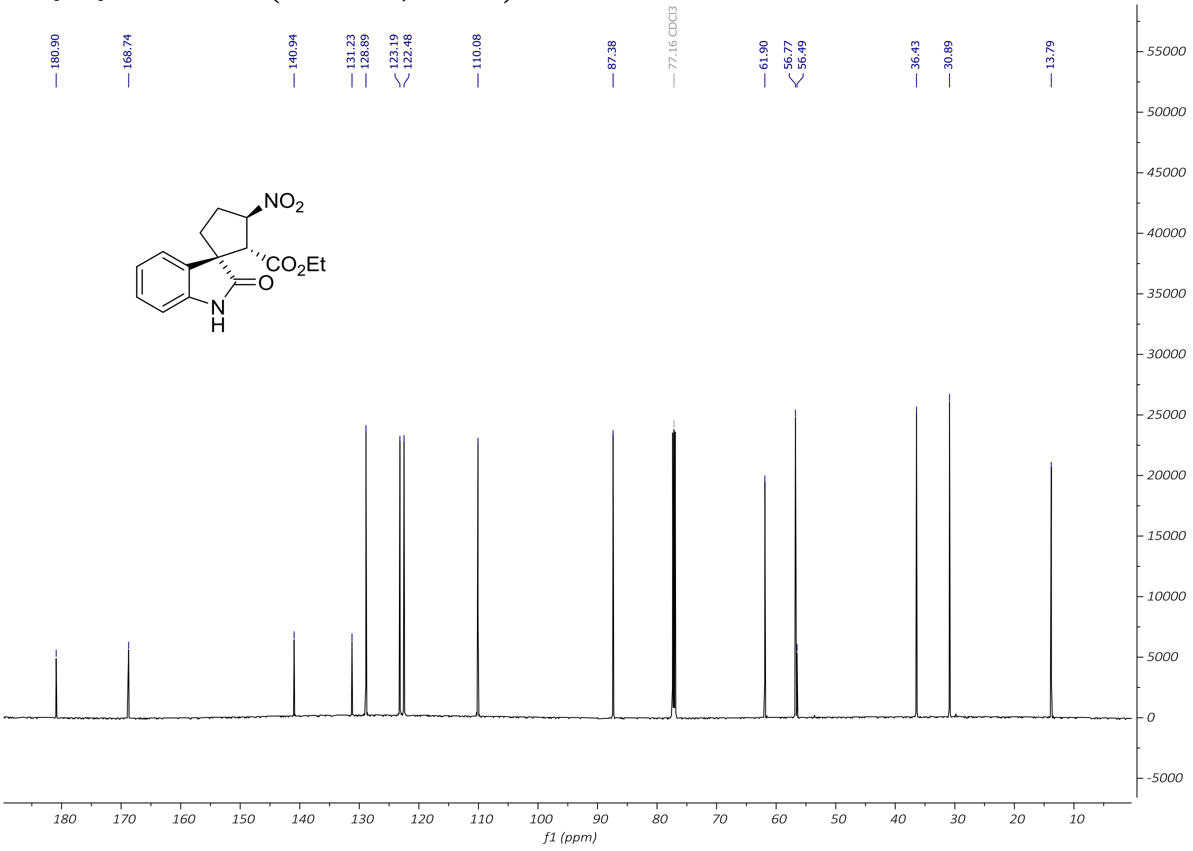

1'-(*tert*-Butyl) 2-ethyl (*S*)-2'-oxospiro[cyclopentane-1,3'-indolin]-2-ene-1',2-dicarboxylate (5a)

^{1}H NMR of **5a** (600 MHz, $CDCl_3$)

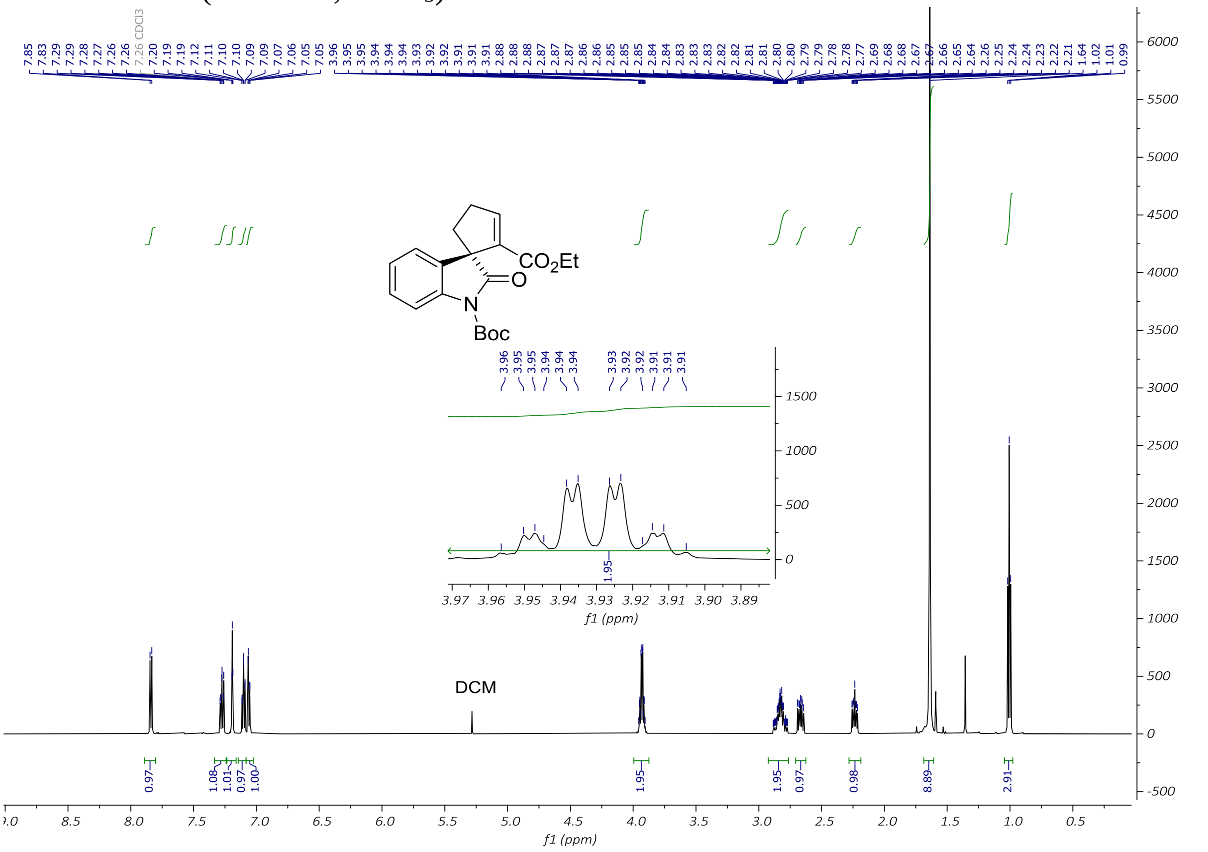

$^{13}C\{^1H\}$ NMR of **5a** (151 MHz, $CDCl_3$)

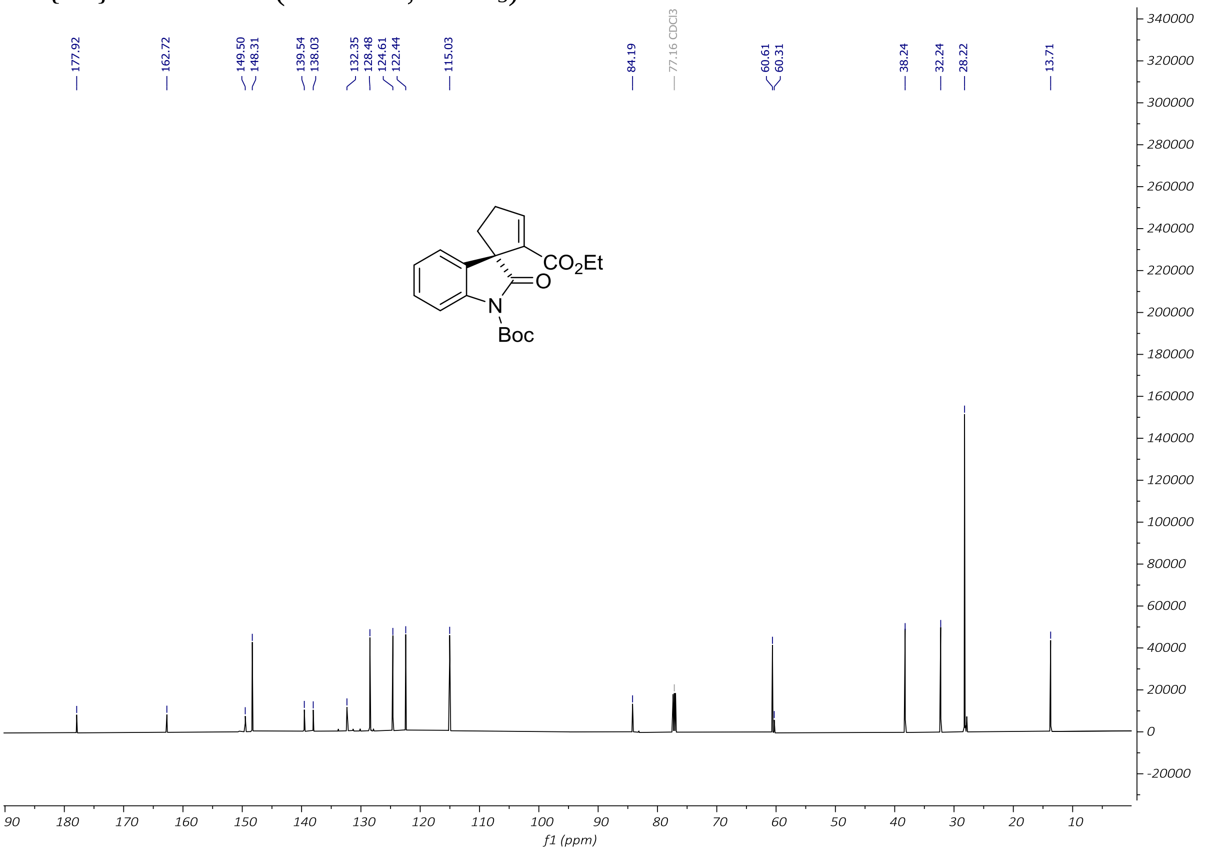

1'-(*tert*-Butyl) 2-ethyl (1*R*,2*S*)-2'-oxospiro[cyclopentane-1,3'-indoline]-1',2-dicarboxylate (9/9′)

^{1}H NMR of **9/9′** (400 MHz, $CDCl_3$)

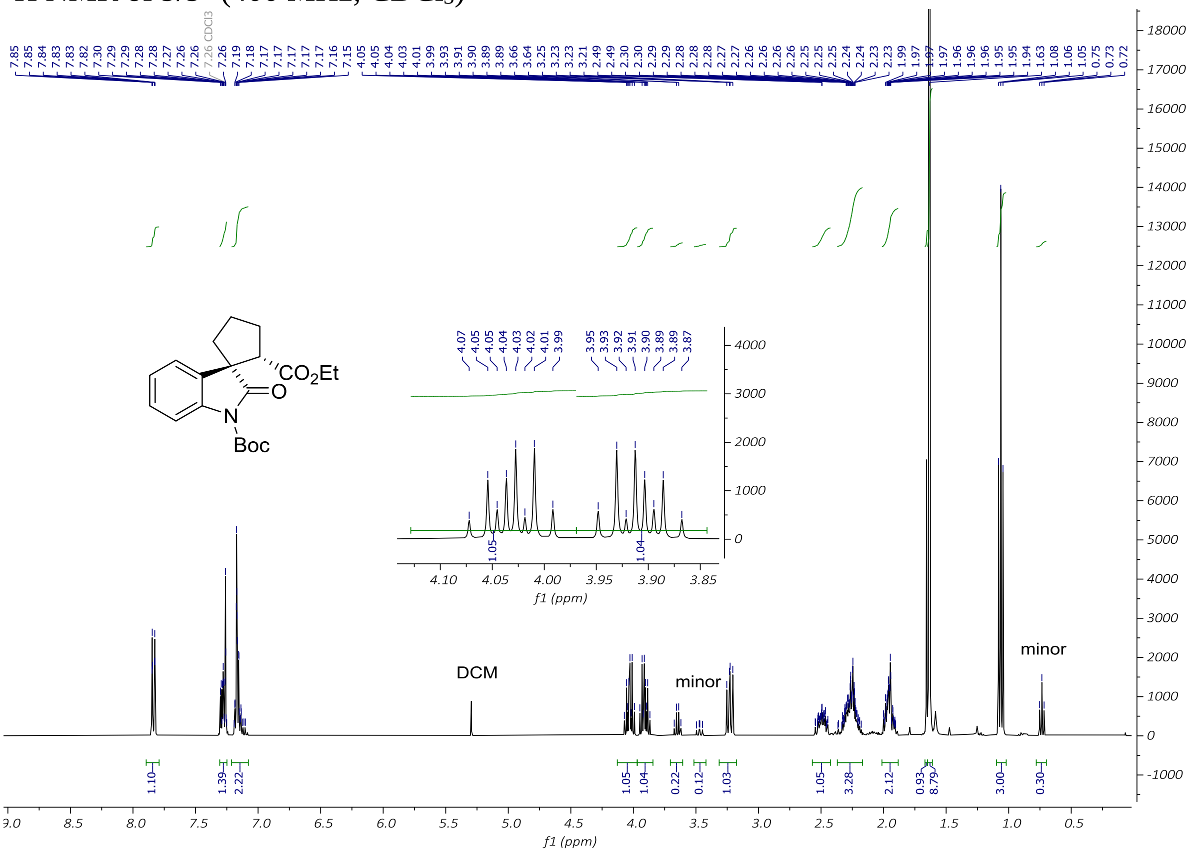

^{13}C{^{1}H} NMR of **9/9′** (101 MHz, $CDCl_3$)

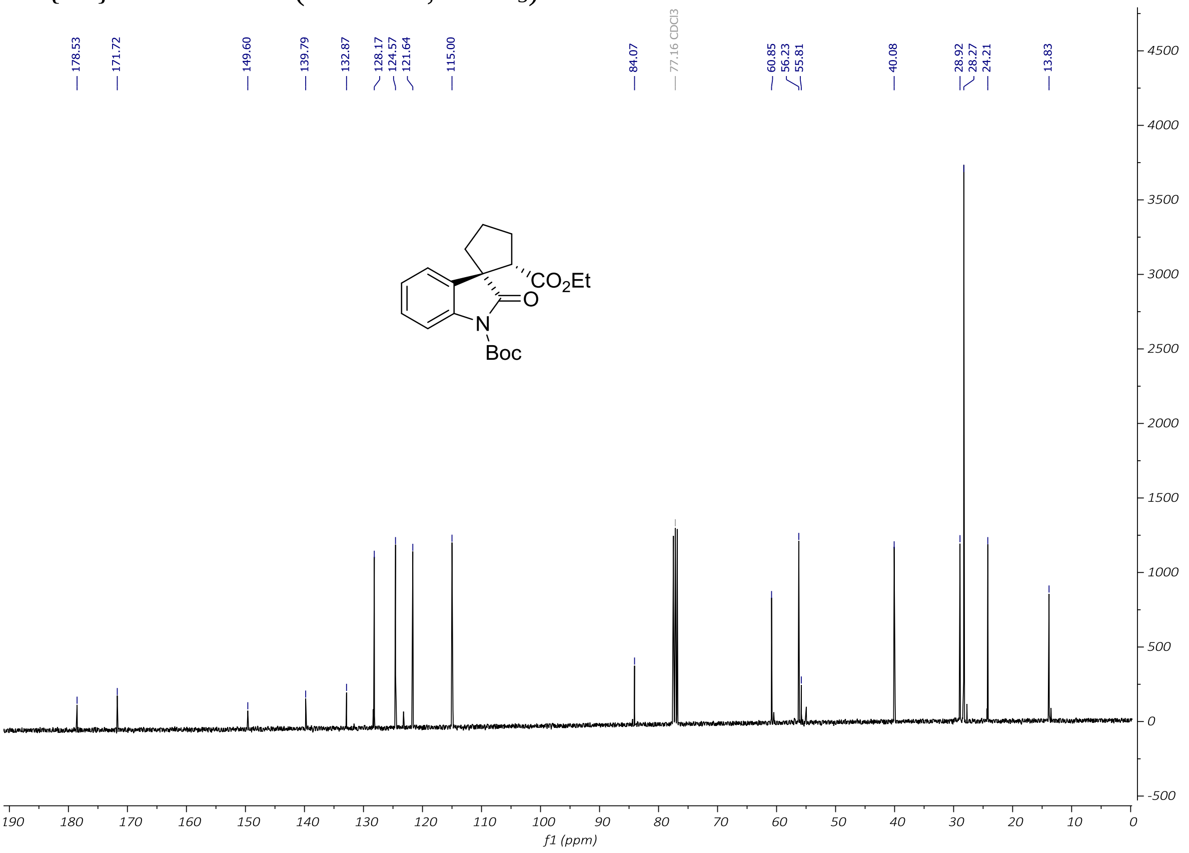

***tert*-Butyl (*S*)-2-(hydroxymethyl)-2'-oxospiro[cyclopentane-1,3'-indolin]-2-ene-1'-carboxylate (10)**

^{1}H NMR of **10** (400 MHz, $CDCl_3$)

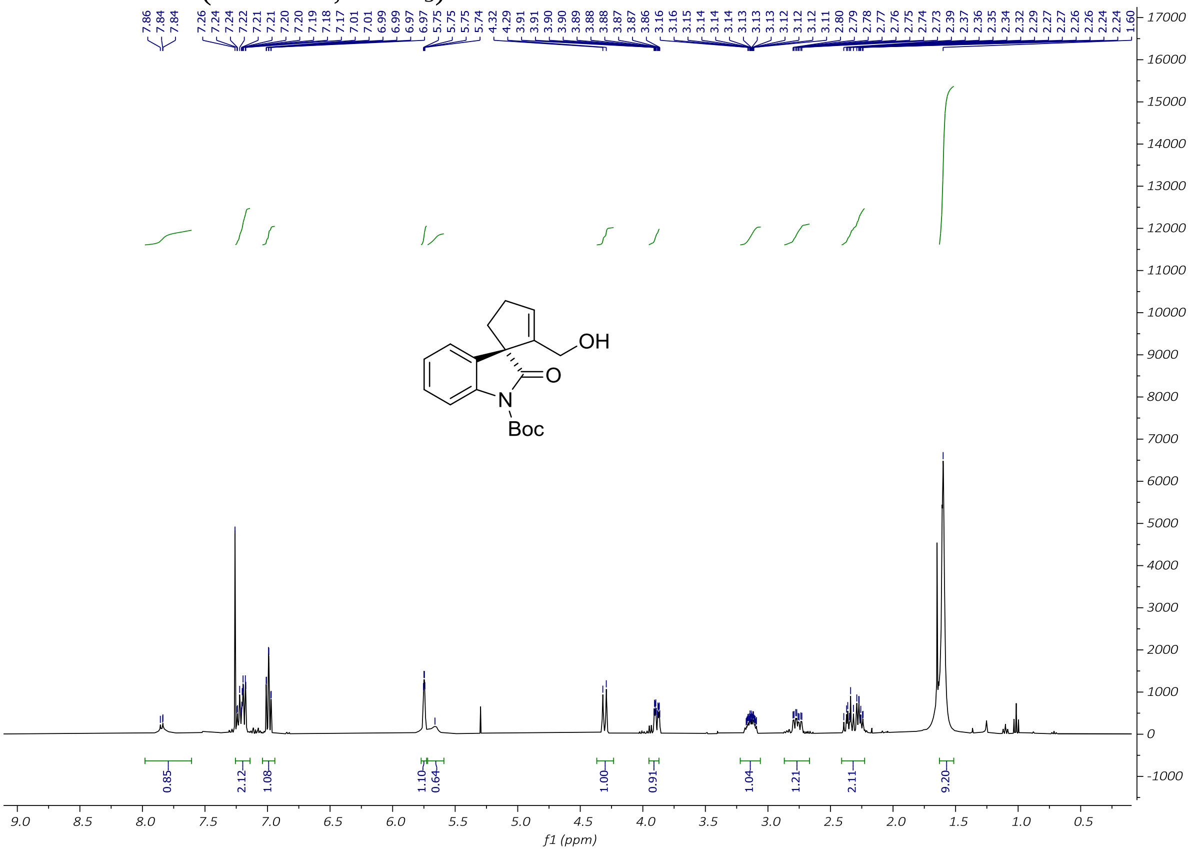

^{13}C{^{1}H} NMR of **10** (101 MHz, $CDCl_3$)

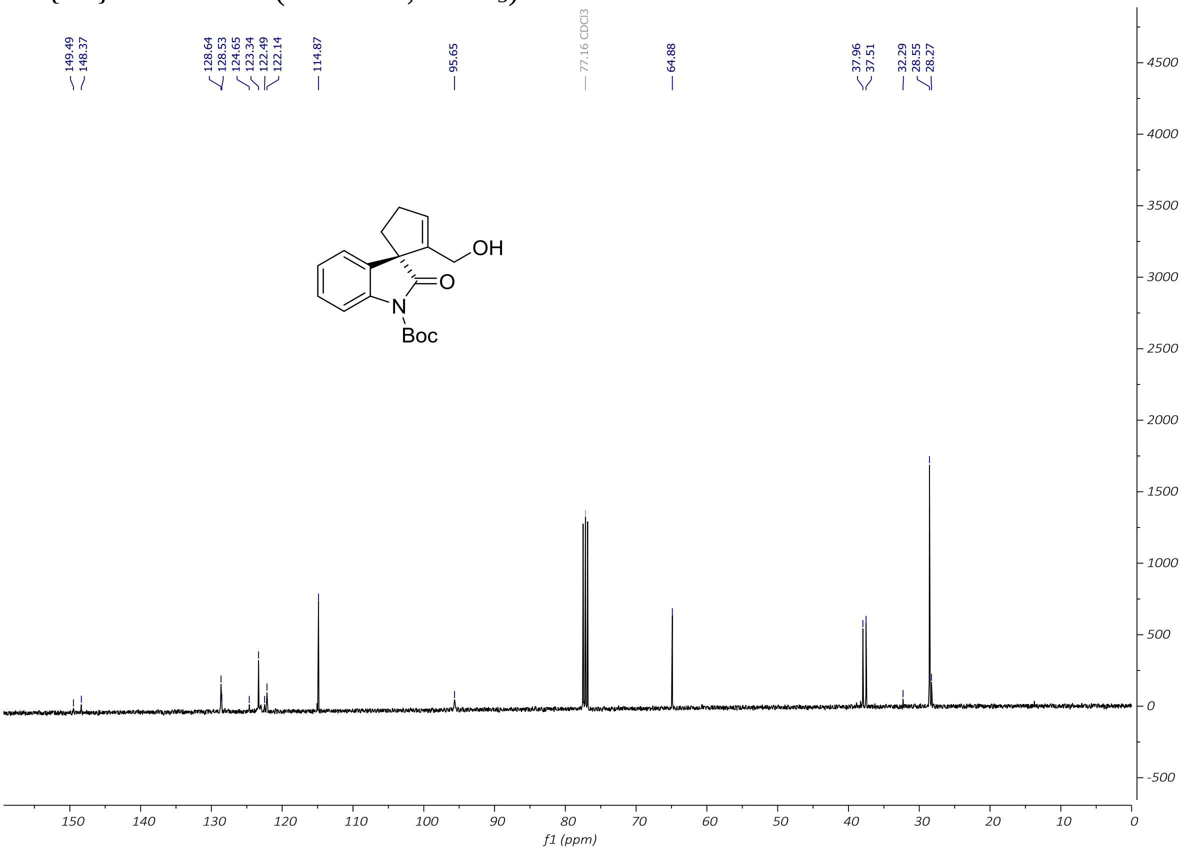

Chiral HPLC

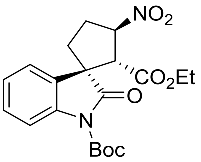

Conditions: IA column
mobile phase: *n*-heptane/*i*-PrOH = 80/20
λ = 208 nm, V = 1.0 ml/min, t = 25 °C
for **3a**: t_R = 4.4 min (minor), t_R = 5.0 min (major).

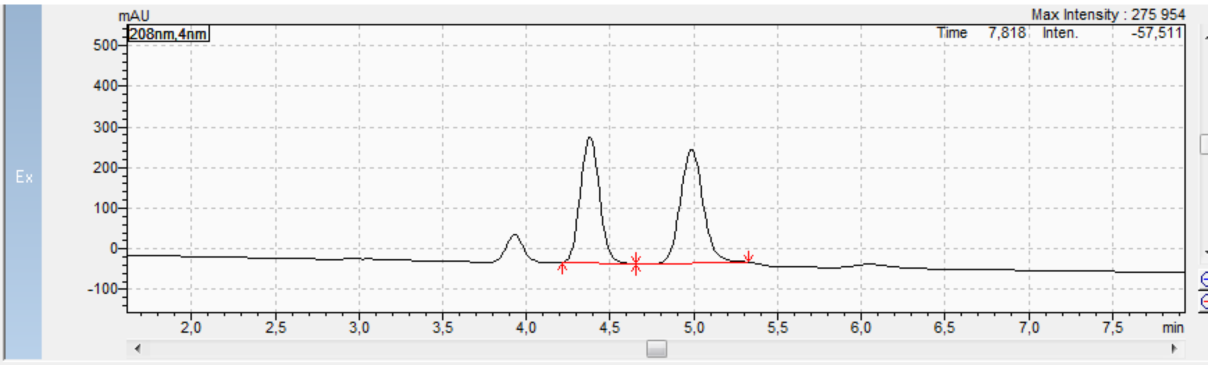

Results View - Peak Table

Peak Table | Compound | Group | Calibration Curve

Peak#	Ret. Time	Conc.	Area	Height	Similarity Index	Mark	Peak Start	Peak End	Area%
1	4,380	48,643	2476157	311319	0,000000	M	4,213	4,651	48,643
2	4,987	51,357	2614290	277257	0,000000	M	4,651	5,323	51,357
Total		100,000	5090447	588576					100,000

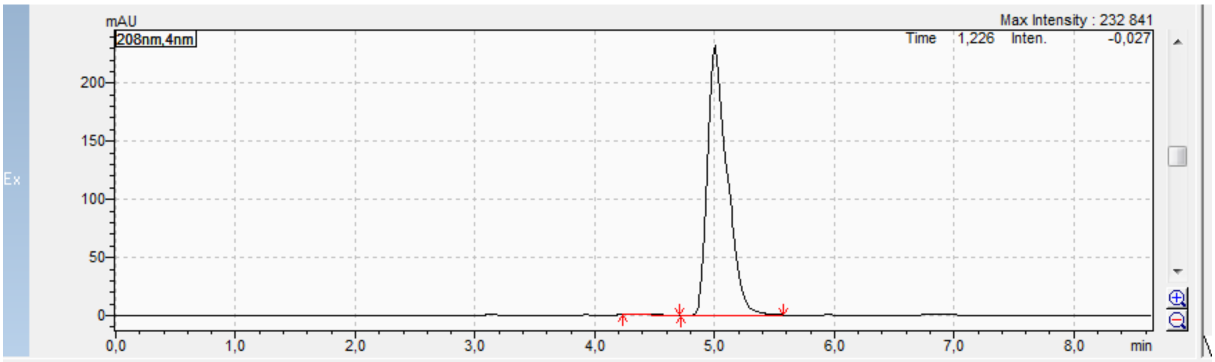

Results View - Peak Table

Peak Table | Compound | Group | Calibration Curve

Peak#	Ret. Time	Conc.	Area	Height	Similarity Index	Mark	Peak Start	Peak End	Area%
1	4,450	0,311	8324	856	0,000000	M	4,235	4,704	0,311
2	5,003	99,689	2669359	232477	0,000000	M	4,715	5,579	99,689
Total		100,000	2677683	233333					100,000

for **3a**: *ee* = 99%

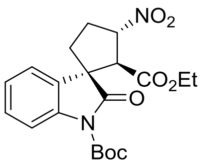

Conditions: IA column
mobile phase: *n*-heptane/*i*-PrOH = 80/20
λ = 216 nm, V = 1.0 ml/min, t = 25 °C
for *ent*-**3a**: t_R = 4.4 min (minor), t_R = 5.0 min (major).

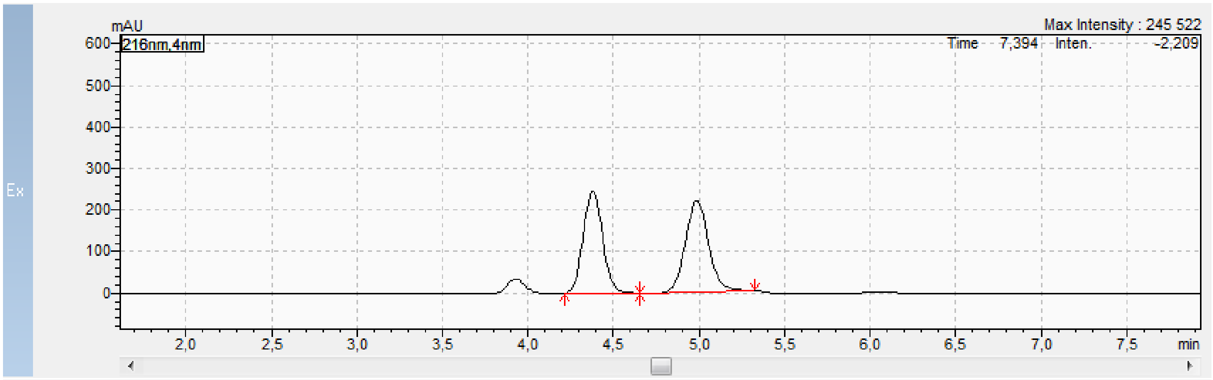

Results View - Peak Table

Peak Table | Compound | Group | Calibration Curve

Peak#	Ret. Time	Conc.	Area	Height	Similarity Index	Mark	Peak Start	Peak End	Area%
1	4,380	48,765	1951866	245730	0,000000	M	4,213	4,651	48,765
2	4,987	51,235	2050751	217952	0,000000	M	4,651	5,323	51,235
Total		100,000	4002617	463682					100,000

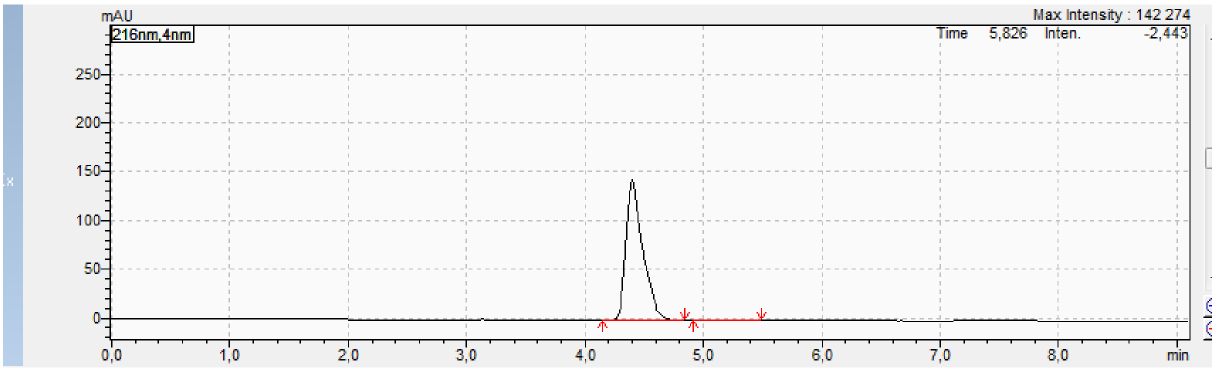

Results View - Peak Table

Peak Table | Compound | Group | Calibration Curve

Peak#	Ret. Time	Conc.	Area	Height	Similarity Index	Mark	Peak Start	Peak End	Area%
1	4,400	99,709	1424395	144277	0,000000	M	4,149	4,843	99,709
2	5,071	0,291	4164	400	0,000000	M	4,917	5,493	0,291
Total		100,000	1428558	144677					100,000

for *ent*-**3a**: *ee* = 99%

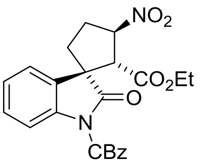

Conditions: IB column
mobile phase: *n*-heptane/*i*-PrOH = 80/20
λ = 208 nm, V = 1.0 ml/min, t = 25 °C
for **3b**: t_R = 11.1 min (minor), t_R = 12.9 min (major)

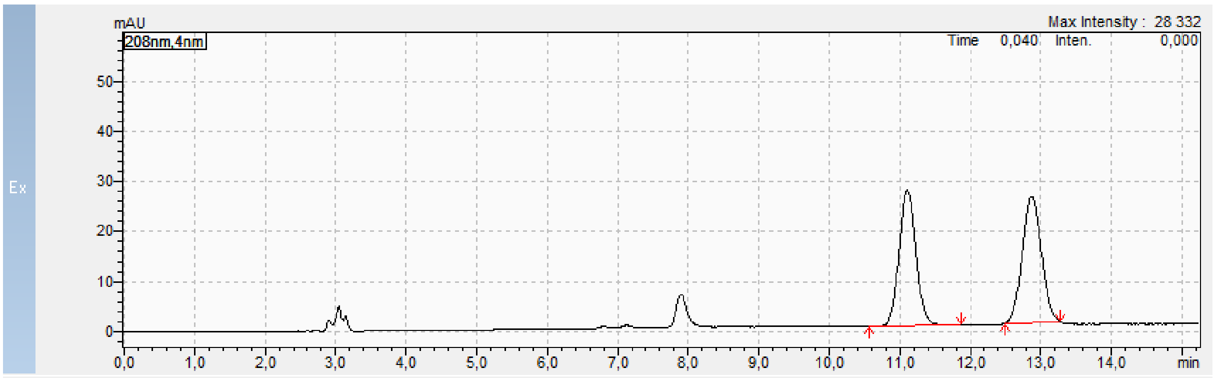

Results View - Peak Table

Peak Table | Compound | Group | Calibration Curve

Peak#	Ret. Time	Conc.	Area	Height	Similarity Index	Mark	Peak Start	Peak End	Area%
1	11,106	48,923	453483	27162	0,000000	M	10,560	11,861	48,923
2	12,874	51,077	473447	25131	0,000000	M	12,501	13,280	51,077
Total		100,000	926930	52293					100,000

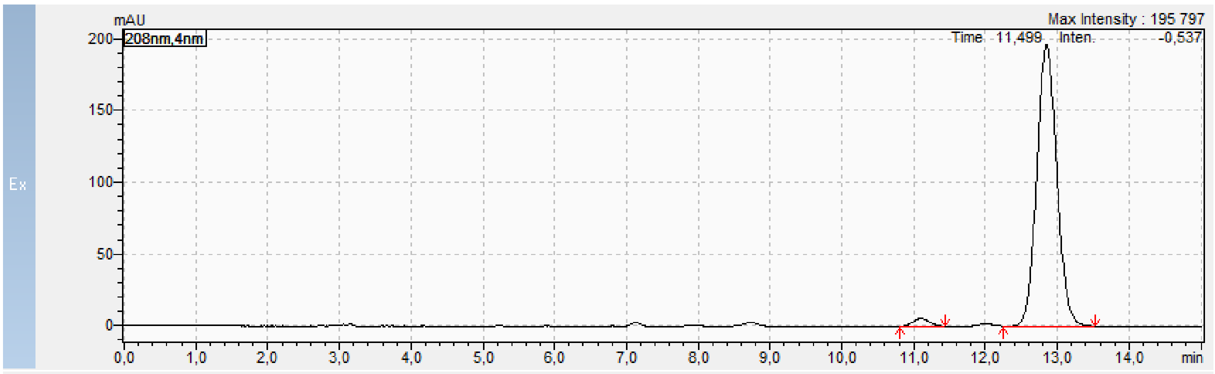

Results View - Peak Table

Peak Table | Compound | Group | Calibration Curve

Peak#	Ret. Time	Conc.	Area	Height	Similarity Index	Mark	Peak Start	Peak End	Area%
1	11,112	2,230	86530	5386	0,000000	M	10,805	11,435	2,230
2	12,852	97,770	3792938	196035	0,000000	M	12,245	13,525	97,770
Total		100,000	3879469	201421					100,000

for **3b**: *ee* = 96%

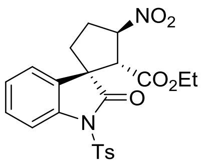

Conditions: IB column
mobile phase: heptane/*i*-PrOH = 90/10
λ = 207 nm, V = 1.0 ml/min, t = 25 °C
for **3c**: t_R = 10.6 min (minor), t_R = 11.9 min (major)

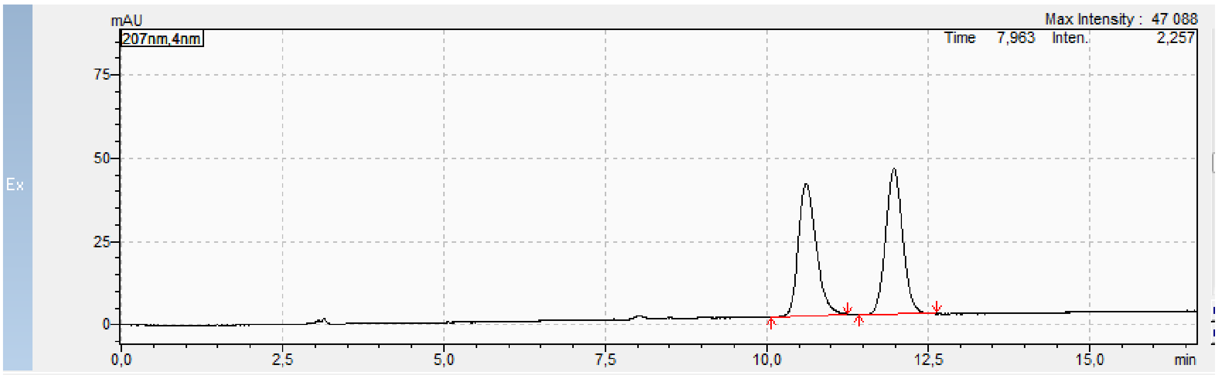

Results View - Peak Table

Peak Table | Compound | Group | Calibration Curve

Peak#	Ret. Time	Conc.	Area	Height	Similarity Index	Mark	Peak Start	Peak End	Area%
1	10,614	48,534	761135	39552	0,000000	M	10,059	11,264	48,534
2	11,975	51,466	807130	43897	0,000000	M	11,424	12,640	51,466
Total		100,000	1568266	83449					100,000

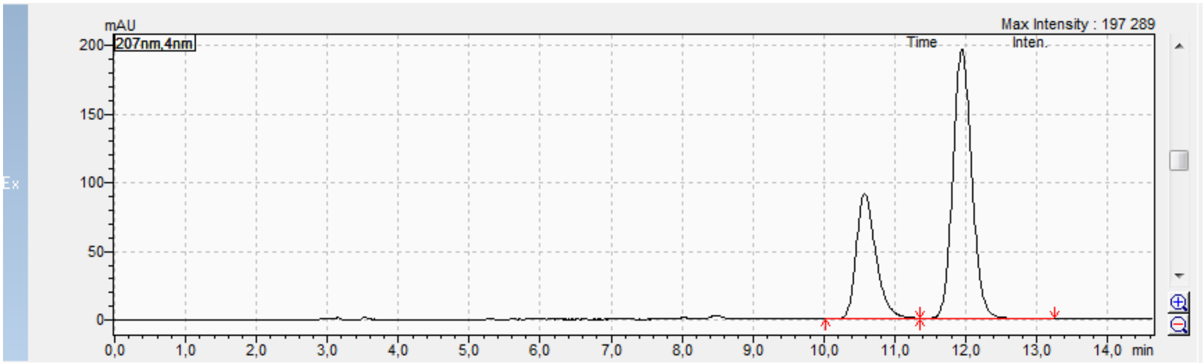

Results View - Peak Table

Peak Table | Compound | Group | Calibration Curve

Peak#	Ret. Time	Conc.	Area	Height	Similarity Index	Mark	Peak Start	Peak End	Area%
1	10,575	32,488	1738790	90742	0,000000	M	10,027	11,349	32,488
2	11,948	67,512	3613230	195895	0,000000	M	11,349	13,248	67,512
Total		100,000	5352020	286637					100,000

for **3c**: *ee* = 35%

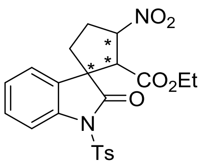

Conditions: IB column
mobile phase: *n*-heptane/*i*-PrOH = 80/20
λ = 207 nm, V = 1.0 ml/min, t = 25 °C
for **4c**: t_R = 14.9 min (major), t_R = 20.4 min (minor)
for **5c**: t_R = 8.5 min (minor), t_R = 9.0 min (major)

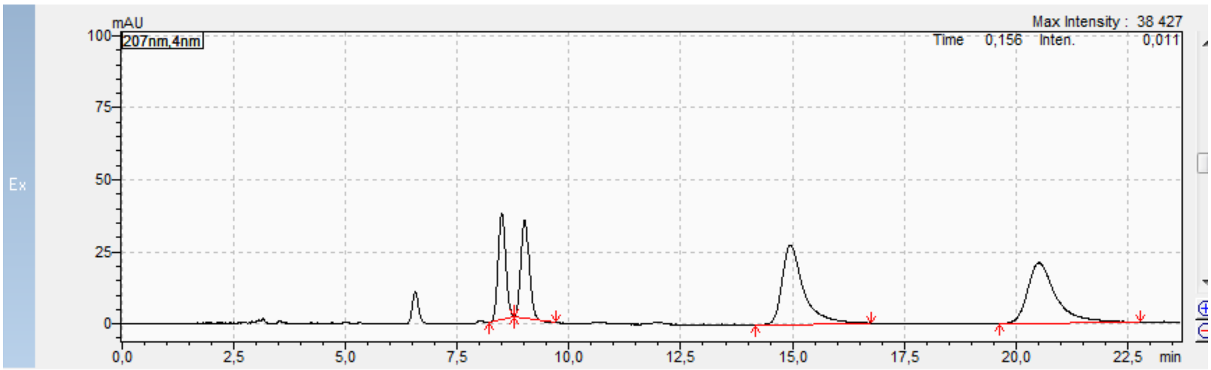

Results View - Peak Table

Peak Table | Compound | Group | Calibration Curve

Peak#	Ret. Time	Conc.	Area	Height	Similarity Index	Mark	Peak Start	Peak End	Area%
1	8,496	16,497	450256	37006	0,000000	M	8,203	8,768	16,497
2	9,010	16,327	445619	33997	0,000000	M	8,768	9,696	16,327
3	14,952	33,582	916560	27303	0,000000	M	14,176	16,757	33,582
4	20,531	33,593	916855	20901	0,000000	M	19,627	22,773	33,593
Total		100,000	2729290	119206					100,000

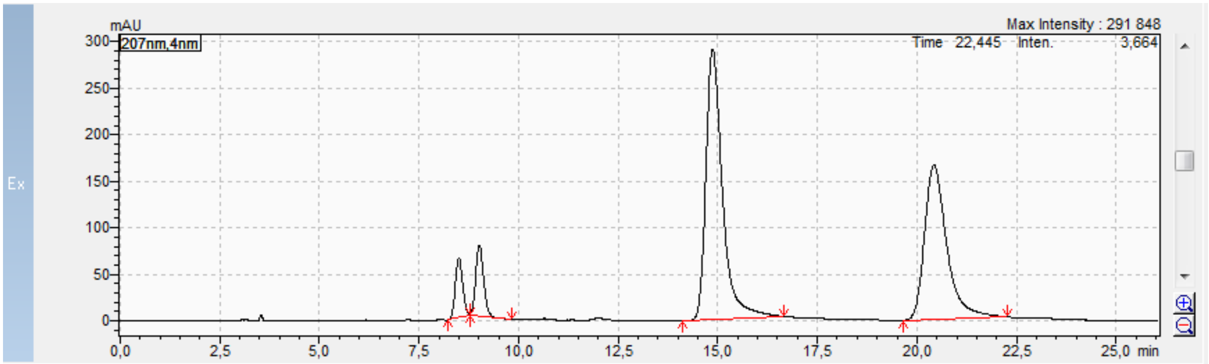

Results View - Peak Table

Peak Table | Compound | Group | Calibration Curve

Peak#	Ret. Time	Conc.	Area	Height	Similarity Index	Mark	Peak Start	Peak End	Area%
1	8,501	4,856	810029	64130	0,000000	M	8,224	8,779	4,856
2	9,016	6,146	1025242	75884	0,000000	M	8,779	9,824	6,146
3	14,873	50,280	8387620	290341	0,000000	M	14,112	16,661	50,280
4	20,428	38,718	6458870	165704	0,000000	M	19,659	22,272	38,718
Total		100,000	16681760	596059					100,000

for **4c**: *ee* = 12%
for **5c**: *ee* = 12%

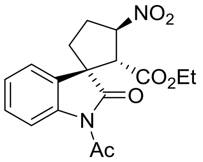

Conditions: IB column
mobile phase: *n*-heptane/*i*-PrOH = 98/2
λ = 224 nm, V = 1.0 ml/min, t = 25 °C
for **3d**: t_R = 17.2 min (minor), t_R = 38.1 min (major)

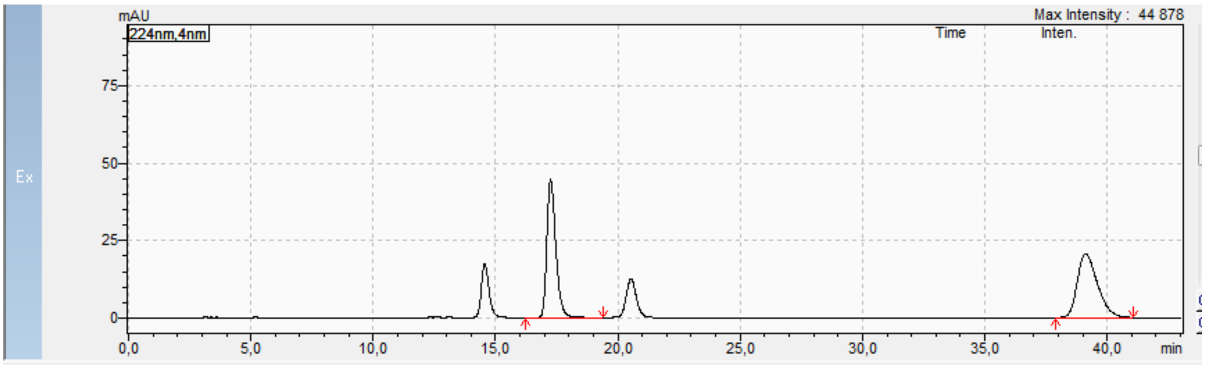

Results View - Peak Table

Peak Table | Compound | Group | Calibration Curve

Peak#	Ret. Time	Conc.	Area	Height	Similarity Index	Mark	Peak Start	Peak End	Area%
1	17,248	49,194	1142605	44743	0,000000	M	16,235	19,392	49,194
2	39,119	50,806	1180067	20539	0,000000	M	37,888	41,067	50,806
Total		100,000	2322671	65283					100,000

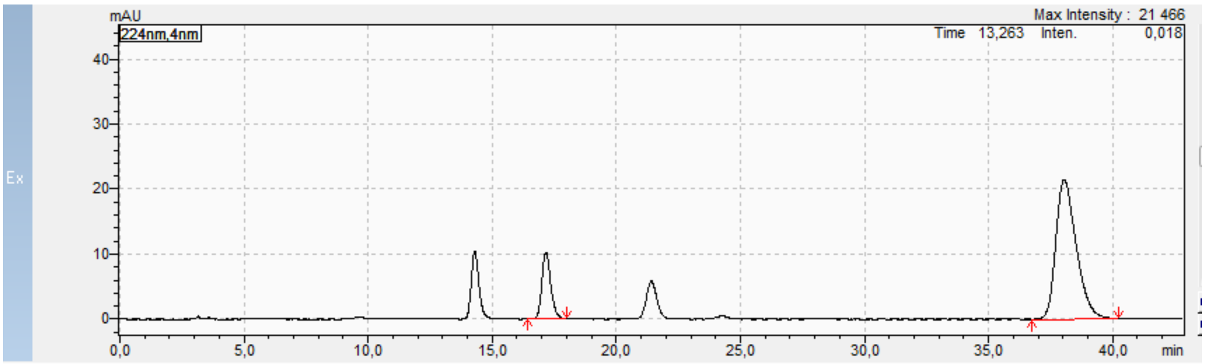

Results View - Peak Table

Peak Table | Compound | Group | Calibration Curve

Peak#	Ret. Time	Conc.	Area	Height	Similarity Index	Mark	Peak Start	Peak End	Area%
1	17,162	17,288	256494	10273	0,000000	M	16,448	18,016	17,288
2	38,047	82,712	1227145	21462	0,000000	M	36,736	40,224	82,712
Total		100,000	1483640	31735					100,000

for **3d**: *ee* = 65%

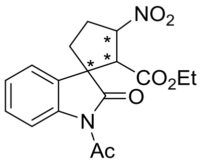

Conditions: IB column
mobile phase: *n*-heptane/*i*-PrOH = 98/2
λ = 216 nm, V = 1.0 ml/min, t = 25 °C
for **4d**: t_R = 23.8 min (major), t_R = 49.2 min (minor)

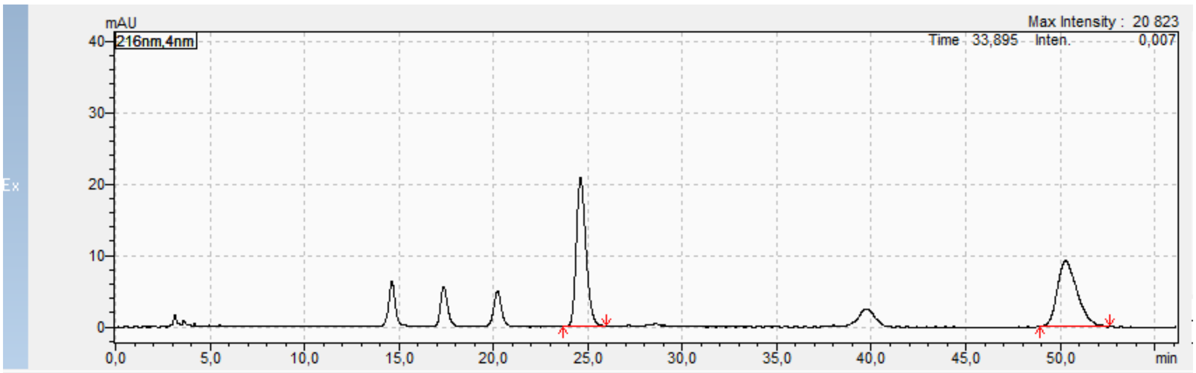

Peak#	Ret. Time	Conc.	Area	Height	Similarity Index	Mark	Peak Start	Peak End	Area%
1	24,608	51,914	734392	20724	0,000000	M	23,680	25,941	51,914
2	50,275	48,086	680231	9276	0,000000	M	48,928	52,629	48,086
Total		100,000	1414623	30000					100,000

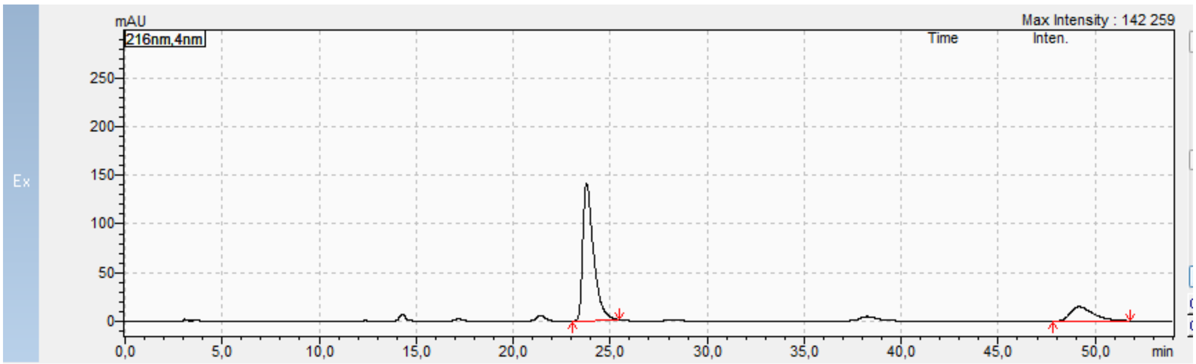

Peak#	Ret. Time	Conc.	Area	Height	Similarity Index	Mark	Peak Start	Peak End	Area%
1	23,779	82,213	5497012	142026	0,000000	M	23,051	25,472	82,213
2	49,158	17,787	1189329	14606	0,000000	M	47,819	51,797	17,787
Total		100,000	6686342	156631					100,000

for **4d**: *ee* = 64%

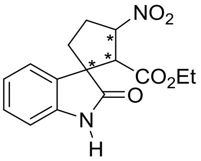

Conditions: IC column
mobile phase: *n*-heptane/*i*-PrOH = 90/10
λ = 208 nm, V = 1.0 ml/min, t = 25 °C
for **3f**: t_R = 9.6 min (minor), t_R = 12.6 min (major)
for **4f´**: t_R = 13.6 min, t_R = 28.1 min

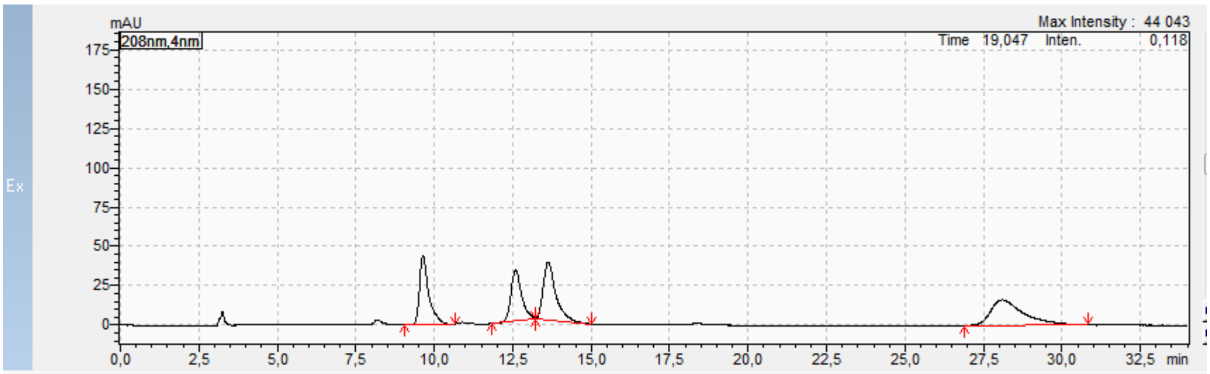

Results View - Peak Table

Peak Table | Compound | Group | Calibration Curve

Peak#	Ret. Time	Conc.	Area	Height	Similarity Index	Mark	Peak Start	Peak End	Area%
1	9,643	23,330	896963	43801	0,000000	M	9,056	10,656	23,330
2	12,595	19,355	744130	31937	0,000000	M	11,840	13,216	19,355
3	13,625	26,592	1022378	36521	0,000000	M	13,216	14,997	26,592
4	28,115	30,723	1181173	16146	0,000000	M	26,880	30,816	30,723
Total		100,000	3844643	128404					100,000

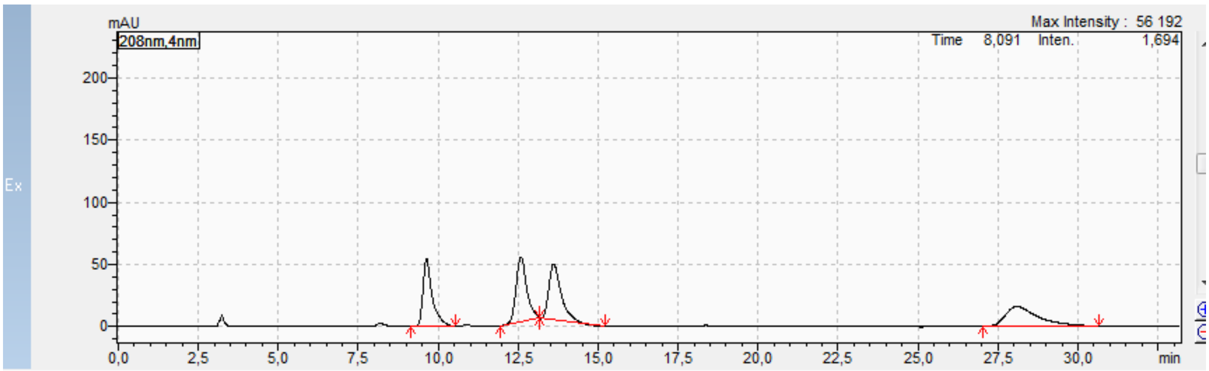

Results View - Peak Table

Peak Table | Compound | Group | Calibration Curve

Peak#	Ret. Time	Conc.	Area	Height	Similarity Index	Mark	Peak Start	Peak End	Area%
1	9,634	24,128	1118558	54601	0,000000	M	9,141	10,539	24,128
2	12,585	25,805	1196320	52011	0,000000	M	11,936	13,163	25,805
3	13,612	25,127	1164869	44810	0,000000	M	13,163	15,221	25,127
4	28,094	24,940	1156198	16024	0,000000	M	27,029	30,656	24,940
Total		100,000	4635946	167446					100,000

for **3f**: *ee* = 2%

for **4f´**: *ee* = 0%

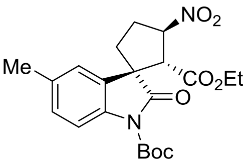

Conditions: IA column
mobile phase: *n*-heptane/*i*-PrOH = 90/10
λ = 204 nm, V = 1.0 ml/min, t = 25 °C
for **3g**: t_R = 4.9 min (minor), t_R = 6.0 min (major)

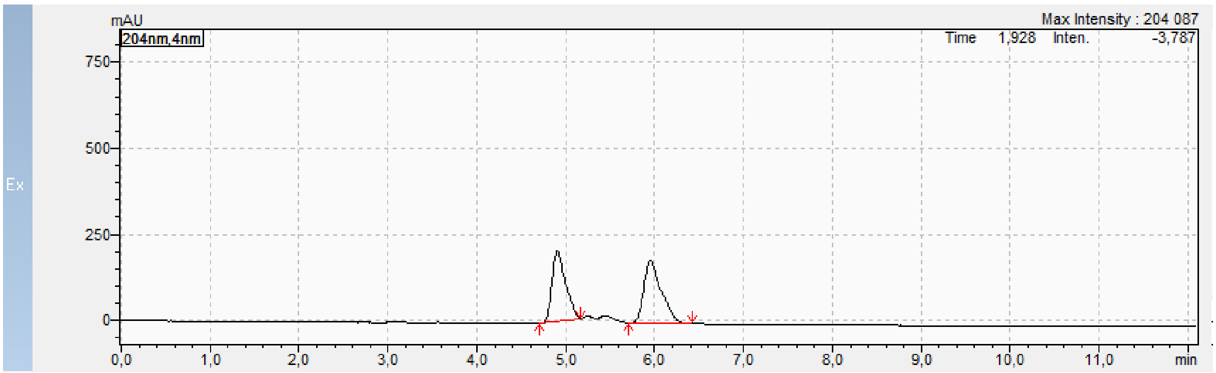

Results View - Peak Table

Peak Table | Compound | Group | Calibration Curve

Peak#	Ret. Time	Conc.	Area	Height	Similarity Index	Mark	Peak Start	Peak End	Area%
1	4,904	46,943	2177163	205444	0,000000	M	4,704	5,163	46,943
2	5,956	53,057	2460756	183312	0,000000	M	5,707	6,421	53,057
Total		100,000	4637919	388755					100,000

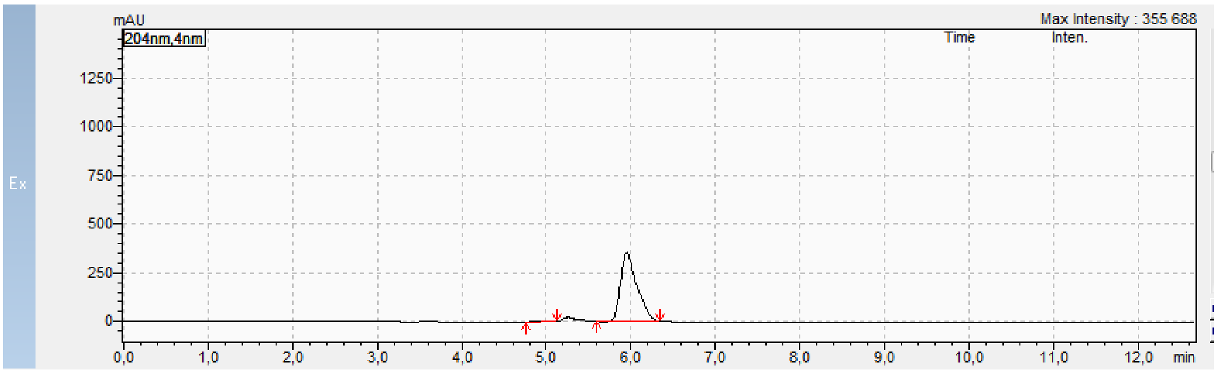

Results View - Peak Table

Peak Table | Compound | Group | Calibration Curve

Peak#	Ret. Time	Conc.	Area	Height	Similarity Index	Mark	Peak Start	Peak End	Area%
1	4,897	0,247	11843	2354	0,000000	M	4,757	5,131	0,247
2	5,953	99,753	4774010	356350	0,000000	M	5,589	6,347	99,753
Total		100,000	4785853	358704					100,000

for **3g**: *ee* = 99%

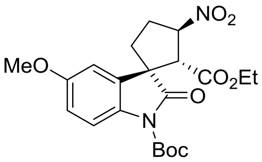

Conditions: IB column
mobile phase: *n*-heptane/*i*-PrOH = 80/20
λ = 207 nm, V = 1.0 ml/min, t = 25 °C
for **3h**: t_R = 6.5 min (minor), t_R = 8.6 min (major)

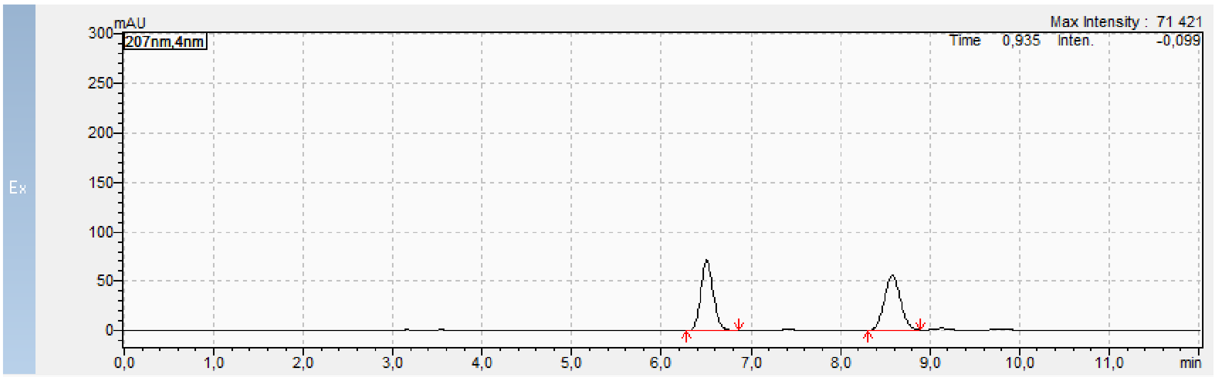

Results View - Peak Table

Peak Table | Compound | Group | Calibration Curve

Peak#	Ret. Time	Conc.	Area	Height	Similarity Index	Mark	Peak Start	Peak End	Area%
1	6,508	49,200	684815	71082	0,000000	M	6,283	6,859	49,200
2	8,579	50,800	707083	55353	0,000000	M	8,309	8,896	50,800
Total		100,000	1391899	126435					100,000

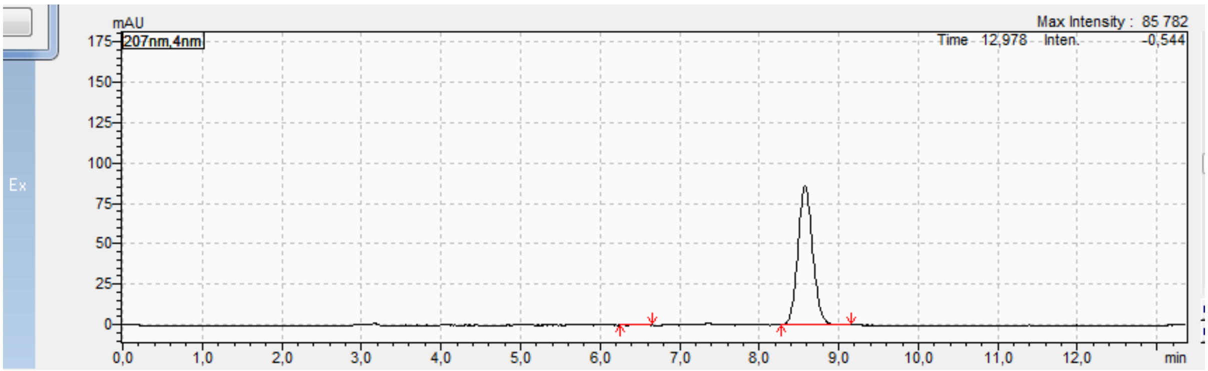

Results View - Peak Table

Peak Table | Compound | Group | Calibration Curve

Peak#	Ret. Time	Conc.	Area	Height	Similarity Index	Mark	Peak Start	Peak End	Area%
1	6,513	0,267	2973	364	0,000000	M	6,251	6,656	0,267
2	8,577	99,733	1108415	85945	0,000000	M	8,277	9,152	99,733
Total		100,000	1111388	86310					100,000

for **3h**: *ee* = 99%

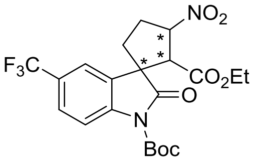

Conditions: IC column
mobile phase: *n*-heptane / *i*-PrOH = 98/2
λ = 222 nm, V = 1.0 ml/min, t = 25 °C
for **3i**: t_R = 17.6 min (minor), t_R = 36.6 min (major)
for **5i**: t_R = 22.1 min (major), t_R = 25.2 min (minor)

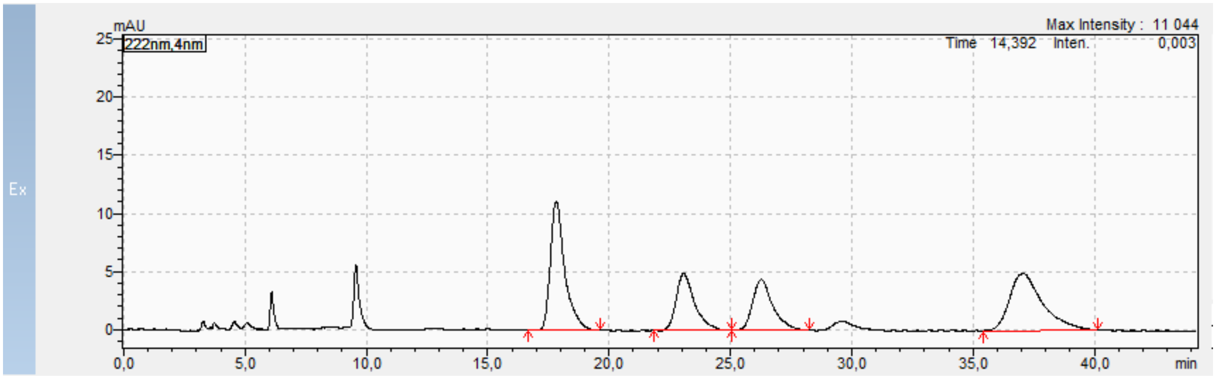

Results View - Peak Table

Peak Table | Compound | Group | Calibration Curve

Peak#	Ret. Time	Conc.	Area	Height	Similarity Index	Mark	Peak Start	Peak End	Area%
1	17,829	32,131	462415	11115	0,000000	M	16,683	19,627	32,131
2	23,069	17,846	256839	4885	0,000000	M	21,845	25,077	17,846
3	26,274	17,574	252926	4381	0,000000	M	25,077	28,267	17,574
4	37,068	32,448	466988	4934	0,000000	M	35,403	40,149	32,448
Total		100,000	1439168	25316					100,000

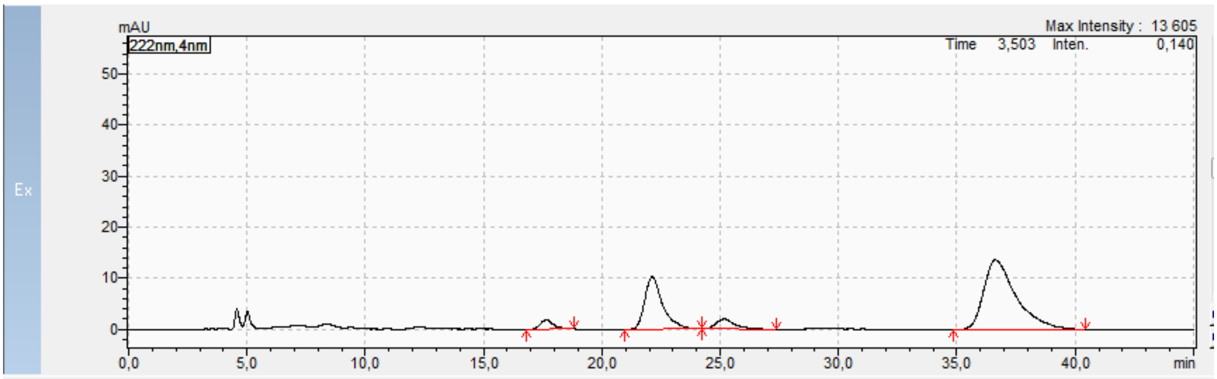

Results View - Peak Table

Peak Table | Compound | Group | Calibration Curve

Peak#	Ret. Time	Conc.	Area	Height	Similarity Index	Mark	Peak Start	Peak End	Area%
1	17,621	3,426	69463	1785	0,000000	M	16,800	18,827	3,426
2	22,132	26,804	543447	10262	0,000000	M	20,960	24,224	26,804
3	25,161	5,609	113728	1978	0,000000	M	24,224	27,371	5,609
4	36,612	64,160	1300811	13628	0,000000	M	34,859	40,437	64,160
Total		100,000	2027449	27653					100,000

for **3i**: *ee* = 90%

for **5i**: *ee* = 66%

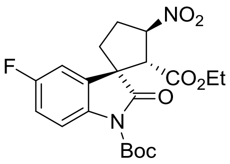

Conditions: IB column
mobile phase: *n*-heptane/*i*-PrOH = 80/20
λ = 208 nm, V = 1.0 ml/min, t = 25 °C
for **3k**: t_R = 5.8 min (minor), t_R = 6.4 min (major)

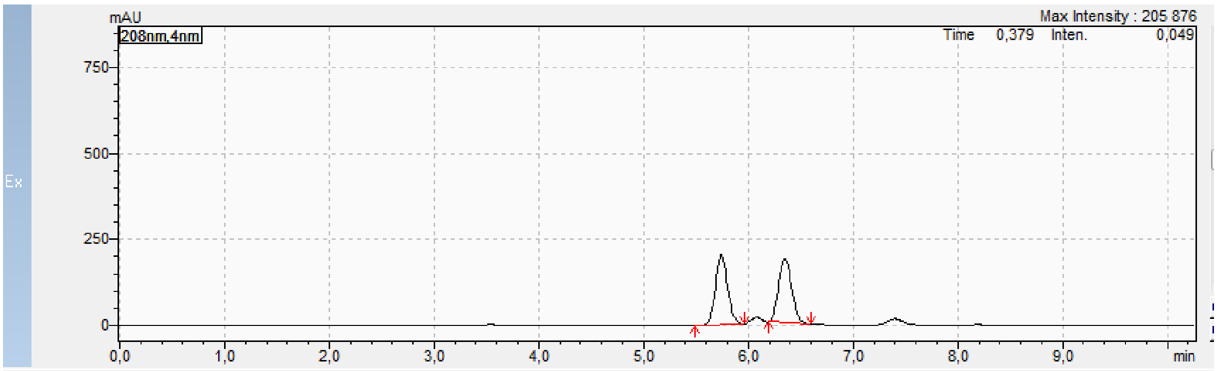

Results View - Peak Table

Peak Table | Compound | Group | Calibration Curve

Peak#	Ret. Time	Conc.	Area	Height	Similarity Index	Mark	Peak Start	Peak End	Area%
1	5,742	49,897	1613410	202507	0,000000	M	5,493	5,963	49,897
2	6,348	50,103	1620089	187636	0,000000	M	6,197	6,603	50,103
Total		100,000	3233499	390142					100,000

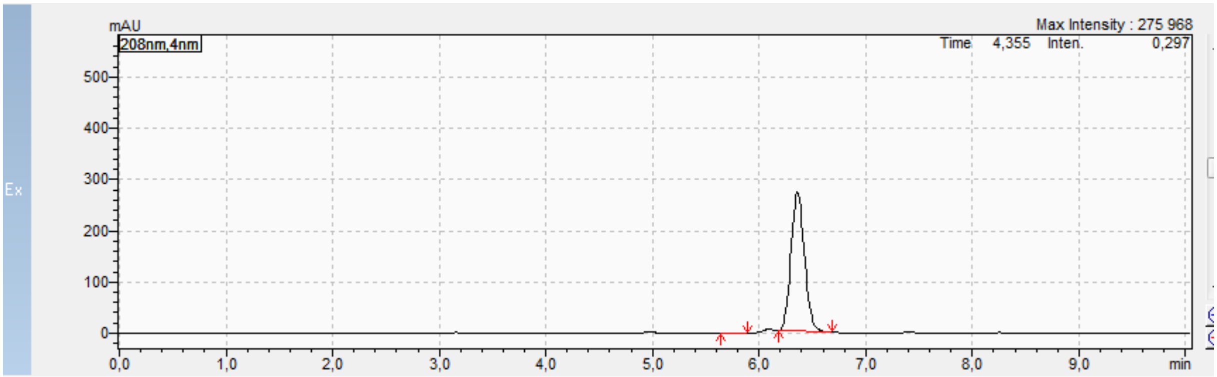

Results View - Peak Table

Peak Table | Compound | Group | Calibration Curve

Peak#	Ret. Time	Conc.	Area	Height	Similarity Index	Mark	Peak Start	Peak End	Area%
1	5,757	0,199	4813	728	0,000000	M	5,643	5,888	0,199
2	6,360	99,801	2409881	271752	0,000000	M	6,187	6,688	99,801
Total		100,000	2414695	272480					100,000

for **3k**: *ee* = 99%

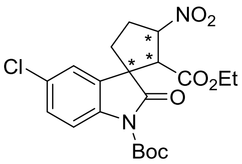

Conditions: IG column
mobile phase: *n*-heptane/*i*-PrOH = 90/10
λ = 209 nm, V = 1.0 ml/min, t = 25 °C
for **3l**: t_R = 8.1 min (minor), t_R = 11.7 min (major)
for **4l**: t_R = 10.0 min (minor), t_R = 10.8 min (major)

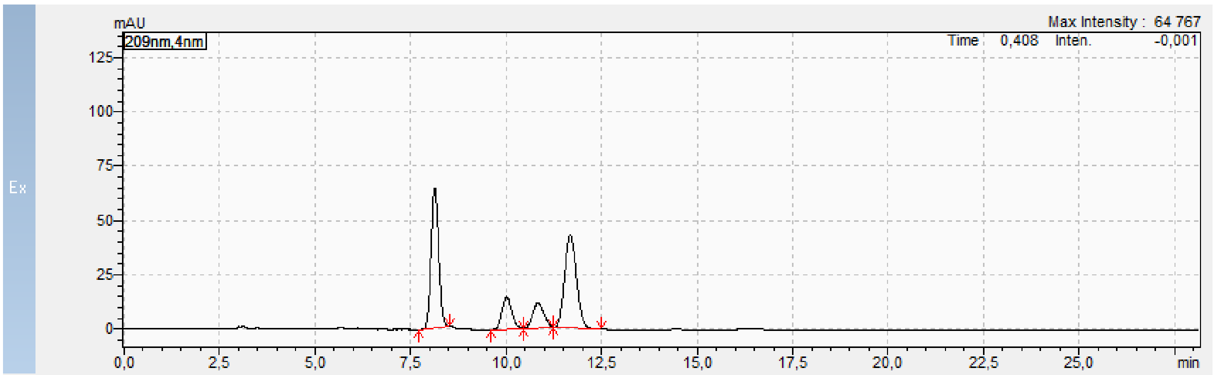

Results View - Peak Table

Peak Table | Compound | Group | Calibration Curve

Peak#	Ret. Time	Conc.	Area	Height	Similarity Index	Mark	Peak Start	Peak End	Area%
1	8,132	38,673	892990	64173	0,000000	M	7,712	8,523	38,673
2	10,016	11,446	264293	14987	0,000000	M	9,600	10,453	11,446
3	10,840	9,970	230206	11370	0,000000	M	10,453	11,232	9,970
4	11,682	39,912	921609	42655	0,000000	M	11,232	12,491	39,912
Total		100,000	2309098	133185					100,000

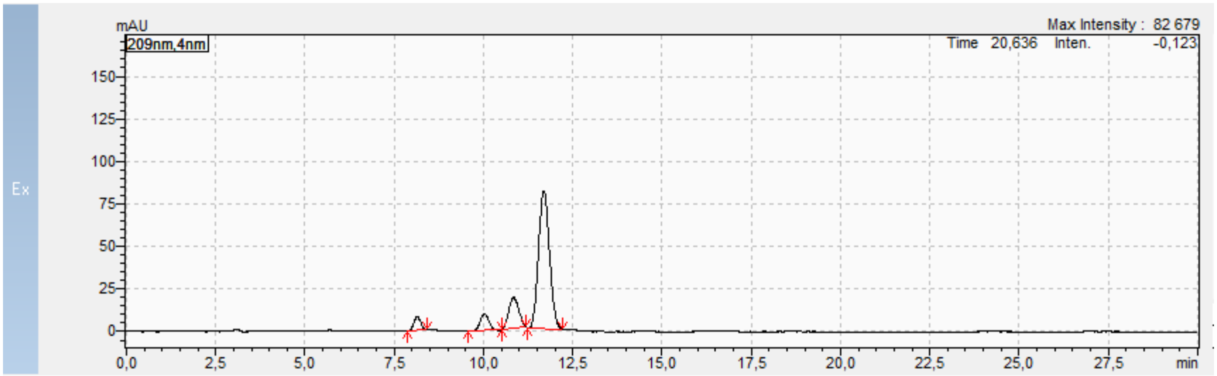

Results View - Peak Table

Peak Table | Compound | Group | Calibration Curve

Peak#	Ret. Time	Conc.	Area	Height	Similarity Index	Mark	Peak Start	Peak End	Area%
1	8,145	4,734	112210	8353	0,000000	M	7,872	8,416	4,734
2	10,026	6,910	163780	9793	0,000000	M	9,568	10,517	6,910
3	10,844	15,068	357156	18104	0,000000	M	10,517	11,189	15,068
4	11,691	73,287	1737072	81029	0,000000	M	11,232	12,213	73,287
Total		100,000	2370218	117279					100,000

for **3l**: *ee* = 88%

for **4l**: *ee* = 37%

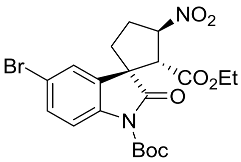

Conditions: IA column
mobile phase: *n*-heptane/*i*-PrOH = 90/10
λ = 205 nm, V = 1.0 ml/min, t = 25 °C
for **3m**: t_R = 5.1 min (minor), t_R = 6.3 min (major)

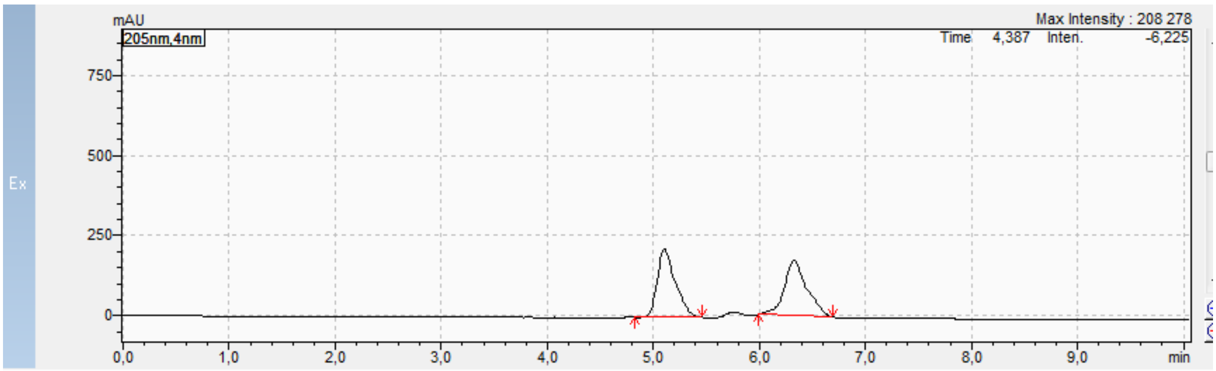

Results View - Peak Table

Peak#	Ret. Time	Conc.	Area	Height	Similarity Index	Mark	Peak Start	Peak End	Area%
1	5,104	49,963	2526742	213840	0,000000	M	4,821	5,461	49,963
2	6,327	50,037	2530494	170975	0,000000	M	5,995	6,699	50,037
Total		100,000	5057236	384815					100,000

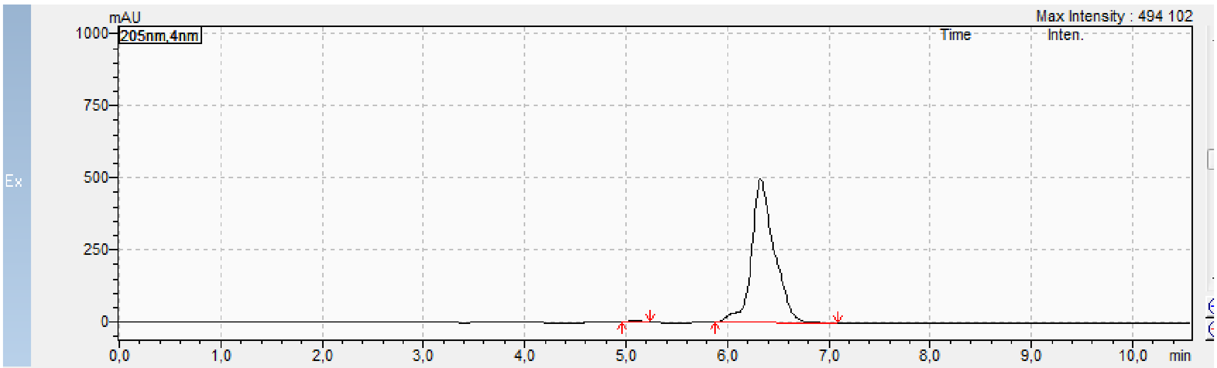

Results View - Peak Table

Peak#	Ret. Time	Conc.	Area	Height	Similarity Index	Mark	Peak Start	Peak End	Area%
1	5,082	0,569	44291	4943	0,000000	M	4,960	5,237	0,569
2	6,330	99,431	7733995	495306	0,000000	M	5,877	7,093	99,431
Total		100,000	7778286	500250					100,000

for **3m**: *ee* = 99%

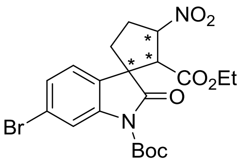

Conditions: IA column
mobile phase: *n*-heptane/*i*-PrOH = 80/20
λ = 221 nm, V = 1.0 ml/min, t = 25 °C
for **3n**: t_R = 4.4 min (minor), t_R = 5.6 min (major)
for **4n**: t_R = 5.2 min (major), t_R = 6.2 min (minor)

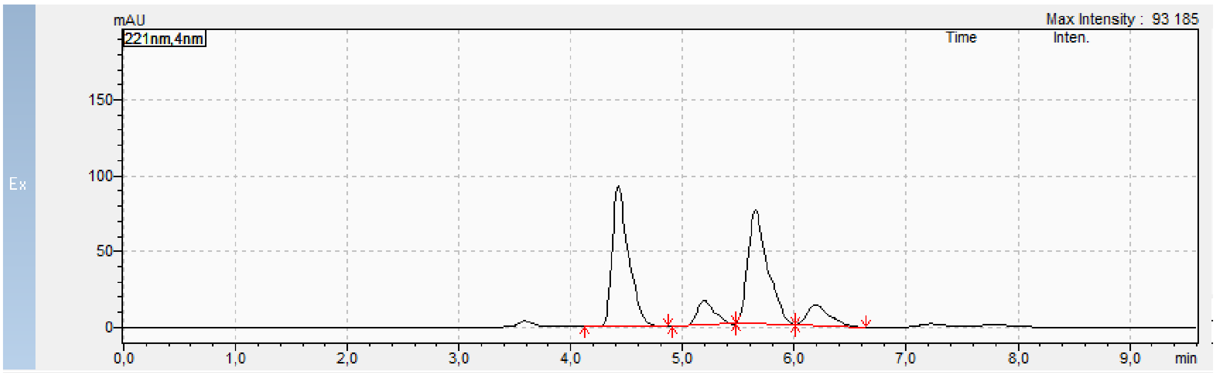

Results View - Peak Table

Peak Table | Compound | Group | Calibration Curve

Peak#	Ret. Time	Conc.	Area	Height	Similarity Index	Mark	Peak Start	Peak End	Area%
1	4,425	42,329	939528	92522	0,000000	M	4,128	4,864	42,329
2	5,195	7,830	173794	16594	0,000000	M	4,907	5,472	7,830
3	5,653	41,777	927266	74644	0,000000	M	5,472	6,005	41,777
4	6,190	8,064	178985	13649	0,000000	M	6,005	6,645	8,064
Total		100,000	2219573	197409					100,000

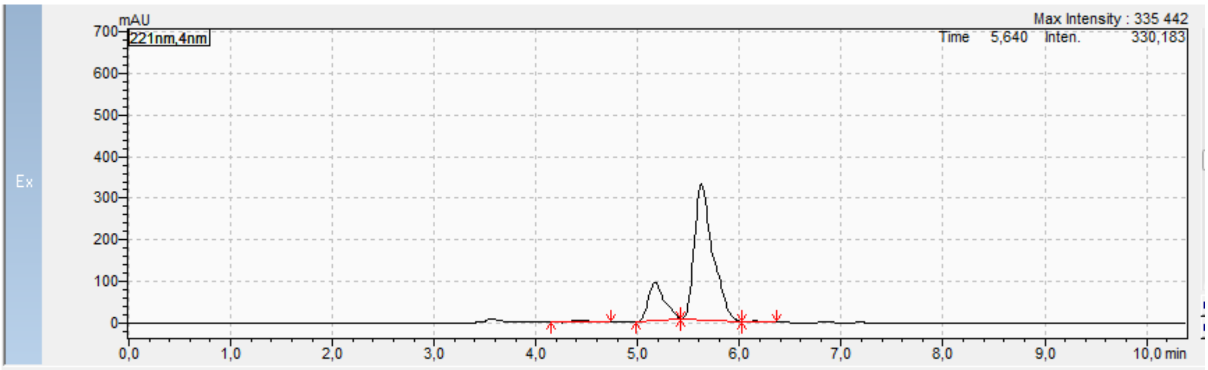

Results View - Peak Table

Peak Table | Compound | Group | Calibration Curve

Peak#	Ret. Time	Conc.	Area	Height	Similarity Index	Mark	Peak Start	Peak End	Area%
1	4,403	1,168	60668	5971	0,000000	M	4,149	4,736	1,168
2	5,169	19,336	1003939	92460	0,000000	M	4,981	5,419	19,336
3	5,627	79,227	4113618	327870	0,000000	M	5,419	6,027	79,227
4	6,157	0,269	13952	1732	0,000000	M	6,027	6,368	0,269
Total		100,000	5192177	428033					100,000

for **3n**: *ee* = 97%

for **4n**: *ee* = 97%

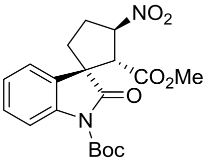

Conditions: IA column
mobile phase: *n*-heptane/*i*-PrOH = 80/20
λ = 210 nm, V = 1.0 ml/min, t = 25 °C
for **3o**: t_R = 4.6 min (minor), t_R = 5.2 min (major)

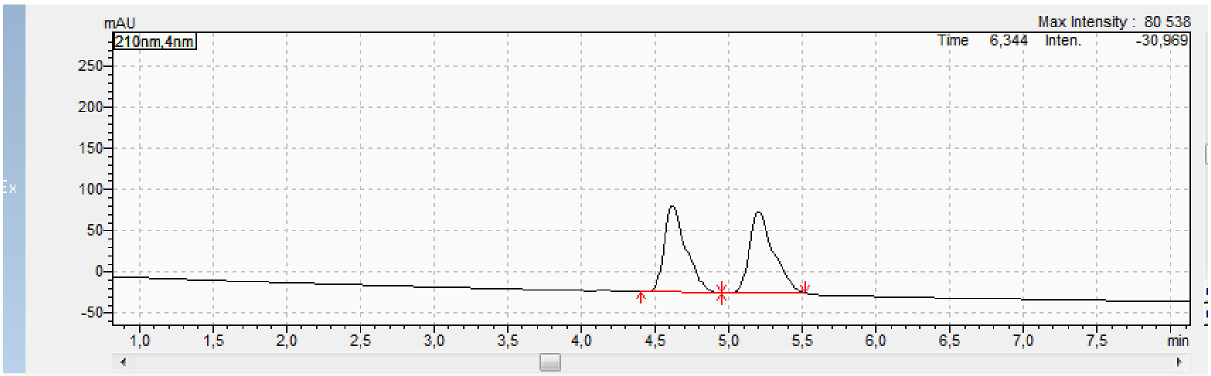

Results View - Peak Table

Peak Table | Compound | Group | Calibration Curve

Peak#	Ret. Time	Conc.	Area	Height	Similarity Index	Mark	Peak Start	Peak End	Area%
1	4,618	48,858	1081987	104995	0,000000	M	4,405	4,949	48,858
2	5,203	51,142	1132553	98974	0,000000	M	4,949	5,515	51,142
Total		100,000	2214540	203968					100,000

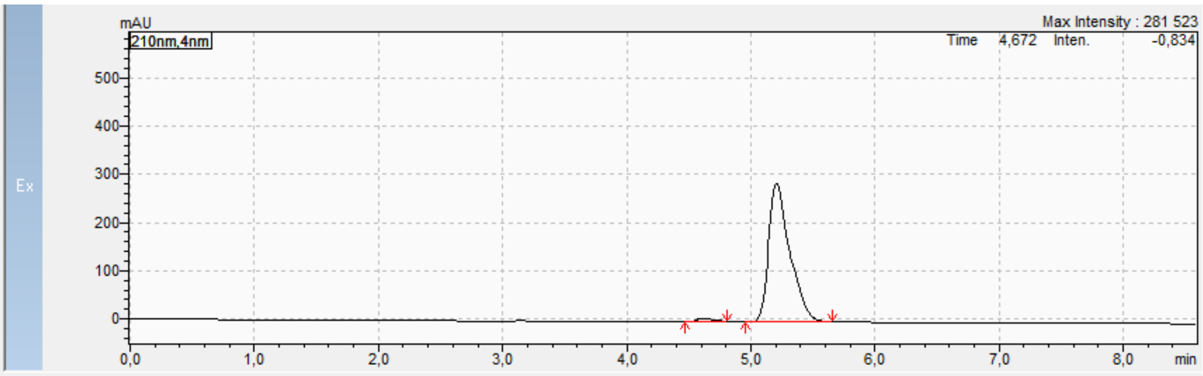

Results View - Peak Table

Peak Table | Compound | Group | Calibration Curve

Peak#	Ret. Time	Conc.	Area	Height	Similarity Index	Mark	Peak Start	Peak End	Area%
1	4,621	1,806	62767	6775	0,000000	M	4,469	4,811	1,806
2	5,208	98,194	3412929	287893	0,000000	M	4,960	5,653	98,194
Total		100,000	3475695	294667					100,000

for **3o**: *ee* = 96%

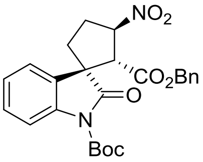

Conditions: IA column
mobile phase: *n*-heptane/*i*-PrOH = 80/20
λ = 217 nm, V = 1.0 ml/min, t = 25 °C
for **3p**: t_R = 4.7 min (minor), t_R = 6.5 min (major)

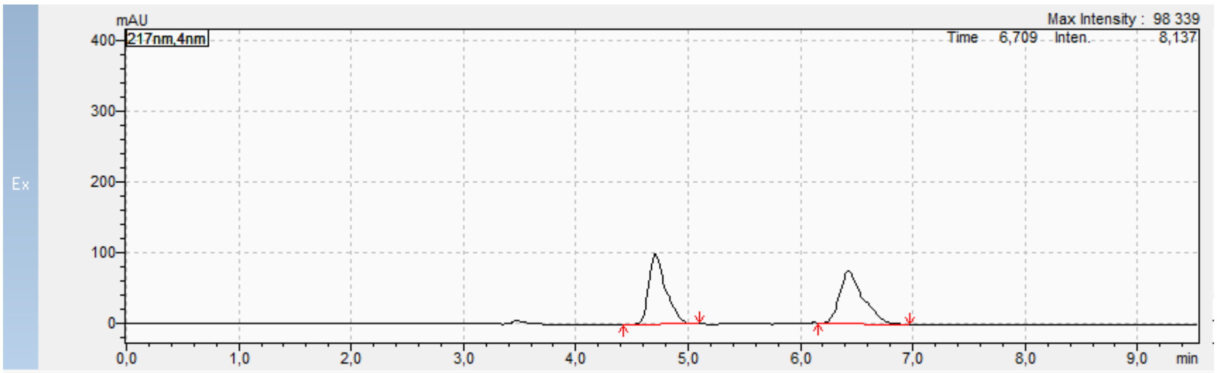

Results View - Peak Table

Peak Table | Compound | Group | Calibration Curve

Peak#	Ret. Time	Conc.	Area	Height	Similarity Index	Mark	Peak Start	Peak End	Area%
1	4,708	49,777	1041668	99000	0,000000	M	4,427	5,099	49,777
2	6,431	50,223	1050984	73292	0,000000	M	6,155	6,976	50,223
Total		100,000	2092653	172292					100,000

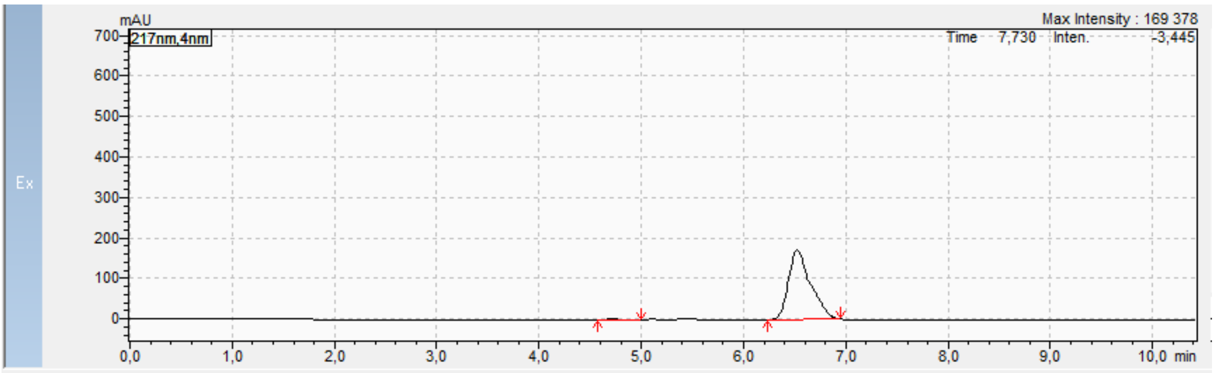

Results View - Peak Table

Peak Table | Compound | Group | Calibration Curve

Peak#	Ret. Time	Conc.	Area	Height	Similarity Index	Mark	Peak Start	Peak End	Area%
1	4,720	0,383	9873	989	0,000000	M	4,576	5,003	0,383
2	6,524	99,617	2567890	170937	0,000000	M	6,229	6,944	99,617
Total		100,000	2577763	171925					100,000

for **3p**: *ee* = 99%

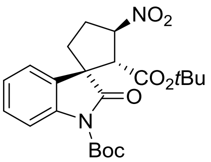

Conditions: IA column
mobile phase: *n*-heptane/*i*-PrOH = 80/20
λ = 211 nm, V = 1.0 ml/min, t = 25 °C
for **3q**: t_R = 4.0 min (minor), t_R = 4.4 min (major)

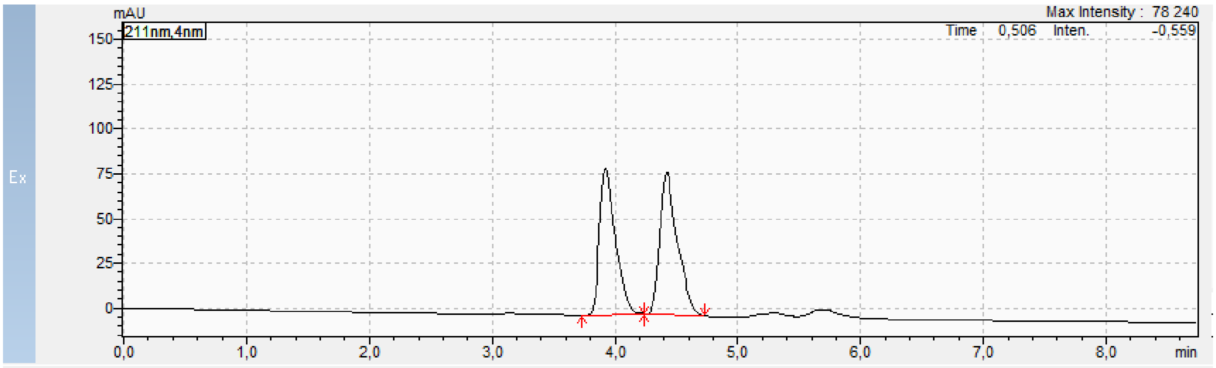

Results View - Peak Table

Peak Table | Compound | Group | Calibration Curve

Peak#	Ret. Time	Conc.	Area	Height	Similarity Index	Mark	Peak Start	Peak End	Area%
1	3,922	48,093	730559	81874	0,000000	M	3,733	4,235	48,093
2	4,419	51,907	788488	79488	0,000000	M	4,235	4,736	51,907
Total		100,000	1519047	161363					100,000

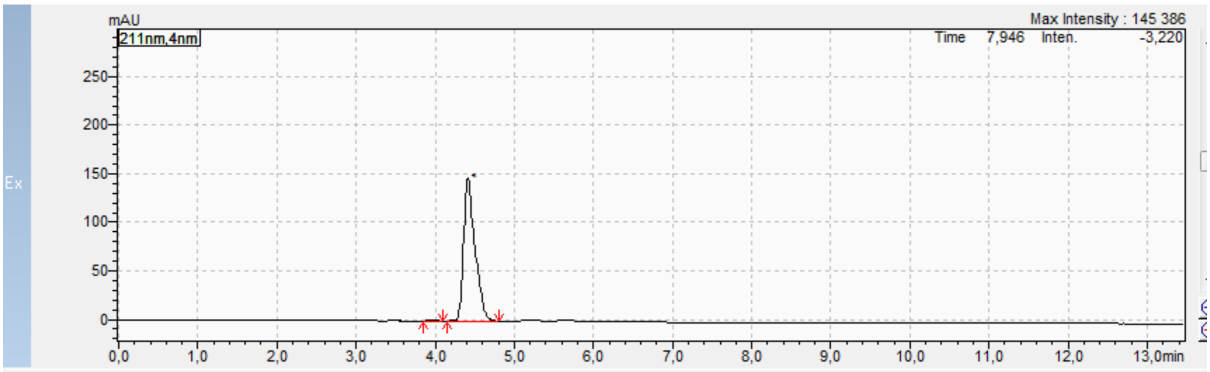

Results View - Peak Table

Peak Table | Compound | Group | Calibration Curve

Peak#	Ret. Time	Conc.	Area	Height	Similarity Index	Mark	Peak Start	Peak End	Area%
1	3,987	0,202	2973	328	0,000000	M	3,851	4,096	0,202
2	4,414	99,798	1470939	146999	0,000000	M	4,149	4,811	99,798
Total		100,000	1473911	147327					100,000

for **3q**: *ee* = 99%

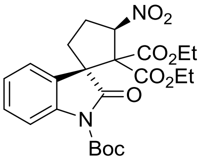

Conditions: IB column
mobile phase: n-heptane/i-PrOH = 80/20
λ = 208 nm, V = 1.0 ml/min, t = 25 °C
for **3r**: t_R = 4.8 min (minor), t_R = 5.4 min (major)

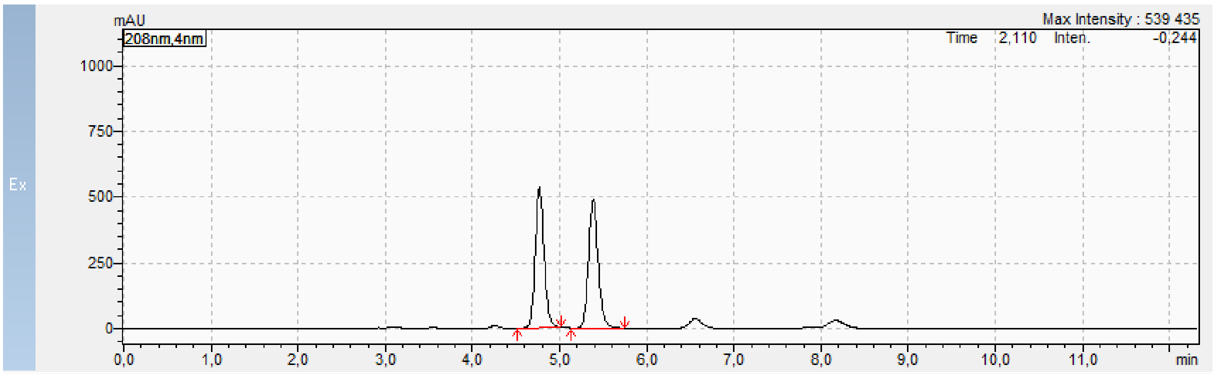

Results View - Peak Table

Peak Table | Compound | Group | Calibration Curve

Peak#	Ret. Time	Conc.	Area	Height	Similarity Index	Mark	Peak Start	Peak End	Area%
1	4,769	49,134	3715114	536338	0,000000	M	4,512	5,013	49,134
2	5,383	50,866	3846046	490502	0,000000	M	5,131	5,749	50,866
Total		100,000	7561160	1026839					100,000

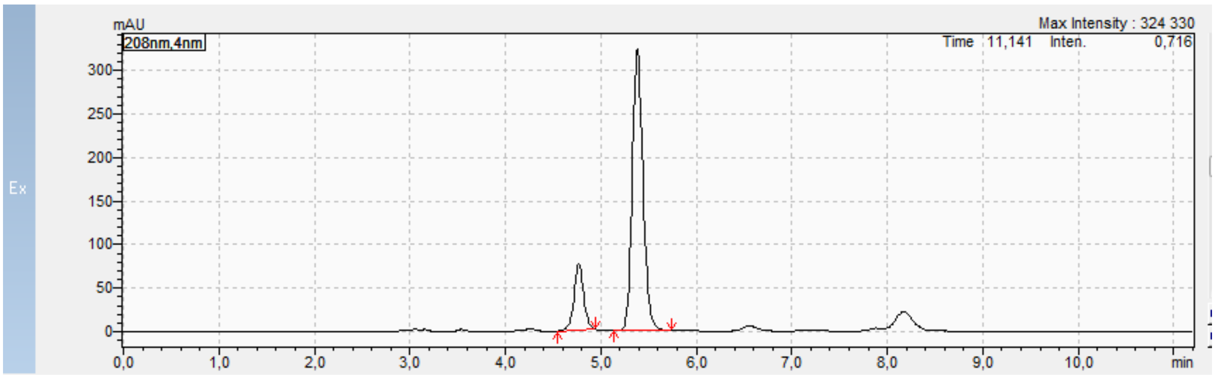

Results View - Peak Table

Peak Table | Compound | Group | Calibration Curve

Peak#	Ret. Time	Conc.	Area	Height	Similarity Index	Mark	Peak Start	Peak End	Area%
1	4,768	17,442	536120	76171	0,000000	M	4,544	4,939	17,442
2	5,382	82,558	2537584	323230	0,000000	M	5,131	5,739	82,558
Total		100,000	3073704	399402					100,000

for **3r**: *ee* = 65%

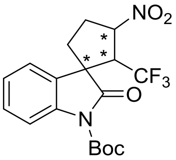

Conditions: IB column
mobile phase: *n*-heptane/*i*-PrOH = 80/20
λ = 208 nm, V = 1.0 ml/min, t = 25 °C
for **3s**: t_R = 5.9 min (major), t_R = 7.6 min (minor)

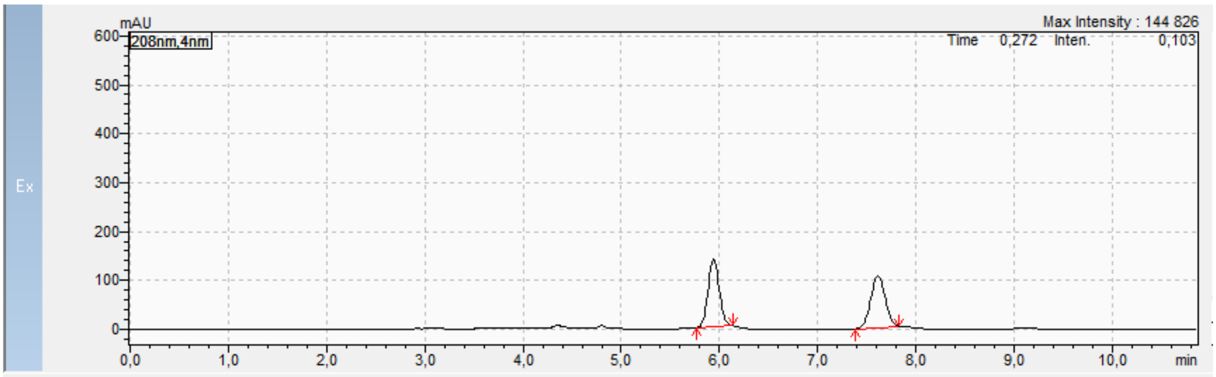

Results View - Peak Table

Peak Table | Compound | Group | Calibration Curve

Peak#	Ret. Time	Conc.	Area	Height	Similarity Index	Mark	Peak Start	Peak End	Area%
1	5,938	50,512	1088958	139027	0,000000	M	5,760	6,144	50,512
2	7,613	49,488	1066901	104636	0,000000	M	7,381	7,819	49,488
Total		100,000	2155859	243664					100,000

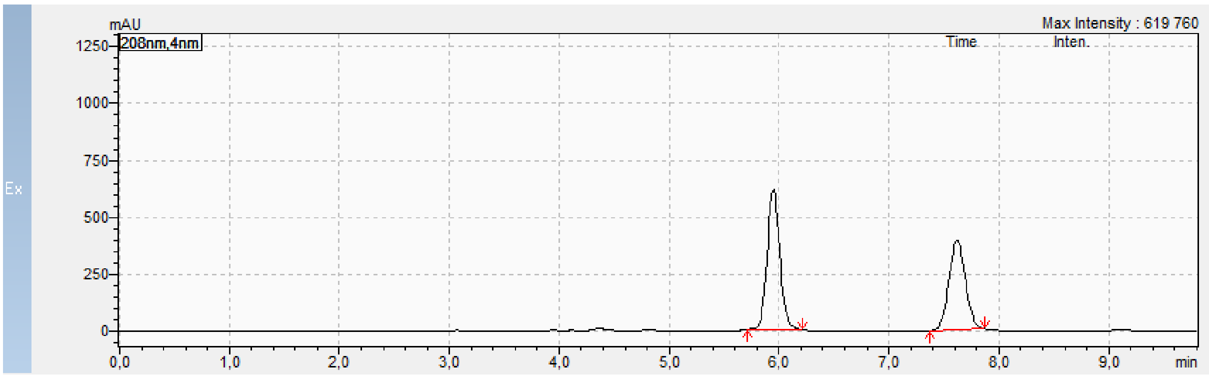

Results View - Peak Table

Peak Table | Compound | Group | Calibration Curve

Peak#	Ret. Time	Conc.	Area	Height	Similarity Index	Mark	Peak Start	Peak End	Area%
1	5,947	54,679	4986173	612509	0,000000	M	5,717	6,219	54,679
2	7,620	45,321	4132846	394371	0,000000	M	7,371	7,872	45,321
Total		100,000	9119020	1006881					100,000

for **3s**: *ee* = 9%

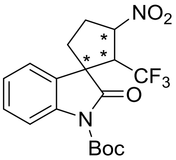

Conditions: IB column
mobile phase: *n*-heptane/*i*-PrOH = 80/20
λ = 261 nm, V = 1.0 ml/min, t = 25 °C
for **4s**: t_R = 4.8 min (minor), t_R = 6.1 min (major)

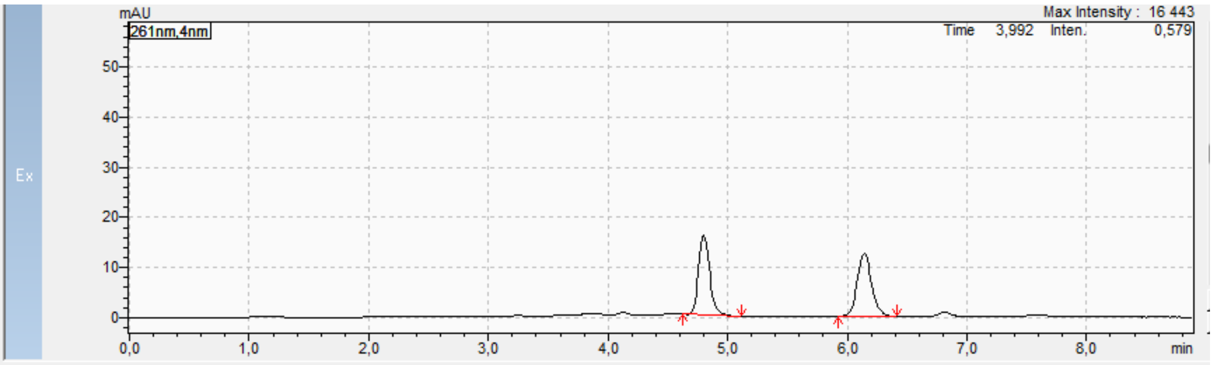

Results View - Peak Table

Peak Table | Compound | Group | Calibration Curve

Peak#	Ret. Time	Conc.	Area	Height	Similarity Index	Mark	Peak Start	Peak End	Area%
1	4,801	48,853	99418	15763	0,000000	M	4,619	5,120	48,853
2	6,142	51,147	104085	12501	0,000000	M	5,920	6,411	51,147
Total		100,000	203503	28264					100,000

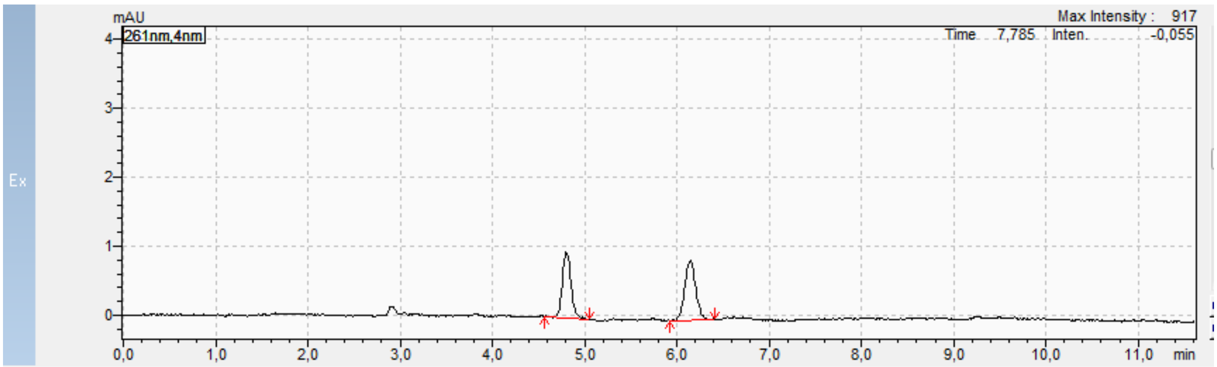

Results View - Peak Table

Peak Table | Compound | Group | Calibration Curve

Peak#	Ret. Time	Conc.	Area	Height	Similarity Index	Mark	Peak Start	Peak End	Area%
1	4,803	46,399	6176	959	0,000000	M	4,565	5,056	46,399
2	6,147	53,601	7134	865	0,000000	M	5,920	6,411	53,601
Total		100,000	13310	1825					100,000

for **4s**: *ee* = 6%

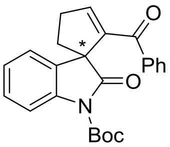

Conditions: IA column
mobile phase: *n*-heptane/*i*-PrOH = 80/20
λ = 216 nm, V = 1.0 ml/min, t = 25 °C
for **5t**: t_R = 4.9 min (minor), t_R = 6.6 min (major)

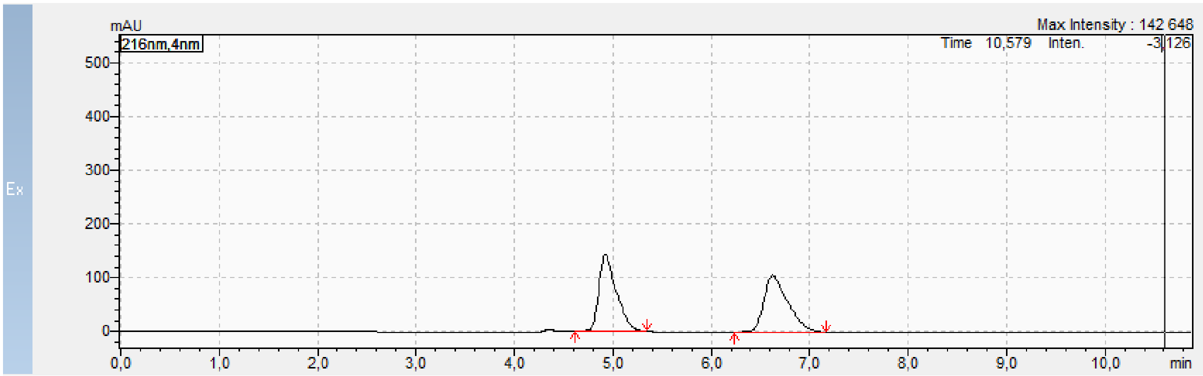

Results View - Peak Table

Peak Table | Compound | Group | Calibration Curve

Peak#	Ret. Time	Conc.	Area	Height	Similarity Index	Mark	Peak Start	Peak End	Area%
1	4,923	50,091	1787455	142842	0,000000	M	4,619	5,344	50,091
2	6,626	49,909	1780986	105804	0,000000	M	6,240	7,168	49,909
Total		100,000	3568441	248646					100,000

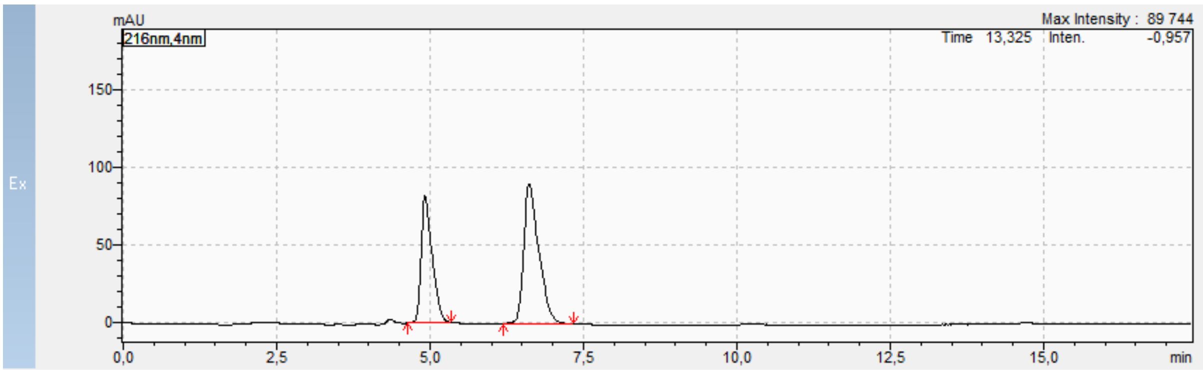

Results View - Peak Table

Peak Table | Compound | Group | Calibration Curve

Peak#	Ret. Time	Conc.	Area	Height	Similarity Index	Mark	Peak Start	Peak End	Area%
1	4,919	39,825	1024574	81891	0,000000	M	4,629	5,333	39,825
2	6,617	60,175	1548123	90301	0,000000	M	6,187	7,349	60,175
Total		100,000	2572697	172191					100,000

for **5t**: *ee* = 20%

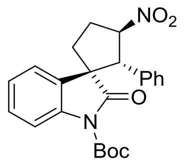

Conditions: IB column
mobile phase: *n*-heptane/*i*-PrOH = 80/20
λ = 199 nm, V = 1.0 ml/min, t = 25 °C
for **3v**: t_R = 5.2 min (major), t_R = 5.9 min (minor)

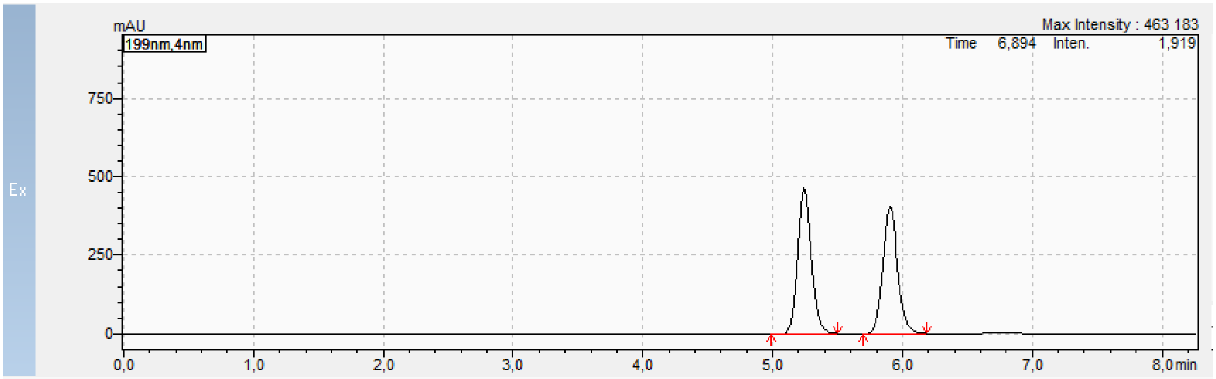

Results View - Peak Table

Peak Table | Compound | Group | Calibration Curve

Peak#	Ret. Time	Conc.	Area	Height	Similarity Index	Mark	Peak Start	Peak End	Area%
1	5,240	49,961	3310640	462846	0,000000	M	4,992	5,504	49,961
2	5,902	50,039	3315760	404381	0,000000	M	5,696	6,187	50,039
Total		100,000	6626400	867228					100,000

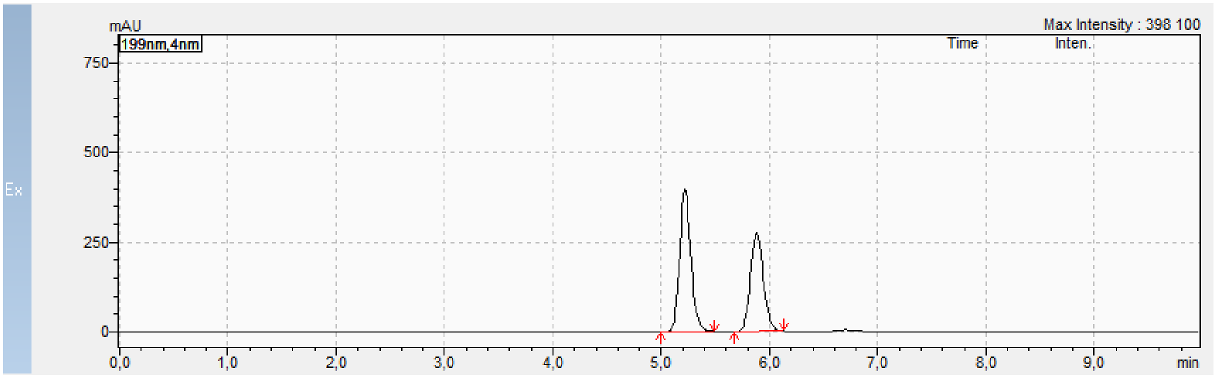

Results View - Peak Table

Peak Table | Compound | Group | Calibration Curve

Peak#	Ret. Time	Conc.	Area	Height	Similarity Index	Mark	Peak Start	Peak End	Area%
1	5,220	55,837	2890323	397657	0,000000	M	4,992	5,493	55,837
2	5,881	44,163	2286078	276829	0,000000	M	5,675	6,133	44,163
Total		100,000	5176401	674486					100,000

for **3v**: *ee* = 12%

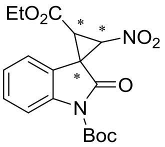

Conditions: IB column
mobile phase: *n*-heptane/*i*-PrOH = 80/20
λ = 227 nm, V = 1.0 ml/min, t = 25 °C
for **6**: t_R = 11.9 min (major), t_R = 16.8 min (minor)
for **6′**: t_R = 12.7 min (major), t_R = 24.0 min (minor)

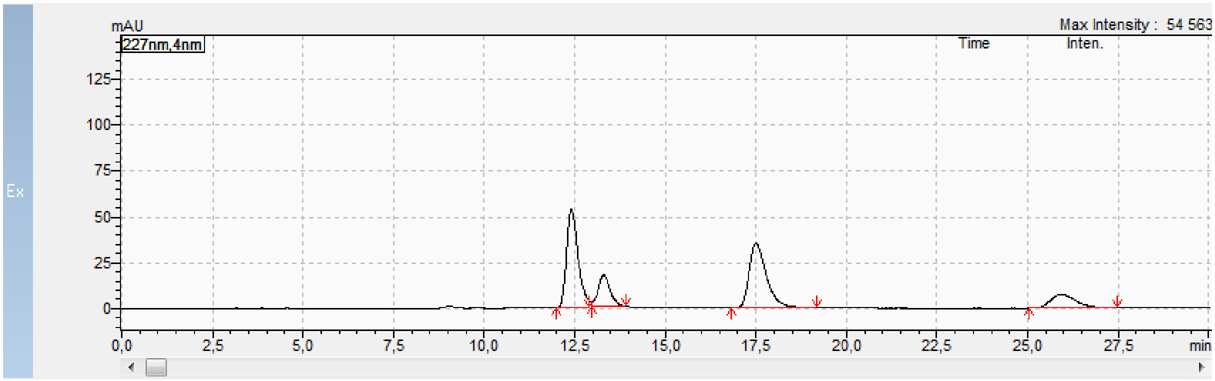

Results View - Peak Table

Peak Table | Compound | Group | Calibration Curve

Peak#	Ret. Time	Conc.	Area	Height	Similarity Index	Mark	Peak Start	Peak End	Area%
1	12,406	38,267	1183959	53754	0,000000	M	11,979	12,896	38,267
2	13,302	11,755	363682	16594	0,000000	M	12,981	13,909	11,755
3	17,504	38,142	1180117	35535	0,000000		16,843	19,179	38,142
4	25,938	11,836	366213	7503	0,000000		25,045	27,477	11,836
Total		100,000	3093971	113387					100,000

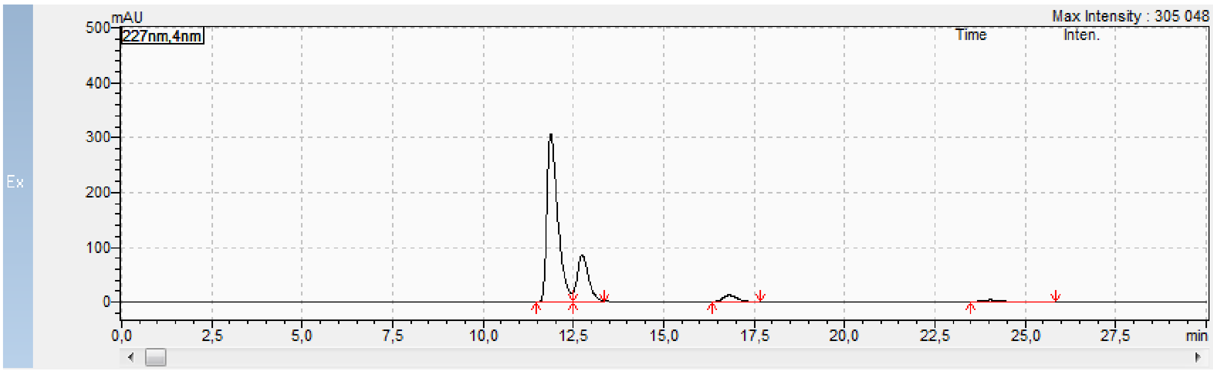

Results View - Peak Table

Peak Table | Compound | Group | Calibration Curve

Peak#	Ret. Time	Conc.	Area	Height	Similarity Index	Mark	Peak Start	Peak End	Area%
1	11,876	72,467	6469312	305316	0,000000	M	11,477	12,469	72,467
2	12,743	21,633	1931219	87123	0,000000	M	12,469	13,333	21,633
3	16,803	3,956	353133	11828	0,000000	M	16,352	17,685	3,956
4	24,034	1,945	173645	4105	0,000000	M	23,477	25,835	1,945
Total		100,000	8927309	408373					100,000

for **6**: *ee* = 90%

for **6′**: *ee* = 84%

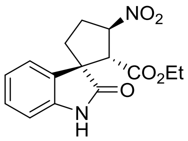

Conditions: IC column
mobile phase: *n*-heptane/*i*-PrOH = 90/10
λ = 209 nm, V = 1.0 ml/min, t = 25 °C
for **3f**: t_R = 9.5 min (major), t_R = 12.2 min (minor)

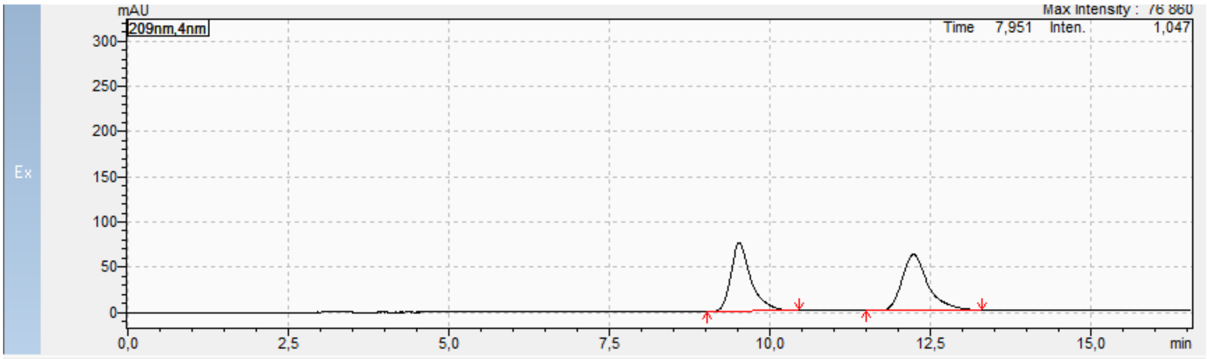

Results View - Peak Table

Peak Table | Compound | Group | Calibration Curve

Peak#	Ret. Time	Conc.	Area	Height	Similarity Index	Mark	Peak Start	Peak End	Area%
1	9,521	49,144	1642384	75267	0,000000	M	9,013	10,453	49,144
2	12,228	50,856	1699617	61724	0,000000	M	11,488	13,291	50,856
Total		100,000	3342001	136991					100,000

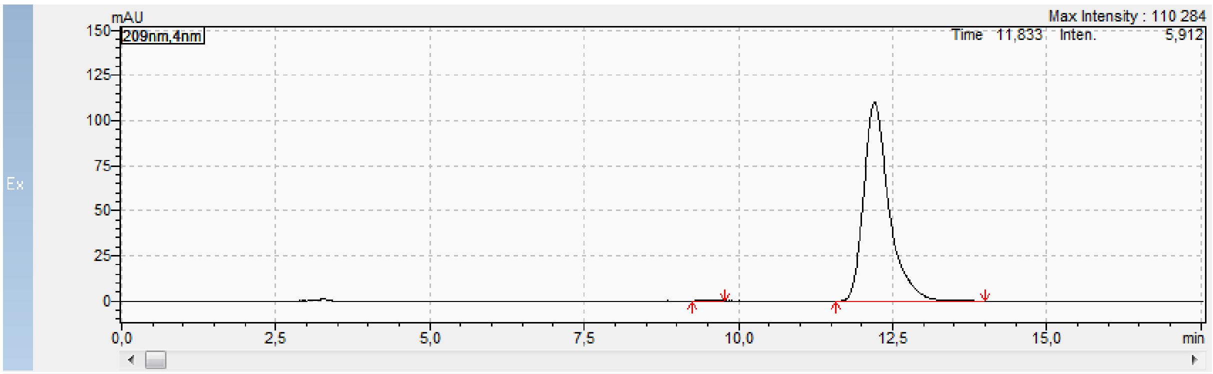

Results View - Peak Table

Peak Table | Compound | Group | Calibration Curve

Peak#	Ret. Time	Conc.	Area	Height	Similarity Index	Mark	Peak Start	Peak End	Area%
1	9,503	0,112	3528	233	0,000000		9,248	9,771	0,112
2	12,210	99,888	3143178	110290	0,000000	S	11,584	14,005	99,888
Total		100,000	3146706	110523					100,000

for **3f**: *ee* = 99%

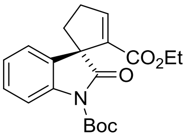

Conditions: IB column
mobile phase: *n*-heptane/*i*-PrOH = 98/2
λ = 243 nm, V = 1.0 ml/min, t = 25 °C
for **5a**: t_R = 10.5 min (major), t_R = 14.7 min (minor)

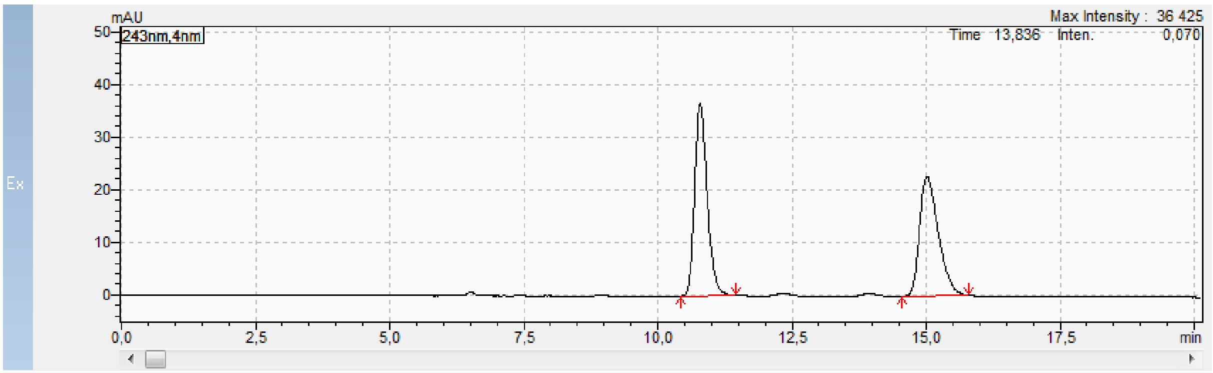

Results View - Peak Table

Peak Table | Compound | Group | Calibration Curve

Peak#	Ret. Time	Conc.	Area	Height	Similarity Index	Mark	Peak Start	Peak End	Area%
1	10,790	51,883	595664	36570	0,000000	M	10,421	11,467	51,883
2	15,017	48,117	552432	22720	0,000000	M	14,549	15,797	48,117
Total		100,000	1148096	59290					100,000

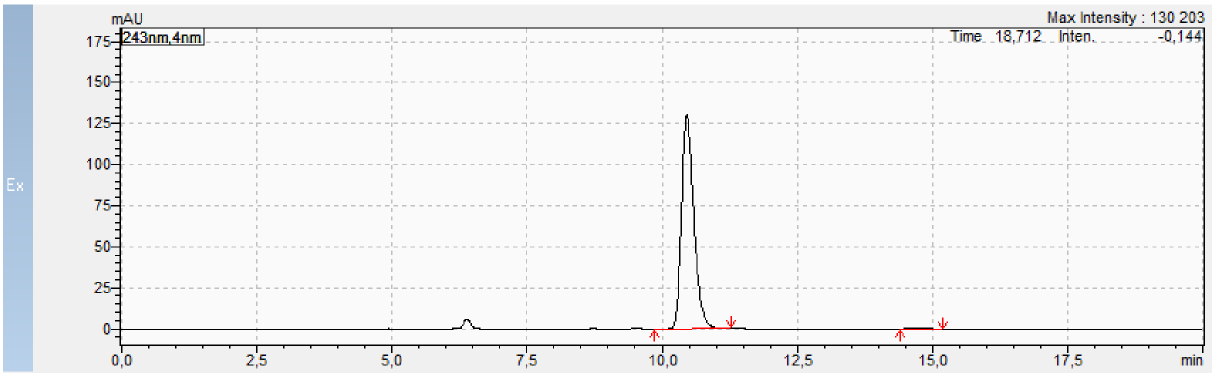

Results View - Peak Table

Peak Table | Compound | Group | Calibration Curve

Peak#	Ret. Time	Conc.	Area	Height	Similarity Index	Mark	Peak Start	Peak End	Area%
1	10,453	99,387	2068681	130165	0,000000	M	9,856	11,275	99,387
2	14,720	0,613	12769	581	0,000000	M	14,400	15,189	0,613
Total		100,000	2081449	130746					100,000

for **5a**: *ee* = 99%

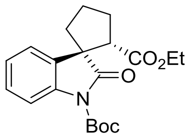

Conditions: IC column
mobile phase: *n*-heptane/*i*-PrOH = 80/20
λ = 208 nm, V = 1.0 ml/min, t = 25 °C
for **9**: t_R = 7.6 min (minor), t_R = 13.9 min (major)

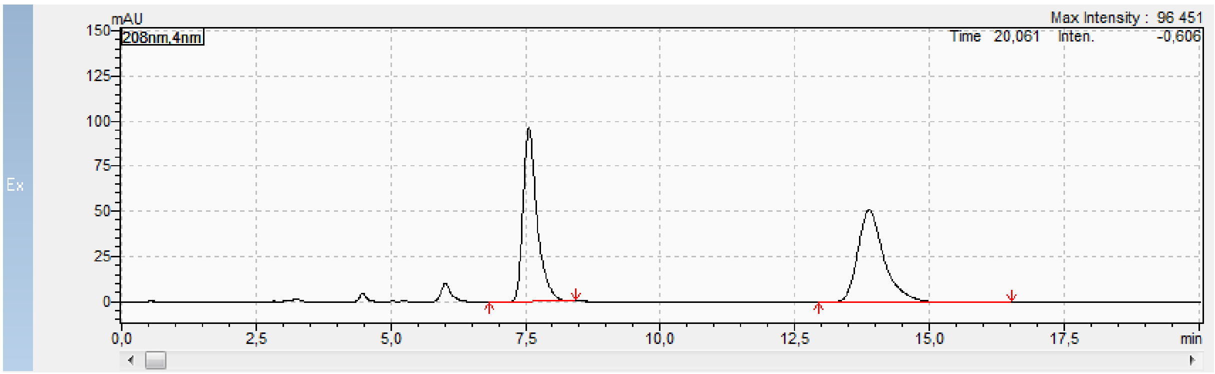

Results View - Peak Table

Peak Table | Compound | Group | Calibration Curve

Peak#	Ret. Time	Conc.	Area	Height	Similarity Index	Mark	Peak Start	Peak End	Area%
1	7,565	49,999	1715239	96292	0,000000	M	6,837	8,427	49,999
2	13,885	50,001	1715301	51031	0,000000	M	12,939	16,523	50,001
Total		100,000	3430540	147324					100,000

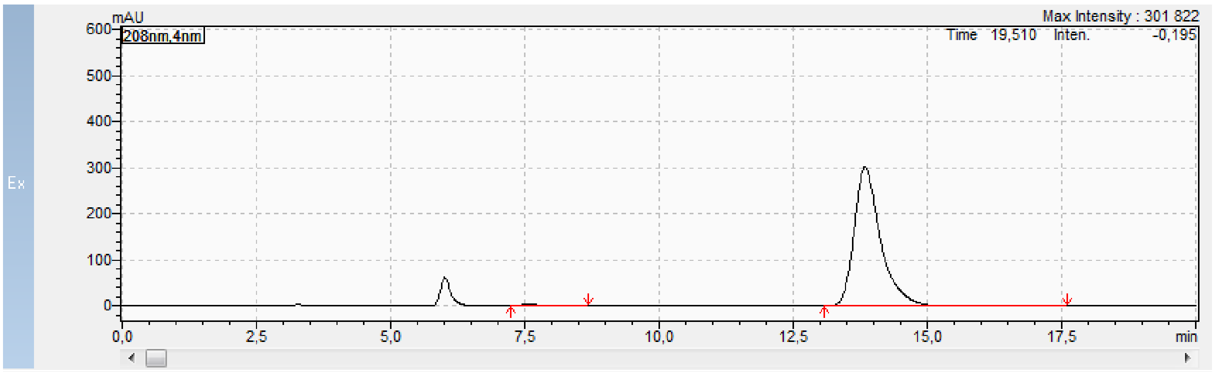

Results View - Peak Table

Peak Table | Compound | Group | Calibration Curve

Peak#	Ret. Time	Conc.	Area	Height	Similarity Index	Mark	Peak Start	Peak End	Area%
1	7,567	0,808	83778	4546	0,000000		7,243	8,693	0,808
2	13,848	99,192	10286707	301955	0,000000	SV	13,077	17,621	99,192
Total		100,000	10370485	306501					100,000

for **9**: *ee* = 98%

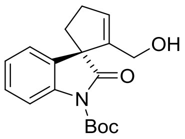

Conditions: IC column
mobile phase: *n*-heptane/*i*-PrOH = 80/20
λ = 240 nm, V = 1.0 ml/min, t = 25 °C
for **10**: t_R = 8.0 min (minor), t_R = 10.9 min (major)

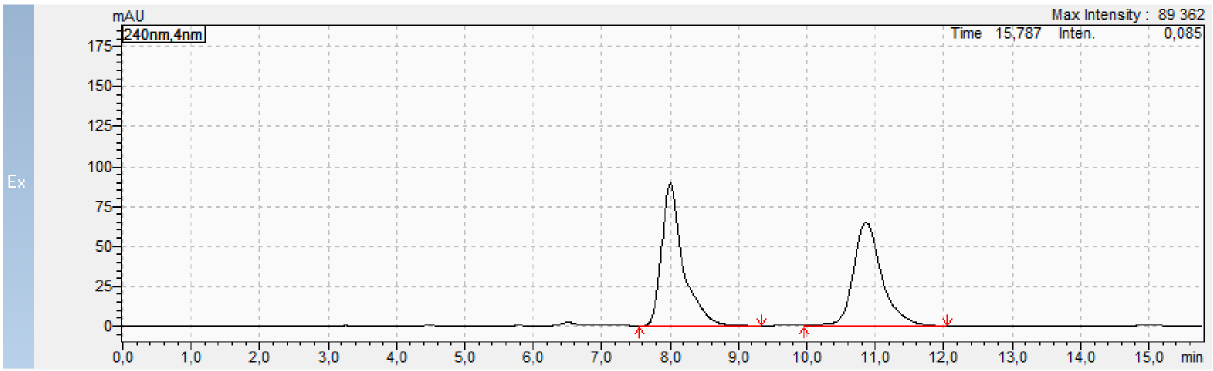

Results View - Peak Table

Peak Table | Compound | Group | Calibration Curve

Peak#	Ret. Time	Conc.	Area	Height	Similarity Index	Mark	Peak Start	Peak End	Area%
1	8,001	51,058	1950529	89148	0,000000		7,552	9,333	51,058
2	10,864	48,942	1869720	64870	0,000000		9,952	12,053	48,942
Total		100,000	3820249	154018					100,000

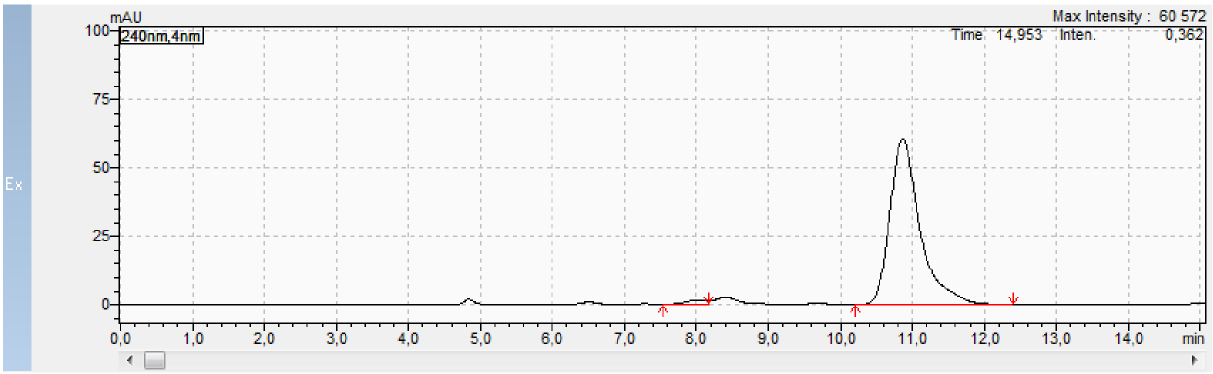

Results View - Peak Table

Peak Table | Compound | Group | Calibration Curve

Peak#	Ret. Time	Conc.	Area	Height	Similarity Index	Mark	Peak Start	Peak End	Area%
1	8,015	1,815	32719	1680	0,000000		7,541	8,160	1,815
2	10,866	98,185	1769597	60539	0,000000		10,208	12,395	98,185
Total		100,000	1802316	62219					100,000

for **10**: *ee* = 96%

References

(1) Sheldrick, G. M. SHELXT - Integrated Space-Group and Crystal-Structure Determination. *Acta Crystallogr. Sect. A Found. Crystallogr.* **2015**, *71*, 3–8.

(2) Sheldrick, G. M. Crystal Structure Refinement with SHELXL. *Acta Crystallogr. Sect. C Struct. Chem.* **2015**, *71*, 3–8.
