# Supplementary figures and images for: Access to Spirooxindole-Fused Cyclopentanes via a Stereoselective Organocascade Reaction Using Bifunctional Catalysis
Source: J Org Chem. 2023 Jan 27;88(12):7724–35. doi: 10.1021/acs.joc.2c02478 (PMC10278142; doi:10.1021/acs.joc.2c02478)

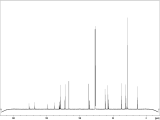

Supplement: Supplementary file 3 — jo2c02478_si_003.zip [file jo2c02478_si_003.zip › 3l_4l_R1=Boc,R2=5-Cl,R3=CO2Et/2/pdata/1/thumb.png]

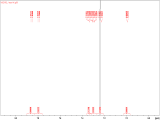

Supplement: Supplementary file 3 — jo2c02478_si_003.zip [file jo2c02478_si_003.zip › 3l_4l_R1=Boc,R2=5-Cl,R3=CO2Et/27/pdata/1/thumb.png]

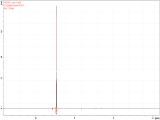

Supplement: Supplementary file 3 — jo2c02478_si_003.zip [file jo2c02478_si_003.zip › 3l_4l_R1=Boc,R2=5-Cl,R3=CO2Et/30/pdata/1/thumb.png]

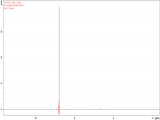

Supplement: Supplementary file 3 — jo2c02478_si_003.zip [file jo2c02478_si_003.zip › 3l_4l_R1=Boc,R2=5-Cl,R3=CO2Et/33/pdata/1/thumb.png]
